# Efficient construction of functionalized pyrroloindolines through cascade radical cyclization/intermolecular coupling†

**DOI:** 10.1039/d3sc05210a

**Published:** 2024-01-04

**Authors:** Yonggang Jiang, Dongxiang Liu, Lening Zhang, Cuirong Qin, Hui Li, Haitao Yang, Patrick J. Walsh, Xiaodong Yang

**Affiliations:** a Key Laboratory of Medicinal Chemistry for Natural Resources, Ministry of Education, Yunnan Provincial Center for Research & Development of Natural Products, School of Pharmacy, Yunnan University Kunming 650091 P. R. China xdyang@ynu.edu.cn; b Roy and Diana Vagelos Laboratories, Penn/Merck Laboratory for High-Throughput Experimentation, Department of Chemistry, University of Pennsylvania 231 South 34th Street Philadelphia Pennsylvania 19104 USA

## Abstract

Pyrroloindolines are important structural units in nature and the pharmaceutical industry, however, most approaches to such structures involve transition-metal or photoredox catalysts. Herein, we describe the first tandem SET/radical cyclization/intermolecular coupling between 2-azaallyl anions and indole acetamides. This method enables the transition-metal-free synthesis of C3a-substituted pyrroloindolines under mild and convenient conditions. The synthetic utility of this transformation is demonstrated by the construction of an array of C3a-methylamine pyrroloindolines with good functional group tolerance and yields. Gram-scale sequential one-pot synthesis and hydrolysis reactions demonstrate the potential synthetic utility and scalability of this approach.

## Abstract 2

A novel tandem radical cyclization/intermolecular coupling between 2-azaallyl anions and indole *N*-aryloxy acetamides is developed, which enables the synthesis of C3a-substituted pyrroloindolines under mild conditions.

## Graphical abstract

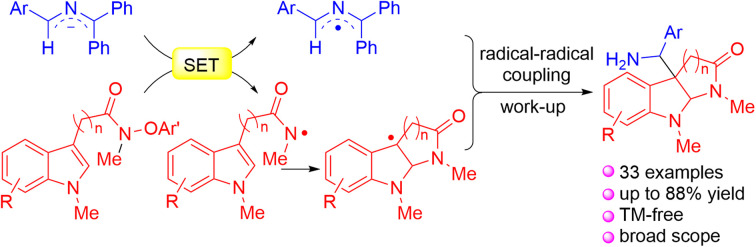

## Introduction

**Fig. 1:**
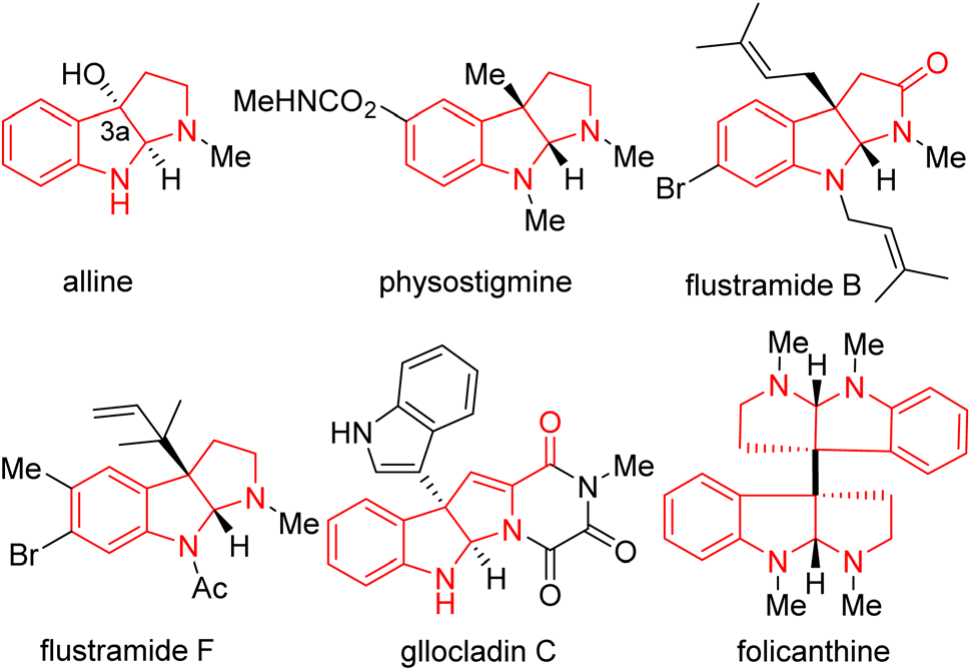
Representative C3a-substituted pyrroloindoline natural products.

Nitrogen-containing heterocycles are among the most fundamental structural motifs in organic compounds.^1–7^ Among N-heterocycles, pyrroloindoline alkaloids (also termed hexahydropyrrolo[2,3-*b*]indoles), particularly C3a-substituted pyrroloindolines, are an important subclass in nature and the pharmaceutical industry (such as alline, physostigmine, flustramide B and F, gllocladin C, folicanthine, Fig. 1).^8–12^ These molecules have gained interest owing to their extensive biological activities, including antitumor, antimicrobial, antinematodal, vasodilating activities, as well as inhibitory activities against cholinesterases and topoisomerases.^13,14^ Consequently, the efficient and straightforward construction of functionalized pyrroloindolines remains in demand.

**Scheme 1:**
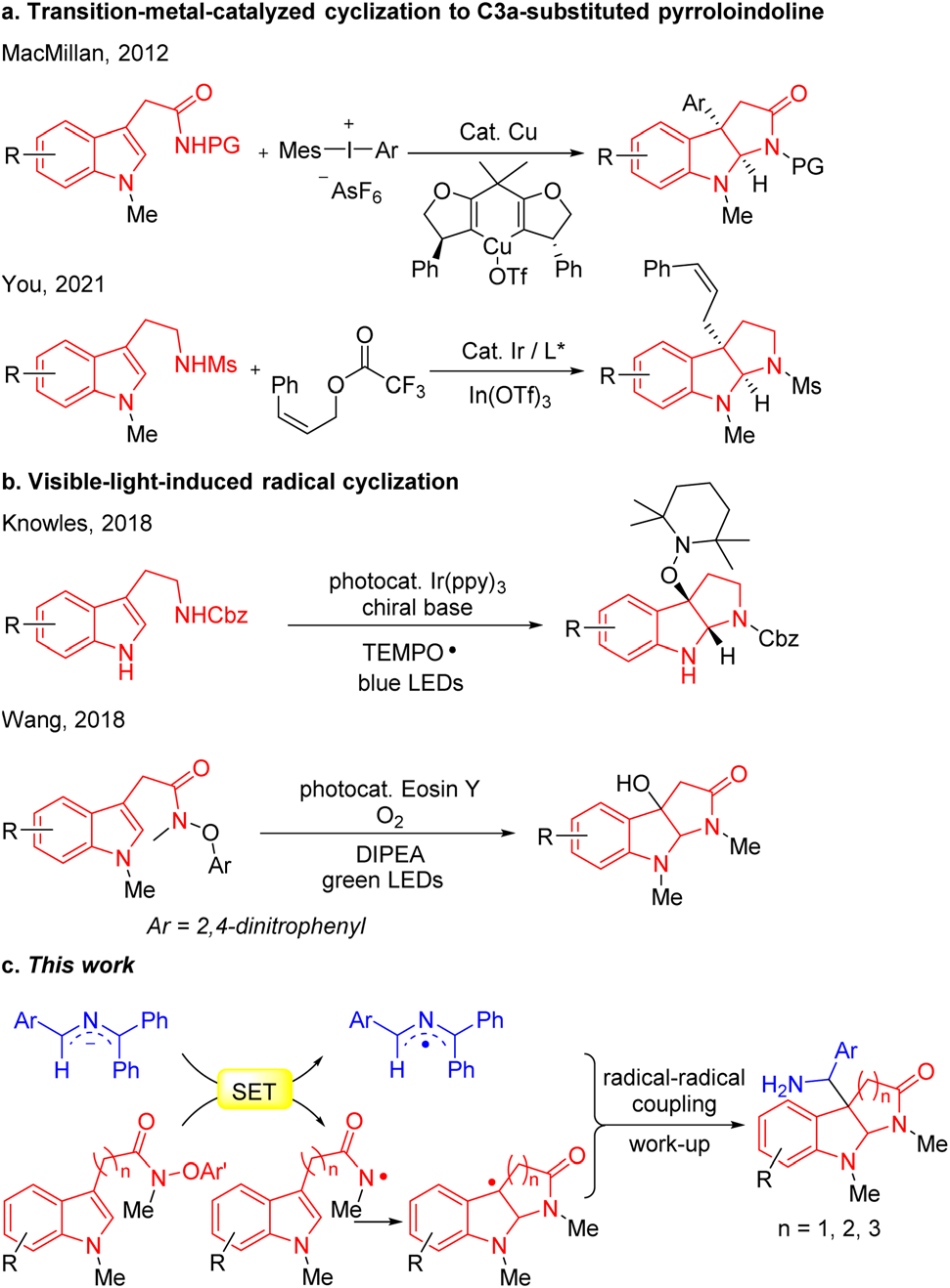
(a) Transition-metal-catalyzed cyclization to C3a-substituted pyrroloindoline. (b) Visible-light-induced radical cyclization to C3a-OH pyrroloindoline. (c) SED 2-azaallyl anions enable synthesis of C3a-substituted pyrroloindolines (this work).

The classical approach to C3a-substituted pyrroloindolines involves the transition-metal-catalyzed cyclization (Scheme 1a). For example, MacMillan's group developed the copper-catalyzed arylation/cyclization cascade of indole acetamides with diaryliodonium salts to access enantioenriched C3a-aryl pyrroloindolines,^15^ while You reported an elegant iridium-catalyzed allylation of tryptamines with *Z*-cinnamyl acetate to obtain Z-retentive C3a-allyl pyrroloindolines.^16^ Recently, visible-light- mediated radical cyclizations have emerged as potent approaches for the construction of heterocycles,^17–27^ as presented in the representative illustrations in Scheme 1b. Knowles developed a Ir(ppy)_3_ photocatalytic proton-coupled electron transfer reaction for synthesis of C3a-TEMPO-substituted pyrroloindolines,^28^ and Wang reported a eosin Y visible-light-induced radical cascade reaction of indole acetamides to access C3a-hydroxypyrroloindolines.^29^ Most of these protocols, however, involve transition-metal catalysts or photoredox catalysts.

Since the pioneering studies by Murphy,^30–32^ super electron donors (SEDs), that is neutral and anionic organic compounds that exhibit strong reducing tendencies through single-electron transfer (SET), have emerged as an effective partner in radical–radical couplings to form C–C bonds. In particular, 2-azaallyl anions, which possess the ability to behave as strong single electron reducing agents, have attracted attention in the synthetic community.^33–37^ Based on the SED properties of 2-azaallyl anions, our team developed a series of tandem cyclization reactions to construct benzofuran, isochromene and isoquinoline derivatives,^38–40^ among others. We further employed 2-azaallyl anions to develop a series of radical C(sp^3^)–C(sp^2^) and C(sp^3^)–C(sp^3^) coupling strategies.^41–47^

In view of the medicinal value of pyrroloindolines, we felt compelled to apply this radical coupling approach to the synthesis of C3a-substituted pyrroloindolines. Based on our prior generation of amidyl radicals,^48^ we hypothesized that SET between the 2-azaallyl anions and indole *N*-aryloxy acetamides would generate 2-azaallyl radicals and amidyl radicals, the latter of which would trigger a radical cyclization to furnish C3a-pyrroloindoline radicals. Finally, coupling between 2-azaallyl radicals and pyrroloindoline radicals was expected to afford C3a-methylamine pyrroloindoline derivatives (Scheme 1c).

Herein, we describe the first tandem SET/radical cyclization/intermolecular coupling between 2-azaallyl anions and indole acetamides, which enables the synthesis of C3a-substituted pyrroloindolines under mild and convenient conditions. The synthetic utility of this transformation is demonstrated by the construction of an array of C3a-methylamine pyrroloindolines with good functional group tolerance and yields (33 examples, up to 88% yield).

## Results and discussion

We initiated the reaction optimization by choosing indole *N*-aryloxy acetamide 2a as the model substrate. Reaction of 2a was performed with *N*-benzylketimine 1a in the presence of base in DMSO at room temperature for 3 h. Initially, a series of bases including LiO^*t*^Bu, NaO^*t*^Bu, KO^*t*^Bu, LiN(SiMe_3_)_2_, NaN(SiMe_3_)_2_ and KN(SiMe_3_)_2_ were evaluated (Table 1, entries 1–6). Among them, NaN(SiMe_3_)_2_ generated the radical cyclization/coupling product 3aa in 89% assay yield (AY, as determined by ^1^H NMR integration against an internal standard) and 86% isolated yield (dr = 1.2 : 1, entry 5), while the other five bases led to product 3aa in 56–65% AY. Next, we then turned our attention to probe the effect of solvent and concentration. Using NaN(SiMe_3_)_2_ as base, we examined a range of solvents [THF, DMF, CPME, MTBE (methyl *tert*-butyl ether) and MeCN]. Surprisingly, no desired products formed in these solvents (entries 7–11). Furthermore, decreasing the concentration to 0.1 M or 0.05 M led to a reduction in the AY to 78% and 68% (entries 12 and 13). When the equiv of base was increased from 1.5 to 2.0 equiv, a decrease in the AY to 72% (entry 14) was observed. Finally, increasing the reaction temperature to 60 °C or decreasing it to 0 °C resulted in a decrease in the AY to 22% or 8%, respectively (entries 15 and 16).

**Table 1:** Optimization of coupling of ketimine 1a and amide 2a^a^^,^^b^

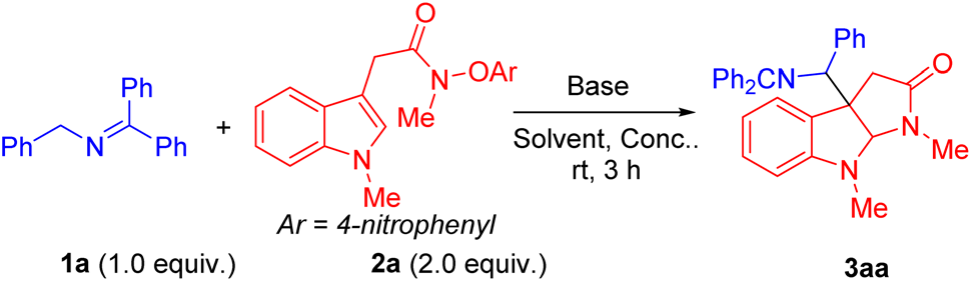				
Entry	Base (equiv.)	Solvent	Conc. [M]	Yield (%)^b^
1	LiO^*t*^Bu (1.5)	DMSO	0.2	56 (dr = 1 : 1)
2	NaO^*t*^Bu (1.5)	DMSO	0.2	61 (dr = 1 : 1)
3	KO^*t*^Bu (1.5)	DMSO	0.2	65 (dr = 1.3 : 1)
4	LiHMDS (1.5)	DMSO	0.2	59 (dr = 1 : 1)
5	NaHMDS (1.5)	DMSO	0.2	89 (86)^c^ (dr = 1.2 : 1)
6	KHMDS (1.5)	DMSO	0.2	65 (dr = 1 : 1)
7	NaHMDS (1.5)	THF	0.2	0
8	NaHMDS (1.5)	DMF	0.2	0
9	NaHMDS (1.5)	CPME	0.2	0
10	NaHMDS (1.5)	MTBE	0.2	0
11	NaHMDS (1.5)	MeCN	0.2	0
12	NaHMDS (1.5)	DMSO	0.1	78 (dr = 1 : 1)
13	NaHMDS (1.5)	DMSO	0.05	68 (dr = 1 : 1)
14	NaHMDS (2.0)	DMSO	0.2	72 (dr = 1 : 1)
15^d^	NaHMDS (1.5)	DMSO	0.1	22 (dr = 1 : 1)
16^e^	NaHMDS (1.5)	DMSO/THF = 1 : 1	0.1	8 (dr = 1 : 1)

a Reaction conditions: 1a (0.1 mmol, 1.0 equiv), 2a (0.2 mmol, 2.0 equiv), base, rt., 3 h.

b Assay yield (AY) determined by ^1^H NMR spectroscopy of the crude reaction mixture using C_2_H_2_Cl_4_ as an internal standard.

c Isolated yield after chromatographic purification.

d 60 °C.

e 0 °C.

With the optimized conditions in hand (Table 1, entry 5), we next focused our attention on exploring the scope of *N*-benzyl ketimines. As shown in Table 2, in general, we found that *N*-benzyl ketimines 1 bearing various substituted *N*-benzyl or *N*-alkyl groups provided C3a-substituted pyrroloindolines in moderate to good yields (48–88%) as a mixture of diastereomers. *N*-Benzyl groups bearing electron-donating substituents 4-OMe (1b) and 3,4-methylenedioxy (1c) generated cyclization products 3ba and 3ca in 56% and 60% yields, respectively. *N*-Benzyl ketimines decorated with electronegative and electron-withdrawing groups, such as 4-F (1d), 4-Cl (1e), 4-Br (1f), 2,4-di-F (1g) and 4-CF_3_ (1h) afforded products 3da, 3ea, 3fa, 3ga, 3ha in 80%, 76%, 66%, 63% and 73% yields, respectively. The structures of products 3ga′ and 3ga′′, which were separable by column chromatography and HPLC, were confirmed by X-ray crystallography (CCDC 2293492 and 2293493). The sterically hindered 2-tolyl (1i) and 1-naphthyl (1j) *N*-benzyl derivatives provided cyclization products 3ia and 3ja in 56% and 68% yields, respectively. Notably, this approach also proved tolerant of medicinally relevant heterocyclic derivatives. *N*-benzyl groups decorated with 3-pyridyl (1k), 2-furanyl (1l) and 2-thiophenyl (1m) substituents furnished the corresponding products 3ka, 3la and 3ma in 62%, 48% and 56% yields, respectively. Furthermore, switching *N*-benzyl ketimine with *N*-(9*H*-fluoren-9-yl)alkylanimine, we could expand the scope of imine substrates to those with *N*-alkyl groups. Methyl (1n), *i*-Pr (1o), isobutyl (1p), cyclobutyl (1q), cyclopentyl (1r) and cyclohexyl (1s) were also suitable substituents, giving the corresponding products (3na-3sa) in 72–88% yields, respectively. Meanwhile, when imines bearing tetraphenyl ketimine and alpha-substituted benzyl amines were employed, the radical cyclization/intermolecular coupling did not take place, likely due to increased steric interactions.

**Table 2:** Scope of Ketimines 1^a^^,^^b^

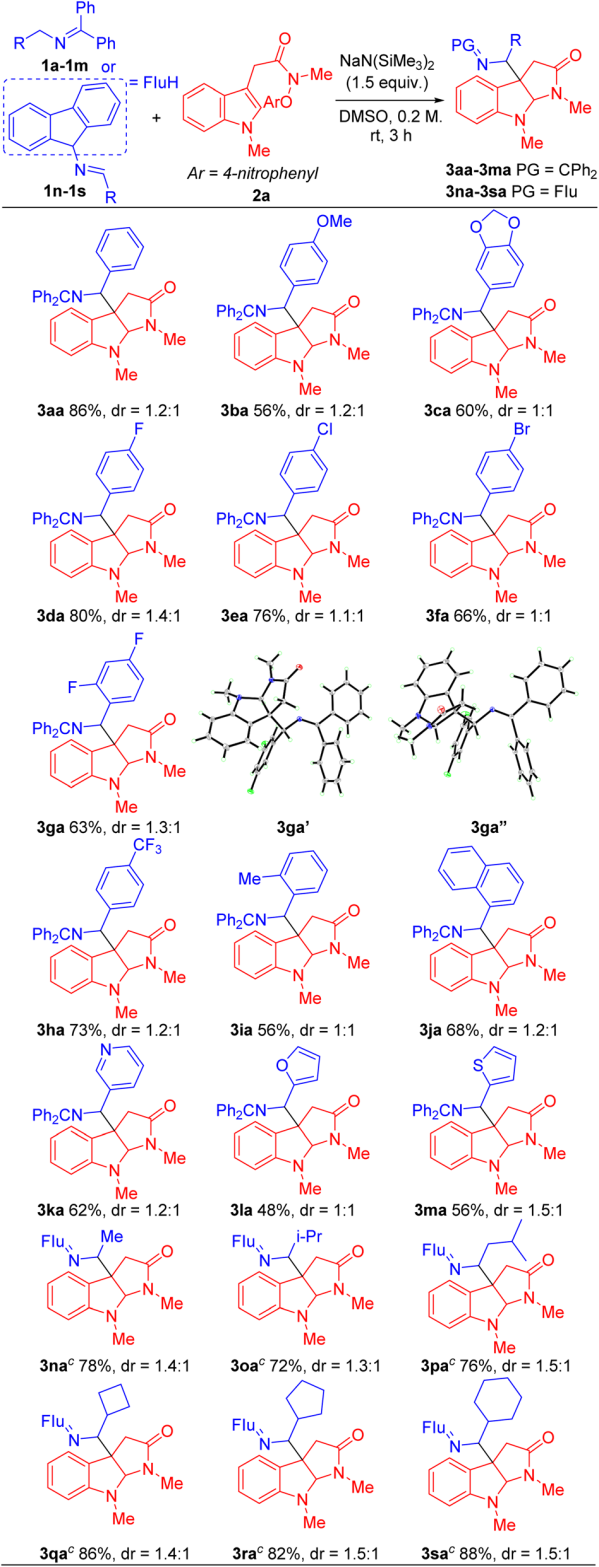

a Reactions conducted on 0.4 mmol scale using 1 equiv of 1a–1s and 2 equiv of 2a.

b Isolated yield after chromatographic purification, Flu = 9-fuorenyl. PG = protect group.

c *N*-(9*H*-Fluoren-9- yl)alkylanimine as the 2-azaallyl precursor.

Next, we evaluated the scope of the indole acetamides 2, which were easily synthesized using the method of Wang^29,49^ (see ESI for details†). A wide range of indole *N*-aryloxy acetamides bearing various groups were all compatible with our method, generating the pyrroloindoline products in moderate to good yields (46–78%, Table 3). For instance, indole derivatives with electron-donating substituents, such as 7-Me (2b), 5-OMe (2c) and 5-OBn (2d), afforded cyclization products 3ab, 3ac and 3ad in 63%, 65% and 60% yields, respectively. Indole acetamides with electronegative substituents, such as 5-F (2e) and 5-Br (2f), provided the corresponding products 3ae and 3af in 58% and 60% yields. It is noteworthy that indole derivatives with a heterocyclic piperonyl group (2g) and sterically hindered naphthyl group (2h) led to coupling products 3ag and 3ah in 78% and 72% yields, respectively. In addition, we explored a range of indole *N*-aryloxy acetamide substrates. Gratifyingly, when we extended the alkyl chain of indole acetamides to two or three methylenes, the corresponding six- and seven-member ring products 3ai and 3aj were obtained in 52% and 46% yields, respectively. Furthermore, we introduced steric hindrance at the C2 position of indole substrates [2-methyl (2k) and 2-ethyl (2l)], leading to cyclization products 3ak and 3al in 56% and 65% yields. Next, we use indole derivatives bearing benzyl (2m), allyl (2n) and Boc (2o) substituents, which afforded cyclization products 3sm, 3sn and 3so in 67, 70 and 56% yields, respectively.

**Table 3:** Scope of indole acetamides 2^a^^,^^b^

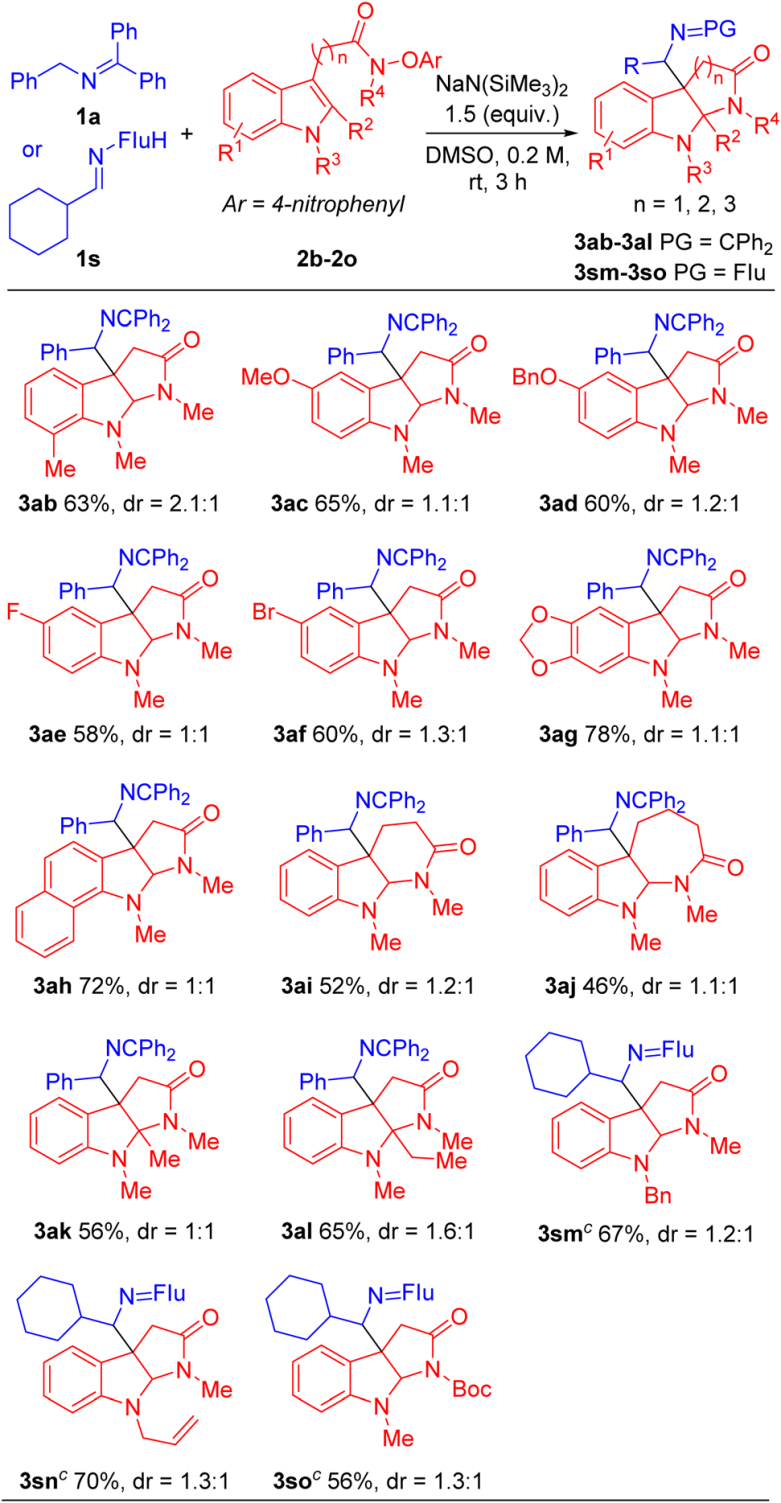

a Reactions conducted on 0.4 mmol scale using 2 equiv of 2b–2o, and 1 equiv of 1a or 1s.

b Isolated yield after chromatographic purification, Flu = 9-fuorenyl. PG = protect group.

c *N*-(9*H*-Fluoren-9- yl)alkylanimine as the 2-azaallylprecursor.

**Scheme 2:**
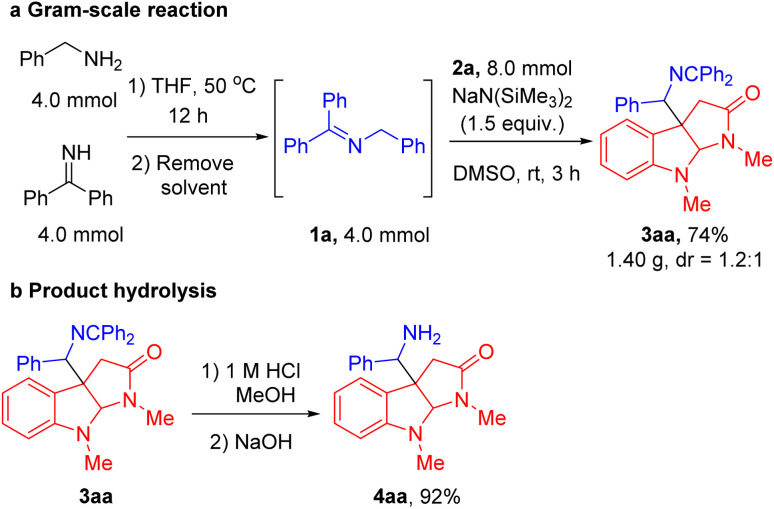
(a) Gram-scale sequential one-pot synthesis. (b) Hydrolysis of the product imine.

To demonstrate the utility and scalability of our cascade radical cyclization/intermolecular coupling reaction, a gram-scale sequential one-pot synthesis and product hydrolysis were conducted. A telescoped gram-scale experiment was performed by employing benzylamine and diphenyl methyl imine in THF at 50 °C for 12 h, followed by solvent removal to afford imine 1a. The unpurified 1a was coupled with indole *N*-aryloxy acetamide 2a under the standard reaction conditions. The product 3aa was obtained in 74% yield (1.40 g, Scheme 2a). Subsequently, imine hydrolysis of the cyclization product 3aa under mildly acidic conditions furnished the free C3a-methylamine pyrroloindoline derivative 4aa in excellent yield (92%, Scheme 2b).

**Scheme 3:**
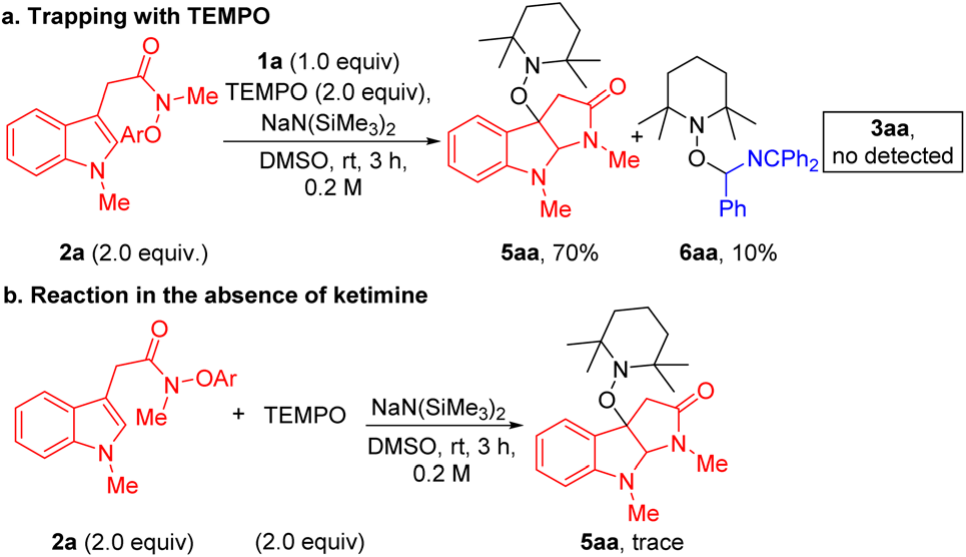
Control experiments. (a) Radical trapping experiment. (b) Reaction in the absence of ketimine.

Finally, to gain some information on the reaction mechanism, we carried out control experiments. First, the experiment with the addition of 2.0 equiv of radical scavenger 2,2,6,6-tetramethylpiperidine-1-oxyl (TEMPO) was conducted under the standard conditions. However, no desired product 3aa was detected, only affording TEMPO trapping compounds 5aa and 6aa in 70% and 10% yields (Scheme 3a). A control experiment with 2.0 equiv of TEMPO in the absence of *N*-benzyl ketimine 1a was carried out under the standard conditions, and radical coupling product 5aa was not observed (Scheme 3b). The lack of product formation indicates that NaN(SiMe_3_)_2_ is not the active reductant in this chemistry. Together, these results suggest that the reaction proceeds *via* a radical pathway, supporting the key SET/radical cyclization/coupling pathway proposed in Scheme 1c.

**Scheme 4:**
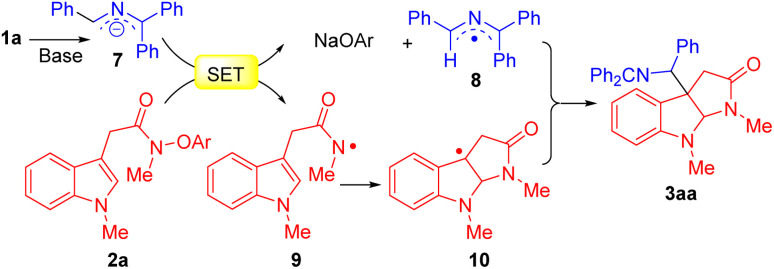
Proposed reaction pathway.

A plausible mechanism for the reaction is outlined in Scheme 4. Ketimine 1a is deprotonated by the NaN(SiMe_3_)_2_ to afford the 2-azaallyl anion 7. Next, SED 7 undergoes an SET process with acetamides 2a to form azaallyl radical 8 and *N*-centered radical 9. The amidyl radical 9 initiates a radical cyclization to generate C3a-pyrroloindoline radical 10. Finally, pyrroloindoline radical 10 couples with 2-azaallyl radical 8 to obtain coupling product 3aa.

## Conclusions

In summary, we have developed a unique strategy for constructing functionalized pyrroloindolines in a single synthetic step. Unlike many previous reports, which generally involve transition-metal catalysts or photoredox catalysts, this chemistry utilizes readily generated SED, 2-azaallyl anions. In this transformation, the tandem SET/radical cyclization/intermolecular coupling between 2-azaallyl anions and indole *N*-aryloxy acetamides provides the functionalized pyrroloindolines related to biologically active compounds by simple combination of base and DMSO at room temperature. A gram-scale sequential one-pot synthesis and hydrolysis reaction demonstrate the potential synthetic utility and scalability of this approach. It is noteworthy that this method includes a multistep tandem reaction with a rapid increase in molecular complexity. The sustainability of this method enhances its potential utility in the pharmaceutical industry.^50^

## Data availability

All experimental data, procedures for data analysis, and pertinent data sets are provided in the ESI.†

## Author contributions

X. Y. and Y. J. conceived of the project. X. Y. and P. J. W. designed the experiments. Y. J., D. L., L. Z., C. Q., H. L. and H. Y. performed the research. X. Y. and P. J. W. wrote the manuscript.

## Conflicts of interest

There are no conflicts to declare.

## Supplementary Material

SC-015-D3SC05210A-s001

SC-015-D3SC05210A-s002
