# Supplementary material for: Efficient construction of functionalized pyrroloindolines through cascade radical cyclization/intermolecular coupling
Source: Chem Sci. 2024 Jan 4;15(6):2205–10. doi: 10.1039/d3sc05210a (PMC10848758; doi:10.1039/d3sc05210a)
Supplement: SC-015-D3SC05210A-s001 [file SC-015-D3SC05210A-s001.pdf]

Electronic Supplementary Material (ESI) for Chemical Science.
This journal is © The Royal Society of Chemistry 2024

Electronic Supplementary Information (ESI)

Efficient Construction of Functionalized Pyrroloindolines through Cascade Radical Cyclization/Intermolecular Coupling

Yonggang Jiang,† Dongxiang Liu,† Lening Zhang,† Cuirong Qin,† Hui Li,† Haitao Yang,† Patrick J. Walsh,*,‡ and Xiaodong Yang*,†

†Key Laboratory of Medicinal Chemistry for Natural Resource, Ministry of Education; Yunnan Provincial Center for Research & Development of Natural Products; School of Pharmacy, Yunnan University, Kunming, 650091, P. R. China. *e-mail: xdyang@ynu.edu.cn

‡Roy and Diana Vagelos Laboratories, Penn/Merck Laboratory for High-Throughput Experimentation, Department of Chemistry, University of Pennsylvania, 231 South 34th Street, Philadelphia, Pennsylvania, 19104, USA. *e-mail: pwalsh@sas.upenn.edu

TABLE OF CONTIENT

General methods 2
Preparation of ketimines 2
General procedure for preparation of amides 2
Product Characterization: 11
Gram scale synthesis of **3aa** 45
Imine product hydrolysis 46
Mechanistic study 47
X-ray crystal structures of compound **3ga’** 48
X-ray crystal structures of compound **3ga”** 48
Supplementary references 49
NMR Spectra 50
HRMS data 125

Supplementary Methods:

General methods

All air- and moisture-sensitive solutions and chemicals were handled under a nitrogen atmosphere in a glovebox and solutions were transferred via "Eppendorf" brand pipettor. Anhydrous solvents were purchased from Sigma-Aldrich and used without further purification. Unless otherwise stated, all reagents were commercially available and used as received without further purification. Chemicals were obtained from Sigma-Aldrich, Acros, TCI and Alfa-Aesar. TLC was performed with Merck TLC Silica gel60 F_{254} plates with detection under UV light at 254 nm. Silica gel (200-300mesh, Qingdao) was used for flash chromatography. Deactivated silica gel was prepared by addition of 15 mL Et_3N to 1 L of silica gel. The products were purified with XDB-C_{18} (9.4 × 250 mm, 5 μm) column on an Agilent HPLC 1260 system. Proton nuclear magnetic resonance (^{1}H-NMR) spectra were recorded on a Bruker DRX 400, Bruker DRX 500 & Bruker DRX 600 spectrometer at 400, 500 or 600 MHz. Carbon-13 nuclear magnetic resonance (^{13}C-NMR) were recorded on Bruker DRX 400, Bruker DRX 500 or Bruker DRX 600 spectrometer at 100, 125 or 150 MHz. Chemical shifts were reported in units of parts per million (ppm) downfield from tetramethylsilane (TMS), and all coupling constants were reported in hertz. The infrared (IR) spectra were measured on a Nicolet iS10 FTIR spectrometer with 4 cm^{-1} resolution and 32 scans between wave number of 4000 cm^{-1} and 400 cm^{-1}. High Resolution Mass spectra were taken on AB QSTAR Pulsar mass spectrometer. Melting points were obtained on a XT-4 melting-point apparatus and were uncorrected.

Preparation of ketimines

Ketimines (**1a-1m**) were prepared according to the literature procedure[1].

N-fluorenyl imines (**1n-1s**) were prepared according to the literature procedure.[2]

General procedure for preparation of amides

The hydroxamic acid **S3** was prepared according to the literature procedure.[3]

General procedure for the synthesis of amides (2a-2n) from hydroxamic acids S3 – GP1

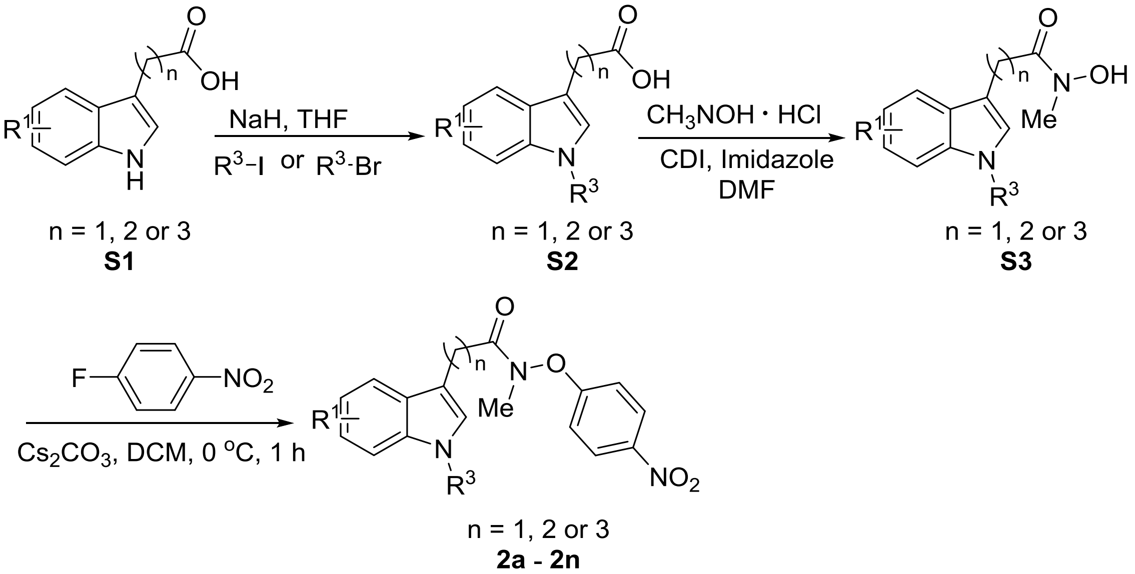

Following the literature procedure[3] with slight modification, in a dry Schlenk tube equipped with a stirring bar the hydroxamic acid **S3** (1.0 equiv) was added, dissolved in anhydrous DCM (0.2 M), cooled to 0 °C and stirred for 5 minutes before Cs_2CO_3 (2.0 equiv) and 1-fluoro -4-nitrobenzene (1.5 equiv) was added in one portion and the reaction mixture was allowed to stir for 1 hour at 0 °C and then quenched with water. The aqueous layer was extracted with DCM (3 X 20 mL) and the combined organic layers were dried (Na_2SO_4), filtered and evaporated under reduced pressure. The product was purified by column chromatography on silica gel eluting with (petroleum ether: ethyl acetate = 5:1) to give **2a-2l**. [**Note**: In the above reaction process, amide **2a-2n** is obtained as well as recovered **S3**, which can be recycled repeatedly to increase the overall material throughput of the indole acetamides product.]

_N_-methyl-2-(1-methyl-1_H_-indol-3-yl)-_N_-(4-nitrophenoxy)acetamide (2a)

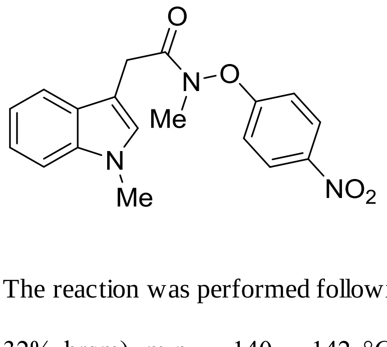

The reaction was performed following **GP1**, compound **2a** was obtained as a yellow solid (22% yield, 32% brsm). m.p. = 140 – 142 °C; R_f = 0.50 (hexanes:ethyl acetate = 1:1); ^1H NMR (400 MHz, Chloroform-*d*) δ 8.11 (d, *J* = 9.2 Hz, 2H), 7.49 (d, *J* = 8.0 Hz, 1H), 7.20 (d, *J* = 4.4 Hz, 2H), 7.09 – 7.05 (m, 1H), 6.94 (d, *J* = 9.2 Hz, 2H), 6.82 (s, 1H), 3.85 (s, 2H), 3.62 (s, 3H), 3.31 (s, 3H) ppm; $^{13}C\{^1H\}$

NMR (100 MHz, Chloroform-*d*) δ 174.8, 162.5, 143.3, 136.7, 127.9, 127.6, 125.8, 121.9, 119.3, 118.8, 113.2, 109.3, 106.0, 35.3, 32.6, 30.3 ppm; IR (thin film): 3077, 2933, 1683, 1590, 1518, 1344, 1111, 863, 748 cm^{-1}; HRMS calc'd for $C_{18}H_{18}N_3O_4^+$: 340.1292, found: 340.1291 $[M+H]^+$.

2-(1, 7-dimethyl-1*H*-indol-3-yl)-*N*-methyl-*N*-(4-nitrophenoxy)acetamide (2b)

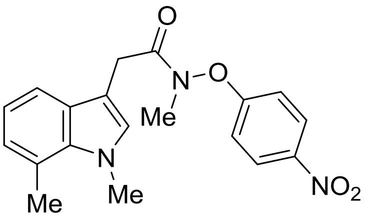

The reaction was performed following **GP1**, compound **2b** was obtained as a yellow solid (21% yield, 64% brsm). m.p. = 146 – 148 °C; R_f = 0.53 (hexanes:ethyl acetate = 1:1); ^{1}H NMR (400 MHz, Chloroform-*d*) δ 8.02 (d, *J* = 9.2 Hz, 2H), 7.21 (d, *J* = 8.0 Hz, 1H), 6.88 – 6.83 (m, 3H), 6.78 (d, *J* = 7.2 Hz, 1H), 6.62 (s, 1H), 3.79 (s, 3H), 3.73 (s, 2H), 3.23 (s, 3H), 2.58 (s, 3H) ppm; $^{13}C\{^1H\}$ NMR (100 MHz, Chloroform-*d*) δ 174.8, 162.5, 143.3, 135.4, 129.5, 128.6, 125.7, 124.5, 121.3, 119.6, 116.8, 113.2, 105.7, 36.5, 35.3, 30.3, 19.5 ppm; IR (thin film): 3078, 2929, 1683, 1590, 1519, 1344, 1111, 862, 747 cm^{-1}, HRMS calc'd for $C_{19}H_{20}N_3O_4^+$: 354.1448, found: 354.1446 $[M+H]^+$.

2-(5-methoxy-1-methyl-1*H*-indol-3-yl)-*N*-methyl-*N*-(4-nitrophenoxy)acetamide (2c)

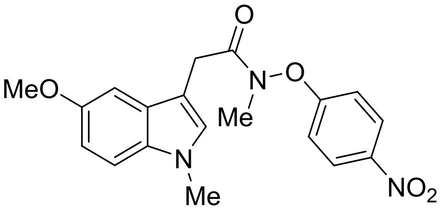

The reaction was performed following **GP1**, compound **2c** was obtained as a yellow solid (27% yield, 45% brsm). m.p. = 136 – 138 °C; R_f = 0.40 (hexanes:ethyl acetate = 1:1); ^{1}H NMR (400 MHz, Chloroform-*d*) δ 8.04 (d, *J* = 9.2 Hz, 2H), 7.01 (d, *J* = 8.8 Hz, 1H), 6.88 – 6.84 (m, 3H), 6.79 – 6.76 (m, 1H), 6.68 (s, 1H), 3.76 (s, 3H), 3.75 (s, 2H), 3.50 (s, 3H), 3.24 (s, 3H) ppm; $^{13}C\{^1H\}$ NMR (100 MHz, Chloroform-*d*) δ 173.8, 161.4, 153.0, 142.3, 131.1, 127.5, 126.8, 124.7, 112.2, 111.0, 109.0, 104.3, 99.8, 54.9, 34.2, 31.7, 29.4 ppm; IR (thin film): 3079, 2935, 1682, 1590, 1519, 1112, 863, 750 cm^{-1}, HRMS calc'd for $C_{19}H_{20}N_3O_5^+$: 370.1397, found: 370.1397 $[M+H]^+$.

2-(5-(benzyloxy)-1-methyl-1*H*-indol-3-yl)-*N*-methyl-*N*-(4-nitrophenoxy)acetamide (2d)

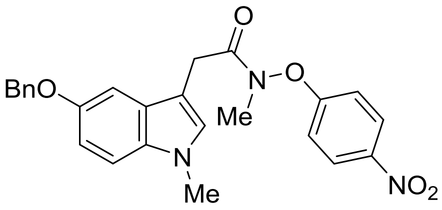

The reaction was performed following **GP1**, compound **2d** was obtained as a yellow solid (34% yield, 49% brsm). m.p. = 146 – 148 °C; R_f = 0.41 (hexanes:ethyl acetate = 1:1); ^{1}H NMR (600 MHz, Chloroform-*d*) δ 7.98 (s, 2H), 7.40 – 7.18 (m, 5H), 6.99 – 6.66 (m, 6H), 5.01 (s, 2H), 3.73 (s, 2H), 3.47 (s, 3H), 3.21 (s, 3H). ^{13}C{^{1}H} NMR (150 MHz, Chloroform-*d*) δ 162.4, 153.1, 143.7, 137.7, 132.3, 128.6, 127.8, 127.6, 126.2, 125.7, 113.2, 112.8, 115.7, 115.0, 110.0, 105.4, 102.5, 70.9, 35.2, 32.7, 30.6 ppm. IR (thin film): 3048, 2997, 1675, 1591, 1519, 1337, 1275, 952, 734 cm^{-1}, HRMS calc'd for $C_{25}H_{24}N_3O_5^+$: 446.1710, found: 446.1713 [M+H]$^+$.

2-(5-fluoro-1-methyl-1*H*-indol-3-yl)-*N*-methyl-*N*-(4-nitrophenoxy)acetamide (2e)

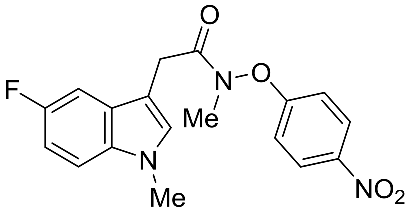

The reaction was performed following **GP1**, compound **2e** was obtained as a yellow solid (36% yield, 53% brsm). m.p. = 66 – 68 ℃; R_f = 0.43 (hexanes:ethyl acetate = 1:1); ^{1}H NMR (400 MHz, Chloroform-*d*) δ 8.06 (d, *J* = 9.2 Hz, 2H), 7.06 – 7.00 (m, 2H), 6.90 (dt, *J* = 9.2, 3.6 Hz, 2H), 6.84 (td, *J* = 9.2, 2.4 Hz, 1H), 6.79 (s, 1H), 3.72 (s, 2H), 3.54 (s, 3H), 3.25 (s, 3H) ppm; ^{13}C{^{1}H} NMR (100 MHz, Chloroform-*d*) δ 173.5, 161.3, 156.8 (d, $^1J_{C\text{-}F}$ = 234.5 Hz), 142.4, 132.3, 128.6, 126.8 (d, $^3J_{C\text{-}F}$ = 9.9 Hz), 125.0, 124.8, 114.5, 112.2, 109.2 (d, $^2J_{C\text{-}F}$ = 26.0 Hz), 109.0 (d, $^3J_{C\text{-}F}$ = 9.6 Hz), 104.9 (d, $^4J_{C\text{-}F}$ = 4.7 Hz), 102.7 (d, $^2J_{C\text{-}F}$ = 21.6Hz), 34.2, 31.8, 29.1 ppm; ^{19}F NMR (377 MHz, Chloroform-*d*) δ -126.11 ppm; IR (thin film): 3113, 2929, 1683, 1590, 1519, 1344, 1291, 913, 749 cm^{-1}, HRMS calc'd for $C_{18}H_{17}FN_3O_4^+$: 358.1198, found: 358.1197 [M+H]$^+$.

2-(5-bromo-1-methyl-1*H*-indol-3-yl)-*N*-methyl-*N*-(4-nitrophenoxy)acetamide (2f)

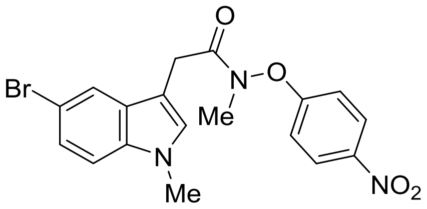

The reaction was performed following **GP1**, compound **2f** was obtained as a yellow solid (29% yield, 48% brsm). m.p. = 62 – 64 °C; R_f = 0.40 (hexanes:ethyl acetate = 1:1); ^{1}H NMR (400 MHz, Chloroform-*d*) δ 8.08 (d, *J* = 9.2 Hz, 2H), 7.46 (d, *J* = 2.0 Hz, 1H), 7.17 (t, *J* = 6.8 Hz, 1H), 7.00 (d, *J* = 8.8 Hz, 1H), 6.92 (dt, *J* = 9.6, 3.2 Hz, 2H), 6.81 (s, 1H), 3.72 (s, 2H), 3.56 (s, 3H), 3.25 (s, 3H) ppm; ^{13}C{^{1}H} NMR (100 MHz, Chloroform-*d*) δ 170.1, 161.3, 142.5, 134.3, 128.2, 128.1, 124.9, 123.7, 120.3, 112.2, 111.7, 109.7, 104.7, 34.2, 31.8, 28.8 ppm; IR (thin film): 3112, 2926, 1683, 1590, 1519, 1344, 1216, 1111, 913, 748 cm^{-1}, HRMS calc'd for $C_{18}H_{17}BrN_3O_4^+$: 418.0397, found: 418.0399 [M+H]$^+$.

***N*-methyl-2-(5-methyl-5*H*-[1,3]dioxolo[4,5-*f*]indol-7-yl)-*N*-(4-nitrophenoxy)acetamide (2g)**

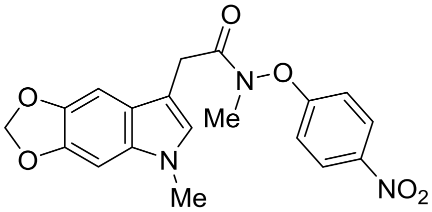

The reaction was performed following **GP1**, compound **2g** was obtained as a yellow solid (27% yield, 50% brsm). m.p. = 112 – 114 °C; R_f = 0.40 (hexanes:ethyl acetate = 1:1); ^{1}H NMR (400 MHz, Chloroform-*d*) δ 8.05 (d, *J* = 9.2 Hz, 2H), 6.88 (d, *J* = 9.2 Hz, 2H), 6.77 (s, 1H), 6.57 (s, 2H), 5.83 (s, 2H), 3.68 (s, 2H), 3.44 (s, 3H), 3.23 (s, 3H) ppm; ^{13}C{^{1}H} NMR (100 MHz, Chloroform-*d*) δ 174.7, 162.5, 145.1, 143.4, 142.8, 132.0, 126.5, 125.8, 121.5, 113.2, 106.1, 100.7, 97.5, 90.2, 35.2, 32.9, 30.4 ppm; IR (thin film): 3072, 2918, 1681, 1589, 1373, 1240, 1101, 863, 750 cm^{-1}, HRMS calc'd for $C_{19}H_{18}N_3O_6^+$: 384.1190, found: 384.1187 [M+H]$^+$.

***N*-methyl-2-(1-methyl-1*H*-benzo[*g*]indol-3-yl)-*N*-(4-nitrophenoxy)acetamide (2h)**

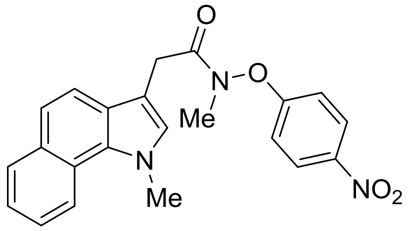

The reaction was performed following **GP1**, compound **2h** was obtained as a yellow solid (31% yield, 49% brsm). m.p. = 190 – 192 °C; R_f = 0.40 (hexanes:ethyl acetate = 1:1); ^{1}H NMR (400 MHz, Chloroform-*d*) δ 8.29 (d, *J* = 8.4 Hz, 1H), 8.00 (d, *J* = 8.0 Hz, 2H), 7.86 (d, *J* = 8.0 Hz, 1H), 7.49 – 7.41 (m, 3H), 7.36 (dd, *J* = 14.0, 6.0 Hz, 1H), 6.88 (d, *J* = 9.2 Hz, 2H), 6.78 (s, 1H), 4.06 (s, 3H), 3.85 (s, 2H), 3.25 (s, 3H) ppm; ^{13}C{^{1}H} NMR (100 MHz, Chloroform-*d*) δ 172.9, 161.3, 142.3, 130.3, 129.0, 128.1, 127.1, 124.7, 124.4, 123.7, 122.5, 122.2, 119.9, 119.4, 117.5, 112.1, 105.8, 37.3, 34.2, 29.2 ppm; IR (thin film): 3060, 2964, 1685, 1589, 1460, 1344, 1110, 913, 748 cm^{-1}, HRMS calc'd for $C_{22}H_{20}N_3O_4^+$: 390.1448, found: 390.1450 [M+H]$^+$.

N-methyl-3-(1-methyl-1H-indol-3-yl)-N-(4-nitrophenoxy)propanamide (2i)

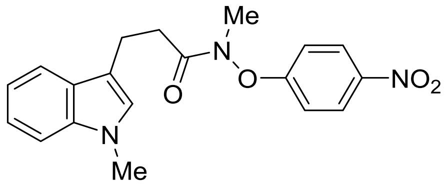

The reaction was performed following **GP1**, compound **2i** was obtained as a yellow oil (28% yield, 51% brsm). R_f = 0.48 (hexanes:ethyl acetate = 1:1); ^{1}H NMR (400 MHz, Chloroform-*d*) δ 8.00 (d, *J* = 9.2 Hz, 2H), 7.33 (d, *J* = 8.0 Hz, 1H), 7.17 (t, *J* = 8.4 Hz, 1H), 7.09 (t, *J* = 8.4 Hz, 1H), 6.92 (t, *J* = 7.6 Hz, 1H), 6.81 (d, *J* = 9.2 Hz, 2H), 6.74 (s, 1H), 3.62 (s, 3H), 3.21 (s, 3H), 3.01 (t, *J* = 7.2 Hz, 2H), 2.65 (t, *J* = 7.2 Hz, 2H) ppm; ^{13}C{^{1}H} NMR (100 MHz, Chloroform-*d*) δ 175.0, 161.3, 142.4, 135.9, 132.3, 126.4, 125.5, 125.0, 120.5, 117.7, 117.6, 112.1, 108.2, 34.0, 32.4, 31.5, 19.1 ppm; IR (thin film): 3004, 2981, 1671, 1590, 1455, 1345, 1055, 737 cm^{-1}; HRMS calc'd for $C_{19}H_{20}N_3O_4^+$: 354.1448, found: 354.1449 [M+H]$^+$.

N-methyl-4-(1-methyl-1H-indol-3-yl)-N-(4-nitrophenoxy)butanamide (2j)

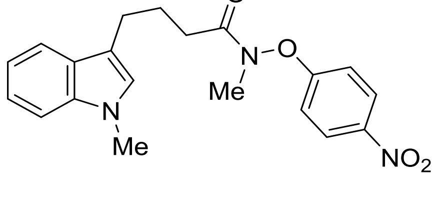

The reaction was performed following **GP1**, compound **2j** was obtained as a yellow oil (24% yield, 44% brsm). R_f = 0.50 (hexanes:ethyl acetate = 1:1); ^{1}H NMR (400 MHz, Chloroform-*d*) δ 8.05 (d, *J* = 9.2 Hz, 2H), 7.41 (d, *J* = 8.0 Hz, 1H), 7.17 – 7.09 (m, 2H), 6.96 (t, *J* = 7.2 Hz, 1H), 6.84 (dt, *J* = 9.2 Hz, 3.6 Hz, 2H), 6.65 (s, 1H), 3.57 (s, 3H), 3.20 (s, 3H), 2.67 (t, *J* = 7.2 Hz, 2H), 2.30 (t, *J* = 7.6 Hz, 2H), 1.97 – 1.89 (m, 2H) ppm; ^{13}C{^{1}H} NMR (100 MHz, Chloroform-*d*) δ 176.7, 162.5, 143.4, 137.0, 127.8, 126.3, 126.2, 121.5, 118.9, 118.6, 113.9, 113.2, 109.2, 35.1, 32.5, 32.0, 24.8, 24.3 ppm; IR (thin film): 3077, 2931, 1683, 1590, 1519, 1486, 1344, 1112, 742 cm^{-1}, HRMS calc'd for $C_{20}H_{22}N_3O_4^+$: 368.1605, found: 368.1602 $[M+H]^+$.

2-(1,2-dimethyl-1*H*-indol-3-yl)-*N*-methyl-*N*-(4-nitrophenoxy)acetamide (2k)

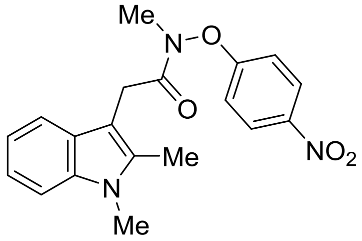

The reaction was performed following **GP1**, compound **2k** was obtained as a yellow solid (40% yield, 58% brsm). m.p. = 144 – 146 °C; R_f = 0.53 (hexanes:ethyl acetate = 1:1); ^{1}H NMR (400 MHz, Chloroform-*d*) δ 7.99 (d, *J* = 9.6 Hz, 2H), 7.44 (d, *J* = 7.6 Hz, 1H), 7.14 – 7.03 (m, 3H), 6.76 (d, *J*= 7.6 Hz, 2H), 3.84 (s, 2H), 3.42 (s, 3H), 3.26 (s, 3H), 2.21 (s, 3H) ppm; ^{13}C{^{1}H} NMR (100 MHz, Chloroform-*d*) δ 175.0, 162.1, 143.2, 136.3, 134.1, 127.4, 125.3, 121.0, 119.3, 117.9, 113.0, 108.6, 102.9, 35.1, 30.6, 29.3, 10.4 ppm; IR (thin film): 3052, 2918, 1682, 1590, 1519, 1344, 1217, 1110, 749 cm^{-1}, HRMS calc'd for $C_{19}H_{20}N_3O_4^+$: 354.1448, found: 354.1448 $[M+H]^+$.

2-(2-ethyl-1-methyl-1*H*-indol-3-yl)-*N*-methyl-*N*-(4-nitrophenoxy)acetamide (2l)

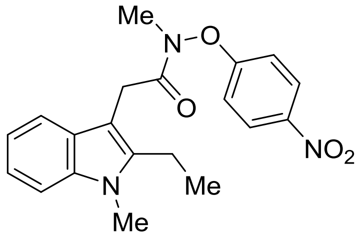

The reaction was performed following **GP1**, compound **2l** was obtained as a yellow solid (37% yield, 53% brsm). m.p. = 98 – 100 °C; R_f = 0.55 (hexanes:ethyl acetate = 1:1); ^{1}H NMR (400 MHz, Chloroform-

d) δ 7.95 (d, *J* = 9.2 Hz, 2H), 7.35 (d, *J* = 8.0 Hz, 1H), 7.06 – 7.03 (m, 2H), 6.99 – 6.95 (m, 1H), 6.73 (d, *J* = 9.2 Hz, 2H), 3.76 (s, 2H), 3.41 (s, 3H), 3.20 (s, 3H), 2.57 (q, *J* = 7.2 Hz, 2H), 1.08 (t, *J* = 7.6 Hz, 3H) ppm; $^{13}C\{^1H\}$ NMR (100 MHz, Chloroform-*d*) δ 174.0, 161.1, 142.1, 138.8, 135.4, 126.4, 124.4, 120.0, 118.3, 117.0, 112.0, 107.7, 101.1, 34.1, 29.2, 28.3, 16.8, 13.2 ppm; IR (thin film): 3052, 2968, 1679, 1590, 1519, 1486, 1344, 1110, 863, 749 cm^{-1}, HRMS calc'd for $C_{20}H_{22}N_3O_4^+$: 368.1605, found: 368.1600 $[M+H]^+$.

2-(1-benzyl-1*H*-indol-3-yl)-*N*-methyl-*N*-(4-nitrophenoxy)acetamide (2m)

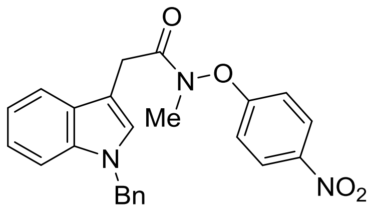

The reaction was performed following **GP1**, compound **2m** was obtained as yellow oil (36% yield, 42% brsm); R*f* = 0.46 (hexanes:ethyl acetate = 5:1); ^1H NMR (400 MHz, Chloroform-*d*) δ 7.98 (d, *J* = 9.2 Hz, 2H), 7.54 (d, *J* = 7.6 Hz, 1H), 7.20 – 7.13 (m, 4H), 7.10 – 7.02 (m, 2H), 7.00 – 6.98 (m, 2H), 6.89 – 6.86 (m, 3H), 5.03 (s, 2H), 3.83 (s, 2H), 3.23 (s, 3H) ppm. ^{13}C NMR (100 MHz, Chloroform-*d*) δ 162.5, 143.4, 137.5, 136.5, 128.8, 128.0, 127.8, 127.4, 127.1, 126.0, 122.2, 119.6, 119.2, 113.4, 109.9, 106.9, 49.9, 30.3, 27.0 ppm (one resonance was not observed due to overlapping peaks); IR (thin film): 3079, 2922, 1683, 1590, 1519, 1486, 1344, 1217, 863, 747 cm^{-1}, HRMS calc'd for $C_{24}H_{22}N_3O_4^+$: 416.1605, found:416.1601 $[M+H]^+$.

2-(1-allyl-1*H*-indol-3-yl)-*N*-methyl-*N*-(4-nitrophenoxy)acetamide (2n)

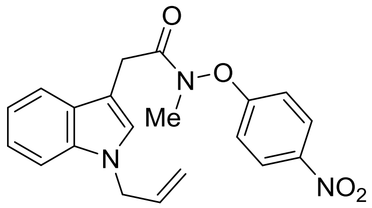

The reaction was performed following **GP1**, compound **2n** was obtained as yellow solid (33% yield, 46% brsm). m.p. = 142 – 144 °C; R*f* = 0.46 (hexanes:ethyl acetate = 5:1); ^1H NMR (400 MHz, Chloroform-*d*) δ 8.13 – 8.09 (m, 2H), 7.51 (d, *J* = 8.0 Hz, 1H), 7.22 – 7.15 (m, 2H), 7.08 (ddd, *J* = 8.0, 6.4, 1.2 Hz, 1H), 6.96 – 6.92 (m, 2H), 6.85 (s, 1H), 5.90 – 5.81 (m, 1H), 5.15 (dd, *J* = 10.0, 1.2 Hz, 1H), 5.04 (dd, *J* = 17.2,

1.6 Hz, 1H), 4.55 (d, J = 5.6 Hz, 2H), 3.87 (d, J = 0.8 Hz, 2H), 3.31 (s, 3H) ppm. ^{13}C NMR (100 MHz, Chloroform-d) δ 162.5, 143.4, 136.1, 133.2, 127.8, 126.8, 125.9, 121.9, 119.5, 118.9, 117.5, 113.2, 109.6, 106.5, 48.7, 35.2, 30.4 ppm (one resonance was not observed due to overlapping peaks); IR (thin film): 3080, 2920, 1684, 1590, 1518, 1486, 1344, 1218, 863, 747 cm^{-1}, HRMS calc'd for $C_{20}H_{20}N_3O_4^+$: 366.1448, found:366.1446 [M+H]$^+$.

***tert*-butyl (2-(1-methyl-1*H*-indol-3-yl)acetyl)(4-nitrophenoxy)carbamate (2o)**

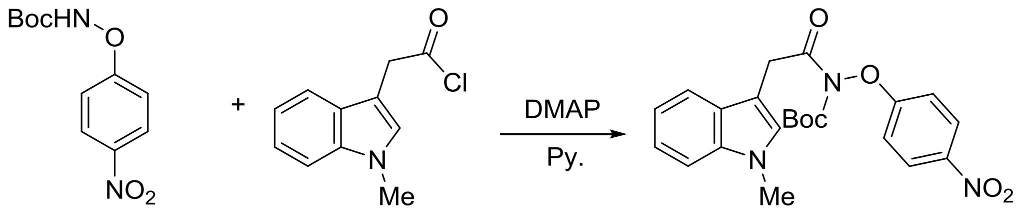

S4 **S5** **2o**

In a dry Schlenk tube equipped with a stirring bar the Boc-*O*-4-nitrophenyl-hydroxylamine **S4**[4] (1.0 g, 3.9 mmol) and DMAP (58 mg, 0.47 mmol) in anhydrous pyridine (18 mL) was cooled to 0 ºC. The mixture was stirred for 5 minutes and then indole-3-acetyl chloride **S5** (1.63 g, 7.9 mmol) in CH_2Cl_2 6 mL was added dropwise. The reaction mixture was allowed to warm to room temperature and stirred for 12 hours. The reaction was quenched with water and extracted with CH_2Cl_2. The combined organic extracts were dried and concentrated. The residue was purified on silica gel to afford the product **2o** as yellow solid (534 mg, 32%). m.p. = 136 – 138 °C; R_f = 0.67 (hexanes:ethyl acetate = 5:1); ^{1}H NMR (400 MHz, Chloroform-d) δ 8.02 (d, J = 9.2 Hz, 2H), 7.58 (d, J = 8.0 Hz, 1H), 7.27 (d, J = 8.0 Hz, 1H), 7.23 – 7.19 (m, 1H), 7.09 (td, J = 8.0, 1.2 Hz, 1H), 7.04 (s, 1H), 6.84 (d, J = 9.2 Hz, 2H), 4.41 – 4.31 (m, 2H), 3.69 (s, 3H), 1.43 (s, 9H) ppm. ^{13}C NMR (100 MHz, Chloroform-d) δ 169.0, 163.3, 149.8, 143.5, 136.8, 128.6, 127.7, 125.7, 122.0, 119.5, 119.1, 113.1, 109.4, 105.5, 86.0, 33.8, 32.7, 27.8 ppm; IR (thin film): 3080, 2919, 1633, 1590, 1344, 1135, 847, 742 cm^{-1}, HRMS calc'd for $C_{22}H_{24}N_3O_6^+$: 426.1660, found: 426.1657 [M+H]$^+$.

Procedure and characterization for the radical cyclization/coupling of *N*-benzylimine and *N*-methyl-2- (indole substitution)-*N*-(4-nitrophenoxy) acetamide – GP2

An oven-dried 20 mL reaction vial equipped with a stir bar was charged with ketimine **1a** (0.4 mmol) and amide **2a** (0.8 mmol) under a nitrogen atmosphere in a glove box. A solution of $NaN(SiMe_3)_2$ (0.8 mmol) in 8.0 mL dry DMSO was added by a "Eppendorf" brand 1000 μL pipettor to the reaction vial at

room temperature with stirring. The reaction mixture turned to a dark purple color on addition of the solution. The vial was sealed with a cap, removed from the glove box, and stirred for 3 h at room temperature. The reaction mixture was opened to air and quenched with three drops of H_2O. The aqueous layer was extracted with ethyl acetate (3 X 15 mL) and the combined organic layers were washed with saturated brine solution, dried (Na_2SO_4), filtered and evaporated under reduced pressure. The crude material was loaded onto a deactivated silica gel column and purified with ethyl acetate:hexanes = 1:8. Further purification was performed on an Agilent HPLC 1260 system using acetonitrile:H_2O (80:20 vol./vol.) as mobile phase and flow rate of 3.0 mL/min at 254 nm to give product **3aa**.

Product Characterization:

3a-(((diphenylmethylene)amino)(phenyl)methyl)-1,8-dimethyl-3,3a,8,8a-tetrahydropyrrolo[2,3-*b*]indol-2(1*H*)-one (3aa)

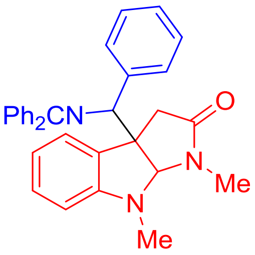

The reaction was performed following the **GP2** with *N*-benzyl-1,1-diphenylmethanimine **1a** (108.5 mg, 0.4 mmol) and *N*-methyl-2-(1-methyl-1*H*-indol-3-yl)-*N*-(4-nitrophenoxy)acetamide **2a** (271.3 mg, 0.8 mmol). The crude product was separated by flash chromatography on deactivated silica gel (ethyl acetate:hexanes = 1:8). Further purification was performed on an Agilent HPLC 1260 system using acetonitrile:H_2O (80:20 vol./vol.) as mobile phase and flow rate of 3.0 mL/min at 254 nm to give the product **3aa** in 86% overall yield (dr = 1.2:1, **3aa**(major), 88.5 mg, 47% yield; **3aa**(minor), 73.7 mg, 39% yield).

3aa(major): white solid , m.p. = 176 – 178 ℃ , R_f = 0.56 (hexanes:ethyl acetate = 1:1); ^{1}H NMR (600 MHz, Chloroform-*d*) δ 7.68 (d, *J* = 7.8 Hz, 2H), 7.43 (t, *J* = 7.2 Hz, 1H), 7.40 – 7.36 (m, 3H), 7.33 (t, *J* = 7.2 Hz, 2H), 7.12 – 7.05 (m, 4H), 6.92 – 6.88 (m, 3H), 6.81 (d, *J* = 7.2 Hz, 2H), 6.67 (t, *J* = 7.2 Hz, 1H), 6.22 (d, *J* = 7.8 Hz, 1H), 5.31 (s, 1H), 4.51 (s, 1H), 2.97 (d, *J* = 16.8 Hz, 1H), 2.88 (s, 3H), 2.68 (d, *J* = 16.8 Hz, 1H), 2.55 (s, 3H) ppm; ^{13}C{^{1}H} NMR (150 MHz, Chloroform-*d*) δ 172.7, 169.4, 150.8, 140.7, 139.4, 136.4, 133.1, 130.6, 129.1, 128.8, 128.7, 128.4, 128.3, 127.9, 127.6, 127.5, 127.2, 124.3,

118.3, 108.2, 86.6, 70.0, 56.2, 40.0, 35.4, 27.6 ppm. IR (thin film): 3057, 2926, 1692, 1492, 1447, 1208, 750, 703 cm^{-1}; HRMS calc'd for $C_{32}H_{30}N_3O^+$: 472.2383, found: 472.2387 $[M+H]^+$.

3aa(minor): colorless oil, R_f = 0.43 (hexanes:ethyl acetate = 1:1); ^1H NMR (600 MHz, Chloroform-*d*) δ 7.63 (d, *J* = 7.2 Hz, 2H), 7.41 – 7.39 (m, 2H), 7.37 – 7.33 (m, 4H), 7.22 – 7.16 (m, 3H), 7.12 (t, *J* = 8.4 Hz, 1H), 6.94 (d, *J* = 6.6 Hz, 2H), 6.84 – 6.81 (m, 3H), 6.69 (t, *J* = 7.8 Hz, 1H), 6.36 (d, *J* = 7.8 Hz, 1H), 4.83 (s, 1H), 4.40 (s, 1H), 3.16 (d, *J* = 16.8 Hz, 1H), 2.81 (s, 3H), 2.75 (s, 3H), 2.67 (d, *J* = 16.8 Hz, 1H) ppm; $^{13}C\{^1H\}$ NMR (150 MHz, Chloroform-*d*) δ 172.9, 168.6, 150.8, 140.3, 139.7, 136.7, 132.2, 130.4, 129.1, 128.8, 128.7, 128.4, 128.2, 128.0, 127.8, 127.7, 126.2, 118.4, 108.1, 88.0, 71.6, 56.3, 40.1, 35.8, 27.8 ppm (one resonance was not observed due to overlapping peaks).

3a-(((diphenylmethylene)amino)(4-methoxyphenyl)methyl)-1,8-dimethyl-3,3a,8,8a-tetrahydropyrrolo[2,3-*b*]indol-2(1*H*)-one (3ba)

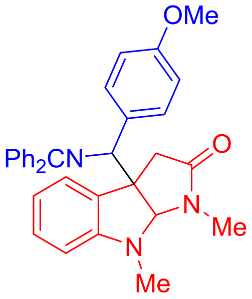

The reaction was performed following the **GP2** with *N*-(4-methoxybenzyl)-1,1-diphenylmethanimine **1b** (120.5 mg, 0.4 mmol) and *N*-methyl-2-(1-methyl-1*H*-indol-3-yl)-*N*-(4-nitrophenoxy)acetamide **2a** (271.3 mg, 0.8mmol). The crude product was separated by flash chromatography on deactivated silica gel (ethyl acetate:hexanes = 1:8). Further purification was performed on an Agilent HPLC 1260 system using acetonitrile:H_2O (80:20 vol./vol.) as mobile phase and flow rate of 3.0 mL/min at 254 nm to give the product **3ba** in 56% overall yield (dr = 1.2:1, **3ba**(major), 61.3 mg, 31% yield; **3ba**(minor), 51.1 mg, 25% yield).

3ba(major): white solid , m.p. = 194 – 196 °C, R_f = 0.44 (hexanes:ethyl acetate = 1:1); ^1H NMR (600 MHz, Methanol-d_4) δ 7.53 (d, *J* = 7.8 Hz, 2H), 7.34 – 7.23 (m, 6H), 6.96 (t, *J* = 7.2 Hz, 1H), 6.81 (d, *J* = 7.2 Hz, 1H), 6.71 (d, *J* = 7.8 Hz, 2H), 6.68 (d, *J* = 7.2 Hz, 2H), 6.58 – 6.55 (m, 3H), 6.22 (d, *J* = 7.8 Hz, 1H), 5.29 (s, 1H), 4.40 (s, 1H), 3.59 (s, 3H), 2.84 (d, *J* = 17.4 Hz, 1H), 2.78 (s, 3H), 2.54 (s, 3H), 2.51 (d, *J* = 17.4 Hz, 1H) ppm; $^{13}C\{^1H\}$ NMR (150 MHz, Methanol-d_4) δ 173.8, 169.6, 158.9, 150.9, 139.4,

136.4, 132.8, 132.5, 130.2, 128.9, 128.6, 128.4, 128.2, 128.1, 127.9, 127.1, 123.8, 118.2, 112.7, 108.3, 87.1, 69.2, 56.1, 54.2, 39.8, 34.5, 26.5 ppm; IR (thin film): 3053, 2927, 1686, 1511, 1220, ,985, 772 cm^{-1}; HRMS calc'd for $C_{33}H_{32}N_3O_2^+$: 502.2489, found: 502.2489 $[M+H]^+$.

3ba(minor): colorless oil, R_f = 0.34 (hexanes:ethyl acetate = 1:1); ^{1}H NMR (600 MHz, Methanol-d_4) δ 7.55 (d, J = 7.2 Hz, 2H), 7.43 – 7.36 (m, 4H), 7.31 (t, J = 7.8 Hz, 2H), 7.09 (t, J = 7.8 Hz, 1H), 6.88 (d, J = 8.4 Hz, 2H), 6.84 (d, J = 7.2 Hz, 1H), 6.81 (d, J = 6.6 Hz, 2H), 6.74 (d, J = 8.4 Hz, 2H), 6.67 (t, J = 7.2 Hz, 1H), 6.41 (d, J = 7.8 Hz, 1H), 5.07 (s, 1H), 4.43 (s, 1H), 3.73 (s, 3H) , 3.06 (d, J = 16.8 Hz, 1H), 2.81 (s, 3H), 2.79 (s, 3H), 2.56 (d, J = 16.8 Hz, 1H) ppm; $^{13}C\{^1H\}$ NMR (150 MHz, Methanol-d_4) δ 175.2, 170.1, 160.6, 152.2, 141.0, 138.0, 133.6, 133.4, 131.3, 130.7, 130.2, 129.8, 129.5, 129.4, 129.1, 128.8, 126.6, 119.4, 114.4, 109.4, 89.4, 72.0, 57.5, 55.7, 41.3, 36.2, 28.0 ppm;

3a-(benzo[*d*][1,3]dioxol-5-yl((diphenylmethylene)amino)methyl)-1,8-dimethyl-3,3a,8,8a-tetrahydropyrrolo[2,3-*b*]indol-2(1*H*)-one (3ca)

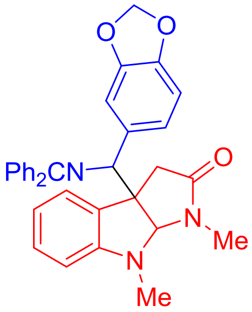

The reaction was performed following the **GP2** with *N*-(benzo[*d*][1,3]dioxol-5-ylmethyl)-1,1-diphenylmethanimine **1c** (126.1 mg, 0.4 mmol) and *N*-methyl-2-(1-methyl-1*H*-indol-3-yl)-*N*-(4-nitrophenoxy)acetamide **2a** (271.3 mg, 0.8 mmol). The crude product was separated by flash chromatography on deactivated silica gel (ethyl acetate:hexanes = 1:10). Further purification was purified on an Agilent HPLC 1260 system using acetonitrile:H_2O (80:20vol./vol.) as mobile phase and flow rate of 3.0 mL/min at 254 nm to give the product **3ca** in 60% overall yield (dr = 1:1, **3ca'**, 61.9 mg, 30% yield; **3ca"**, 61.9 mg, 30% yield).

3ca': colorless oil, R_f = 0.46 (hexanes:ethyl acetate = 1:1); ^{1}H NMR (600 MHz, Chloroform-d) δ 7.58 (d, J = 7.8 Hz, 2H), 7.36 (t, J = 7.2 Hz, 1H), 7.32 – 7.25 (m, 5H), 7.00 (t, J = 7.8 Hz, 1H), 6.81 (d, J = 7.2 Hz, 1H), 6.74 (d, J = 7.2 Hz, 2H), 6.59 (t, J = 7.2 Hz, 1H), 6.46 (d, J = 7.8 Hz, 1H), 6.42 (s, 1H), 6.28 (d, J = 8.4 Hz, 1H), 6.20 (d, J = 7.8 Hz, 1H), 5.79 (d, J = 9.0 Hz, 2H), 5.20 (s, 1H), 4.34 (s, 1H), 2.85 (d,

J = 16.8 Hz, 1H), 2.80 (s, 3H), 2.60 – 2.58 (m, 4H) ppm; $^{13}C\{^1H\}$ NMR (150 MHz, Chloroform-*d*) δ 171.6, 168.2, 149.6, 146.0, 145.4, 138.2, 135.2, 133.4, 132.0, 129.5, 128.1, 127.7, 127.6, 127.3, 127.2, 126.3, 123.2, 120.1, 117.3, 107.3, 107.1, 106.4, 99.8, 85.5, 68.6, 55.0, 38.7, 34.4, 26.6 ppm.

3ca": yellow oil, R_f = 0.37 (hexanes:ethyl acetate = 1:1); ^1H NMR (600 MHz, Methanol-d_4) δ 7.56 (d, J = 7.2 Hz, 2H), 7.43 – 7.35 (m, 4H), 7.30 (t, J = 7.8 Hz, 2H), 7.09 (t, J = 7.8 Hz, 1H), 6.90 (d, J = 7.2 Hz, 1H), 6.83 (d, J = 6.6 Hz, 2H), 6.69 (t, J = 7.2 Hz, 1H), 6.63 (d, J = 7.8 Hz, 1H), 6.49 (s, 1H), 6.40 (t, J = 7.8 Hz, 2H), 5.84 (d, J = 5.4 Hz, 2H), 5.06 (s, 1H), 4.41 (s, 1H), 3.01 (d, J = 17.4 Hz, 1H), 2.82 (s, 3H), 2.78 (s, 3H), 2.57 (d, J = 17.4 Hz, 1H) ppm; $^{13}C\{^1H\}$ NMR (150 MHz, Methanol-d_4) δ 175.0, 170.2, 152.1, 148.8, 148.4, 140.9, 137.8, 135.3, 133.1, 131.4, 130.3, 129.8, 129.6, 129.4, 129.1, 128.8, 126.6, 123.0, 119.4, 109.8, 109.4, 108.5, 102.3, 89.2, 72.2, 57.5, 41.3, 36.1, 28.0 ppm; IR (thin film): 3055, 2924, 1693, 1487, 1445, 1235, 1039, 769 cm^{-1}; HRMS calc'd for $C_{33}H_{30}N_3O_3^+$: 516.2282, found: 516.2281 $[M+H]^+$.

3a-(((diphenylmethylene)amino)(4-fluorophenyl)methyl)-1,8-dimethyl-3,3a,8,8a-tetrahydropyrrolo[2,3-*b*]indol-2(1*H*)-one (3da)

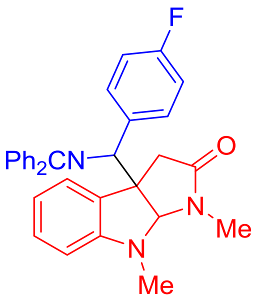

The reaction was performed following the **GP2** with *N*-(4-fluorobenzyl)-1,1-diphenylmethanimine **1d** (115.7 mg, 0.4 mmol) and *N*-methyl-2-(1-methyl-1*H*-indol-3-yl)-*N*-(4-nitrophenoxy)acetamide **2a** (271.3 mg, 0.8 mmol). The crude product was separated by flash chromatography on deactivated silica gel (ethyl acetate:hexanes = 1:8). Further purification was performed on an Agilent HPLC 1260 system using acetonitrile:H_2O (80:20 vol./vol.) as mobile phase and flow rate of 3.0 mL/min at 254 nm to give the product **3da** in 80% overall yield (dr = 1.4:1, **3da**(major), 91.4 mg, 47% yield; **3da**(minor), 65.3 mg, 33% yield).

3da(major): white solid, m.p. = 222 – 224 ℃, R_f = 0.50 (hexanes:ethyl acetate = 1:1); ^1H NMR (400 MHz, Chloroform-*d*) δ 7.65 (d, J = 6.8 Hz, 2H), 7.43 (q, J = 7.2 Hz, 1H) , 7.40 – 7.32 (m, 5H), 7.06 (t, J

= 7.6 Hz, 1H), 6.88 (d, J = 7.2 Hz, 1H), 6.84 – 6.81 (m, 4H), 6.76 (t, J = 8.4 Hz, 2H), 6.66 (t, J = 7.2 Hz, 1H), 6.21 (d, J = 7.6 Hz, 1H), 5.30 (s, 1H), 4.47 (s, 1H), 2.91 – 2.86 (m, 4H), 2.64 (d, J = 16.8 Hz, 1H), 2.57 (s, 3H) ppm; ^{13}C{^{1}H} NMR (100 MHz, Chloroform-d) δ 172.6, 169.8, 161.9 (d, $^{1}J_{C-F}$ = 244.3 Hz), 150.8, 139.2, 136.5 (d, $^{4}J_{C-F}$ = 3.0 Hz), 136.3, 132.7, 130.8, 129.3 (d, $^{3}J_{C-F}$ = 5.4 Hz), 129.2, 128.7, 128.5, 128.4, 127.4, 124.2, 118.4, 114.4 (d, $^{2}J_{C-F}$ = 20.9 Hz), 108.2, 86.4, 69.2, 56.1, 39.9, 35.3, 27.7 ppm (one resonance was not observed due to overlapping peaks); ^{19}F NMR (377 MHz, Chloroform-d) δ -115.17 ppm; IR (thin film): 3054, 2920, 1690, 1506, 1443, 1220, 772, 748 cm^{-1}; HRMS calc'd for $C_{32}H_{29}FN_3O^+$: 490.2289, found: 490.2295 [M+H]$^+$.

3da(minor): yellow solid, m.p. = 204 – 206 ℃, R$_f$ = 0.38 (hexanes:ethyl acetate = 1:1); ^{1}H NMR (400 MHz, Chloroform-d) δ 7.55 (d, J = 7.2 Hz, 2H), 7.35 – 7.25 (m, 6H), 7.04 (t, J = 7.6 Hz, 1H), 6.80 – 6.75 (m, 7H), 6.62 (t, J = 7.6 Hz, 1H), 6.27 (d, J = 8.0 Hz, 1H), 4.66 (s, 1H), 4.30 (s, 1H), 3.05 (d, J = 16.8 Hz, 1H), 2.72 (s, 3H), 2.66 (s, 3H), 2.59 (d, J = 16.8 Hz, 1H) ppm; ^{13}C{^{1}H} NMR (100 MHz, Chloroform-d) δ 172.7, 168.9, 162.2 (d, $^{1}J_{C-F}$ = 244.7 Hz), 150.6, 139.5, 136.6, 136.1 (d, $^{4}J_{C-F}$ = 3.2 Hz), 131.7, 130.6, 130.1 (d, $^{3}J_{C-F}$ = 7.6 Hz), 129.2, 128.81, 128.75, 128.5, 128.3, 127.6, 126.2, 118.4, 114.8 (d, $^{2}J_{C-F}$ = 21.0 Hz), 108.1, 87.9, 70.7, 56.2, 39.9, 35.6, 27.8 ppm; ^{19}F NMR (377 MHz, Chloroform-d) δ -114.49 ppm.

3a-((4-chlorophenyl)((diphenylmethylene)amino)methyl)-1,8-dimethyl-3,3a,8,8a-tetrahydropyrrolo[2,3-*b*]indol-2(1*H*)-one (3ea)

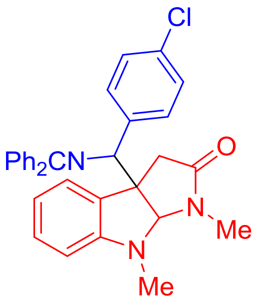

The reaction was performed following the **GP2** with *N*-(4-chlorobenzyl)-1,1-diphenylmethanimine **1e** (122.0 mg, 0.4 mmol) and *N*-methyl-2-(1-methyl-1*H*-indol-3-yl)-*N*-(4-nitrophenoxy)acetamide **2a** (271.3 mg, 0.8mmol). The crude product was separated by flash chromatography on deactivated silica gel (ethyl acetate:hexanes = 1:8). Further purification was performed on an Agilent HPLC 1260 system using acetonitrile:H_2O (80:20 vol./vol.) as mobile phase and flow rate of 3.0 mL/min at 254 nm to give

the product **3ea** in 76% overall yield (dr = 1.1:1, **3ea**(major), 80.6 mg, 40% yield; **3ea**(minor), 73.3 mg, 36% yield).

3ea(major): white solid, m.p. = 194 – 195 °C, R_f = 0.53 (hexanes:ethyl acetate = 1:1); ^{1}H NMR (600 MHz, Chloroform-*d*) δ 7.64 (d, *J* = 7.8 Hz, 2H), 7.44 (t, *J* = 7.2 Hz, 1H), 7.41 – 7.33 (m, 5H), 7.08 – 7.03 (m, 3H), 6.88 (d, *J* = 7.2 Hz, 1H), 6.81 (d, *J* = 7.2 Hz, 2H), 6.78 (d, *J* = 8.4 Hz, 2H), 6.66 (t, *J* = 7.2 Hz, 1H), 6.22 (d, *J* = 7.8 Hz, 1H), 5.27 (s, 1H), 4.46 (s, 1H), 2.89 –2.87 (m, 4H) , 2.62 (d, *J* = 16.8 Hz, 1H), 2.57 (s, 3H) ppm; ^{13}C{^{1}H} NMR (150 MHz, Chloroform-*d*) δ 172.5, 170.0, 150.8, 139.4, 139.3, 136.4, 132.9, 132.7, 130.8, 129.4, 129.1, 128.88, 128.85, 128.6, 128.4, 127.7, 127.4, 124.2, 118.4, 108.3, 86.4, 69.4, 56.1, 40.0, 35.3, 27.7 ppm; IR (thin film): 3058, 2921, 1693, 1490, 1445, 1220, 913, 773, 748 cm^{-1}; HRMS calc'd for $C_{32}H_{29}ClN_3O^+$: 506.1994, found: 506.1995 [M+H]$^+$.

3ea(minor): colorless oil, R_f = 0.37 (hexanes:ethyl acetate = 1:1); ^{1}H NMR (600 MHz, Chloroform-*d*) δ 7.53 (d, *J* = 7.8 Hz, 2H), 7.34 (t, *J* = 7.2 Hz, 2H), 7.30 – 7.26 (m, 4H), 7.07 (d, *J* = 8.4 Hz, 2H), 7.04 (t, *J* = 7.8 Hz, 1H), 6.80 (d, *J* = 8.4 Hz, 2H), 6.76 – 6.75 (m, 3H), 6.61 (t, *J* = 7.2 Hz, 1H), 6.28 (d, *J* = 7.8 Hz, 1H), 4.71 (s, 1H), 4.30 (s, 1H), 3.04 (d, *J* = 16.8 Hz, 1H), 2.72 (s, 3H), 2.69 (s, 3H), 2.59 (d, *J* = 16.8 Hz, 1H) ppm; ^{13}C{^{1}H} NMR (150 MHz, Chloroform-*d*) δ 170.5, 167.5, 148.5, 137.1, 136.5, 134.3, 131.4, 129.4, 128.7, 127.8, 127.2, 126.9, 126.8, 126.4, 126.2, 126.0, 125.6, 124.0, 116.3, 105.9, 85.6, 68.7, 54.0, 37.7, 33.4, 25.7 ppm.

3a-((4-bromophenyl)((diphenylmethylene)amino)methyl)-1,8-dimethyl-3,3a,8,8a-tetrahydropyrrolo[2,3-*b*]indol-2(1*H*)-one (3fa)

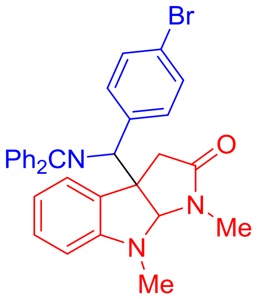

The reaction was performed following the **GP2** with *N*-(4-bromobenzyl)-1,1-diphenylmethanimine **1f** (139.6 mg, 0.4 mmol) and *N*-methyl-2-(1-methyl-1*H*-indol-3-yl)-*N*-(4-nitrophenoxy)acetamide **2a** (271.3 mg, 0.8mmol). The crude product was separated by flash chromatography on deactivated silica gel (ethyl acetate:hexanes = 1:10). Further purification was performed on an Agilent HPLC 1260 system

using acetonitrile:H_2O (80:20 vol./vol.) as mobile phase and flow rate of 3.0 mL/min at 254 nm to give the product **3fa** in 66% overall yield (dr = 1:1, **3fa'**, 72.7 mg, 33% yield; **3fa''**, 72.7 mg, 33% yield).

3fa': yellow solid, m.p. = 215 – 216 °C, R_f = 0.54 (hexanes:ethyl acetate = 1:1); ^{1}H NMR (400 MHz, Chloroform-*d*) δ 7.64 (d, *J* = 7.6 Hz, 2H), 7.43 (dd, *J* = 14.4, 7.6 Hz, 2H), 7.39 – 7.33 (m, 4H), 7.19 (d, *J* = 8.0 Hz, 2H), 7.07 (t, *J* = 7.6 Hz, 1H), 6.87 (d, *J* = 7.2 Hz, 1H), 6.81 (d, *J* = 7.2 Hz, 2H), 6.72 (d, *J* = 8.0 Hz, 2H), 6.66 (t, *J* = 7.2 Hz, 1H), 6.22 (d, *J* = 7.6 Hz, 1H), 5.27 (s, 1H), 4.44 (s, 1H), 2.90 – 2.86 (m, 4H), 2.64 (d, *J* = 16.8 Hz, 1H), 2.57 (s, 3H) ppm; ^{13}C{^{1}H} NMR (100 MHz, Chloroform-*d*) δ 172.4, 169.9, 150.6, 139.7, 139.1, 136.2, 132.5, 130.7, 130.5, 129.3, 128.8, 128.7, 128.5, 128.3, 127.3, 124.1, 121.0, 118.3, 108.2, 86.2, 69.2, 56.0, 39.8, 35.2, 27.6 ppm (one resonance was not observed due to overlapping peaks).

3fa'': yellow oil, R_f = 0.38 (hexanes:ethyl acetate = 1:1); ^{1}H NMR (400 MHz, Chloroform-*d*) δ 7.62 (d, *J* = 7.6 Hz, 2H), 7.44 –7.34 (m, 6H), 7.30 (d, *J* = 8.0 Hz, 2H), 7.13 (t, *J* = 7.6 Hz, 1H), 6.84 – 6.82 (m, 3H), 6.80 (d, *J* = 8.0 Hz, 2H), 6.70 (t, *J* = 7.2 Hz, 1H), 6.36 (d, *J* = 7.6 Hz, 1H), 4.74 (s, 1H), 4.35 (s, 1H), 3.14 (d, *J* = 16.8 Hz, 1H), 2.81 (s, 3H), 2.75 (s, 3H), 2.67 (d, *J* = 16.8 Hz, 1H) ppm; ^{13}C{^{1}H} NMR (100 MHz, Chloroform-*d*) δ 172.7, 169.2, 150.6, 139.4, 139.3, 136.5, 131.6, 131.1, 130.7, 130.3, 129.3, 128.9, 128.8, 128.6, 128.3, 127.6, 126.2, 121.6, 118.4, 108.1, 87.8, 70.8, 56.1, 39.8, 35.6, 27.8 ppm; IR (thin film): 3057, 2897, 1691, 1489, 1445, 1398, 1220, 773, 747 cm^{-1}; HRMS calc'd for $C_{32}H_{29}BrN_3O^+$: 550.1489, found: 550.1492 [M+H]$^+$.

3a-((3,5-difluorophenyl)((diphenylmethylene)amino)methyl)-1,8-dimethyl-3,3a,8,8a-tetrahydropyrrolo[2,3-*b*]indol-2(1*H*)-one (3ga)

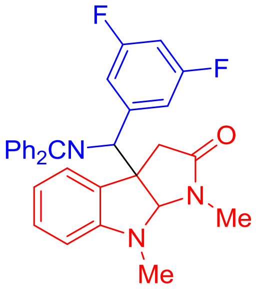

The reaction was performed following the **GP2** with *N*-(3,5-difluorobenzyl)-1,1-diphenylmethanimine **1g** (122.8 mg, 0.4 mmol) and *N*-methyl-2-(1-methyl-1*H*-indol-3-yl)-*N*-(4-nitrophenoxy)acetamide **2a** (271.3 mg, 0.8mmol). The crude product was separated by flash chromatography on deactivated silica

gel (ethyl acetate:hexanes = 1:10). Further purification was purified on an Agilent HPLC 1260 system using acetonitrile:H_2O (80:20 vol./vol.) as mobile phase and flow rate of 3.0 mL/min at 254 nm to give the product **3ga** in 63% overall yield (dr = 1.3:1, **3ga**(major), 72.3 mg, 36% yield; **3ga**(minor), 55.6 mg, 27% yield).

3ga(major): colorless oil, R_f = 0.58 (hexanes:ethyl acetate = 1:1); ^{1}H NMR (600 MHz, Chloroform-*d*) δ 7.65 (d, *J* = 7.8 Hz, 2H), 7.47 – 7.42 (m, 2H), 7.39 – 7.37 (m, 4H), 7.10 (t, *J* = 7.8 Hz, 1H), 6.88 – 6.84 (m, 3H), 6.69 (t, *J* = 7.2 Hz, 1H), 6.56 (t, *J* = 9.0 Hz, 1H), 6.39 (d, *J* = 6.0 Hz, 2H), 6.25 (d, *J* = 7.8 Hz, 1H), 5.31 (s, 1H), 4.45 (s, 1H), 2.90 (s, 3H), 2.85 (d, *J* = 16.8 Hz, 1H), 2.66 – 2.63 (m, 4H) ppm; ^{13}C{^{1}H} NMR (150 MHz, Chloroform-*d*) δ 172.1, 170.6, 162.2 (dd, $^1J_{C\text{-}F}$ = 246.5, $^3J_{C\text{-}F}$ = 12.6 Hz), 150.5, 144.6 (t, $^3J_{C\text{-}F}$ = 8.9 Hz), 138.9, 136.0, 132.0, 130.9, 129.6, 128.9, 128.8, 128.6, 128.3, 127.2, 123.9, 118.5, 110.5 (dd, $^2J_{C\text{-}F}$ = 20.7, $^4J_{C\text{-}F}$ = 5.3 Hz), 108.1, 102.5 (t, $^3J_{C\text{-}F}$ = 25.2 Hz), 85.9, 69.1, 56.0, 39.7, 35.0, 27.6 ppm; ^{19}F NMR (565 MHz, Chloroform-*d*) δ -110.47 ppm; IR (thin film): 3055, 2893, 1693, ,1491, 1219, 1116, 773, 744 cm^{-1}; HRMS calc'd for $C_{32}H_{28}F_2N_3O^+$: 508.2195, found 508.2197 [M+H]$^+$.

3ga(minor): colorless oil, R_f = 0.42 (hexanes:ethyl acetate = 1:1); ^{1}H NMR (600 MHz, Methanol-*d*$_4$) δ 7.63 (d, *J* = 7.2 Hz, 2H), 7.45 – 7.38 (m, 4H), 7.33 (t, *J* = 7.8 Hz, 2H), 7.13 (td, *J* = 7.8, 1.2 Hz, 1H), 7.10 (d, *J* = 7.8 Hz, 1H), 6.86 (d, *J* = 5.4 Hz, 2H), 6.77 – 6.73 (m, 2H), 6.52 (dd, *J* = 8.4, 2.4 Hz, 2H), 6.39 (d, *J* = 7.8 Hz, 1H), 5.02 (s, 1H), 4.53 (s, 1H), 2.97 (d, *J* = 17.4 Hz, 1H), 2.78 (s, 3H), 2.75 (s, 3H), 2.68 (d, *J* = 17.4 Hz, 1H) ppm; ^{13}C{^{1}H} NMR (150 MHz, Methanol-*d*$_4$) δ 174.6, 171.5, 163.8 (dd, $^1J_{C\text{-}F}$ = 245.9, $^3J_{C\text{-}F}$ = 12.6 Hz), 151.9, 145.7 (t, $^3J_{C\text{-}F}$ = 8.6 Hz), 140.5, 137.6, 132.1, 131.7, 130.7, 130.0, 129.7, 129.2, 128.6, 126.9, 119.4, 112.4 (dd, $^2J_{C\text{-}F}$ = 20.9, $^4J_{C\text{-}F}$ = 5.0 Hz), 109.2, 103.7 (t, $^3J_{C\text{-}F}$ = 25.4 Hz), 88.8, 71.9, 57.3, 41.3, 35.5, 27.9 ppm (one resonance was not observed due to overlapping peaks); ^{19}F NMR (565 MHz, Methanol-*d*$_4$) δ -111.68 ppm.

3a-(((diphenylmethylene)amino)(4-(trifluoromethyl)phenyl)methyl)-1,8-dimethyl-3,3a,8,8a-tetrahydropyrrolo[2,3-*b*]indol-2(1*H*)-one (3ha)

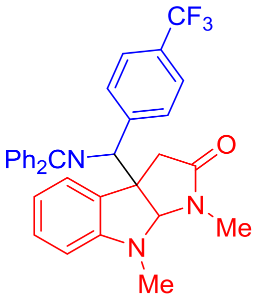

The reaction was performed following the **GP2** with 1,1-diphenyl-*N*-(4-(trifluoromethyl)benzyl)methanimine **1h** (135.6 mg, 0.4 mmol) and *N*-methyl-2-(1-methyl-1*H*-indol-3-yl)-*N*-(4-nitrophenoxy)acetamide **2a** (271.3 mg, 0.8mmol). The crude product was separated by flash chromatography on deactivated silica gel (ethyl acetate:hexanes = 1:8). Further purification was purified on an Agilent HPLC 1260 system using acetonitrile:H_2O (80:20 vol./vol.) as mobile phase and flow rate of 3.0 mL/min at 254 nm to give the product **3ha** in 73% overall yield (dr = 1.2:1, **3ha**(major), 85.9 mg, 40% yield; **3ha**(minor), 71.6 mg, 33% yield).

3ha(major): yellow oil, R_f = 0.56 (hexanes:ethyl acetate = 1:1); ^{1}H NMR (600 MHz, Chloroform-*d*) δ 7.66 (d, *J* = 7.8 Hz, 2H), 7.45 (t, *J* = 7.2 Hz, 1H), 7.41 –7.34 (m, 5H), 7.32 (d, *J* = 8.4 Hz, 2H), 7.07 (t, *J* = 7.8 Hz, 1H), 6.97 (d, *J* = 7.8 Hz, 2H), 6.90 (d, *J* = 7.2 Hz, 1H), 6.82 (d, *J* = 7.2 Hz, 2H), 6.68 (t, *J* = 7.2 Hz, 1H), 6.19 (d, *J* = 7.8 Hz, 1H), 5.30 (s, 1H), 4.55 (s, 1H), 2.92 – 2.89 (m, 4H), 2.67 (d, *J* = 16.8 Hz, 1H), 2.51 (s, 3H) ppm; ^{13}C{^{1}H} NMR (150 MHz, Chloroform-*d*) δ 172.4, 170.4, 150.6, 144.9, 139.1, 136.2, 132.3, 130.9, 129.6, 129.5 (q, $J_{C\text{-}F}$ = 31.7 Hz), 129.0, 128.9, 128.6, 128.4, 128.1, 127.3, 124.3 (q, $J_{C\text{-}F}$ = 3.2 Hz), 124.18 (q, $J_{C\text{-}F}$ = 270.1 Hz), 124.16, 118.5, 108.2, 86.2, 69.6, 56.2, 39.8, 35.0, 27.7 ppm; ^{19}F NMR (565 MHz, Chloroform-*d*) δ -62.51 ppm; IR (thin film): 3006, 2990, 1693, 1493, 1446, 1325, 1261, 750 cm^{-1}; HRMS calc'd for $C_{33}H_{29}F_3N_3O^+$: 540.2257, found: 540.2262 [M+H]$^+$.

3ha(minor): yellow oil, R_f = 0.36 (hexanes:ethyl acetate = 1:1); ^{1}H NMR (600 MHz, Chloroform-*d*) δ 7.63 (d, *J* = 8.4 Hz, 2H), 7.44 – 7.42 (m, 4H), 7.40 – 7.35 (m, 4H), 7.14 (td, *J* = 7.8, 1.2 Hz, 1H), 7.05 (d, *J* = 7.8 Hz, 2H), 6.86 – 6.83 (m ,3H), 6.71 (t, *J* = 7.2 Hz, 1H), 6.35 (d, *J* = 7.8 Hz, 1H), 4.78 (s, 1H), 4.47 (s, 1H), 3.12 (d, *J* = 16.8 Hz, 1H), 2.80 (s, 3H), 2.73 (s, 3H), 2.70 (d, *J* = 16.8 Hz, 1H) ppm; ^{13}C{^{1}H} NMR (150 MHz, Chloroform-*d*) δ 172.5, 169.7, 150.5, 144.4, 139.3, 136.5, 132.5, 131.3, 130.8, 130.0 (q, $J_{C\text{-}F}$ = 32.3 Hz), 129.2 (q, $J_{C\text{-}F}$ = 49.9 Hz), 129.0, 128.8, 128.6, 128.4, 127.6, 126.3, 124.8 (q, $J_{C\text{-}F}$ = 3.9 Hz), 124.2 (q, $J_{C\text{-}F}$ = 270.5 Hz), 118.5, 108.1, 87.9, 71.2, 56.2, 39.9, 35.4, 27.8 ppm; ^{19}F NMR (565 MHz, Chloroform-*d*) δ -62.51 ppm.

3a-(((diphenylmethylene)amino)(o-tolyl)methyl)-1,8-dimethyl-3,3a,8,8a-tetrahydropyrrolo[2,3-*b*]indol-2(1*H*)-one (3ia)

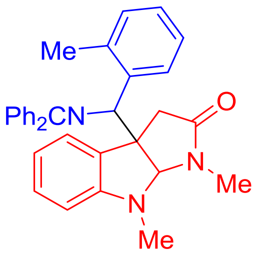

The reaction was performed following the **GP2** with *N*-(2-methylbenzyl)-1,1-diphenylmethanimine **1i** (114.1 mg, 0.4 mmol) and *N*-methyl-2-(1-methyl-1*H*-indol-3-yl)-*N*-(4-nitrophenoxy)acetamide **2a** (271.3mg, 0.8 mmol). The crude product was separated by flash chromatography on deactivated silica gel (ethyl acetate:hexanes = 1:8). Further purification was performed on an Agilent HPLC 1260 system using acetonitrile:H_2O (80:20 vol./vol.) as mobile phase and flow rate of 3.0 mL/min at 254 nm to give the product **3ia** in 56% overall yield (dr = 1:1, **3ia'**, 54.4 mg, 28% yield; **3ia"**, 54.4 mg, 28% yield).

3ia': colorless oil, R_f = 0.52 (hexanes:ethyl acetate = 1:1); ^{1}H NMR (600 MHz, Methanol-*d*$_4$) δ 7.70 (d, *J* = 7.8 Hz, 1H), 7.66 (dd, *J* = 7.8, 1.8 Hz, 2H), 7.41 (t, *J* = 7.2 Hz, 1H), 7.37 – 7.32 (m, 3H), 7.26 (t, *J* = 7.8 Hz, 2H), 7.17 (t, *J* = 7.8 Hz, 1H), 7.09 – 7.03 (m, 3H), 6.92 (d, *J* = 7.2 Hz, 1H), 6.62 (t, *J* = 7.8 Hz, 1H), 6.52 (d, *J* = 7.2 Hz, 2H), 6.36 (d, *J* = 7.8 Hz, 1H), 5.13 (s, 1H), 4.80 (s, 1H), 3.35 (d, *J* = 16.8 Hz, 1H), 2.89 (s, 3H), 2.86 (d, *J* = 16.8 Hz, 1H), 2.76 (s, 3H), 1.46 (s, 3H) ppm; ^{13}C{^{1}H} NMR (150 MHz, Methanol-*d*$_4$) δ 175.3, 170.6, 150.5, 140.6, 140.2, 138.5, 136.8, 134.1, 131.5, 131.4, 130.4, 130.1, 129.6, 129.5, 129.4, 129.2, 128.14, 128.06, 126.8, 125.8, 118.9, 108.4, 87.9, 65.7, 57.5, 39.8, 34.4, 28.3, 19.6 ppm.

3ia": colorless oil, R_f = 0.52 (hexanes:ethyl acetate = 1:1); ^{1}H NMR (600 MHz, Methanol-*d*$_4$) δ 7.53 – 7.49 (m, 3H), 7.30 – 7.24 (m, 4H), 7.22 (t, *J* = 7.8 Hz, 2H), 7.08 (t, *J* = 7.2 Hz, 1H), 7.00 (td, *J* = 7.2, 1.2 Hz, 1H), 6.96 (td, *J* = 7.8, 1.2 Hz, 1H), 6.76 (d, *J* = 7.8 Hz, 1H), 6.70 (s, 2H), 6.34 (t, *J* = 7.2 Hz, 2H), 5.94 (d, *J* = 7.2 Hz, 1H), 4.94 (s, 1H), 4.60 (s, 1H), 3.73 (d, *J* = 16.8 Hz, 1H), 2.97 (s, 3H), 2.75 (s, 3H), 2.41 (d, *J* = 16.8 Hz, 1H), 0.84 (s, 3H) ppm; ^{13}C{^{1}H} NMR (150 MHz, Methanol-*d*$_4$) δ 175.7, 171.0, 151.4, 140.7, 140.5, 138.9, 138.0, 133.0, 131.5, 131.0, 130.3, 129.8, 129.6, 129.5, 129.2, 129.0, 128.3, 128.2, 127.0, 126.0, 119.4, 108.9, 89.6, 64.2, 58.0, 38.9, 35.6, 28.1, 18.9 ppm; IR (thin film): 3056, 2925, 1694, 1492, 1461, 1445, 731, 699 cm^{-1}; HRMS calc'd for $C_{33}H_{32}N_3O^+$: 486.2540, found: 486.2539 $[M+H]^+$.

3a-(((diphenylmethylene)amino)(naphthalen-1-yl)methyl)-1,8-dimethyl-3,3a,8,8a-tetrahydropyrrolo[2,3-*b*]indol-2(1*H*)-one (3ja)

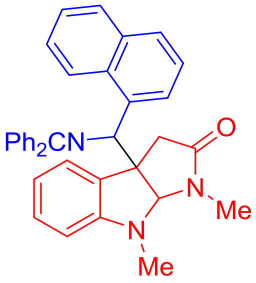

The reaction was performed following the **GP2** with *N*-(naphthalen-2-ylmethyl)-1,1-diphenylmethanimine **1j** (128.5 mg, 0.4 mmol) and *N*-methyl-2-(1-methyl-1*H*-indol-3-yl)-*N*-(4-nitrophenoxy)acetamide **2a** (271.3 mg, 0.8 mmol). The crude product was separated by flash chromatography on deactivated silica gel (ethyl acetate:hexanes = 1:10). Further purification was purified on an Agilent HPLC 1260 system using acetonitrile:H_2O (80:20 vol./vol.) as mobile phase and flow rate of 3.0 mL/min at 254 nm to give the product **3ja** in 68% overall yield (dr = 1.2:1, **3ja**(major), 77.4 mg, 37% yield; **3ja**(minor), 64.5 mg, 31% yield).

3ja(major): yellow oil, R_f = 0.51 (hexanes:ethyl acetate = 1:1); ^{1}H NMR (600 MHz, Methanol-*d*4) δ 7.72 – 7.69 (m, 3H), 7.60 (dd, *J* = 6.6, 3.6 Hz, 1H), 7.55 (d, *J* = 8.4 Hz, 1H), 7.46 (td, *J* = 7.2, 1.2 Hz, 1H), 7.42 – 7.37 (m, 6H), 7.34 (t, *J* = 7.8 Hz, 2H), 7.04 (td, *J* = 7.8, 1.2 Hz, 1H), 7.00 (d, *J* = 7.2 Hz, 1H), 6.96 (dd, *J* = 8.4, 1.8 Hz, 1H), 6.80 (d, *J* = 7.2 Hz, 2H), 6.72 (t, *J* = 7.2 Hz, 1H), 6.19 (d, *J* = 7.8 Hz, 1H), 5.55 (s, 1H), 4.72 (s, 1H), 3.02 (d, *J* = 17.4 Hz, 1H), 2.87 (s, 3H), 2.68 (d, *J* = 17.4 Hz, 1H), 2.44 (s, 3H) ppm; ^{13}C{^{1}H} NMR (150 MHz, Methanol-*d*4) δ 175.1, 171.6, 152.2, 140.7, 139.4, 137.8, 134.4, 134.2, 134.0, 131.7, 130.4, 129.9, 129.7, 129.5, 129.3, 128.9, 128.5, 128.4, 128.0, 127.9, 127.1, 127.0, 126.9, 125.3, 119.6, 109.6, 88.4, 71.3, 57.7, 41.2, 35.6, 27.9 ppm; IR (thin film): 3055, 2924, 1693, 1489, 1434, 984, 670 cm^{-1}, HRMS calc'd for $C_{36}H_{32}N_3O^+$: 522.2540, found: 522.2543 [M+H]$^+$.

3ja(minor): yellow oil, R_f = 0.40 (hexanes:ethyl acetate = 1:1); ^{1}H NMR (600 MHz, Methanol-*d*4) δ 7.78 – 7.76 (m, 1H), 7.66 (dd, *J* = 9.0 , 4.2 Hz, 2H), 7.62 (d, *J* = 7.2 Hz, 2H), 7.45 – 7.37 (m, 7H), 7.34 (t, *J* = 7.8 Hz, 2H), 7.14 – 7.09 (m, 2H), 6.91 (d, *J* = 7.2 Hz, 1H), 6.82 (d, *J* = 6.6 Hz, 2H), 6.69 (t, *J* = 7.2 Hz, 1H), 6.37 (d, *J* = 7.8 Hz, 1H), 5.16 (s, 1H), 4.67 (s, 1H), 3.15 (d, *J* = 17.4 Hz, 1H), 2.78 (s, 3H), 2.69 (s, 3H), 2.65 (d, *J* = 17.4 Hz, 1H) ppm; ^{13}C{^{1}H} NMR (150 MHz, Methanol-*d*4) δ 175.1, 170.8, 152.2, 141.0, 139.1, 138.0, 134.5, 134.4, 133.1, 131.5, 130.3, 129.9, 129.6, 129.5, 129.1, 128.9, 128.8, 128.7, 128.5, 128.4, 127.6, 127.03, 126.96, 126.8, 119.4, 109.4, 89.4, 72.8, 57.7, 41.4, 36.0, 27.9.ppm.

3a-(((diphenylmethylene)amino)(pyridin-3-yl)methyl)-1,8-dimethyl-3,3a,8,8a-tetrahydropyrrolo[2,3-*b*]indol-2(1*H*)-one (3ka)

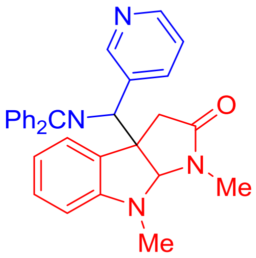

The reaction was performed following the **GP2** with 1,1-diphenyl-*N*-(pyridin-3-ylmethyl)methanimine **1k** (108.9 mg, 0.4 mmol) and *N*-methyl-2-(1-methyl-1*H*-indol-3-yl)-*N*-(4-nitrophenoxy)acetamide **2a** (271.3 mg, 0.8 mmol). The crude product was separated by flash chromatography on deactivated silica gel (ethyl acetate:hexanes = 1:8). Further purification was purified on an Agilent HPLC 1260 system using acetonitrile:H_2O (85:15 vol./vol.) as mobile phase and flow rate of 3.0 mL/min at 254 nm to give the product **3ka** in 62% overall yield (dr = 1.2:1, **3ka**(major), 63.9 mg, 34% yield; **3ka**(minor), 53.3 mg, 28% yield).

3ka(major): yellow oil, R_f = 0.53 (methanol : ethyl acetate = 1:15); ^{1}H NMR (400 MHz, Chloroform-*d*) δ 8.23 (d, *J* = 4.8 Hz, 1H), 8.00 (s, 1H), 7.55 (d, *J* = 8.0 Hz, 2H), 7.38 – 7.31 (m, 2H), 7.30 – 7.25 (m, 4H), 7.03 (d, *J* = 8.0 Hz, 1H), 6.96 (t, *J* = 7.6 Hz, 1H), 6.88 (dd, *J* = 8.0, 4.8 Hz, 1H), 6.80 (d, *J* = 7.6 Hz, 1H), 6.72 (d, *J* = 6.8 Hz, 2H), 6.57 (t, *J* = 7.2 Hz, 1H), 6.07 (d, *J* = 8.0 Hz, 1H), 5.28 (s, 1H), 4.42 (s, 1H), 2.80 – 2.76 (m, 4H), 2.57 (d, *J* = 16.8 Hz, 1H), 2.49 (s, 3H) ppm; $^{13}C\{^1H\}$ NMR (100 MHz, Chloroform-*d*) δ 171.2, 169.5, 149.3, 147.8, 147.5, 137.8, 135.3, 135.0, 134.1, 130.9, 129.8, 128.5, 127.9, 127.7, 127.6, 127.2, 126.1, 122.9, 121.4, 117.4, 106.9, 84.9, 66.8, 54.9, 38.5, 33.8, 26.6 ppm; IR (thin film): 3055, 2926, 1693, 1493, 1446, 1421, 1317, 772, 749 cm^{-1}; HRMS calc'd for $C_{31}H_{29}N_4O^+$: 473.2336, found: 473.2338 $[M+H]^+$.

3ka(minor): yellow oil, R_f = 0.40 (methanol : ethyl acetate = 1:15); ^{1}H NMR (400 MHz, Chloroform-*d*) δ 8.36 (dd, *J* = 4.8, 1.6 Hz, 1H), 7.99 (s, 1H), 7.58 (d, *J* = 7.2 Hz, 2H), 7.37 – 7.27 (m, 6H), 7.12 (d, *J* = 8.0 Hz, 1H), 7.07 (t, *J* = 8.4 Hz, 1H), 6.98 (dd, *J* = 8.0, 4.8 Hz, 1H), 6.93 (d, *J* = 7.2 Hz, 1H), 6.78 (d, *J* = 6.4 Hz, 2H), 6.67 (t, *J* = 7.6 Hz, 1H), 6.24 (d, *J* = 7.6 Hz, 1H), 4.62 (s, 1H), 4.36 (s, 1H), 2.97 (d, *J* = 16.8 Hz, 1H), 2.72 (s, 3H), 2.66 (d, *J* = 16.8 Hz, 1H), 2.59 (s, 3H) ppm; $^{13}C\{^1H\}$ NMR (100 MHz,

Chloroform-*d*) δ 171.2, 168.6, 149.4, 148.2, 148.0, 138.1, 135.2, 135.0, 134.9, 129.9, 129.7, 128.4, 127.9, 127.64, 127.62, 127.2, 126.3, 125.1, 121.9, 117.4, 106.9, 86.5, 68.2, 54.9, 38.9, 34.1, 26.6 ppm.

3a-(((diphenylmethylene)amino)(furan-2-yl)methyl)-1,8-dimethyl-3,3a,8,8a-tetrahydropyrrolo[2,3-*b*]indol-2(1*H*)-one (3la)

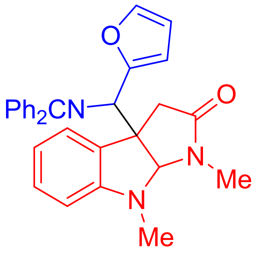

The reaction was performed following the **GP2** with *N*-(furan-2-ylmethyl)-1,1-diphenylmethanimine **1l** (104.4 mg, 0.4 mmol) and *N*-methyl-2-(1-methyl-1*H*-indol-3-yl)-*N*-(4-nitrophenoxy)acetamide **2a** (271.3 mg, 0.8 mmol). The crude product was separated by flash chromatography on deactivated silica gel (ethyl acetate:hexanes = 1:10). Further purification was purified on an Agilent HPLC 1260 system using acetonitrile:H_2O (85:15 vol./vol.) as mobile phase and flow rate of 3.0 mL/min at 254 nm to give the product **3la** in 48% overall yield (88.6 mg, dr = 1.5:1).

3la: colorless oil, R_f = 0.44 (hexanes:ethyl acetate = 1:1); Diastereomeric ratio was determined based on H^a (1H, 4.55 ppm, 5.25 ppm) and H^b (1H, 4.63 ppm, 5.25 ppm), see ^1H spectra (Figure S51, Page S78) for determination of diastereomeric ratio; HRMS calc'd for $C_{30}H_{28}N_3O_2^+$ 462.2176, found 462.2179 $[M+H]^+$.

3a-(((diphenylmethylene)amino)(thiophen-2-yl)methyl)-1,8-dimethyl-3,3a,8,8a-tetrahydropyrrolo[2,3-*b*]indol-2(1*H*)-one (3ma)

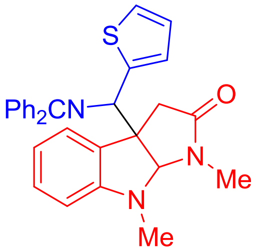

The reaction was performed following the **GP2** with 1,1-diphenyl-*N*-(thiophen-2-ylmethyl)methanimine **1m** (110.8 mg, 0.4 mmol) and *N*-methyl-2-(1-methyl-1*H*-indol-3-yl)-*N*-(4-nitrophenoxy)acetamide **2a**

(271.3 mg, 0.8 mmol). The crude product was separated by flash chromatography on deactivated silica gel (ethyl acetate:hexanes = 1:10). Further purification was purified on an Agilent HPLC 1260 system using acetonitrile:H_2O (80:20 vol./vol.) as mobile phase and flow rate of 3.0 mL/min at 254 nm to give the product **3ma** in 56% overall yield (dr = 1.5:1, **3ma**(major), 64.2 mg, 34% yield; **3ma**(minor), 42.8 mg, 22% yield).

3ma(major): yellow oil, R_f = 0.52 (hexanes:ethyl acetate = 1:1); ^{1}H NMR (600 MHz, Methanol-d_4) δ 7.54 (d, J = 7.2 Hz, 2H), 7.38 – 7.33 (m, 2H), 7.32 – 7.29 (m, 4H), 7.08 (d, J = 5.4 Hz, 1H), 7.02 (t, J = 7.8 Hz, 1H), 6.81 (d, J = 7.2 Hz, 1H), 6.76 (d, J = 6.6 Hz, 2H), 6.68 (dd, J = 5.4, 3.6 Hz, 1H), 6.61 (t, J = 7.2 Hz, 1H), 6.32 (d, J = 7.8 Hz, 1H), 6.11 (d, J = 3.6 Hz, 1H), 5.32 (s, 1H), 4.75 (s, 1H), 2.85 – 2.82 (m, 4H), 2.58 (s, 3H), 2.54 (d, J = 16.8 Hz, 1H) ppm; ^{13}C{^{1}H} NMR (150 MHz, Methanol-d_4) δ 173.6, 170.9, 151.2, 142.5, 139.0, 135.9, 132.5, 130.5, 129.2, 128.7, 128.4, 128.2, 127.9, 126.9, 125.6, 124.0, 123.9, 123.7, 118.5, 108.6, 87.4, 66.6, 55.8, 39.8, 34.8, 26.6 ppm; IR (thin film): 3065, 2927, 1692, 1495, 1346, 1220, 772, 748 cm^{-1}; HRMS calc'd for $C_{30}H_{28}N_3OS^+$:478.1948, found: 478.1949 $[M+H]^+$.

3ma(minor): yellow oil, R_f = 0.42 (hexanes:ethyl acetate = 1:1); ^{1}H NMR (600 MHz, Chloroform-d) δ 7.46 (d, J = 7.8 Hz, 2H), 7.36 (t, J = 7.2 Hz, 1H), 7.33 – 7.31 (m, 3H), 7.24 (t, J = 7.8 Hz, 2H), 7.12 (d, J = 5.4 Hz, 1H), 7.05 (t, J = 7.8 Hz, 1H), 6.83 (t, J = 4.2 Hz, 1H), 6.77 (d, J = 7.2 Hz, 2H), 6.71 (d, J = 7.2 Hz, 1H), 6.61 (t, J = 7.2 Hz, 2H), 6.34 (d, J = 7.8 Hz, 1H), 5.09 (s, 1H), 4.77 (s, 1H), 2.99 (d, J = 16.8 Hz, 1H), 2.88 (s, 3H), 2.78 (s, 3H), 2.58 (d, J = 16.8 Hz, 1H) ppm; ^{13}C{^{1}H} NMR (150 MHz, Chloroform-d) δ 171.5, 169.4, 149.9, 141.0, 137.9, 134.7, 130.9, 129.7, 128.2, 127.9, 127.3, 127.0, 126.8, 124.9, 124.6, 124.22, 124.18, 117.4, 107.0, 86.7, 66.7, 54.8, 39.1, 35.0, 26.9 ppm (one resonance was not observed due to overlapping peaks).

3a-(1-((9*H*-fluoren-9-ylidene)amino)ethyl)-1,8-dimethyl-3,3a,8,8a-tetrahydropyrrolo[2,3-*b*]indol-2(1*H*)-one (3na)

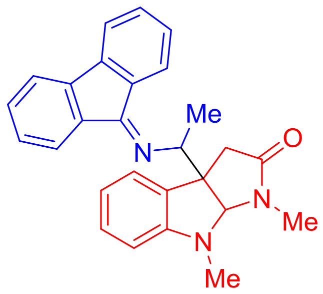

The reaction was performed following the **GP2** with *N*-ethyl-9*H*-fluoren-9-imine **1n** (82.8 mg, 0.4 mmol) and *N*-methyl-2-(1-methyl-1*H*-indol-3-yl)-*N*-(4-nitrophenoxy)acetamide **2a** (271.3 mg, 0.8 mmol). The crude product was separated by flash chromatography on deactivated silica gel (ethyl acetate:hexanes = 1:10). Further purification was purified on an Agilent HPLC 1260 system using acetonitrile:H_2O (70:30 vol./vol.) as mobile phase and flow rate of 3.0 mL/min at 254 nm to give the product **3na** in 78% overall yield (dr = 1.4:1, **3na**(major), 74.2 mg, 46% yield; **3na**(minor), 53.0 mg, 32% yield).

3na(major): yellow oil, R_f = 0.36 (hexanes:ethyl acetate = 1:1); ^{1}H NMR (400 MHz, Chloroform-*d*) δ 7.68 (d, *J* = 7.6 Hz, 1H), 7.62 (t, *J* = 7.6 Hz, 2H), 7.50 (d, *J* = 7.6 Hz, 1H), 7.36 (dt, *J* = 14.8, 7.6 Hz, 2H), 7.22 (t, *J* = 7.2 Hz, 2H), 7.13 (t, *J* = 7.6 Hz, 1H), 7.05 (d, *J* = 7.2 Hz, 1H), 6.71 (t, *J* = 7.2 Hz, 1H), 6.47 (d, *J* = 7.6 Hz, 1H), 5.26 (s, 1H), 4.72 (q, *J* = 6.4 Hz, 1H), 3.07 (s, 3H), 2.95 (s, 3H), 2.73 (d, *J* = 16.8 Hz, 1H), 2.61 (d, *J* = 16.8 Hz, 1H), 1.04 (d, *J* = 6.4 Hz, 3H) ppm; ^{13}C{^{1}H} NMR (100 MHz, Chloroform-*d*) δ 173.2, 162.9, 151.0, 144.1, 140.7, 138.3, 132.9, 131.6, 131.4, 131.1, 129.2, 128.5, 128.1, 127.2, 123.5, 122.7, 120.6, 119.3, 118.7, 107.9, 86.7, 60.7, 55.1, 41.3, 35.6, 28.2, 16.0 ppm; IR (thin film): 3054, 2925, 1687, 1494, 1449, 1399, 1294, 734 cm^{-1}; HRMS calc'd for $C_{27}H_{26}N_3O^+$: 408.2070, found: 408.2068 $[M+H]^+$.

3na(minor): yellow solid, m.p. = 197 – 199 °C , R_f = 0.34 (hexanes:ethyl acetate = 1:1); ^{1}H NMR (600 MHz, Chloroform-*d*) δ 7.72 (d, *J* = 7.2 Hz, 1H), 7.63 (d, *J* = 7.8 Hz, 1H), 7.60 (d, *J* = 7.8 Hz, 1H), 7.51 (d, *J* = 7.2 Hz, 1H), 7.37 – 7.33 (m, 2H), 7.26 – 7.23 (m, 2H), 7.15 (dt, *J* = 23.4, 7.8 Hz, 2H), 6.71 (t, *J* = 7.2 Hz, 1H), 6.42 (d, *J* = 7.8 Hz, 1H), 4.68 (s, 1H), 4.56 (q, *J* = 6.6 Hz, 1H), 3.34 (d, *J* = 16.8 Hz, 1H), 2.97 (s, 3H), 2.81 (s, 3H), 2.70 (d, *J* = 16.8 Hz, 1H), 1.34 (d, *J* = 6.6 Hz, 3H) ppm; ^{13}C{^{1}H} NMR (150 MHz, Chloroform-*d*) δ 173.8, 162.2, 150.6, 144.2, 140.9, 138.7, 132.3, 131.6, 131.4, 131.2, 129.3, 128.6, 128.2, 127.4, 125.5, 123.0, 120.7, 119.4, 118.5, 107.7, 88.7, 59.6, 55.5, 38.2, 35.2, 28.5, 16.5 ppm.

3a-(1-((9*H*-fluoren-9-ylidene)amino)-2-methylpropyl)-1,8-dimethyl-3,3a,8,8a-tetrahydropyrrolo[2,3-*b*]indol-2(1*H*)-one (3oa)

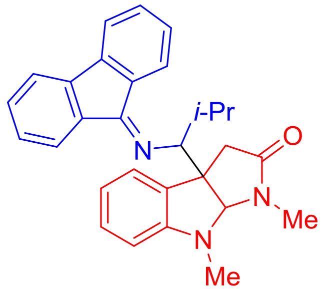

The reaction was performed following the **GP2** with *N*-isobutyl-9*H*-fluoren-9-imine **1o** (94.1 mg, 0.4 mmol) and *N*-methyl-2-(1-methyl-1*H*-indol-3-yl)-*N*-(4-nitrophenoxy)acetamide **2a** (271.5mg, 0.8 mmol). The crude product was separated by flash chromatography on deactivated silica gel (ethyl acetate:hexanes = 1:10). Further purification was purified on an Agilent HPLC 1260 system using acetonitrile:H_2O (80:20 vol./vol.) as mobile phase and flow rate of 3.0 mL/min at 254 nm to give the product **3oa** in 72% overall yield (**3oa**(major), 70.9 mg, 41% yield; **3oa**(minor), 54.4 mg, 31% yield, dr = 1.3:1).

3oa(major): yellow oil, R_f = 0.46 (hexanes:ethyl acetate = 1:1); ^{1}H NMR (600 MHz, Methanol-d_4) δ 8.05 (d, *J* = 7.8 Hz, 1H), 7.66 (d, *J* = 7.2 Hz, 1H), 7.60 (d, *J* = 7.2 Hz, 1H), 7.56 (d, *J* = 7.2 Hz, 1H), 7.36 (t, *J* = 7.8 Hz, 1H), 7.33 (td, *J* = 7.2, 1.2 Hz, 1H), 7.24 (t, *J* = 7.8 Hz, 1H), 7.19 (t, *J* = 7.2 Hz, 1H), 7.11 (d, *J* = 7.2 Hz, 1H), 7.07 (td, *J* = 7.8, 1.2 Hz, 1H), 6.66 (t, *J* = 7.2 Hz, 1H), 6.46 (d, *J* = 7.8 Hz, 1H), 5.54 (s, 1H), 4.86 (d, *J* = 4.8 Hz, 1H), 3.04 (s, 3H), 2.82 (s, 3H), 2.54 (s, 2H), 1.96 – 1.91 (m, 1H), 0.76 (d, *J* = 6.6 Hz, 3H), 0.70 (d, *J* = 6.6 Hz, 3H) ppm; ^{13}C{^{1}H} NMR (150 MHz, Methanol-d_4) δ 175.2, 164.4, 152.0, 145.8, 142.2, 139.6, 133.6, 133.4, 132.8, 132.4, 130.3, 129.4, 129.3, 128.6, 125.2, 123.5, 121.7, 120.5, 119.6, 108.7, 88.5, 70.7, 56.1, 44.2, 35.7, 32.6, 28.7, 22.8, 18.7 ppm; IR (thin film): 3096, 2966, 1690, 1498, 1454, 1055, 1011 cm^{-1}; HRMS calc'd for $C_{29}H_{30}N_3O^+$: 436.2383, found: 436.2380 [M+H]$^+$.

3oa(minor): yellow oil, R_f = 0.44 (hexanes:ethyl acetate = 1:1); ^{1}H NMR (600 MHz, Methanol-d_4) δ 7.93 (d, *J* = 7.8 Hz, 1H), 7.68 (d, *J* = 7.2 Hz, 1H), 7.64 (d, *J* = 7.8 Hz, 1H), 7.55 (d, *J* = 7.8 Hz, 1H), 7.33 (q, 6.6 Hz, 2H), 7.24 – 7.16 (m, 3H), 7.04 (td, *J* = 7.8, 1.2 Hz, 1H), 6.65 (t, *J* = 7.8 Hz, 1H), 6.43 (d, *J* = 7.8 Hz, 1H), 4.80 (s, 1H), 4.71 (d, *J* = 3.0 Hz, 1H), 3.31 (d, *J* = 16.8 Hz, 1H), 2.96 (s, 3H), 2.65 (s, 3H), 2.60 (d, *J* = 16.8 Hz, 1H), 2.29 – 2.24 (m, 1H), 0.94 (d, *J* = 6.6 Hz, 3H), 0.69 (d, *J* = 6.6 Hz, 3H) ppm; ^{13}C{^{1}H} NMR (150 MHz, Methanol-d_4) δ 175.6, 163.9, 151.2, 145.9, 142.1, 139.6, 133.39, 133.37, 132.7, 132.3, 130.5, 129.4, 129.2, 128.6, 126.7, 123.6, 121.6, 120.4, 119.6, 109.0, 90.1, 68.1, 56.8, 40.1, 35.2, 32.2, 28.3, 22.6, 17.1 ppm.

3a-(1-((9*H*-fluoren-9-ylidene)amino)-3-methylbutyl)-1,8-dimethyl-3,3a,8,8a-tetrahydropyrrolo[2,3-*b*]indol-2(1*H*)-one (3pa)

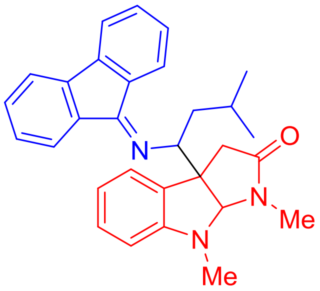

The reaction was performed following the **GP2** with (*E*)-*N*-(9*H*-fluoren-9-yl)-3-methylbutan-1-imine **1p** (99.7 mg, 0.4 mmol) and *N*-methyl-2-(1-methyl-1*H*-indol-3-yl)-*N*-(4-nitrophenoxy)acetamide **2a** (271.3 mg, 0.8 mmol). The crude product was separated by flash chromatography on deactivated silica gel (ethyl acetate:hexanes = 1:5). Further purification was performed on an Agilent HPLC 1260 system using acetonitrile:H_2O (80:20 vol./vol.) as mobile phase and flow rate of 3.0 mL/min at 254 nm to give the product **3pa** in 76% overall yield (136.7 mg, dr = 1.5:1).

3pa: colorless oil, R_f = 0.53 (hexanes:ethyl acetate = 1:1); Diastereomeric ratio was determined based on H^a (1H, 5.26 ppm) and H^b (1H, 4.63 ppm), see ^1H spectra (Figure S65, Page S85) for determination of diastereomeric ratio; HRMS calc'd for $C_{30}H_{32}N_3O^+$: 450.2540, found: 450.2539 $[M+H]^+$.

3a-(((9*H*-fluoren-9-ylidene)amino)(cyclobutyl)methyl)-1,8-dimethyl-3,3a,8,8a-tetrahydropyrrolo[2,3-*b*]indol-2(1*H*)-one (3qa)

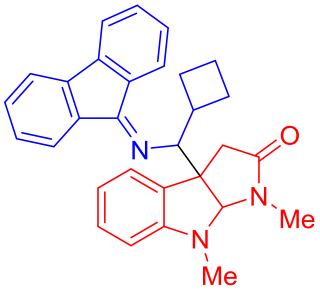

The reaction was performed following the **GP2** with (*E*)-1-cyclobutyl-*N*-(9*H*-fluoren-9-yl)methanimine **1q** (98.9 mg, 0.4 mmol) and *N*-methyl-2-(1-methyl-1*H*-indol-3-yl)-*N*-(4-nitrophenoxy)acetamide **2a** (271.3 mg, 0.8 mmol). The crude product was separated by flash chromatography on deactivated silica gel (ethyl acetate:hexanes = 1:5). Further purification was performed on an Agilent HPLC 1260 system

using acetonitrile:H_2O (80:20 vol./vol.) as mobile phase and flow rate of 3.0 mL/min at 254 nm to give the product **3qa** in 86% overall yield (dr = 1.4:1, **3qa**(major), 89.8 mg, 50% yield; **3qa**(minor), 64.2 mg, 36% yield).

3qa(major): yellow oil, R_f = 0.57 (hexanes:ethyl acetate = 1:1); ^{1}H NMR (400 MHz, Chloroform-*d*) δ 7.94 (d, *J* = 7.6 Hz, 1H), 7.62 (dd, *J* = 7.6, 4.8 Hz, 2H), 7.51 (d, *J* = 7.2 Hz, 1H), 7.39 – 7.32 (m, 2H), 7.25 – 7.19 (m, 2H), 7.11 (td, *J* = 7.6, 1.2 Hz, 1H), 7.03 (dd, *J* = 7.2, 1.2 Hz, 1H), 6.69 (t, *J* = 7.2 Hz, 1H), 6.42 (d, *J* = 7.6 Hz, 1H), 5.37 (s, 1H), 4.82 (d, *J* = 7.2 Hz, 1H), 3.07 (s, 3H), 2.86 (s, 3H), 2.60 – 2.50 (m, 3H), 1.84 – 1.74 (m, 1H), 1.61 – 1.51 (m, 2H), 1.49 – 1.36 (m, 2H), 1.25 – 1.17 (m, 1H) ppm. ^{13}C NMR (100 MHz, Chloroform-*d*) δ 171.7, 161.7, 149.5, 143.4, 139.7, 137.4, 131.5, 130.4, 130.1, 128.1, 127.3, 126.9, 126.3, 122.9, 121.8, 119.6, 118.2, 117.2, 106.4, 85.0, 67.9, 53.2, 40.4, 37.1, 34.2, 27.1, 26.4, 17.8 ppm (one resonance was not observed due to overlapping peaks); IR (thin film): 3056, 2932, 1688, 1606, 1495, 1279, 793, 733 cm^{-1}, HRMS calc'd for $C_{30}H_{30}N_3O^+$: 448.2383, found: 448.2379 $[M+H]^+$.

3qa(minor): yellow oil, R_f = 0.43 (hexanes:ethyl acetate = 1:1); ^{1}H NMR (400 MHz, Chloroform-*d*) δ 7.79 (d, *J* = 7.2 Hz, 1H), 7.73 (d, *J* = 7.6 Hz, 1H), 7.62 (d, *J* = 7.6 Hz, 1H), 7.55 (d, *J* = 7.2 Hz, 1H), 7.40 – 7.33 (m, 2H), 7.30 – 7.24 (m, 2H), 7.16 – 7.10 (m, 2H), 6.72 (t, *J* = 7.2 Hz, 1H), 6.40 (d, *J* = 7.6 Hz, 1H), 4.62 (s, 1H), 4.58 (d, *J* = 4.4 Hz, 1H), 3.32 (d, *J* = 16.8 Hz, 1H), 2.95 (s, 3H), 2.73 (s, 3H), 2.59 (d, *J* = 16.8 Hz, 1H), 2.18 – 2.10 (m, 1H), 1.76 – 1.62 (m, 4H), 1.51 – 1.49 (m, 2H) ppm. ^{13}C NMR (100 MHz, Chloroform-*d*) δ 173.6, 162.4, 150.0, 144.4, 140.8, 138.5, 132.3, 132.2, 131.3, 131.1, 129.2, 128.5, 127.9, 127.4, 125.8, 123.0, 120.5, 119.3, 118.3, 107.5, 88.2, 65.2, 55.1, 38.2, 37.9, 34.9, 28.3, 26.5, 19.2 ppm.

3a-(((9*H*-fluoren-9-ylidene)amino)(cyclopentyl)methyl)-1,8-dimethyl-3,3a,8,8a-tetrahydropyrrolo[2,3-*b*]indol-2(1*H*)-one (3ra)

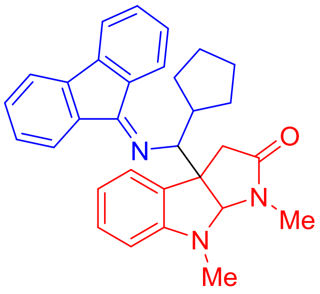

The reaction was performed following the **GP2** with (*E*)-1-cyclopentyl-*N*-(9*H*-fluoren-9-yl)methanimine **1r** (104.5 mg, 0.4 mmol) and *N*-methyl-2-(1-methyl-1*H*-indol-3-yl)-*N*-(4-nitrophenoxy)acetamide **2a** (271.3 mg, 0.8 mmol). The crude product was separated by flash chromatography on deactivated silica gel (ethyl acetate:hexanes = 1:5). Further purification was performed on an Agilent HPLC 1260 system using acetonitrile:H_2O (80:20 vol./vol.) as mobile phase and flow rate of 3.0 mL/min at 254 nm to give the product **3ra** in 82% overall yield (dr = 1.5:1, **3ra**(major), 90.8 mg, 49% yield; **3ra**(minor), 60.6 mg, 33% yield).

3ra(major): yellow oil, R_f = 0.58 (hexanes:ethyl acetate = 1:1); ^{1}H NMR (400 MHz, Chloroform-*d*) δ 7.89 (d, *J* = 7.6 Hz, 1H), 7.62 (t, *J* = 8.0 Hz, 2H), 7.52 (d, *J* = 7.2 Hz, 1H), 7.40 – 7.33 (m, 2H), 7.25 – 7.21 (m, 2H), 7.15 – 7.11 (m, 1H), 7.05 (dd, *J* = 7.2, 1.2 Hz, 1H), 6.71 (td, *J* = 7.6, 1.2 Hz, 1H), 6.43 (d, *J* = 7.6 Hz, 1H), 5.37 (s, 1H), 4.86 (d, *J* = 6.0 Hz, 1H), 3.05 (s, 3H), 2.85 (s, 3H), 2.60 (s, 2H), 2.04 – 1.98 (m, 1H), 1.40 – 1.31 (m, 4H), 1.28 – 1.23 (m, 3H), 0.99 – 0.91 (m, 1H) ppm. ^{13}C NMR (100 MHz, Chloroform-*d*) δ 172.7, 162.2, 150.7, 144.5, 140.7, 138.5, 132.8, 132.5, 131.4, 131.1, 129.2, 128.3, 128.0, 127.3, 124.1, 122.8, 120.7, 119.3, 118.3, 107.4, 86.5, 68.8, 54.8, 42.9, 42.1, 35.3, 31.4, 28.2, 24.9 ppm; IR (thin film): 3056, 2925, 1686, 1606, 1495, 1297, 793, 733 cm^{-1}, HRMS calc'd for $C_{31}H_{32}N_3O^+$: 462.2540, found: 462.2535 $[M+H]^+$.

3ra(minor): yellow oil, R_f = 0.52 (hexanes:ethyl acetate = 1:1); ^{1}H NMR (400 MHz, Chloroform-*d*) δ 7.81 (d, *J* = 7.6 Hz, 1H), 7.75 (d, *J* = 7.2 Hz, 1H), 7.63 (d, *J* = 7.6 Hz, 1H), 7.54 (d, *J* = 7.2 Hz, 1H), 7.40 – 7.34 (m, 2H), 7.29 – 7.22 (m, 2H), 7.17 – 7.12 (m, 2H), 6.74 (td, *J* = 7.6, 1.2 Hz, 1H), 6.43 (d, *J* = 7.6 Hz, 1H), 4.77 (d, *J* = 2.8 Hz, 1H), 4.57 (s, 1H), 3.46 (d, *J* = 16.8 Hz, 1H), 2.96 (s, 3H), 2.68 – 2.64 (m, 4H), 2.44 – 2.35 (m, 1H), 1.89 – 1.79 (m, 1H), 1.49 – 1.39 (m, 4H), 1.30 (q, *J* = 7.2 Hz, 2H), 1.04 – 0.94 (m, 1H) ppm. ^{13}C NMR (100 MHz, Chloroform-*d*) δ 172.5, 161.2, 148.7, 143.6, 139.7, 137.5, 131.28, 131.26, 130.3, 130.0, 128.2, 127.4, 126.9, 126.2, 124.9, 122.0, 119.5, 118.2, 117.3, 106.4, 87.3, 62.8, 54.6, 41.2, 37.1, 33.4, 30.0, 24.8, 24.6 ppm.

3a-(((9*H*-fluoren-9-ylidene)amino)(cyclohexyl)methyl)-1,8-dimethyl-3,3a,8,8a-tetrahydropyrrolo[2,3-*b*]indol-2(1*H*)-one (3sa)

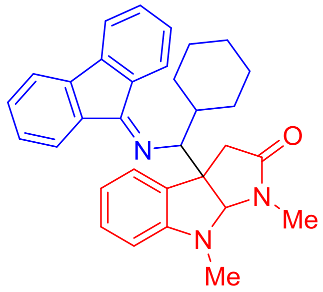

The reaction was performed following the **GP2** with (*E*)-1-cyclohexyl-*N*-(9*H*-fluoren-9-yl)methanimine **1s** (110.2 mg, 0.4 mmol) and *N*-methyl-2-(1-methyl-1*H*-indol-3-yl)-*N*-(4-nitrophenoxy)acetamide **2a** (271.3 mg, 0.8 mmol). The crude product was separated by flash chromatography on deactivated silica gel (ethyl acetate:hexanes = 1:8). Further purification was performed on an Agilent HPLC 1260 system using acetonitrile:H_2O (80:20 vol./vol.) as mobile phase and flow rate of 3.0 mL/min at 254 nm to give the product **3sa** in 88% overall yield (dr = 1.5:1, **3sa**(major), 100.4 mg, 53% yield; **3sa**(minor), 67.0 mg, 35% yield).

3sa(major): yellow oil, R_f = 0.63 (hexanes:ethyl acetate = 1:1); ^{1}H NMR (400 MHz, Chloroform-*d*) δ 7.92 (d, *J* = 7.6 Hz, 1H), 7.61 (dd, *J* = 7.6, 5.2 Hz, 2H), 7.52 (d, *J* = 7.2 Hz, 1H), 7.38 – 7.32 (m, 2H), 7.23 – 7.18 (m, 2H), 7.12 (td, *J* = 7.6, 1.2 Hz, 1H), 7.03 (d, *J* = 7.6 Hz, 1H), 6.70 (t, *J* = 7.6 Hz, 1H), 6.43 (d, *J* = 7.6 Hz, 1H), 5.32 (s, 1H), 4.72 (d, *J* = 4.4 Hz, 1H), 3.02 (s, 3H), 2.82 (s, 3H), 2.63 (s, 2H), 1.64 – 1.59 (m, 2H), 1.48 – 1.40 (m, 3H), 1.10 – 0.81 (m, 6H) ppm. ^{13}C NMR (100 MHz, Chloroform-*d*) δ 172.7, 162.3, 150.7, 144.6, 140.7, 138.5, 132.6, 132.4, 131.4, 131.0, 129.2, 128.3, 128.0, 127.2, 124.0, 122.8, 120.6, 119.3, 118.4, 107.4, 86.96, 70.0, 54.8, 42.7, 41.5, 35.4, 32.9, 28.1, 26.6, 26.4 ppm; IR (thin film): 3056, 2925, 1686, 1606, 1495, 1449, 1279, 793, 732 cm^{-1}, HRMS calc'd for $C_{32}H_{34}N_3O^+$: 476.2696, found: 476.2696 $[M+H]^+$.

3sa(minor): yellow oil, R_f = 0.62 (hexanes:ethyl acetate = 1:1); ^{1}H NMR (400 MHz, Chloroform-*d*) δ 7.85 (d, *J* = 7.6 Hz, 1H), 7.76 (d, *J* = 7.2 Hz, 1H), 7.62 (d, *J* = 7.6 Hz, 1H), 7.53 (d, *J* = 7.2 Hz, 1H), 7.39 – 7.32 (m, 2H), 7.28 – 7.24 (m, 1H), 7.19 – 7.17 (m, 1H), 7.15 – 7.11 (m, 2H), 6.74 (td, *J* = 7.6, 1.2 Hz, 1H), 6.42 (d, *J* = 7.6 Hz, 1H), 4.58 (d, *J* = 2.4 Hz, 1H), 4.53 (s, 1H), 3.57 (d, *J* = 16.8 Hz, 1H), 2.95 (s, 3H), 2.71 (d, *J* = 16.8 Hz, 1H), 2.64 (s, 3H), 1.96 – 1.87 (m, 2H), 1.71 (d, *J* = 13.6 Hz, 1H), 1.57 – 1.42 (m, 3H), 1.28 (d, *J* = 12.8 Hz, 1H), 1.23 – 1.14 (m, 1H), 1.13 – 1.01 (m, 1H), 0.96 – 0.83 (m, 2H) ppm. ^{13}C NMR (100 MHz, Chloroform-*d*) δ 173.6, 162.0, 149.7, 144.7, 140.6, 138.5, 132.4, 132.2, 131.3,

131.1, 129.3, 128.5, 127.9, 127.1, 125.9, 123.1, 120.6, 119.2, 118.5, 107.5, 88.6, 66.5, 55.4, 41.1, 38.2, 34.4, 31.9, 28.2, 26.8, 26.48 ppm.

3a-(((diphenylmethylene)amino)(phenyl)methyl)-1,7,8-trimethyl-3,3a,8,8a-tetrahydropyrrolo[2,3-*b*]indol-2(1*H*)-one (3ab)

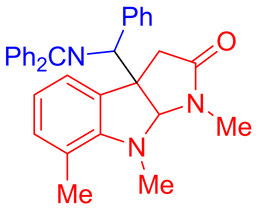

The reaction was performed following the **GP2** with *N*-benzyl-1,1-diphenylmethanimine **1a** (108.5 mg, 0.4 mmol) and 2-(1, 7-dimethyl-1*H*-indol-3-yl)-*N*-methyl-*N*-(4-nitrophenoxy)acetamide **2b** (282.5 mg, 0.8 mmol). The crude product was separated by flash chromatography on deactivated silica gel (ethyl acetate:hexanes = 1:10). Further purification was purified on an Agilent HPLC 1260 system using acetonitrile:H_2O (80:20 vol./vol.) as mobile phase and flow rate of 3.0 mL/min at 254 nm to give the product **3ab** in 63% overall yield (**3ab**(major), 82.9 mg, 43% yield; **3ab**(minor), 39.5 mg, 20% yield, dr = 2.1:1).

3ab(major): colorless oil, R_f = 0.65 (hexanes:ethyl acetate = 1:1); ^{1}H NMR (600 MHz, Chloroform-*d*) δ 7.60 (d, *J* = 7.2 Hz, 2H), 7.34 (t, *J* = 7.8 Hz, 1H), 7.31 – 7.27 (m, 3H), 7.24 (t, *J* = 7.8 Hz, 2H), 7.03 – 7.00 (m, 3H), 6.85 (d, *J* = 4.8 Hz, 2H), 6.80 (d, *J* = 7.2 Hz, 1H), 6.72 – 6.69 (m, 3H), 6.66 (t, *J* = 7.2 Hz, 1H), 5.06 (s, 1H), 4.48 (s, 1H), 2.85 (d, *J* = 16.8 Hz, 1H), 2.79 (s, 3H), 2.49 (d, *J* = 16.8 Hz, 1H), 2.24 (s, 3H), 2.02 (s, 3H) ppm; $^{13}C\{^1H\}$ NMR (150 MHz, Chloroform-*d*) δ 171.1, 168.2, 149.2, 140.0, 138.2, 135.3, 134.6, 130.3, 129.5, 127.7, 127.6, 127.3, 127.2, 127.0, 126.7, 126.3, 126.2, 122.2, 121.1, 120.1, 88.1, 68.9, 55.7, 39.4, 38.5, 25.7, 17.5 ppm; IR (thin film): 3059, 2962, 1694, 1597, 1470, 1447, 1415, 1262, 913, 748 cm^{-1}; HRMS calc'd for $C_{33}H_{32}N_3O^+$: 486.2540, found: 486.2537 $[M+H]^+$.

3ab(minor): colorless oil, R_f = 0.55 (hexanes:ethyl acetate = 1:1); ^{1}H NMR (600 MHz, Chloroform-*d*) δ 7.58 (d, *J* = 7.2 Hz, 2H), 7.34 – 7.32 (m, 2H), 7.30 – 7.26 (m, 4H), 7.15 –7.10 (m, 3H), 6.90 (d, *J* = 7.2 Hz, 2H), 6.85 (d, *J* = 7.8 Hz, 1H), 6.80 (d, *J* = 7.2 Hz, 2H), 6.68 (t, *J* = 7.2 Hz, 1H), 6.59 (d, *J* = 7.2 Hz, 1H), 4.55 (s, 1H), 4.36 (s, 1H), 3.17 (d, *J* = 16.8 Hz, 1H), 2.71 (s, 3H), 2.51 – 2.48 (m, 4H), 2.10 (s, 3H) ppm; $^{13}C\{^1H\}$ NMR (150 MHz, Chloroform-*d*) δ 171.2, 167.4, 149.2, 139.4, 138.5, 135.6, 133.8, 130.2,

129.3, 129.0, 127.8, 127.7, 127.6, 127.3, 127.1, 126.9, 126.8, 126.6, 123.1, 122.5, 120.3, 89.3, 71.0, 55.9, 39.2, 25.6, 17.4 ppm.

3a-(((diphenylmethylene)amino)(phenyl)methyl)-5-methoxy-1,8-dimethyl-3,3a,8,8a-tetrahydropyrrolo[2,3-*b*]indol-2(1*H*)-one (3ac)

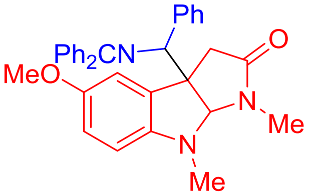

The reaction was performed following the **GP2** with *N*-benzyl-1,1-diphenylmethanimine **1a** (108.5 mg, 0.4 mmol) and 2-(5-methoxy-1-methyl-1*H*-indol-3-yl)-*N*-methyl-*N*-(4-nitrophenoxy)acetamide **2c** (295.3 mg, 0.8 mmol). The crude product was separated by flash chromatography on deactivated silica gel (ethyl acetate:hexanes = 1:10). Further purification was purified on an Agilent HPLC 1260 system using acetonitrile:H_2O (80:20 vol./vol.) as mobile phase and flow rate of 3.0 mL/min at 254 nm to give the product **3ac** in 65% overall yield (**3ac**(major), 68.3 mg, 34% yield; **3ac**(minor), 62.1 mg, 31% yield, dr = 1.1:1).

3ac(major): colorless oil, R_f = 0.30 (hexanes:ethyl acetate = 1:1); ^{1}H NMR (600 MHz, Chloroform-*d*) δ 7.58 (d, *J* = 7.2 Hz, 2H), 7.35 (t, *J* = 7.2 Hz, 1H), 7.32 – 7.27 (m, 3H), 7.25 (t, *J* = 7.8 Hz, 2H), 7.04 – 7.00 (m, 3H), 6.84 (d, *J* = 5.4 Hz, 2H), 6.72 (d, *J* = 7.2 Hz, 2H), 6.58 (dd, *J* = 8.4, 2.4 Hz, 1H), 6.45 (d, *J* = 2.4 Hz, 1H), 6.12 (d, *J* = 8.4 Hz, 1H), 5.14 (s, 1H), 4.42 (s, 1H), 3.58 (s, 3H), 2.86 (d, *J* = 16.8 Hz, 1H), 2.78 (s, 3H), 2.55 (d, *J* = 16.8 Hz, 1H), 2.37 (s, 3H) ppm; $^{13}C\{^1H\}$ NMR (150 MHz, Chloroform-*d*) δ 171.3, 168.3, 152.3, 144.4, 139.7, 138.3, 135.3, 133.6, 129.5, 127.7, 127.6, 127.3, 127.2, 126.9, 126.6, 126.3, 126.2, 113.2, 110.5, 108.4, 86.4, 68.9, 55.4, 55.1, 38.9, 35.6, 26.3 ppm; IR (thin film): 3060, 2962, 1692, 1598, 1497, 1446, 1314, 1262, 803, 704 cm^{-1}; HRMS calc'd for $C_{33}H_{32}N_3O_2^+$: 502.2489, found: 502.2487 $[M+H]^+$.

3ac(minor): yellow oil, R_f = 0.49 (hexanes:ethyl acetate = 1:1); ^{1}H NMR (600 MHz, Chloroform-*d*) δ 7.56 (d, *J* = 8.4 Hz, 2H), 7.35 – 7.28 (m, 4H), 7.26 (t, *J* = 7.8 Hz, 2H), 7.15 – 7.10 (m, 3H), 6.88 (d, *J* = 8.4 Hz, 2H), 6.78 (d, *J* = 7.2 Hz, 2H), 6.63 (dd, *J* = 8.4, 2.4 Hz, 1H), 6.37 (s, 1H), 6.25 (d, *J* = 8.4 Hz, 1H), 4.68 (s, 1H), 4.32 (s, 1H), 3.60 (s, 3H), 3.07 (d, *J* = 16.8 Hz, 1H), 2.71 (s, 3H), 2.56 (s, 3H), 2.55 (d, *J* = 16.8 Hz, 1H) ppm; $^{13}C\{^1H\}$ NMR (150 MHz, Chloroform-*d*) δ 171.4, 167.4, 152.3, 144.1, 139.2,

138.5, 135.5, 132.5, 129.3, 127.64, 127.61, 127.3, 127.1, 126.9, 126.69, 126.66, 114.1, 111.1, 108.6, 87.7, 70.7, 55.5, 54.9, 39.0, 36.2, 26.4 ppm (one resonance was not observed due to overlapping peaks).

5-(benzyloxy)-3a-(((diphenylmethylene)amino)(phenyl)methyl)-1,8-dimethyl-3,3a,8,8a-tetrahydropyrrolo[2,3-*b*]indol-2(1*H*)-one (3ad)

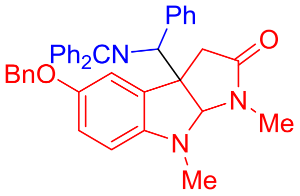

The reaction was performed following the **GP2** with *N*-benzyl-1,1-diphenylmethanimine **1a** (108.5 mg, 0.4 mmol) and 2-(5-(benzyloxy)-1-methyl-1*H*-indol-3-yl)-*N*-methyl-*N*-(4-nitrophenoxy)acetamide **2d** (356.1 mg, 0.8 mmol). The crude product was separated by flash chromatography on deactivated silica gel (ethyl acetate:hexanes = 1:10). Further purification was purified on an Agilent HPLC 1260 system using acetonitrile:H_2O (80:20 vol./vol.) as mobile phase and flow rate of 3.0 mL/min at 254 nm to give the product **3ad** in 60% overall yield (**3ad**(major), 75.6 mg, 33% yield; **3ad**(minor), 63.0 mg, 27% yield, dr = 1.2:1).

3ad(major): colorless oil, R_f = 0.58 (hexanes:ethyl acetate = 1:1); ^{1}H NMR (600 MHz, Chloroform-*d*) δ 7.58 (d, *J* = 7.2 Hz, 2H), 7.36 – 7.29 (m, 5H), 7.26 – 7.21 (m, 6H), 7.02 (t, *J* = 7.2 Hz, 1H), 6.98 (t, *J* = 7.2 Hz, 2H), 6.76 (d, *J* = 7.2 Hz, 2H), 6.70 (d, *J* = 7.2 Hz, 2H), 6.64 (dd, *J* = 8.4, 2.4 Hz, 1H), 6.51 (d, *J* = 2.4 Hz, 1H), 6.08 (d, *J* = 8.4 Hz, 1H), 5.16 (s, 1H), 4.84 (d, *J* = 11.4 Hz, 1H), 4.78 (d, *J* = 11.4 Hz, 1H), 4.38 (s, 1H), 2.83 (d, *J* = 16.8 Hz, 1H), 2.78 (s, 3H), 2.53 (d, *J* = 16.8 Hz, 1H), 2.37 (s, 3H) ppm; ^{13}C{^{1}H} NMR (150 MHz, Chloroform-*d*) δ 171.4, 168.3, 151.2, 144.6, 139.6, 138.3, 136.4, 135.3, 133.5, 129.5, 127.7, 127.6, 127.5, 127.3, 127.2, 126.9, 126.8, 126.5, 126.3, 126.1, 114.8, 111.7, 108.3, 86.3, 70.2, 68.8, 55.4, 39.0, 35.5, 26.4 ppm (one resonance was not observed due to overlapping peaks); IR (thin film): 3060, 2961, 1693, 1498, 1446, 1397, 1261, 1027, 803, 704 cm^{-1}, HRMS calc'd for $C_{39}H_{36}N_3O_2^+$: 578.2802, found: 578.2798 [M+H]$^+$.

3ad(minor): colorless oil, R_f = 0.51 (hexanes:ethyl acetate = 1:1); ^{1}H NMR (600 MHz, Chloroform-*d*) δ 7.56 (d, *J* = 7.8 Hz, 2H), 7.34 – 7.22 (m, 11H), 7.15 – 7.09 (m, 3H), 6.87 (d, *J* = 7.2 Hz, 2H), 6.78 (d, *J* = 7.2 Hz, 2H), 6.70 (d, *J* = 8.4 Hz, 1H), 6.50 (s, 1H), 6.23 (d, *J* = 8.4 Hz, 1H), 4.84 (s, 2H), 4.70 (s, 1H), 4.34 (s, 1H), 3.03 (d, *J* = 16.8 Hz, 1H), 2.71 (s, 3H), 2.57 – 2.54 (m, 4H) ppm; ^{13}C{^{1}H} NMR (150 MHz,

Chloroform-*d*) δ 171.4, 167.5, 151.4, 144.4, 139.2, 138.5, 136.5, 135.5, 132.4, 129.3, 127.6, 127.5, 127.3, 127.1, 126.9, 126.8, 126.69, 126.66, 126.5, 115.4, 112.7, 108.4, 87.6, 70.8, 70.1, 55.4, 39.1, 36.1, 26.4 ppm (two resonance was not observed due to overlapping peaks).

3a-(((diphenylmethylene)amino)(phenyl)methyl)-5-fluoro-1,8-dimethyl-3,3a,8,8a-tetrahydropyrrolo[2,3-*b*]indol-2(1*H*)-one (3ae)

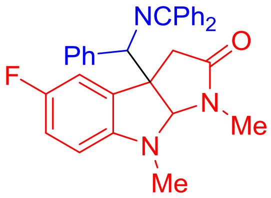

The reaction was performed following the **GP2** with *N*-benzyl-1,1-diphenylmethanimine **1a** (108.5 mg, 0.4 mmol) and 2-(5-fluoro-1-methyl-1*H*-indol-3-yl)-*N*-methyl-*N*-(4-nitrophenoxy)acetamide **2e** (285.7 mg, 0.8 mmol). The crude product was separated by flash chromatography on deactivated silica gel (ethyl acetate:hexanes = 1:10). Further purification was purified on an Agilent HPLC 1260 system using acetonitrile:H_2O (80:20 vol./vol.) as mobile phase and flow rate of 3.0 mL/min at 254 nm to give the product **3ae** in 58% overall yield (**3ae'**, 56.8 mg, 29% yield; **3ae"**, 56.8 mg, 29% yield, dr = 1:1).

3ae': colorless oil, R_f = 0.58 (hexanes:ethyl acetate = 1:1); ^{1}H NMR (600 MHz, Chloroform-*d*) δ 7.57 (d, *J* = 7.2 Hz, 2H), 7.35 (dd, *J* = 16.2, 8.4 Hz, 2H), 7.31 – 7.25 (m, 4H), 7.04 – 7.00 (m, 3H), 6.82 (d, *J* = 7.2 Hz, 2H), 6.74 (d, *J* = 7.2 Hz, 2H), 6.68 (td, *J* = 8.4, 2.4 Hz, 1H), 6.56 (dd, *J* = 8.4, 2.4 Hz, 1H), 6.04 (dd, *J* = 8.4, 4.2 Hz, 1H), 5.23 (s, 1H), 4.40 (s, 1H), 2.84 (d, *J* = 16.8 Hz, 1H), 2.80 (s, 3H), 2.52 (d, *J* = 16.8 Hz, 1H), 2.41 (s, 3H) ppm; ^{13}C{^{1}H} NMR (150 MHz, Chloroform-*d*) δ 171.2, 168.7, 155.7 (d, $^{1}J_{C-F}$ = 234.6 Hz), 146.1, 139.4, 138.2, 135.2, 133.5 (d, $^{3}J_{C-F}$ = 7.7 Hz), 129.6, 127.72, 127.69, 127.4, 127.2, 126.7, 126.6, 126.3, 114.1 (d, $^{2}J_{C-F}$ = 23.1 Hz), 110.6 (d, $^{2}J_{C-F}$ = 24.2 Hz), 107.7 (d, ^{3}J = 8.1 Hz), 86.2, 68.8, 55.2 (d, ^{4}J = 1.5 Hz), 38.9, 35.1, 26.4 ppm (one resonance was not observed due to overlapping peaks); ^{19}F NMR (565 MHz, Chloroform-*d*) δ -126.28 ppm.

3ae": colorless oil, R_f = 0.49 (hexanes:ethyl acetate = 1:1); ^{1}H NMR (600 MHz, Chloroform-*d*) δ 7.53 (d, *J* = 7.2 Hz, 2H), 7.35 – 7.25 (m, 6H), 7.14 – 7.09 (m, 3H)), 6.86 (d, *J* = 7.2 Hz, 2H), 6.77 – 6.72 (m, 3H), 6.61 (d, *J* = 8.4 Hz, 1H), 6.17 (q, *J* =4.2 Hz, 1H), 4.77 (s, 1H), 4.33 (s, 1H), 2.93 (d, *J* = 16.8 Hz, 1H), 2.70 (s, 3H), 2.56 – 2.53 (m, 4H) ppm; ^{13}C{^{1}H} NMR (150 MHz, Chloroform-*d*) δ 171.2, 167.9, 155.7 (d, $^{1}J_{C-F}$ = 234.2 Hz), 146.1, 138.8, 138.3, 135.3, 132.7 (d, $^{3}J_{C-F}$ = 8.0 Hz), 129.4, 127.7, 127.6,

127.4, 127.3, 127.1, 127.0, 126.8, 126.6, 114.1 (d, $^{2}J_{C\text{-}F}$ = 23.1 Hz), 112.3 (d, $^{2}J_{C\text{-}F}$ = 24.6 Hz), 107.6 (d, $^{3}J_{C\text{-}F}$ = 8.3 Hz), 87.3, 70.7, 55.2 (d, ^{4}J = 2.0 Hz), 39.2, 35.5, 26.5 ppm; ^{19}F NMR (565 MHz, Chloroform-*d*) δ -126.10 ppm; IR (thin film):3061, 2926, 1694, 1492, 1419, 1261, 1089, 1028, 802, 704 cm^{-1}, HRMS calc'd for $C_{32}H_{29}FN_3O^+$: 490.2289, found: 490.2290 [M+H]$^+$.

5-bromo-3a-(((diphenylmethylene)amino)(phenyl)methyl)-1,8-dimethyl-3,3a,8,8a-tetrahydropyrrolo[2,3-*b*]indol-2(1*H*)-one (3af)

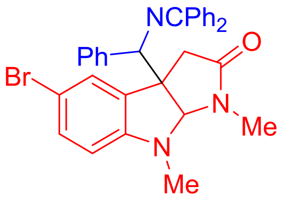

The reaction was performed following the **GP2** with *N*-benzyl-1,1-diphenylmethanimine **1a** (108.5 mg, 0.4 mmol) and 2-(5-bromo-1-methyl-1*H*-indol-3-yl)-*N*-methyl-*N*-(4-nitrophenoxy)acetamide **2f** (333.6 mg, 0.8 mmol). The crude product was separated by flash chromatography on deactivated silica gel (ethyl acetate:hexanes = 1:10). Further purification was purified on an Agilent HPLC 1260 system using acetonitrile:H_2O (80:20 vol./vol.) as mobile phase and flow rate of 3.0 mL/min at 254 nm to give the product **3af** in 60% overall yield (**3af**(major), 74.7 mg, 34% yield; **3af**(minor), 57.4 mg, 26% yield, dr =1.3:1).

3af(major): colorless oil, R_f = 0.56 (hexanes:ethyl acetate = 1:1); ^{1}H NMR (600 MHz, Chloroform-*d*) δ 7.56 (d, *J* = 7.2 Hz, 2H), 7.37 – 7.33 (m, 2H), 7.30 – 7.26 (m, 4H), 7.08 – 7.01 (m, 4H), 6.90 (d, *J* = 1.8 Hz, 1H), 6.83 (d, *J* = 6.6 Hz, 2H), 6.75 (d, *J* = 7.2 Hz, 2H), 5.98 (d, *J* = 8.4 Hz, 1H), 5.26 (s, 1H), 4.39 (s, 1H), 2.83 (d, *J* = 16.8 Hz, 1H), 2.80 (s, 3H), 2.53 (d, *J* = 16.8 Hz, 1H), 2.47 (s, 3H) ppm; ^{13}C{^{1}H} NMR (150 MHz, Chloroform-*d*) δ 171.2, 168.9, 148.8, 139.1, 138.1, 135.0, 134.1, 130.7, 129.7, 129.0, 127.9, 127.8, 127.4, 127.2, 126.7, 126.6, 126.4, 126.1, 108.6, 108.3, 85.6, 68.8, 55.0, 38.7, 34.1, 26.6 ppm; IR (thin film): 3058, 2927, 1692, 1490, 1447, 1261, 1087, 803, 703 cm^{-1}; HRMS calc'd for $C_{32}H_{29}BrN_3O^+$: 550.1489, found: 550.1488 [M+H]$^+$.

3af(minor): colorless oil, R_f = 0.49 (hexanes:ethyl acetate = 1:1); ^{1}H NMR (600 MHz, Chloroform-*d*) δ 7.51 (d, *J* = 7.2 Hz, 2H), 7.37 – 7.31 (m, 4H), 7.26 (t, *J* = 7.8 Hz, 2H), 7.15 – 7.12 (m, 4H), 6.93 (s, 2H), 6.84 (s, 1H), 6.73 (d, *J* = 7.2 Hz, 2H), 6.15 (d, *J* = 8.4 Hz, 1H), 4.92 (s, 1H), 4.35 (s, 1H), 2.89 (d, *J* = 16.8 Hz, 1H), 2.72 (s, 3H), 2.69 (s, 3H), 2.52 (d, *J* = 16.8 Hz, 1H) ppm; ^{13}C{^{1}H} NMR (150 MHz,

Chloroform-*d*) δ 171.2, 167.8, 148.8, 138.6, 138.2, 135.2, 133.4, 130.6, 129.5, 127.8, 127.7, 127.41, 127.35, 127.13, 127.09, 126.9, 126.7, 108.9, 108.2, 86.5, 70.4, 55.0, 38.9, 34.6, 26.7 ppm (one resonance was not observed due to overlapping peaks).

8a-(((diphenylmethylene)amino)(phenyl)methyl)-5,6-dimethyl-5a,6,8,8a-tetrahydro-[1,3]dioxolo[4,5-*f*]pyrrolo[2,3-*b*]indol-7(5*H*)-one (3ag)

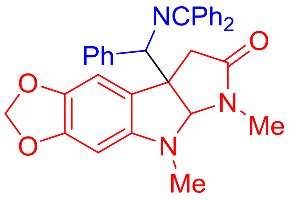

The reaction was performed following the **GP2** with *N*-benzyl-1,1-diphenylmethanimine **1a** (108.5 mg, 0.4 mmol) and *N*-methyl-2-(5-methyl-5*H*-[1,3]dioxolo[4,5-*f*]indol-7-yl)-*N*-(4-nitrophenoxy)acetamide **2g** (306.5 mg, 0.8 mmol). The crude product was separated by flash chromatography on deactivated silica gel (ethyl acetate:hexanes = 1:10). Further purification was purified on an Agilent HPLC 1260 system using acetonitrile:H_2O (80:20 vol./vol.) as mobile phase and flow rate of 3.0 mL/min at 254 nm to give the product **3ag** in 78% overall yield (**3ag**(major), 84.3 mg, 41% yield; **3ag**(minor), 76.6 mg, 37% yield, dr = 1.1:1).

3ag(major): colorless oil, R_f = 0.55 (hexanes:ethyl acetate = 1:1); ^{1}H NMR (600 MHz, Methanol-d_4) δ 7.52 (d, *J* = 7.2 Hz, 2H), 7.33 – 7.28 (m, 2H), 7.26 – 7.22 (m, 4H), 7.01 (s, 3H), 6.81 (s, 2H), 6.70 (d, *J* = 7.2 Hz, 2H), 6.33 (s, 1H), 5.85 (s, 1H), 5.70 (s, 1H), 5.65 (s, 1H), 5.26 (s, 1H), 4.43 (s, 1H), 2.78 (d, *J* = 17.4 Hz, 1H), 2.74 (s, 3H), 2.44 (d, *J* = 17.4 Hz, 1H), 2.31 (s, 3H) ppm; ^{13}C{^{1}H} NMR (150 MHz, Methanol-d_4) δ 173.5, 169.9, 148.6, 146.2, 140.8, 140.7, 139.3, 136.3, 130.1, 128.5, 128.3, 128.1, 127.9, 127.7, 127.4, 127.1, 127.0, 124.0, 104.5, 100.8, 92.4, 87.9, 69.8, 56.2, 40.1, 35.8, 26.3 ppm; IR (thin film): 3057, 1691, 1482, 1446, 1397, 1291, 1057, 737, 704 cm^{-1}; HRMS calc'd for $C_{33}H_{30}N_3O_3^+$: 516.2282, found: 516.2283 $[M+H]^+$.

3ag(minor): colorless oil, R_f = 0.47 (hexanes:ethyl acetate = 1:1); ^{1}H NMR (600 MHz, Methanol-d_4) δ 7.50 (d, *J* = 7.2 Hz, 2H), 7.35 – 7.28 (m, 4H), 7.24 (t, *J* = 7.8 Hz, 2H), 7.11 – 7.10 (m, 3H), 6.88 (dd, *J* = 6.6, 3.0 Hz, 2H), 6.74 (d, *J* = 6.6 Hz, 2H), 6.40 (s, 1H), 5.98 (s, 1H), 5.77 (s, 1H), 5.71 (s, 1H), 4.86 (s, 1H), 4.37 (s, 1H), 2.86 (d, *J* = 16.8 Hz, 1H), 2.65 (s, 3H), 2.48 (s, 3H), 2.44 (d, *J* = 16.8 Hz, 1H) ppm; ^{13}C{^{1}H} NMR (150 MHz, Methanol-d_4) δ 174.8, 170.4, 150.0, 147.7, 142.1, 141.7, 140.9, 138.0, 131.5,

129.9, 129.8, 129.6, 129.5, 129.2, 129.0, 128.82, 128.78, 124.8, 107.5, 102.2, 93.7, 90.3, 73.3, 57.5, 41.8, 37.5, 27.6 ppm.

6b-(((diphenylmethylene)amino)(phenyl)methyl)-9,10-dimethyl-6b,9,9a,10-tetrahydrobenzo[*g*]pyrrolo[2,3-*b*]indol-8(7*H*)-one (3ah)

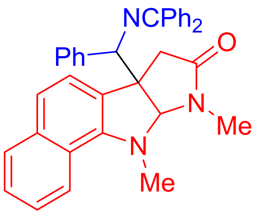

The reaction was performed following the **GP2** with *N*-benzyl-1,1-diphenylmethanimine **1a** (108.5 mg, 0.4 mmol) and *N*-methyl-2-(1-methyl-1*H*-benzo[*g*]indol-3-yl)-*N*-(4-nitrophenoxy)acetamide **2h** (311.3 mg, 0.8 mmol). The crude product was separated by flash chromatography on deactivated silica gel (ethyl acetate:hexanes = 1:10). Further purification was purified on an Agilent HPLC 1260 system using acetonitrile:H_2O (80:20 vol./vol.) as mobile phase and flow rate of 3.0 mL/min at 254 nm to give the product **3ah** in 72% overall yield (**3ah'**, 75.1 mg, 36% yield; **3ah"**, 75.1 mg, 36% yield, dr = 1:1).

3ah': white solid, m.p. = 189 – 190 ℃, R_f = 0.60 (hexanes:ethyl acetate = 1:1); ^{1}H NMR (600 MHz, Chloroform-*d*) δ 7.71 (t, *J* = 7.8 Hz, 2H), 7.62 (d, *J* = 7.2 Hz, 2H), 7.37 (t, *J* = 7.2 Hz, 1H), 7.32 – 7.28 (m, 5H), 7.27 – 7.23 (m, 3H), 7.01 (d, *J* = 8.4 Hz, 1H), 6.97 – 6.94 (m, 3H), 6.87 (d, *J* = 5.4 Hz, 2H), 6.74 (d, *J* = 7.2 Hz, 2H), 5.20 (s, 1H), 4.63 (s, 1H), 2.91 (d, *J* = 16.8 Hz, 1H), 2.87 (s, 3H), 2.55 (d, *J* = 16.8 Hz, 1H), 2.49 (s, 3H) ppm; ^{13}C{^{1}H} NMR (150 MHz, Chloroform-*d*) δ 171.1, 168.3, 146.7, 140.1, 138.2, 135.3, 134.1, 129.5, 129.3, 127.8, 127.7, 127.6, 127.3, 127.2, 126.9, 126.7, 126.3, 124.4, 123.8, 122.5, 122.1, 121.22, 121.18, 89.1, 68.6, 56.2, 40.3, 39.2, 25.9 ppm (one resonance was not observed due to overlapping peaks).

3ah": colorless oil, R_f = 0.53 (hexanes:ethyl acetate = 1:1); ^{1}H NMR (600 MHz, Chloroform-*d*) δ 7.77 (d, *J* = 8.4 Hz, 1H), 7.73 (d, *J* = 6.6 Hz, 1H), 7.61 (d, *J* = 7.2 Hz, 2H), 7.35 – 7.27 (m, 9H), 7.13 – 7.08 (m, 3H), 6.96 (d, *J* = 8.4 Hz, 1H), 6.90 (d, *J* = 7.2 Hz, 2H), 6.83 (d, *J* = 7.2 Hz, 2H), 4.66 (s, 1H), 4.46 (s, 1H), 3.21 (d, *J* = 16.8 Hz, 1H), 2.77 (s, 3H), 2.67 (s, 3H), 2.59 (d, *J* = 16.8 Hz, 1H) ppm; ^{13}C{^{1}H} NMR (150 MHz, Chloroform-*d*) δ 171.2, 167.5, 146.6, 139.4, 138.4, 135.5, 134.1, 129.4, 129.0, 127.8, 127.7, 127.3, 127.2, 127.1, 126.8, 126.7, 124.5, 123.9, 123.1, 122.6, 122.0, 121.1, 90.2, 71.4, 56.4, 40.8,

38.8, 25.7 ppm (two resonance was not observed due to overlapping peaks); IR (thin film): 3020, 2922, 1682, 1463, 1217, 1021, 772, 749 cm^{-1}; HRMS calc'd for $C_{36}H_{32}N_3O^+$: 522.2540, found: 522.2543 $[M+H]^+$.

4a-(((diphenylmethylene)amino)(phenyl)methyl)-1,9-dimethyl-1,3,4,4a,9,9a-hexahydro-2*H*-pyrido[2,3-*b*]indol-2-one (3ai)

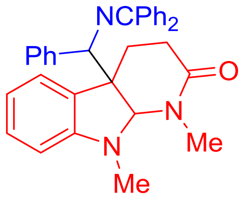

The reaction was performed following the **GP2** with *N*-benzyl-1,1-diphenylmethanimine **1a** (108.5 mg, 0.4 mmol) and *N*-methyl-3-(1-methyl-1*H*-indol-3-yl)-*N*-(4-nitrophenoxy)propanamide **2i** (282.5 mg, 0.8 mmol). The crude product was separated by flash chromatography on deactivated silica gel (ethyl acetate:hexanes = 1:10). Further purification was purified on an Agilent HPLC 1260 system using acetonitrile:H_2O (80:20 vol./vol.) as mobile phase and flow rate of 3.0 mL/min at 254 nm to give the product **3ai** in 52% overall yield (**3ai**(major), 55.1 mg, 28% yield; **3ai**(minor), 45.9 mg, 24% yield, dr = 1.2:1).

3ai(major): colorless oil, R_f = 0.55 (hexanes:ethyl acetate = 1:1); ^{1}H NMR (600 MHz, Methanol-d_4) δ 7.44 (t, *J* = 7.8 Hz, 1H), 7.41 – 7.38 (m, 4H), 7.35 (q, *J* = 7.8 Hz, 2H), 7.28 –7.26 (m, 4H), 7.14 – 7.13 (m, 2H), 7.09 (td, *J* = 7.8, 1.2 Hz, 1H), 6.80 (d, *J* = 7.2 Hz, 1H), 6.74 (d, *J* = 7.2 Hz, 2H), 6.69 (t, *J* = 7.2 Hz, 1H), 6.41 (d, *J* = 7.8 Hz, 1H), 5.43 (s, 1H), 4.54 (s, 1H), 2.95 (s, 3H), 2.83 (s, 3H), 2.12 – 2.09 (m, 1H), 2.03 – 1.95 (m, 3H) ppm; ^{13}C{^{1}H} NMR (150 MHz, Methanol-d_4) δ 175.4, 168.4, 152.2, 140.5, 139.8, 136.6, 130.2, 129.8, 128.6, 128.5, 128.1, 127.9, 127.67, 127.65, 127.6, 127.4, 127.3, 124.4, 117.8, 106.3, 85.0, 72.7, 55.5, 34.3, 34.0, 29.5, 29.1 ppm; IR (thin film): 3056, 2960, 1659, 1493, 1446, 1262, 1029, 735, 703 cm^{-1}; HRMS calc'd for $C_{33}H_{32}N_3O^+$: 486.2540, found: 486.2539 $[M+H]^+$.

3ai(minor): colorless oil, R_f = 0.51 (hexanes:ethyl acetate = 1:1); ^{1}H NMR (600 MHz, Methanol-d_4) δ 7.66 (d, *J* = 7.2 Hz, 2H), 7.43 (t, *J* = 7.2 Hz, 1H), 7.40 – 7.36 (m, 3H), 7.34 (t, *J* = 7.8 Hz, 2H), 7.12 – 7.09 (m, 3H), 7.04 (t, *J* = 7.8 Hz, 1H), 6.92 – 6.89 (m, 3H), 6.73 (d, *J* = 7.2 Hz, 2H), 6.65 (t, *J* = 7.2 Hz, 1H), 6.23 (d, *J* = 7.8 Hz, 1H), 5.29 (s, 1H), 4.44 (s, 1H), 3.05 (s, 3H), 2.53 (s, 3H), 2.25 – 2.18 (m, 2H), 2.12 – 2.02 (m, 2H) ppm; ^{13}C{^{1}H} NMR (150 MHz, Methanol-d_4) δ 174.5, 168.9, 151.9, 140.7, 139.4,

136.4, 130.1, 130.0, 128.7, 128.3, 128.2, 128.01, 128.00, 127.9, 127.3, 127.1, 127.0, 123.4, 117.6, 106.9, 84.3, 72.3, 55.4, 34.1, 33.6, 29.1, 28.0 ppm.

5a-(((diphenylmethylene)amino)(phenyl)methyl)-1,10-dimethyl-3,4,5,5a,10,10a-hexahydroazepino[2,3-*b*]indol-2(1*H*)-one (3aj)

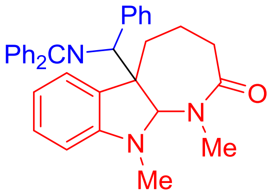

The reaction was performed following the **GP2** with *N*-benzyl-1,1-diphenylmethanimine **1a** (108.5 mg, 0.4 mmol) and *N*-methyl-4-(1-methyl-1*H*-indol-3-yl)-*N*-(4-nitrophenoxy)butanamide **2j** (293.7 mg, 0.8 mmol). The crude product was separated by flash chromatography on deactivated silica gel (ethyl acetate:hexanes = 1:10). Further purification was purified on an Agilent HPLC 1260 system using acetonitrile:H_2O (80:20 vol./vol.) as mobile phase and flow rate of 3.0 mL/min at 254 nm to give the product **3aj** in 46% overall yield (**3aj**(major), 48.2 mg, 24% yield; **3aj**(minor), 43.8 mg, 22% yield, dr = 1.1:1).

3aj(major): colorless oil, R_f = 0.60 (hexanes:ethyl acetate = 1:1); ^{1}H NMR (600 MHz, Methanol-*d*$_4$) δ 7.40 (t, *J* = 7.2 Hz, 1H), 7.34 – 7.29 (m, 10H), 7.23 (t, *J* = 7.8 Hz, 2H), 7.06 (t, *J* = 7.8 Hz, 1H), 6.63 (d, *J* = 7.7 Hz, 2H), 6.57 (d, *J* = 7.8 Hz, 2H), 6.48 (d, *J* = 7.8 Hz, 1H), 5.88 (s, 1H), 4.51 (s, 1H), 3.04 (s, 3H), 2.78 (s, 3H), 2.56 (q, *J* = 11.4 Hz, 1H), 2.01 (d, *J* = 13.2 Hz, 1H), 1.87 – 1.83 (m, 1H), 1.54 – 1.49 (m, 1H), 1.43 – 1.41 (m, 1H), 1.32 – 1.28 (m, 1H) ppm; ^{13}C{^{1}H} NMR (150 MHz, Methanol-*d*$_4$) δ 176.1, 168.0, 151.3, 140.7, 139.8, 136.5, 129.9, 129.7, 129.1, 128.6, 128.4, 128.2, 128.1, 127.8, 127.67, 127.65, 127.4, 123.8, 117.3, 105.0, 85.6, 74.7, 55.2, 35.8, 31.6, 31.2, 29.4, 18.8 ppm; IR (thin film): 3024, 2924, 1647, 1586, 1275, 1260, 1014, 795, 750 cm^{-1}; HRMS calc'd for $C_{34}H_{34}N_3O^+$: 500.2696, found: 500.2701 $[M+H]^+$.

3aj(minor): colorless oil, R_f = 0.47 (hexanes:ethyl acetate = 1:1); ^{1}H NMR (600 MHz, Chloroform-*d*) δ 7.61 (d, *J* = 7.8 Hz, 2H), 7.36 (t, *J* = 7.2 Hz, 1H), 7.31 – 7.29 (m, 3H), 7.24 (t, *J* = 7.8 Hz, 2H), 7.02 (t, *J* = 7.2 Hz, 1H), 6.98 – 6.93 (m, 3H), 6.74 – 6.71 (m, 5H), 6.53 (t, *J* = 7.2 Hz, 1H), 6.04 (d, *J* = 7.8 Hz, 1H), 5.29 (s, 1H), 4.31 (s, 1H), 3.25 (s, 3H), 2.34 (s, 3H), 2.28 – 2.23 (m, 1H), 1.93 – 1.87 (m, 2H), 1.78 – 1.74 (m, 1H), 1.64 – 1.58 (m, 1H), 1.36 – 1.30 (m, 1H) ppm; ^{13}C NMR (150 MHz, Chloroform-*d*) δ

174.0, 167.2, 150.4, 139.8, 138.6, 135.5, 129.3, 127.7, 127.6, 127.5, 127.4, 127.3, 127.20, 127.18, 126.6, 126.3, 126.0, 123.5, 115.9, 104.5, 84.6, 74.1, 54.2, 36.4, 31.2, 31.0, 28.4, 18.4 ppm.

3a-(((diphenylmethylene)amino)(phenyl)methyl)-1,8,8a-trimethyl-3,3a,8,8a-tetrahydropyrrolo[2,3-*b*]indol-2(1*H*)-one (3ak)

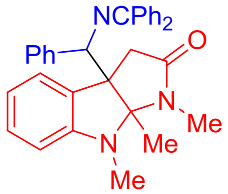

The reaction was performed following the **GP2** with *N*-benzyl-1,1-diphenylmethanimine **1a** (108.5 mg, 0.4 mmol) and 2-(1,2-dimethyl-1*H*-indol-3-yl)-*N*-methyl-*N*-(4-nitrophenoxy)acetamide **2k** (282.5 mg, 0.8 mmol). The crude product was separated by flash chromatography on deactivated silica gel (ethyl acetate:hexanes = 1:10). Further purification was purified on an Agilent HPLC 1260 system using acetonitrile:H_2O (80:20 vol./vol.) as mobile phase and flow rate of 3.0 mL/min at 254 nm to give the product **3ak** in 56% overall yield (**3ak'**, 54.4 mg, 28% yield; **3ak"**, 54.4 mg, 28% yield, dr = 1:1).

3ak': colorless oil, R_f = 0.49 (hexanes:ethyl acetate = 1:1); ^{1}H NMR (600 MHz, Chloroform-*d*) δ 7.60 (d, *J* = 7.2 Hz, 2H), 7.33 (t, *J* = 7.2 Hz, 1H), 7.28 (t, *J* = 7.2 Hz, 3H), 7.23 (t, *J* = 7.8 Hz, 2H), 7.07 (t, *J* = 7.2 Hz, 1H), 6.98 (t, *J* = 7.8 Hz, 3H), 6.70 (d, *J* = 7.2 Hz, 2H), 6.59 (d, *J* = 7.8 Hz, 2H), 6.40 (t, *J* = 7.2 Hz, 1H), 6.22 (d, *J* = 7.8 Hz, 1H), 6.12 (d, *J* = 7.2 Hz, 1H), 4.31 (s, 1H), 3.76 (d, *J* = 16.2 Hz, 1H), 2.74 (d, *J* = 16.2 Hz, 1H), 2.71 (s, 3H), 2.58 (s, 3H), 1.43 (s, 3H) ppm; ^{13}C{^{1}H} NMR (150 MHz, Chloroform-*d*) δ 171.3, 166.2, 147.9, 140.5, 138.6, 135.7, 129.8, 129.3, 128.3, 127.7, 127.5, 127.4, 127.2, 127.1, 126.5, 126.3, 126.1, 125.8, 116.5, 105.1, 89.0, 66.0, 57.6, 36.8, 28.6, 24.7, 16.1 ppm.

3ak": colorless oil, R_f = 0.38 (hexanes:ethyl acetate = 1:1); ^{1}H NMR (400 MHz, Chloroform-*d*) δ 7.36 (d, *J* = 8.4 Hz, 2H), 7.29 – 7.25 (m, 2H), 7.21 – 7.19 (m, 4H), 7.17 – 7.14 (m, 2H), 7.10 (t, *J* = 7.2 Hz, 2H), 6.83 (d, *J* = 8.4 Hz, 2H), 6.62 (d, *J* = 7.2 Hz, 2H), 6.54 (t, *J* = 7.2 Hz, 1H), 6.47 (d, *J* = 7.6 Hz, 1H), 6.29 (d, *J* = 7.2 Hz, 1H), 4.45 (s, 1H), 2.90 (s, 3H), 2.65 (d, *J* = 16.0 Hz, 1H), 2.55 (s, 3H), 2.34 (d, *J* = 16.0 Hz, 1H), 1.40 (s, 3H) ppm; ^{13}C{^{1}H} NMR (100MHz, Chloroform-*d*) δ 169.8, 166.2, 149.3, 140.4, 138.7, 135.1, 129.4, 129.0, 128.0, 127.9, 127.7, 127.6, 127.0, 126.9, 126.48, 126.45, 126.3, 125.0, 116.1, 104.9, 88.6, 68.6, 57.2, 38.6, 29.0, 24.2, 14.4 ppm; IR (thin film): 3057, 2910, 1688, 1493, 1418, 1394, 1314, 1298, 910, 705 cm^{-1}; HRMS calc'd for $C_{33}H_{32}N_3O^+$: 486.2540, found: 486.2539 [M+H]$^+$.

3a-(((diphenylmethylene)amino)(phenyl)methyl)-8a-ethyl-1,8-dimethyl-3,3a,8,8a-tetrahydropyrrolo[2,3-*b*]indol-2(1*H*)-one (3al)

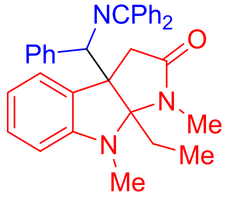

The reaction was performed following the **GP2** with *N*-benzyl-1,1-diphenylmethanimine **1a** (108.5 mg, 0.4 mmol) and 2-(2-ethyl-1-methyl-1*H*-indol-3-yl)-*N*-methyl-*N*-(4-nitrophenoxy)acetamide **2l** (293.7 mg, 0.8 mmol). The crude product was separated by flash chromatography on deactivated silica gel (ethyl acetate:hexanes = 1:10). Further purification was purified on an Agilent HPLC 1260 system using acetonitrile:H_2O (80:20 vol./vol.) as mobile phase and flow rate of 3.0 mL/min at 254 nm to give the product **3al** in 65% overall yield (**3al**(major), 80.0 mg, 40% yield; **3al**(minor), 50.0 mg, 25% yield, dr = 1.6:1).

3al(major): colorless oil, R_f = 0.51 (hexanes:ethyl acetate = 1:1); ^{1}H NMR (600 MHz, Chloroform-*d*) δ 7.33 (d, *J* = 7.2 Hz, 2H), 7.30 – 7.25 (m, 2H), 7.22 – 7.17(m, 5H), 7.16 – 7.13 (m, 3H), 6.89 (d, *J* = 7.2 Hz, 2H), 6.66 (s, 2H), 6.54 (t, *J* = 7.2 Hz, 1H), 6.49 (d, *J* = 7.8 Hz, 1H), 6.22 (d, *J* = 6.6 Hz, 1H), 4.63 (s, 1H), 2.94 (s, 3H), 2.64 (d, *J* = 16.2 Hz, 1H), 2.56 (s, 3H), 2.29 (d, *J* = 16.2 Hz, 1H), 2.16 (dq, *J* = 15.0, 7.8 Hz, 1H), 1.74 (dt, *J* = 15.0, 7.8 Hz, 1H), 0.62 (t, *J* = 7.2 Hz, 3H) ppm; $^{13}C\{^1H\}$ NMR (150 MHz, Chloroform-*d*) δ 170.7, 166.0, 149.6, 140.8, 138.8, 135.1, 129.9, 129.0, 128.14, 128.07, 127.77, 127.75, 127.6, 126.9, 126.7, 126.5, 126.4, 124.8, 116.0, 104.9, 91.0, 67.8, 57.1, 40.2, 29.2, 24.7, 20.7, 6.9 ppm; IR (thin film): 3056, 2967, 1682, 1490, 1463, 1422, 1265, 740, 705 cm^{-1}; HRMS calc'd for $C_{34}H_{34}N_3O^+$: 500.2696, found: 500.2692 $[M+H]^+$.

3al(minor): colorless oil, R_f = 0.44 (hexanes:ethyl acetate = 1:1); ^{1}H NMR (600 MHz, Chloroform-*d*) δ 7.63 (d, *J* = 7.8 Hz, 2H), 7.35 – 7.31 (m, 4H), 7.28 (t, *J* = 7.8 Hz, 2H), 7.12 (d, *J* = 7.2 Hz, 1H), 7.07 (t, *J* = 7.8 Hz, 1H), 6.99 (t, *J* = 7.2 Hz, 1H), 6.92 (t, *J* = 7.8 Hz, 2H), 6.88 (d, *J* = 6.6 Hz, 2H), 6.69 (t, *J* = 7.2 Hz, 1H), 6.52 (d, *J* = 7.8 Hz, 2H), 6.19 (d, *J* = 7.8 Hz, 1H), 4.62 (s, 1H), 2.84 (q, *J* = 16.8 Hz, 2H), 2.50 (s, 3H), 2.48 (s, 3H), 2.03 (dq, *J* = 15.0, 7.8 Hz, 1H), 1.87 (dq, *J* = 15.0, 7.2 Hz, 1H), 0.60 (t, *J* = 7.2 Hz, 3H) ppm; $^{13}C\{^1H\}$ NMR (150 MHz, Chloroform-*d*) δ 171.3, 166.0, 148.6, 139.5, 138.4, 135.8, 130.4, 129.2, 128.1, 127.8, 127.6, 127.5, 127.3, 127.1, 126.7, 126.2, 126.0, 125.5, 117.0, 105.9, 90.8, 67.2, 57.9, 39.8, 28.9, 24.5, 20.8, 6.7 ppm.

3a-(((9*H*-fluoren-9-ylidene)amino)(cyclohexyl)methyl)-8-benzyl-1-methyl-3,3a,8,8a-tetrahydropyrrolo[2,3-*b*]indol-2(1*H*)-one (3sm)

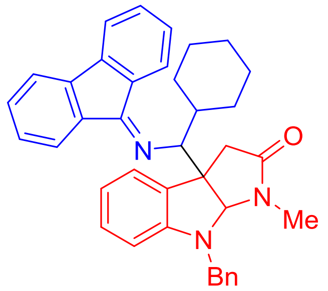

The reaction was performed following the **GP2** with (*E*)-1-cyclohexyl-*N*-(9*H*-fluoren-9-yl)methanimine **1s** (110.2 mg, 0.4 mmol) and 2-(1-benzyl-1*H*-indol-3-yl)-*N*-methyl-*N*-(4-nitrophenoxy)acetamide **2m** (332.4 mg, 0.8 mmol). The crude product was separated by flash chromatography on deactivated silica gel (ethyl acetate:hexanes = 1:8). Further purification was performed on an Agilent HPLC 1260 system using acetonitrile:H_2O (80:20 vol./vol.) as mobile phase and flow rate of 3.0 mL/min at 254 nm to give the product **3sm** in 67% overall yield (dr = 1.2:1, **3sm**(major), 80.6 mg, 37% yield; **3sm**(minor), 67.2 mg, 30% yield).

3sm(major): yellow oil, R_f = 0.66 (hexanes:ethyl acetate = 1:1); ^{1}H NMR (400 MHz, Chloroform-*d*) δ 7.87 (d, *J* = 7.6 Hz, 1H), 7.61 (t, *J* = 8.4 Hz, 2H), 7.53 (d, *J* = 7.2 Hz, 1H), 7.38 – 7.33 (m, 2H), 7.30 – 7.17 (m, 7H), 7.06 – 7.01 (m, 2H), 6.70 (td, *J* = 7.2, 0.8 Hz, 1H), 6.36 (d, *J* = 7.6 Hz, 1H), 5.47 (s, 1H), 4.76 (d, *J* = 4.8 Hz, 1H), 4.57 – 4.47 (m, 2H), 2.70 (s, 2H), 2.63 (s, 3H), 1.72 – 1.68 (m, 1H), 1.59 – 1.45 (m, 5H), 1.10 – 0.86 (m, 5H) ppm. ^{13}C NMR (100 MHz, Chloroform-*d*) δ 172.0, 161.3, 149.6, 143.5, 139.7, 137.8, 137.4, 132.0, 131.4, 130.3, 130.04, 127.99, 127.7, 127.3, 126.9, 126.3, 126.1, 125.9, 123.1, 121.7, 119.6, 118.3, 118.0, 107.5, 86.6, 69.0, 53.8, 53.3, 41.9, 40.5, 31.8, 27.8, 25.5, 25.0 ppm; IR (thin film): 3059, 2925, 1688, 1604, 1492, 1449, 1261, 793, 732 cm^{-1}, HRMS calc'd for $C_{38}H_{38}N_3O^+$: 552.3009, found: 552.3004 [M+H]$^+$.

3sm(minor): yellow oil, R_f = 0.65 (hexanes:ethyl acetate = 1:1); ^{1}H NMR (400 MHz, Chloroform-*d*) δ 7.92 (d, *J* = 7.6 Hz, 1H), 7.76 (d, *J* = 7.2 Hz, 1H), 7.62 (d, *J* = 7.6 Hz, 1H), 7.53 (d, *J* = 7.2 Hz, 1H), 7.35 (dt, *J* = 14.4, 7.6 Hz, 2H), 7.25 (dd, *J* = 15.2, 7.6 Hz, 2H), 7.19 – 7.15 (m, 5H), 7.04 (t, *J* = 7.6 Hz, 2H), 6.77 (td, *J* = 7.6, 0.8 Hz, 1H), 6.35 (d, *J* = 7.6 Hz, 1H), 4.73 (d, *J* = 2.4 Hz, 1H), 4.70 (s, 1H), 4.49 – 4.38 (m, 2H), 3.64 (d, *J* = 16.8 Hz, 1H), 2.75 (d, *J* = 16.8 Hz, 1H), 2.50 (s, 3H), 2.09 – 2.03 (m, 1H), 1.87 (d, *J* = 13.2 Hz, 1H), 1.70 (d, *J* = 13.2 Hz, 1H), 1.51 – 1.41 (m, 4H), 1.23 – 1.08 (m, 2H), 1.01 – 0.90 (m,

2H) ppm. ^{13}C NMR (100 MHz, Chloroform-*d*) δ 172.8, 160.7, 148.7, 143.6, 139.6, 137.5, 137.1, 131.7, 131.3, 130.3, 130.0, 128.1, 127.6, 127.4, 126.8, 126.3, 126.1, 124.9, 122.0, 119.5, 118.18, 118.16, 108.15, 87.8, 65.8, 54.4, 52.1, 40.4, 37.6, 31.1, 27.4, 26.0, 25.4 ppm (one resonance was not observed due to overlapping peaks).

3a-(((9*H*-fluoren-9-ylidene)amino)(cyclohexyl)methyl)-8-allyl-1-methyl-3,3a,8,8a-tetrahydropyrrolo[2,3-*b*]indol-2(1*H*)-one (3sn)

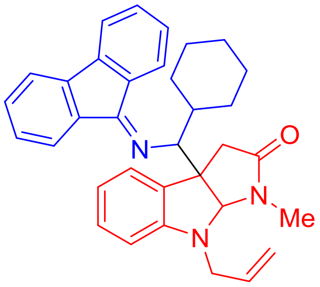

The reaction was performed following the **GP2** with (*E*)-1-cyclohexyl-*N*-(9*H*-fluoren-9-yl)methanimine **1s** (110.2 mg, 0.4 mmol) and 2-(1-allyl-1*H*-indol-3-yl)-*N*-methyl-*N*-(4-nitrophenoxy)acetamide **2n** (292.3 mg, 0.8 mmol). The crude product was separated by flash chromatography on deactivated silica gel (ethyl acetate:hexanes = 1:6). Further purification was performed on an Agilent HPLC 1260 system using acetonitrile:H_2O (80:20 vol./vol.) as mobile phase and flow rate of 3.0 mL/min at 254 nm to give the product **3sn** in 70% overall yield (dr = 1.3:1, **3sn**(major), 79.4 mg, 40% yield; **3sn**(minor), 61.1 mg, 30% yield).

3sn(major): yellow oil, R_f = 0.73 (hexanes:ethyl acetate = 1:1); ^{1}H NMR (400 MHz, Chloroform-*d*) δ 7.91 (d, *J* = 7.6 Hz, 1H), 7.61 (dd, *J* = 10.0, 7.6 Hz, 2H), 7.52 (d, *J* = 7.6 Hz, 1H), 7.39 – 7.33 (m, 2H), 7.24 – 7.20 (m, 2H), 7.10 – 7.03 (m, 2H), 6.71 (td, *J* = 7.2, 1.2 Hz, 1H), 6.49 (d, *J* = 7.6 Hz, 1H), 5.93 – 5.83 (m, 1H), 5.41 (s, 1H), 5.25 – 5.14 (m, 2H), 4.75 (d, *J* = 4.4 Hz, 1H), 3.94 – 3.92 (m, 2H), 2.82 (s, 3H), 2.64 (s, 2H), 1.67 – 1.58 (m, 1H), 1.51 – 1.41 (m, 5H), 1.07 – 0.86 (m, 5H) ppm. ^{13}C NMR (100 MHz, Chloroform-*d*) δ 172.8, 162.3, 150.4, 144.5, 140.7, 138.4, 134.7, 133.0, 132.4, 131.4, 131.1, 129.0, 128.3, 128.0, 127.1, 124.0, 122.8, 120.6, 119.3, 118.9, 117.0, 108.6, 86.9, 70.0, 54.8, 52.9, 42.8, 41.4, 33.0, 29.0, 26.6, 26.4 ppm; IR (thin film): 3059, 2922, 1687, 1605, 1491, 1449, 1261, 913, 747 cm^{-1}, HRMS calc'd for $C_{34}H_{36}N_3O^+$: 502.2853, found: 502.2850 $[M+H]^+$.

3sn(minor): yellow oil, R_f = 0.72 (hexanes:ethyl acetate = 1:1); ^{1}H NMR (400 MHz, Chloroform-*d*) δ 7.89 (d, *J* = 7.6 Hz, 1H), 7.76 (d, *J* = 7.2 Hz, 1H), 7.62 (d, *J* = 7.6 Hz, 1H), 7.54 (d, *J* = 7.2 Hz, 1H), 7.40

– 7.33 (m, 2H), 7.27 (td, J = 7.2, 1.2 Hz, 1H), 7.21 (d, J = 1.2 Hz, 1H), 7.15 – 7.08 (m, 2H), 6.76 (td, J = 7.2, 1.2 Hz, 1H), 6.49 (d, J = 7.6 Hz, 1H), 5.81 – 5.71 (m, 1H), 5.11 – 5.03 (m, 2H), 4.62 (d, J = 2.4 Hz, 1H), 4.58 (s, 1H), 3.91 – 3.79 (m, 2H), 3.63 (d, J = 16.8 Hz, 1H), 2.71 (d, J = 16.8 Hz, 1H), 2.63 (s, 3H), 2.03 – 1.97 (m, 1H), 1.87 (d, J = 13.2 Hz, 1H), 1.70 (d, J = 13.2 Hz, 1H), 1.49 – 1.46 (m, 3H), 1.37 (d, J = 12.8 Hz, 1H), 1.19 – 1.04 (m 2H), 0.97 – 0.87 (m, 2H) ppm. ^{13}C NMR (100 MHz, Chloroform-*d*) δ 172.6, 160.8, 148.3, 143.6, 139.6, 137.5, 133.4, 131.6, 131.3, 130.3, 130.0, 128.1, 127.4, 126.7, 126.4, 124.9, 122.1, 119.5, 118.1, 117.9, 116.5, 107.9, 86.8, 65.7, 54.4, 50.7, 40.1, 37.4, 31.0, 26.9, 25.9, 25.4 ppm.

***tert*-butyl 3a-(((9*H*-fluoren-9-ylidene)amino)(cyclohexyl)methyl)-8-methyl-2-oxo-3,3a,8,8a-tetrahydropyrrolo[2,3-*b*]indole-1(2*H*)-carboxylate (3so)**

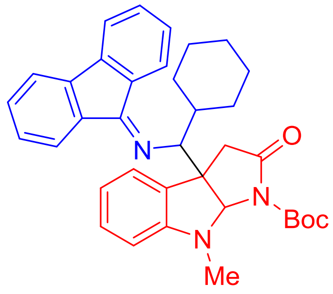

The reaction was performed following the **GP2** with (*E*)-1-cyclohexyl-*N*-(9*H*-fluoren-9-yl)methanimine **1s** (110.2 mg, 0.4 mmol) and *tert*-butyl (2-(1-methyl-1*H*-indol-3-yl)acetyl)(4-nitrophenoxy)carbamate **2o** (340.4 mg, 0.8 mmol). The crude product was separated by flash chromatography on deactivated silica gel (ethyl acetate:hexanes = 1:7). Further purification was performed on an Agilent HPLC 1260 system using acetonitrile:H_2O (80:20 vol./vol.) as mobile phase and flow rate of 3.0 mL/min at 254 nm to give the product **3so** in 56% overall yield (dr = 1.3:1, **3so**(major), 71.1 mg, 32% yield; **3so**(minor), 54.7 mg, 24% yield).

3so(major): yellow oil, R_f = 0.53 (hexanes:ethyl acetate = 3:1); ^{1}H NMR (400 MHz, Chloroform-*d*) δ 8.08 (d, J = 7.6 Hz, 1H), 7.74 – 7.72 (m, 2H), 7.56 – 7.53 (m, 2H), 7.50 (d, J = 7.2 Hz, 1H), 7.36 – 7.30 (m, 2H), 7.27 – 7.21 (m, 2H), 7.17 – 7.15 (m, 1H), 7.04 (ddd, J = 8.0, 7.2, 1.2 Hz, 1H), 5.26 (dd, J = 7.6, 3.6 Hz, 1H), 4.53 (s, 1H), 3.79 (s, 3H), 1.82 – 1.71 (m, 2H), 1.62 – 1.48 (m, 7H), 1.14 (s, 9H), 1.07 – 0.98 (m, 4H) ppm. ^{13}C NMR (100 MHz, Chloroform-*d*) δ 171.1, 161.5, 148.1, 143.0, 139.9, 137.6, 135.5, 131.3, 130.1, 129.8, 128.7, 127.4, 127.2, 127.1, 126.9, 121.5, 120.8, 119.1, 118.4, 118.2, 118.1, 108.2,

106.7, 80.7, 66.4, 47.4, 40.1, 32.1, 29.2, 28.5, 26.6, 25.2, 25.2 ppm; IR (thin film): 3059, 2925, 1644, 1449, 1369, 1261, 1144, 794, 743 cm^{-1}, HRMS calc'd for $C_{36}H_{40}N_3O_3^+$: 562.3064, found: 562.3066 $[M+H]^+$.

3so(minor): yellow oil, R_f = 0.47 (hexanes:ethyl acetate = 3:1); ^{1}H NMR (600 MHz, Chloroform-*d*) δ 7.93 (d, *J* = 7.8 Hz, 1H), 7.66 (d, *J* = 7.2 Hz, 1H), 7.50 (dd, *J* = 7.8, 4.2 Hz, 2H), 7.40 – 7.38 (m, 1H), 7.23 (q, *J* = 7.8 Hz, 2H), 7.09 – 7.07 (m, 1H), 7.04 (t, *J* = 7.8 Hz, 2H), 6.89 (t, *J* = 7.8 Hz, 1H), 6.62 (s, 1H), 4.76 (d, *J* = 7.8 Hz, 1H), 4.59 (s, 1H), 3.38 (s, 3H), 2.18 (d, *J* = 6.6 Hz, 1H), 1.93 (d, *J* = 7.8 Hz, 1H), 1.75 (d, *J* = 10.8 Hz, 1H), 1.70 (d, *J* = 12.6 Hz, 1H), 1.60 (t, *J* = 12.6 Hz, 2H), 1.55 (s, 9H), 1.52 – 1.48 (m, 2H), 1.30 –1.24 (m, 2H), 1.15 – 1.09 (m, 3H) ppm. ^{13}C NMR (150 MHz, Chloroform-*d*) δ 170.7, 162.6, 149.0, 143.0, 140.0, 136.8, 135.5, 130.6, 130.5, 127.3, 126.9, 126.5, 126.4, 125.5, 121.8, 120.8, 119.2, 118.4, 118.3, 117.7, 110.1, 108.1, 80.6, 66.4, 46.9, 41.7, 31.6, 29.6, 28.9, 27.2, 25.2 ppm (one resonance was not observed due to overlapping peaks).

Gram scale synthesis of 3aa

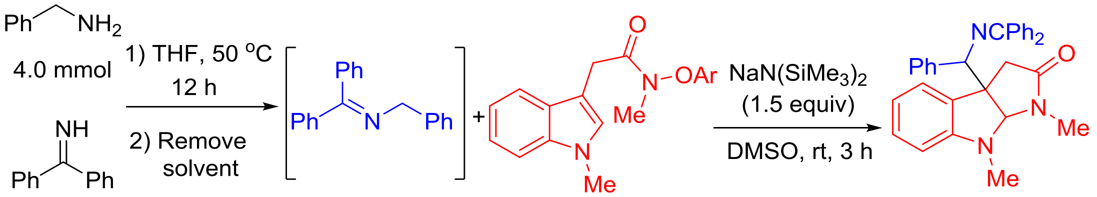

4.0 mmol **1a** 4.0 mmol **2a** 8.0 mmol **3aa** 74%,1.40 g

An oven-dried 200 mL Schlenk tube equipped with a stir bar was sealed with a rubber septum and degassed by nitrogen purge (repeated three times). Tetrahydrofuran (10 mL) was added under nitrogen via syringe through the rubber septum. Benzophenone imine (724.9 mg, 4.0 mmol) and benzyl amine (428.6 mg, 4.0 mmol) were added under nitrogen via syringe through the rubber septum at room temperature. The reaction was heated and stirred at 50 □ for 12 h, cooled to room temperature, the solvent was removed in vacuo and the Schlenk tube was filled with nitrogen. A solution (prepared in the glove box) of *N*-methyl-2-(1-methyl-1*H*-indol-3-yl)-*N*- (4-nitrophenoxy)acetamide **2a** (2.70 g, 8.0 mmol) in 20 mL anhydrous DMSO was added to the Schlenk tube via syringe through the rubber septum. Next, a solution of $NaN(SiMe_3)_2$ (11.0 g, 6.0 mmol) in 60 mL anhydrous DMSO was added by syringe through the rubber septum at room temperature. Upon addition of the base, the reaction turned purple. The reaction mixture was then heated and stirred for 3 h in total at room temperature. The reaction mixture

was opened to air, quenched with 5 ml of H_2O. The mixture was diluted with H_2O and the layers were separated. The aqueous layer was extracted with ethyl acetate (3 X 50 mL) and the combined organic layers were washed with brine, dried ($MgSO_4$), filtered and evaporated. The crude material was loaded onto a deactivated silica gel column via pipette and purified by flash chromatography (ethyl acetate:hexanes = 1:10) to give the product 3aa in 74% overall yield.(dr = 1.2:1, **3aa** (major), 0.74 g, 40% yield; **3aa** (minor), 0.63g, 34% yield).

Imine product hydrolysis

Hydrolysis of product 3aa (major): An oven-dried 10 mL microwave vial equipped with a stir bar was charged with **3aa** (47.2 mg, 0.1 mmol). Next, 1 N HCl (1 mL) and MeOH (1 mL) were added to the reaction vial via syringe at 0 °C. The solution was warmed to room temperature, stirred at room temperature and was monitored by TLC until all **3aa** was consumed (reaction completed in 1 h). The reaction mixture was transferred to a 10 mL separatory funnel via pipette and was extracted with dichloromethane (3 X 2 mL). The aqueous layer was then basified with 1N NaOH until the pH=10 and was extracted with dichloromethane (3 X 2 mL). The combined organic layers were concentrated in vacuo, loaded onto a deactivated silica gel column via pipette and purified by flash chromatography on deactivated silica gel (ethyl acetate to ethyl acetate:methanol = 5:1) to give the amine product **4aa** (28.2 mg, 92% yield) was obtained as a white solid.

3a-(amino(phenyl)methyl)-1,8-dimethyl-3,3a,8,8a-tetrahydropyrrolo[2,3-*b*]indol-2(1*H*)-one (4aa)

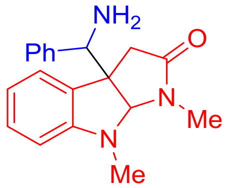

m.p. = 147 – 148 °C, R_f = 0.33 (ethyl acetate:methanol = 4:1); ^{1}H NMR (600 MHz, Methanol-d_4) δ 7.09 – 7.07 (m, 3H), 6.99 (t, *J* = 7.8 Hz, 1H), 6.94 (t, *J* = 3.6 Hz, 2H), 6.87 (d, *J* = 7.2 Hz, 1H), 6.62 (t, *J* = 7.2 Hz, 1H), 6.21 (d, *J* = 7.8 Hz, 1H), 5.08 (s, 1H), 4.22 (s, 1H), 3.09 (d, *J* = 16.8 Hz, 1H), 2.73 (s, 3H), 2.61 (s, 3H), 2.47 (d, *J* = 16.8 Hz, 1H) ppm (amino protons were not observed); ^{13}C{^{1}H} NMR (150 MHz, Methanol-d_4) δ 173.2, 150.2, 139.1, 131.2, 129.3, 127.38, 127.35, 127.3, 124.4, 118.1, 107.9, 86.5, 59.3, 55.2, 39.6, 33.7, 26.7 ppm; IR (thin film): 3027, 1683, 1494, 1452, 1399, 1219, 988, 772 cm^{-1}; HRMS

calc'd for $C_{19}H_{22}N_3O^+$: 308.1757, found: 308.1758 $[M+H]^+$.

Mechanistic study

a. Trapping with TEMPO

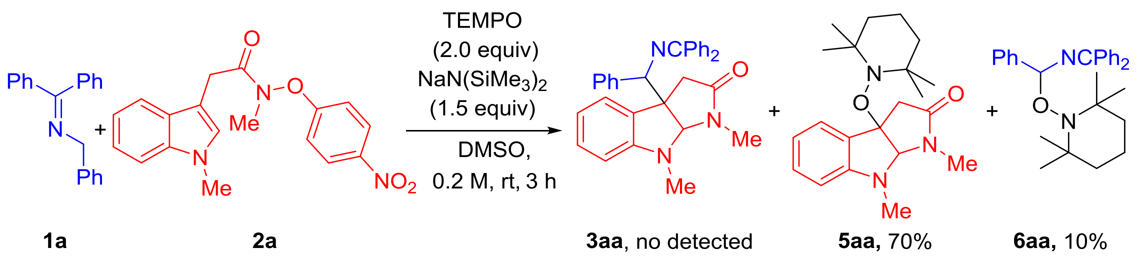

The reaction was performed following the **GP2** with *N*-benzyl-1,1-diphenylmethanimine **1a** (81.4 mg, 0.3 mmol), *N*-methyl-2-(1-methyl-1*H*-indol-3-yl)-*N*-(4-nitrophenoxy)acetamide **2a** (203.6 mg, 0.6 mmol), 2,2,6,6-tetramethylpiperidine-1-oxyl (93.7mg, 0.6 mmol), $NaN(SiMe_3)_2$ (82.4 mg, 0.45 mmol) and 6.0 mL dry DMSO and stirred for 3 h at room temperature. The crude product was separated by flash chromatography on deactivated silica gel (petroleum ether: ethyl acetate = 10:1 to 5:1). Further purification was performed on an Agilent HPLC 1260 system using acetonitrile:H_2O (75:25 vol./vol.) as mobile phase and flow rate of 3.0 mL/min at 254 nm to give the product **5aa** (75.1 mg, 70%) and **6aa** (12.8 mg, 10%).

1,8-dimethyl-3a-((2,2,6,6-tetramethylpiperidin-1-yl)oxy)-3,3a,8,8a-tetrahydropyrrolo[2,3-*b*]indol-2(1*H*)-one (5aa)

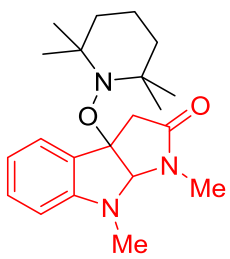

The ^1H and $^{13}C\{^1H\}$ data for this compound match the literature data.[3]

1,1-diphenyl-*N*-(phenyl((2,2,6,6-tetramethylpiperidin-1-yl)oxy)methyl)methanimine (6aa)

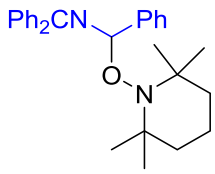

The ^{1}H and $^{13}C\{^{1}H\}$ data for this compound match the literature data.[5]

b. Reaction in the absence of ketimine

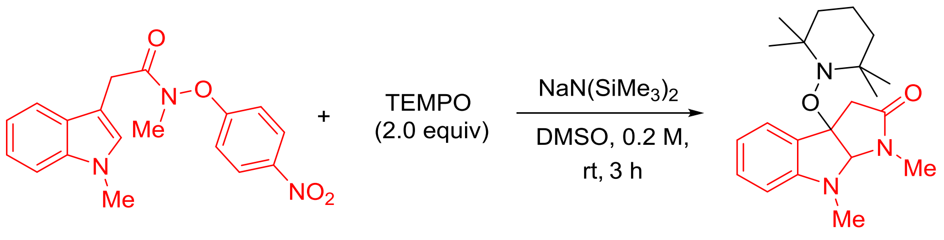

The reaction was performed following the **GP2** with *N*-methyl-2-(1-methyl-1*H*-indol-3-yl)-*N*-(4-nitrophenoxy)acetamide **2a** (33.9 mg, 0.1 mmol), 2,2,6,6-tetramethylpiperidine-1-oxyl (31.3 mg, 0.2 mmol), $NaN(SiMe_3)_2$ (55.0 mg, 0.3 mmol) and 0.5 mL dry DMSO and stirred for 3 h at room temperature. The yield of radical coupling product **5aa** was less than 5%.

X-ray crystal structures of compound 3ga’

Sample preparation: To a 10 mL vial containing **3ga’** (30 mg) was added a 10:1 mixture of acetonitrile and hexanes (about 4 mL). The single crystal **3ga’** was obtained by slowly evaporating mixed solvent at room temperature under the air conditions.
CCDC 2293492 contains the supplementary crystallographic data for compound **3ga’**. The data can be obtained free of charge from The Cambridge Crystallographic Data Center via www.ccdc.cam.ac.uk/data_request/cif.

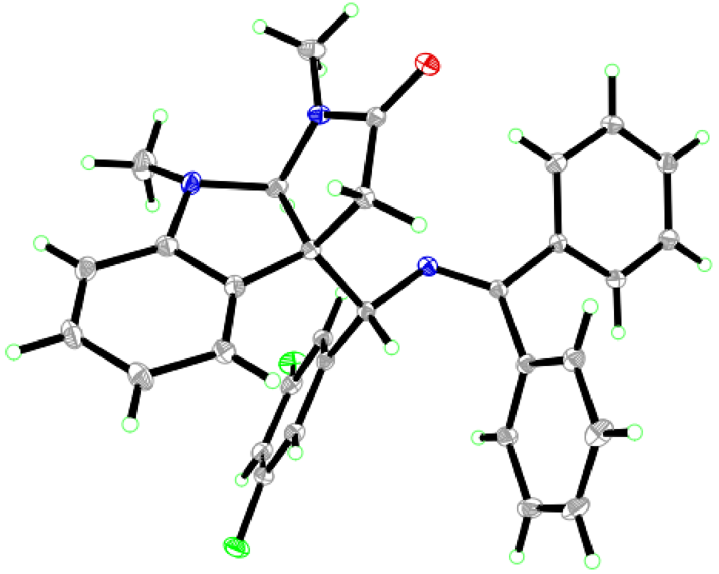

X-ray crystal structures of compound 3ga”

Sample preparation: To a 10 mL vial containing **3ga”** (30 mg) was added a 10:1 mixture of acetonitrile

and hexanes (about 4 mL). The single crystal **3ga”** was obtained by slowly evaporating mixed solvent at room temperature under the air conditions.

CCDC 2293493 contains the supplementary crystallographic data for compound **3ga”**. The data can be obtained free of charge from The Cambridge Crystallographic Data Center via www.ccdc.cam.ac.uk/data_request/cif.

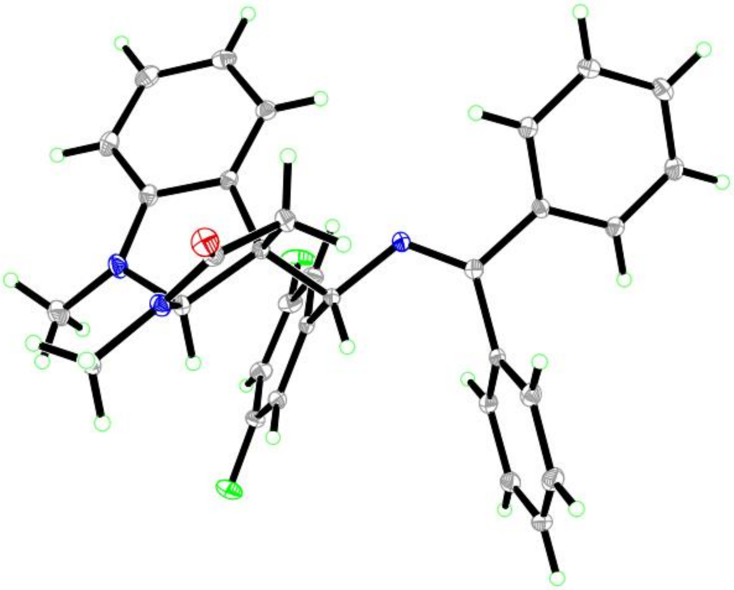

Supplementary references

(1) M. J. Odonnell, W. D. Bennett, W. A. Bruder, W. N. Jacobsen, K. Knuth, B. Leclef, R. L. Polt, F. G. Bordwell, S. R. Mrozack and T. A. Cripe, *J. Am. Chem. Soc.*, **1988**, 110, 8520-8525.

(2) Y. Zhu and S. L. Buchwald, *J. Am. Chem. Soc.*, **2014**, 136, 4500-4503.

(3) a. K. Wu, Y. L. Du and T. Wang, *Org. Lett.*, **2017**, 19, 5669-5672; b. K. Wu, Y. L. Du, Z. Wei and T. Wang, *Chem. Commun.*, **2018**, 54, 7443-7446.

(4) Han, Y., Corey, E. J., *Org. Lett.* **2019,** *21* (1), 283–286

(5) K. L. Yu, M. Y. Li, G. G. Deng, C. X. Liu, J. Wang, Z. F. Liu, H. B. Zhang, X. D. Yang and P. J. Walsh, *Adv. Synth. Catal.*, **2019**, 361, 4354-4359.

NMR Spectra

Figure S1. ^{1}H NMR spectra (600 MHz, Chloroform-*d*) of 3a-(((diphenylmethylene)amino)(phenyl)methyl)-1,8-dimethyl-3,3a,8,8a-tetrahydropyrrolo[2,3-*b*]indol-2(1*H*)-one (3aa(major))

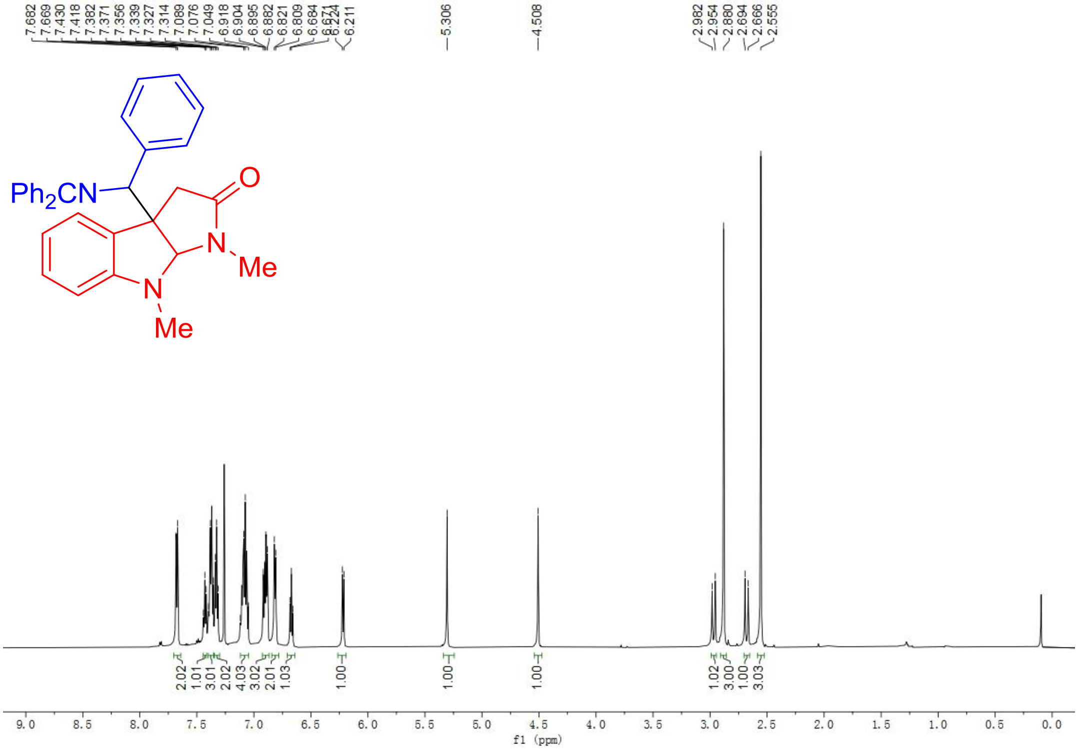

Figure S2. ^{13}C{^{1}H} NMR spectra (150 MHz, Chloroform-*d*) of 3a-(((diphenylmethylene)amino)(phenyl)methyl)-1,8-dimethyl-3,3a,8,8a-tetrahydropyrrolo[2,3-*b*]indol-2(1*H*)-one (3aa(major))

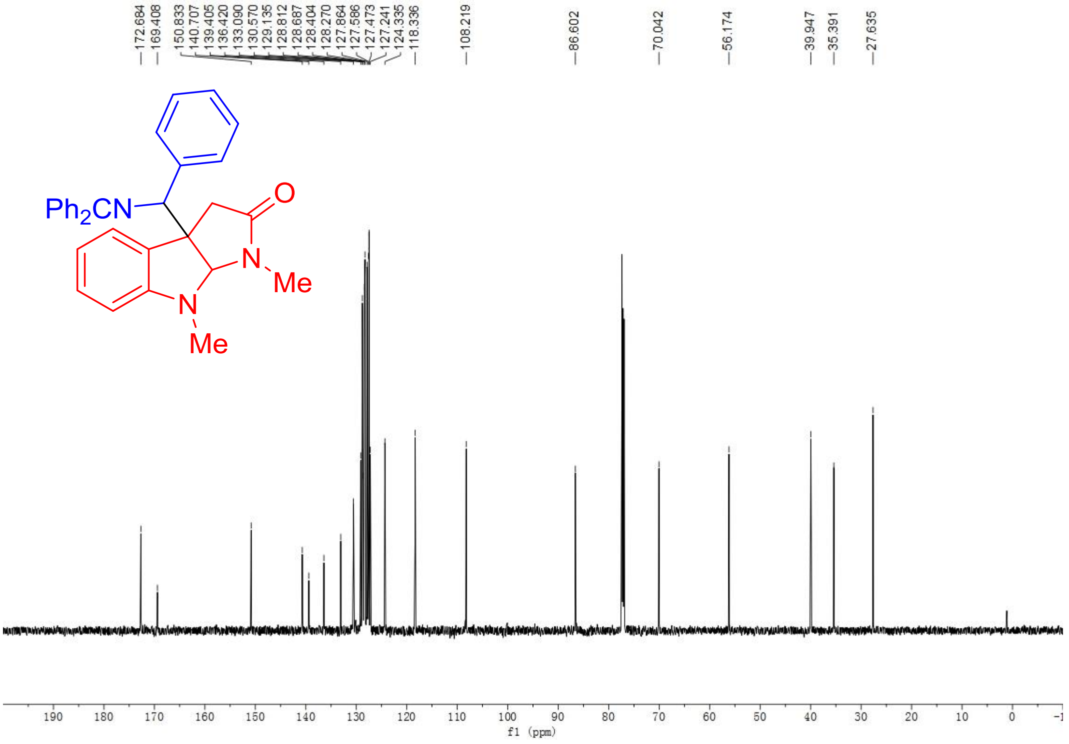

Figure S3. ^{1}H NMR spectra (600 MHz, Chloroform-*d*) of 3a-(((diphenylmethylene)amino)(phenyl)methyl)-1,8-dimethyl-3,3a,8,8a-tetrahydropyrrolo[2,3-*b*]indol-2(1*H*)-one (3aa((minor)))

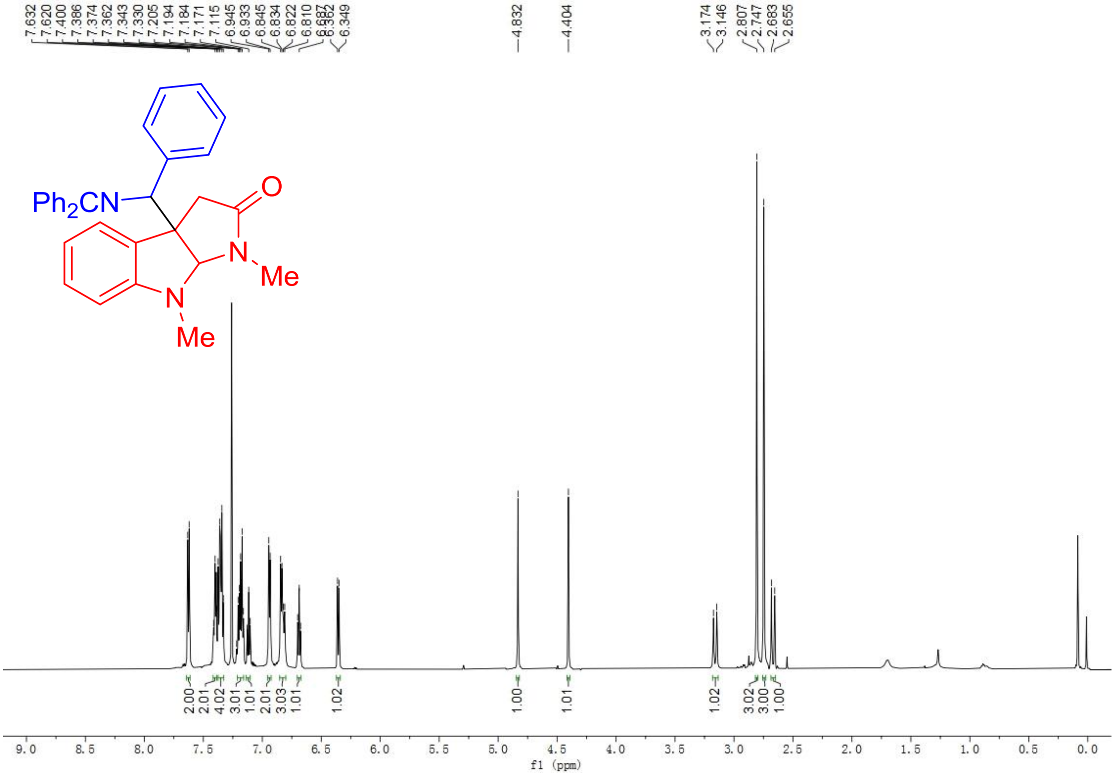

Figure S4. ^{13}C{^{1}H} NMR spectra (150 MHz, Chloroform-*d*) of 3a-(((diphenylmethylene)amino)(phenyl)methyl)-1,8-dimethyl-3,3a,8,8a-tetrahydropyrrolo[2,3-*b*]indol-2(1*H*)-one (3aa((minor)))

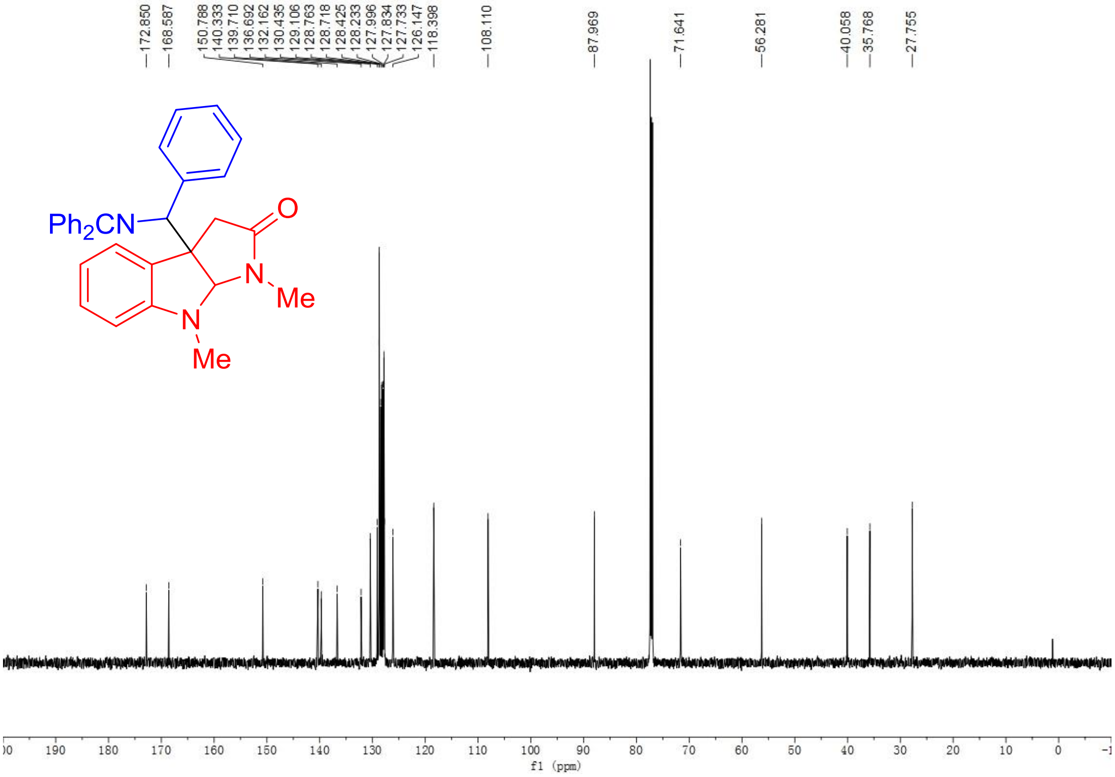

Figure S5. **^{1}H NMR spectra (600 MHz, Methanol-d_4) of 3a-(((diphenylmethylene)amino)(4-methoxyphenyl)methyl)-1,8-dimethyl-3,3a,8,8a-tetrahydropyrrolo[2,3-*b*]indol-2(1*H*)-one (3ba(major))**

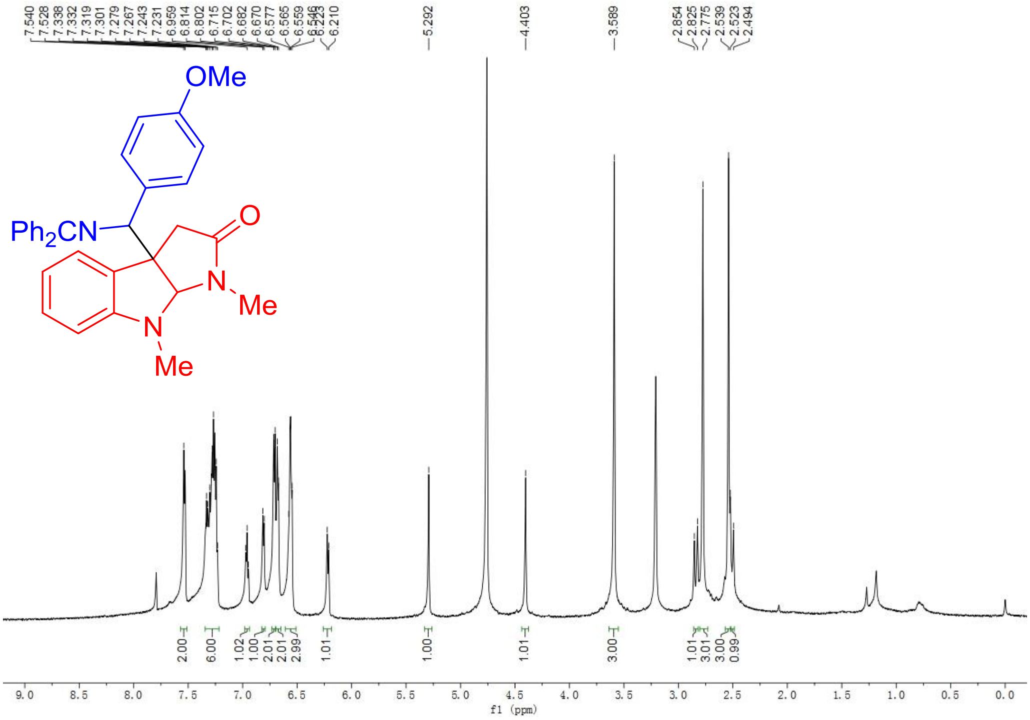

Figure S6. **^{13}C{^{1}H} NMR spectra (150 MHz, Methanol-d_4) of 3a-(((diphenylmethylene)amino)(4-methoxyphenyl)methyl)-1,8-dimethyl-3,3a,8,8a-tetrahydropyrrolo[2,3-*b*]indol-2(1*H*)-one (3ba(major))**

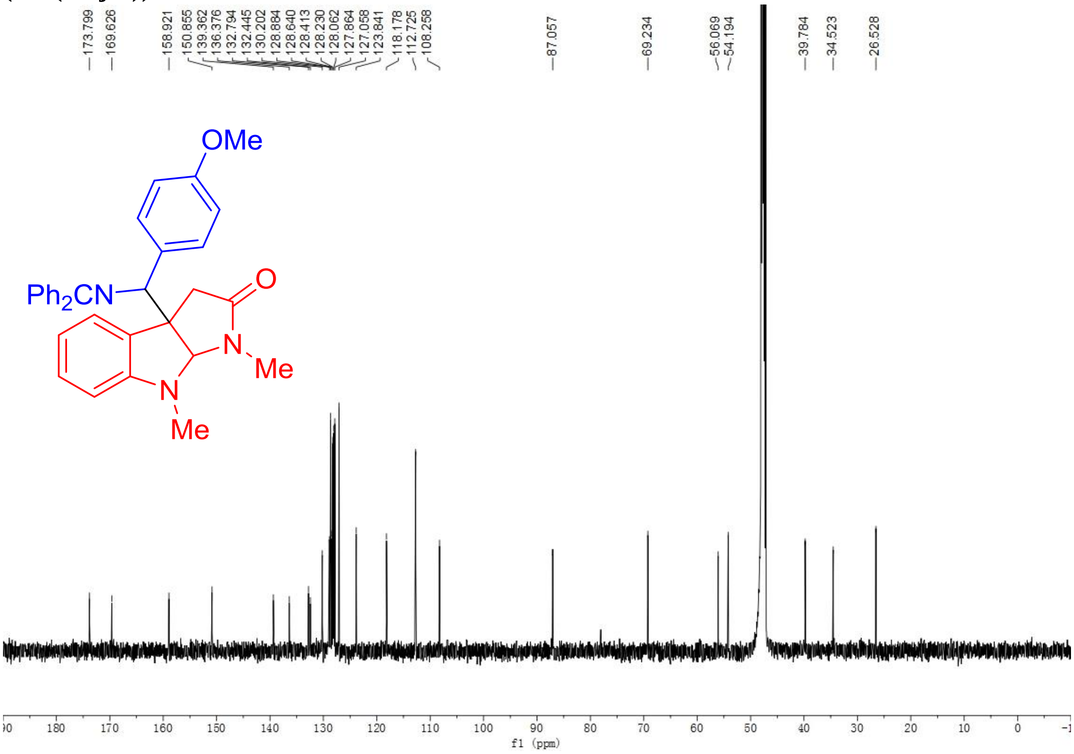

Figure S7. ^{1}H NMR spectra (600 MHz, Methanol-d_4) of 3a-(((diphenylmethylene)amino)(4-methoxyphenyl)methyl)-1,8-dimethyl-3,3a,8,8a-tetrahydropyrrolo[2,3-*b*]indol-2(1*H*)-one (3ba(minor))

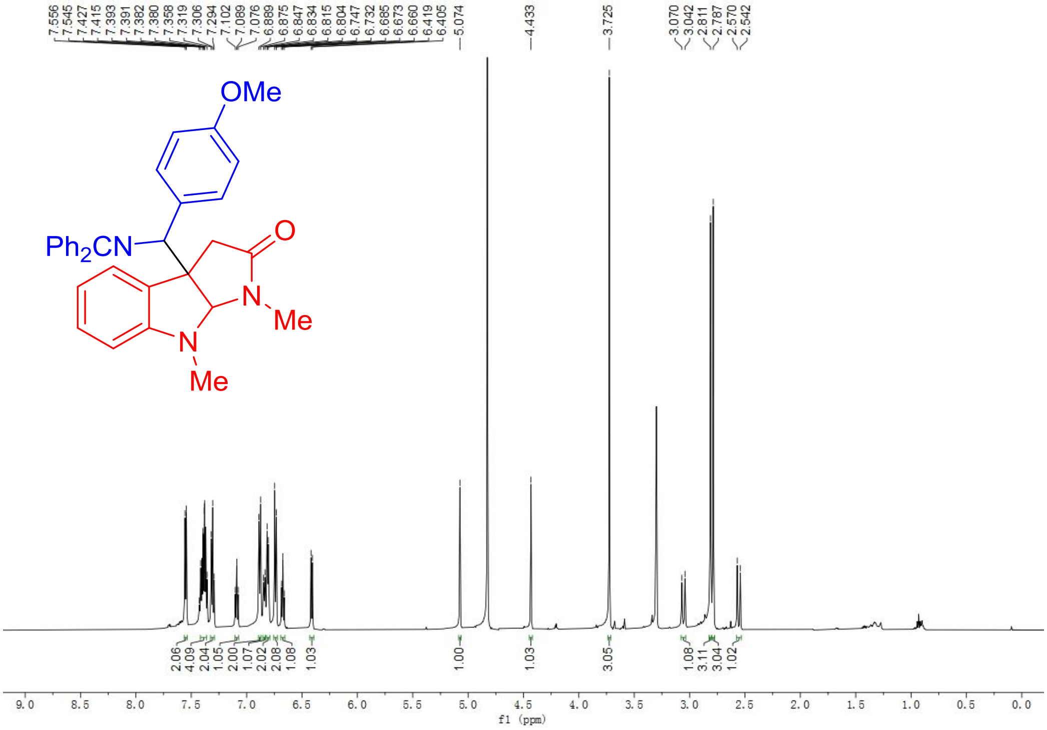

Figure S8. ^{13}C{^{1}H} NMR spectra (150 MHz, Methanol-d_4) of 3a-(((diphenylmethylene)amino)(4-methoxyphenyl)methyl)-1,8-dimethyl-3,3a,8,8a-tetrahydropyrrolo[2,3-*b*]indol-2(1*H*)-one (3ba(minor))

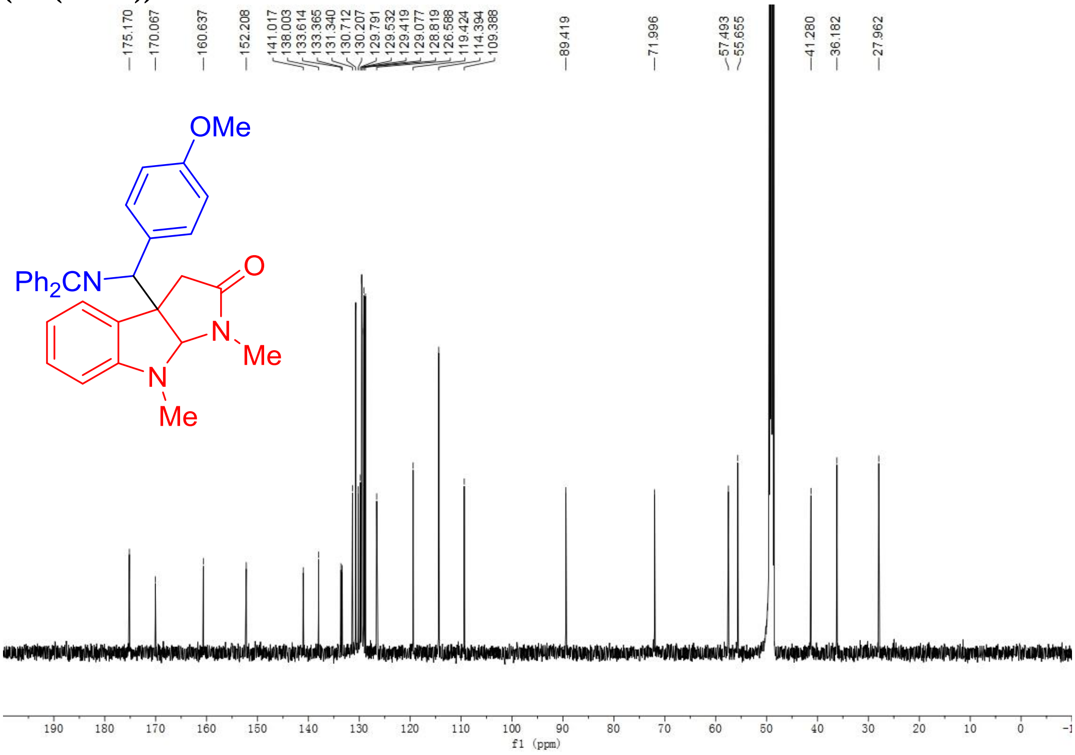

Figure S9. ^{1}**H NMR spectra (600 MHz, Chloroform-*d*) of 3a-(benzo[*d*][1,3]dioxol-5-yl((diphenylmethylene)amino)methyl)-1,8-dimethyl-3,3a,8,8a-tetrahydropyrrolo[2,3-*b*]indol-2(1*H*)-one (3ca')**

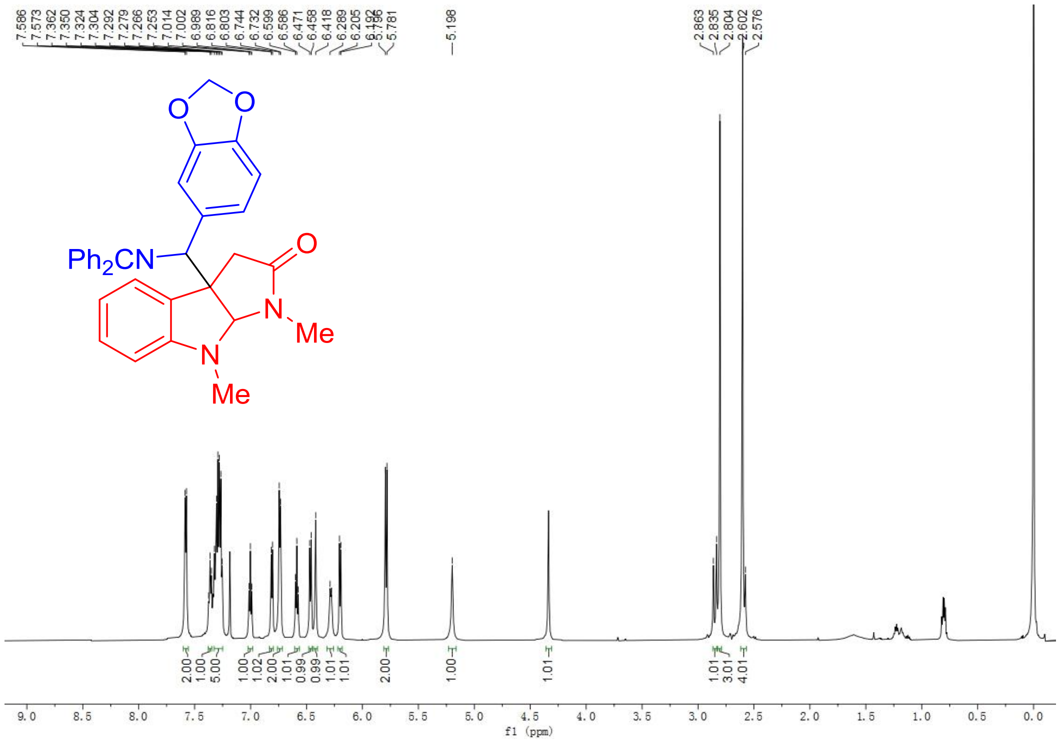

Figure S10. ^{13}**C{^{1}H} NMR spectra (150 MHz, Chloroform-*d*) of 3a-(benzo[*d*][1,3]dioxol-5-yl((diphenylmethylene)amino)methyl)-1,8-dimethyl-3,3a,8,8a-tetrahydropyrrolo[2,3-*b*]indol-2(1*H*)-one (3ca')**

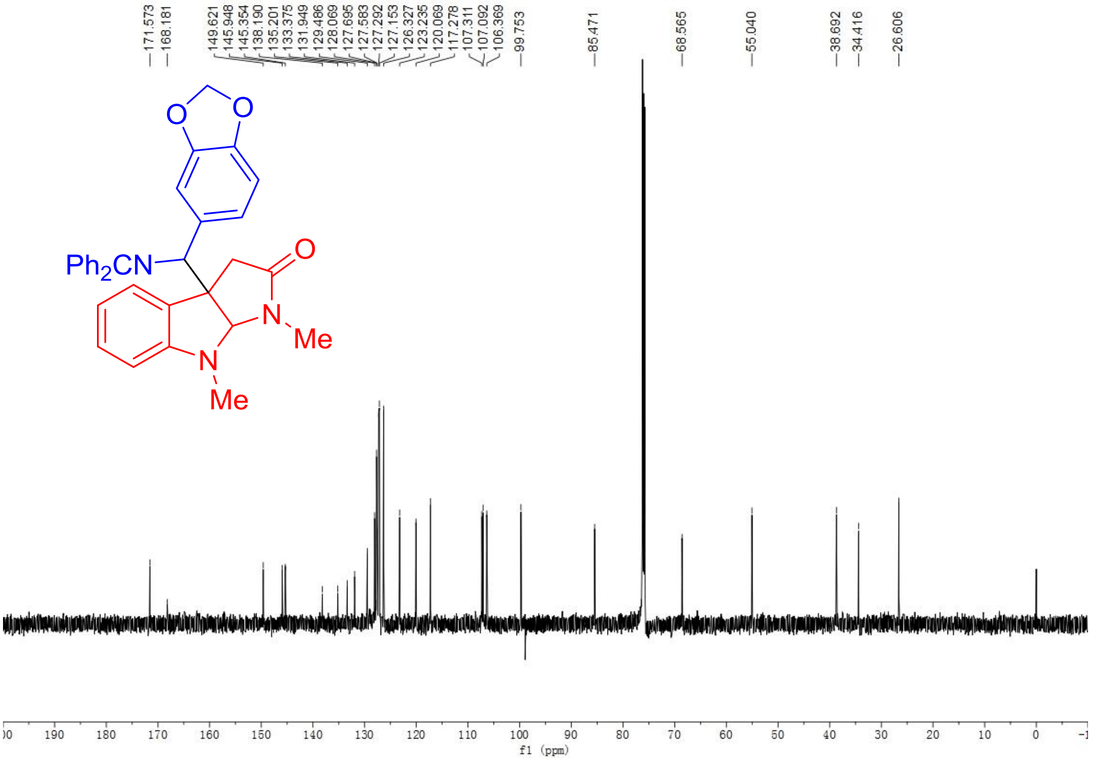

Figure S11. ^{1}H **NMR spectra (600 MHz, Methanol-*d*$_4$) of 3a-(benzo[*d*][1,3]dioxol-5-yl((diphenylmethylene)amino)methyl)-1,8-dimethyl-3,3a,8,8a-tetrahydropyrrolo[2,3-*b*]indol-2(1*H*)-one (3ca")**

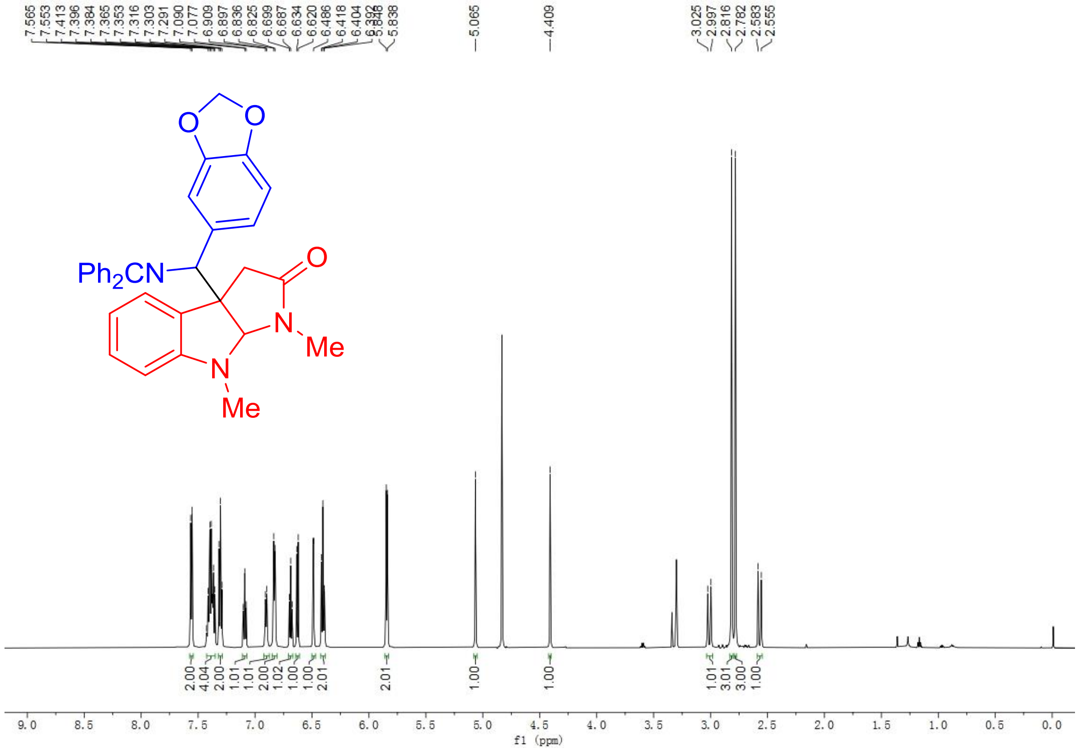

Figure S12. $^{13}C\{^{1}H\}$ **NMR spectra (150 MHz, Methanol-*d*$_4$) of 3a-(benzo[*d*][1,3]dioxol-5-yl((diphenylmethylene)amino)methyl)-1,8-dimethyl-3,3a,8,8a-tetrahydropyrrolo[2,3-*b*]indol-2(1*H*)-one (3ca")**

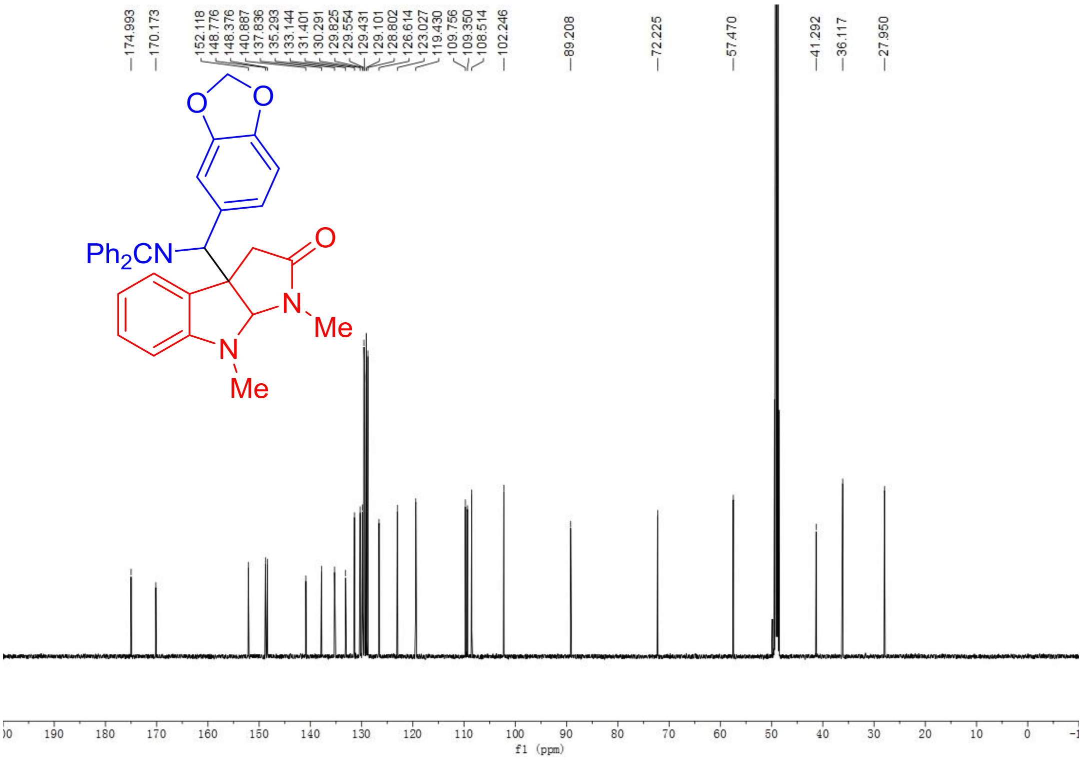

Figure S13. ^{1}H NMR spectra (400 MHz, Chloroform-*d*) of 3a-(((diphenylmethylene)amino)(4-fluorophenyl)methyl)-1,8-dimethyl-3,3a,8,8a-tetrahydropyrrolo[2,3-*b*]indol-2(1*H*)-one (3da(major))

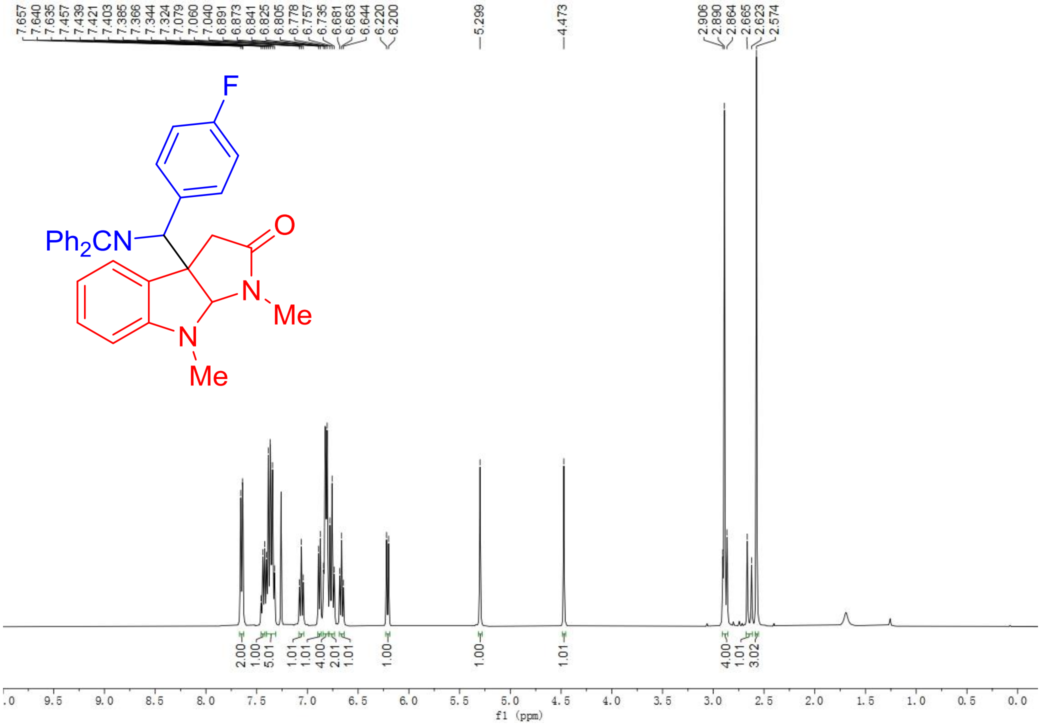

Figure S14. ^{13}C{^{1}H} NMR spectra (100 MHz, Chloroform-*d*) of 3a-(((diphenylmethylene)amino)(4-fluorophenyl)methyl)-1,8-dimethyl-3,3a,8,8a-tetrahydropyrrolo[2,3-*b*]indol-2(1*H*)-one (3da(major))

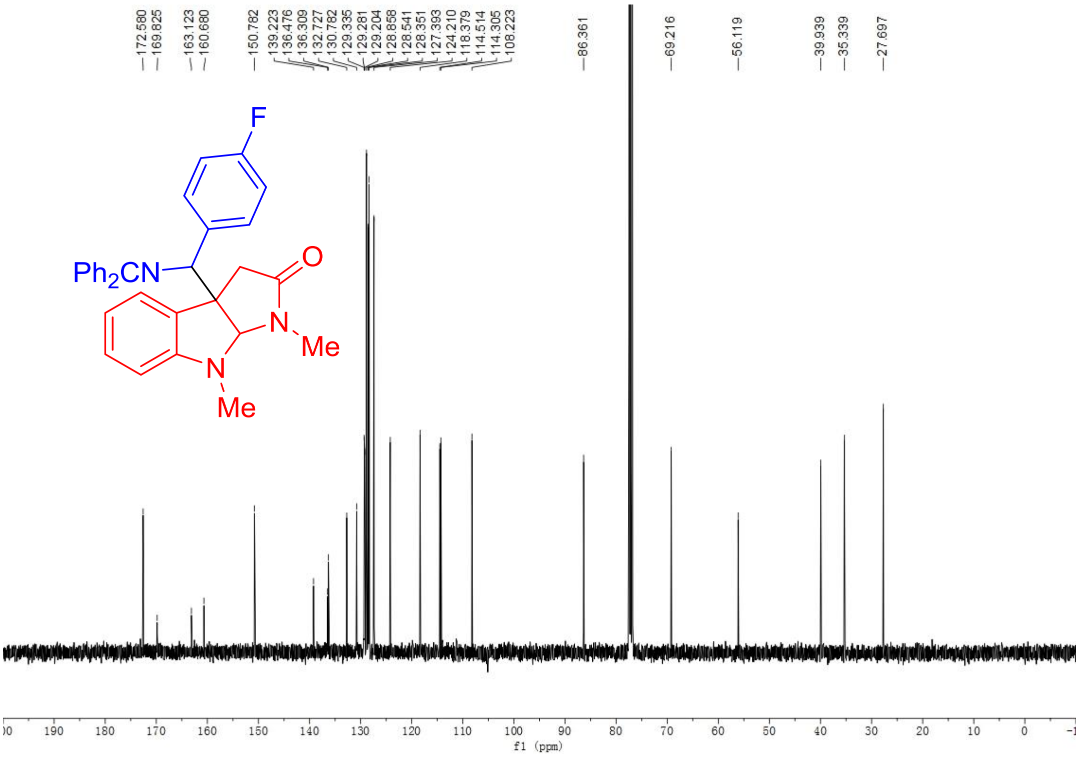

Figure S15. ^{19}F NMR spectra (377 MHz, Chloroform-*d*) of 3a-(((diphenylmethylene)amino)(4-fluorophenyl)methyl)-1,8-dimethyl-3,3a,8,8a-tetrahydropyrrolo[2,3-*b*]indol-2(1*H*)-one (3da(major)

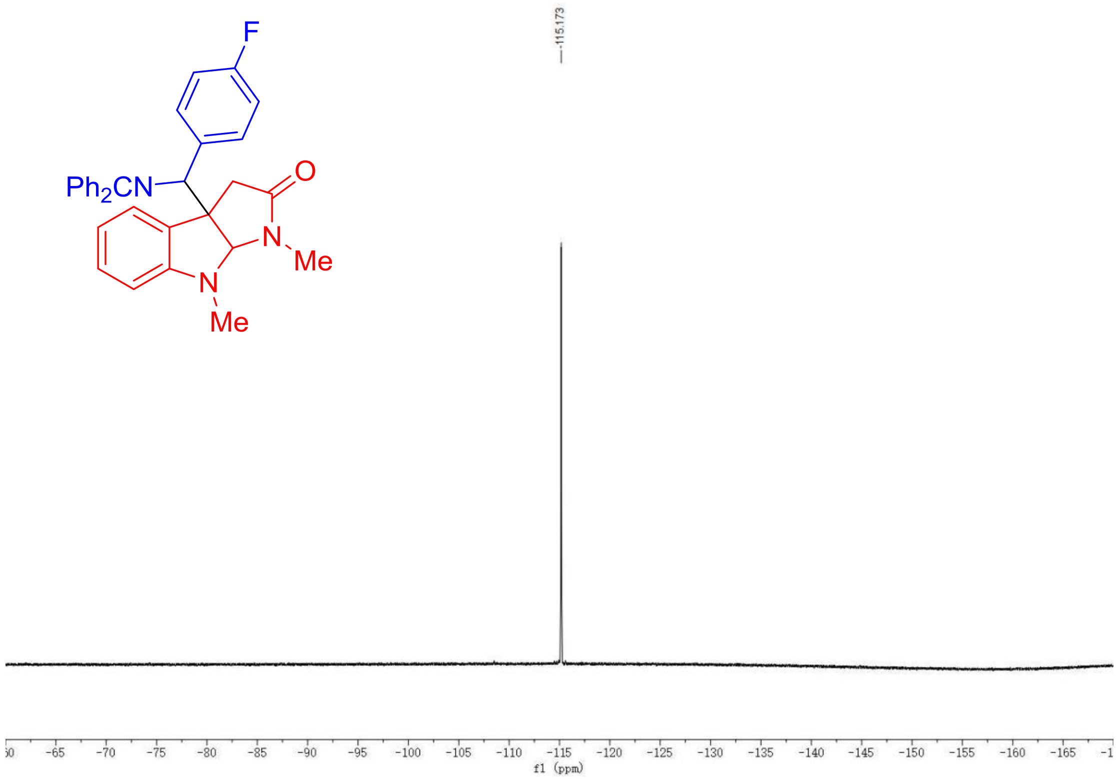

Figure S16. ^{1}H NMR spectra (400 MHz, Chloroform-*d*) of 3a-(((diphenylmethylene)amino)(4-fluorophenyl)methyl)-1,8-dimethyl-3,3a,8,8a-tetrahydropyrrolo[2,3-*b*]indol-2(1*H*)-one (3da(minor))

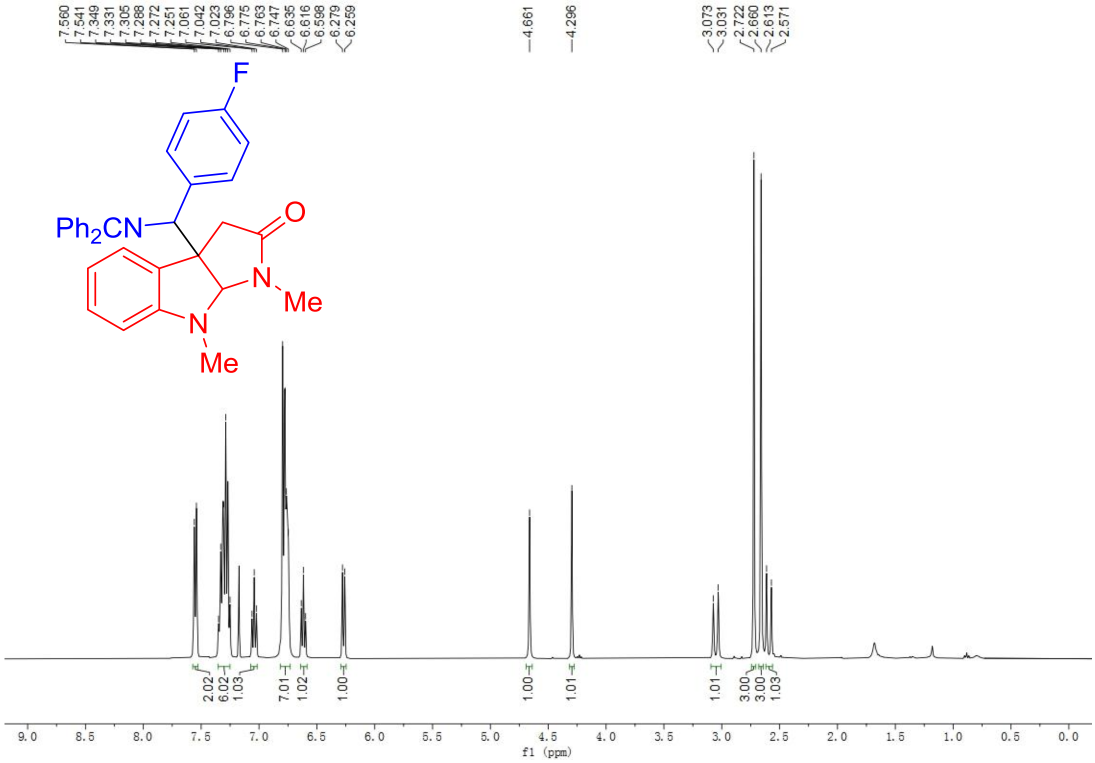

Figure S17. ^{13}C{^{1}H} NMR spectra (100 MHz, Chloroform-*d*) of 3a-(((diphenylmethylene)amino)(4-fluorophenyl)methyl)-1,8-dimethyl-3,3a,8,8a-tetrahydropyrrolo[2,3-*b*]indol-2(1*H*)-one (3da(minor))

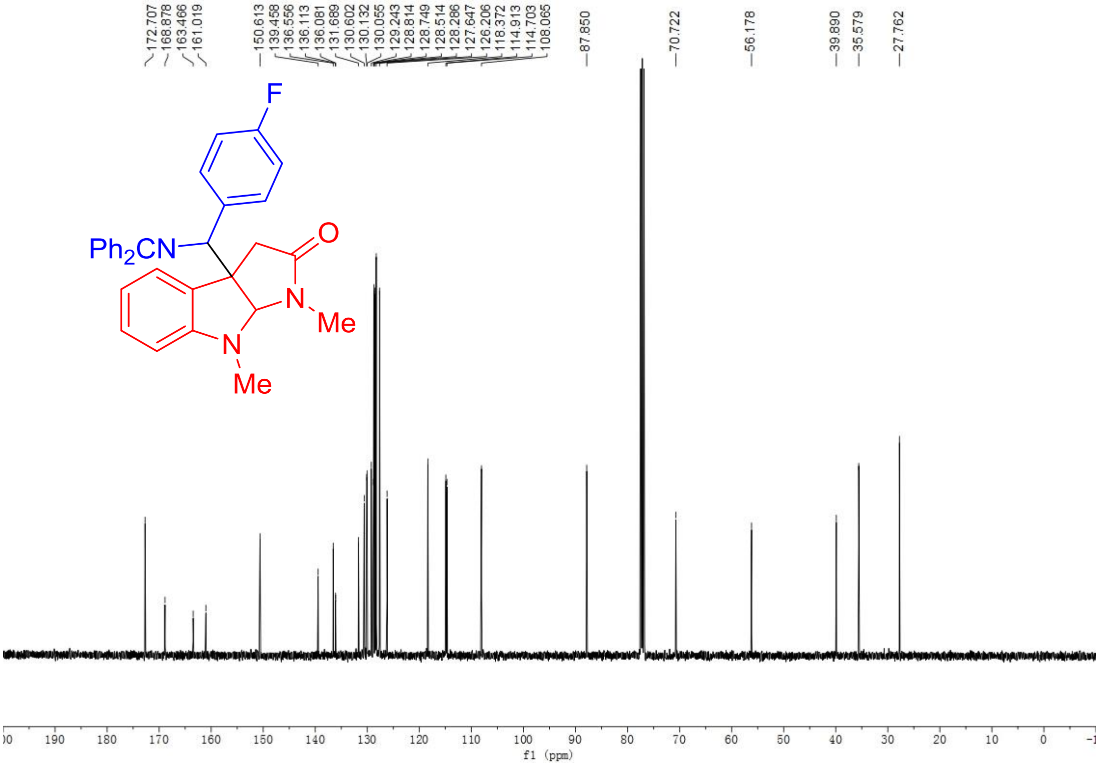

Figure S18. ^{19}F NMR spectra (377 MHz, Chloroform-*d*) of 3a-(((diphenylmethylene)amino)(4-fluorophenyl)methyl)-1,8-dimethyl-3,3a,8,8a-tetrahydropyrrolo[2,3-*b*]indol-2(1*H*)-one (3da(minor)

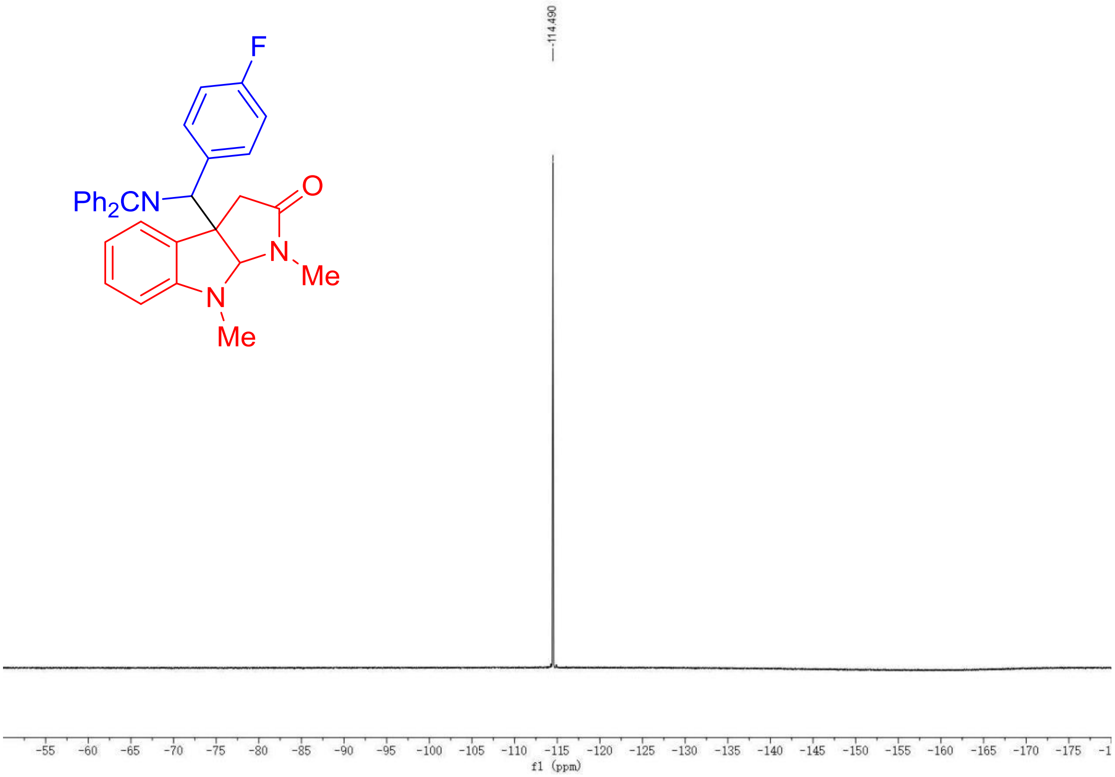

Figure S19. ^{1}H NMR spectra (600 MHz, Chloroform-*d*) of 3a-((4-chlorophenyl)((diphenylmethylene)amino)methyl)-1,8-dimethyl-3,3a,8,8a-tetrahydropyrrolo[2,3-*b*]indol-2(1*H*)-one (3ea(major))

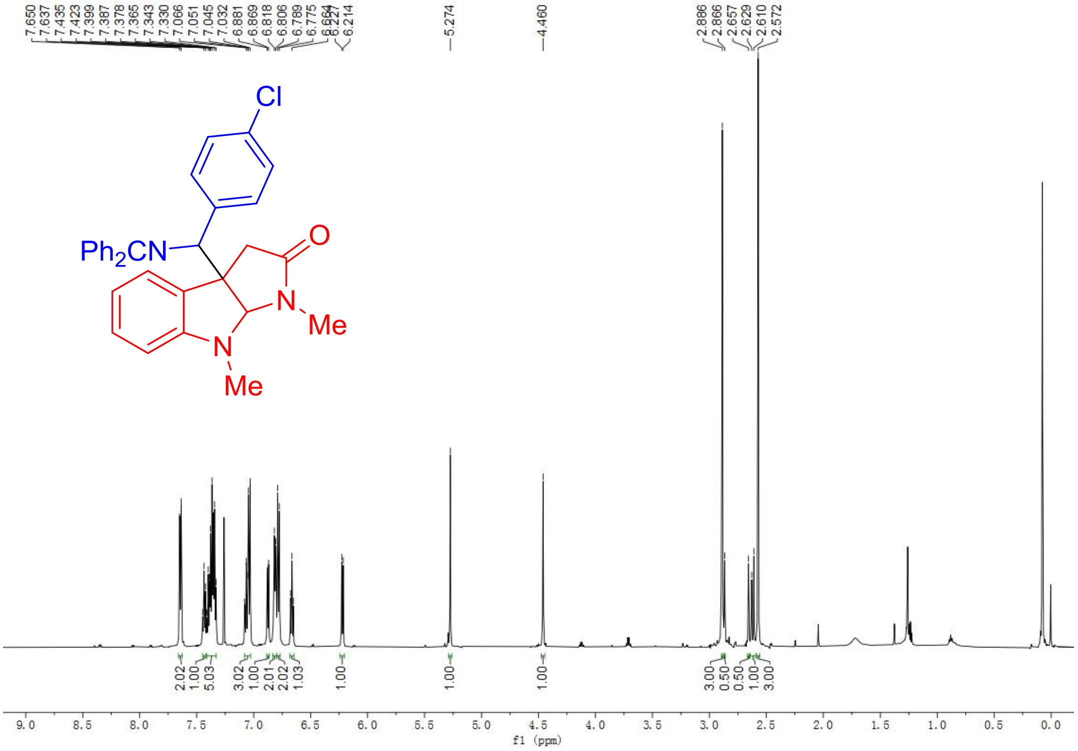

Figure S20. ^{13}C{^{1}H} NMR spectra (150 MHz, Chloroform-*d*) of 3a-((4-chlorophenyl)((diphenylmethylene)amino)methyl)-1,8-dimethyl-3,3a,8,8a-tetrahydropyrrolo[2,3-*b*]indol-2(1*H*)-one (3ea(major))

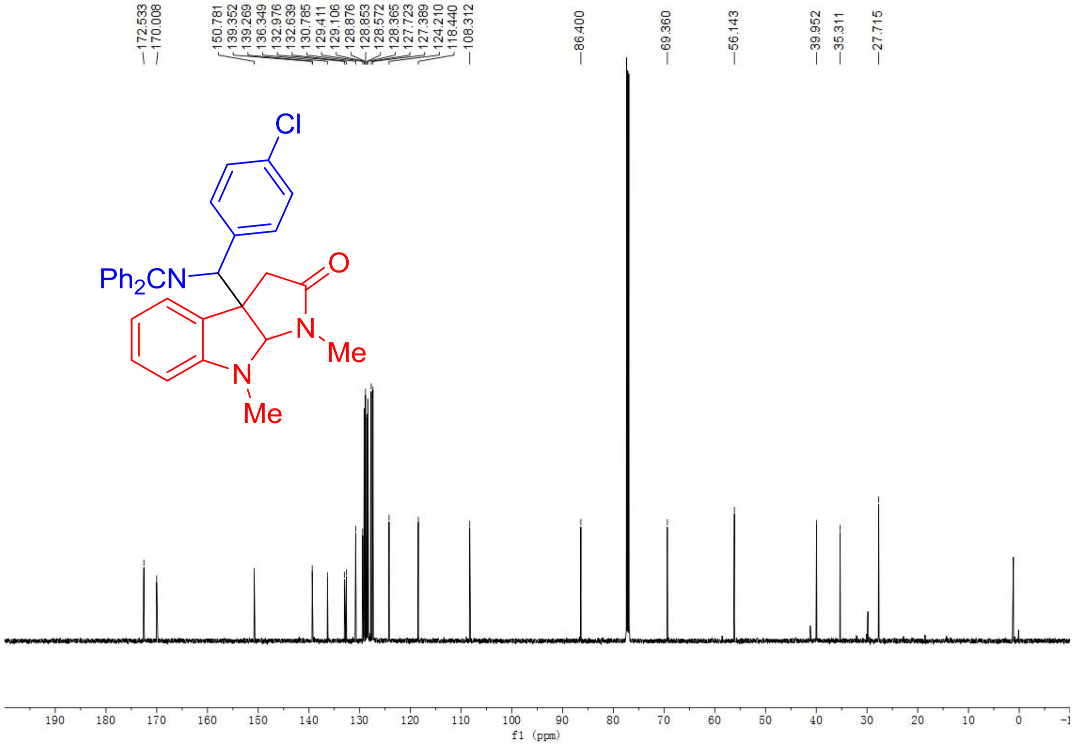

Figure S21. ^{1}H NMR spectra (600 MHz, Chloroform-*d*) of 3a-((4-chlorophenyl)((diphenylmethylene)amino)methyl)-1,8-dimethyl-3,3a,8,8a-tetrahydropyrrolo[2,3-*b*]indol-2(1*H*)-one (3ea(minor))

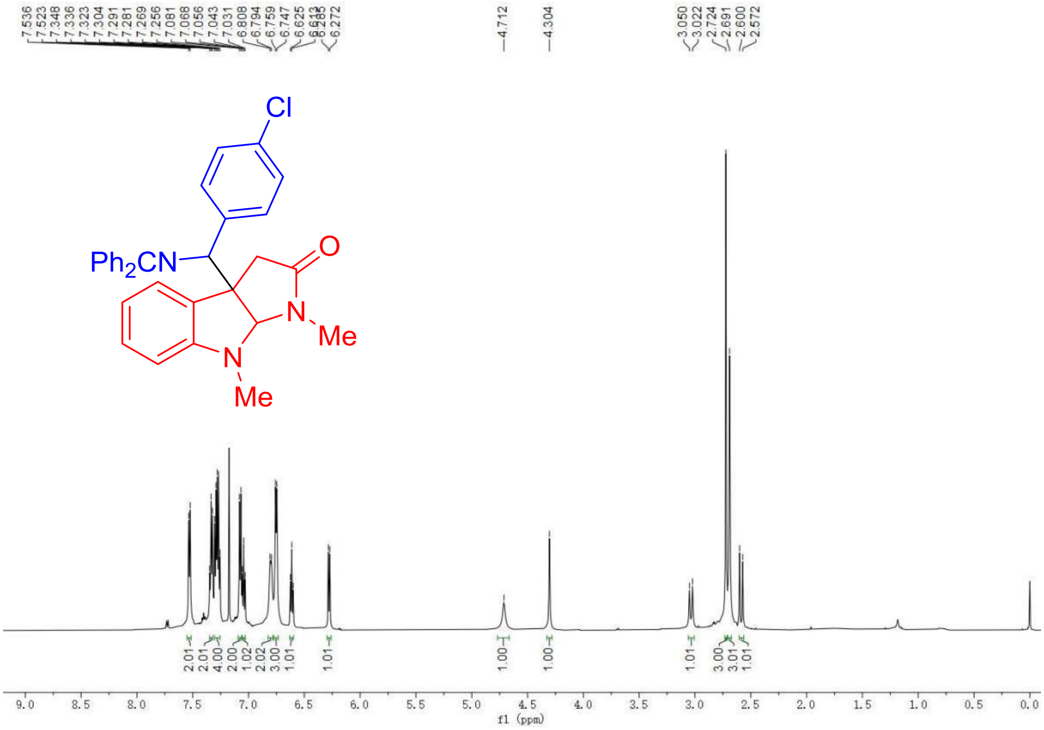

Figure S22. ^{13}C{^{1}H} NMR spectra (150 MHz, Chloroform-*d*) of 3a-((4-chlorophenyl)((diphenylmethylene)amino)methyl)-1,8-dimethyl-3,3a,8,8a-tetrahydropyrrolo[2,3-*b*]indol-2(1*H*)-one (3ea(minor))

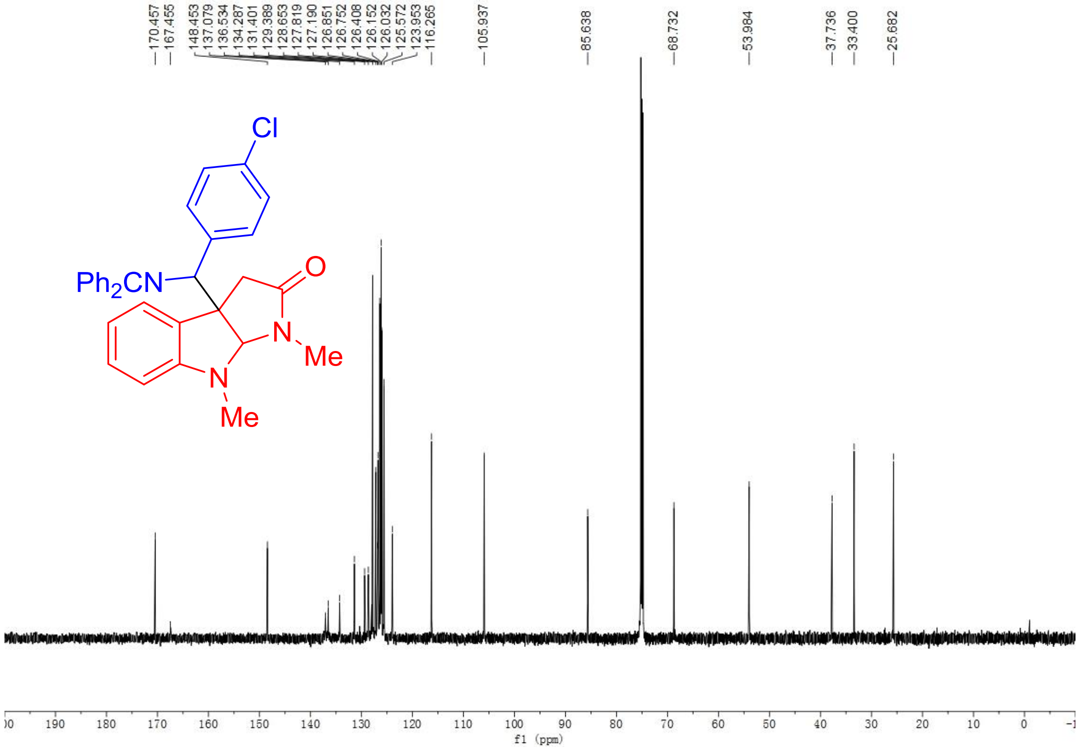

Figure S23. ^{1}H **NMR spectra (400 MHz, Chloroform-*d*) of 3a-((4-bromophenyl)((diphenylmethylene)amino)methyl)-1,8-dimethyl-3,3a,8,8a-tetrahydropyrrolo[2,3-*b*]indol-2(1*H*)-one (3fa')**

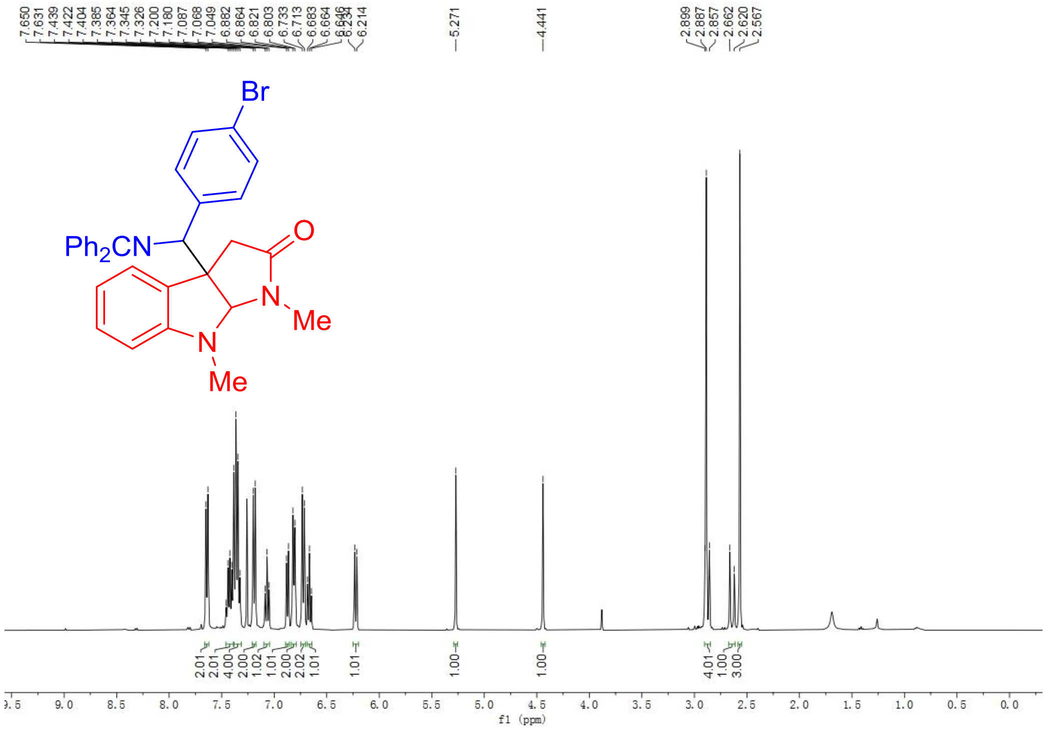

Figure S24. $^{13}C\{^{1}H\}$ **NMR spectra (100 MHz, Chloroform-*d*) of 3a-((4-bromophenyl)((diphenylmethylene)amino)methyl)-1,8-dimethyl-3,3a,8,8a-tetrahydropyrrolo[2,3-*b*]indol-2(1*H*)-one (3fa')**

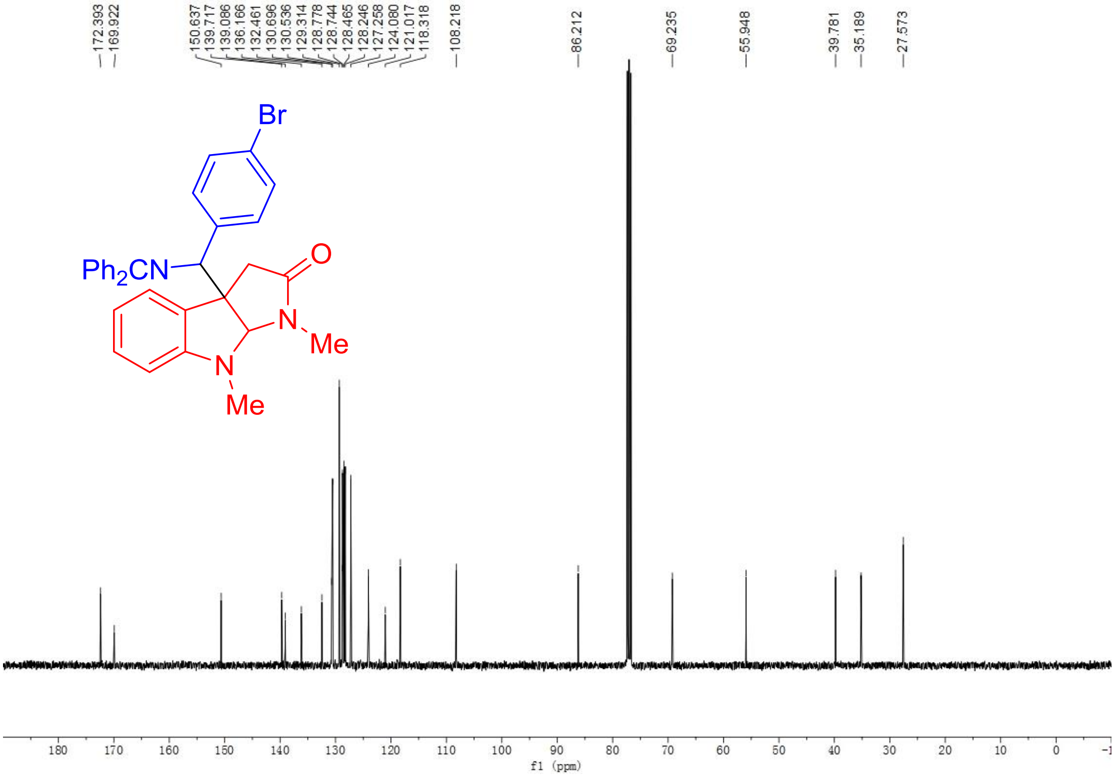

Figure S25. ^{1}H NMR spectra (400 MHz, Chloroform-*d*) of 3a-((4-bromophenyl)((diphenylmethylene)amino)methyl)-1,8-dimethyl-3,3a,8,8a-tetrahydropyrrolo[2,3-*b*]indol-2(1*H*)-one (3fa")

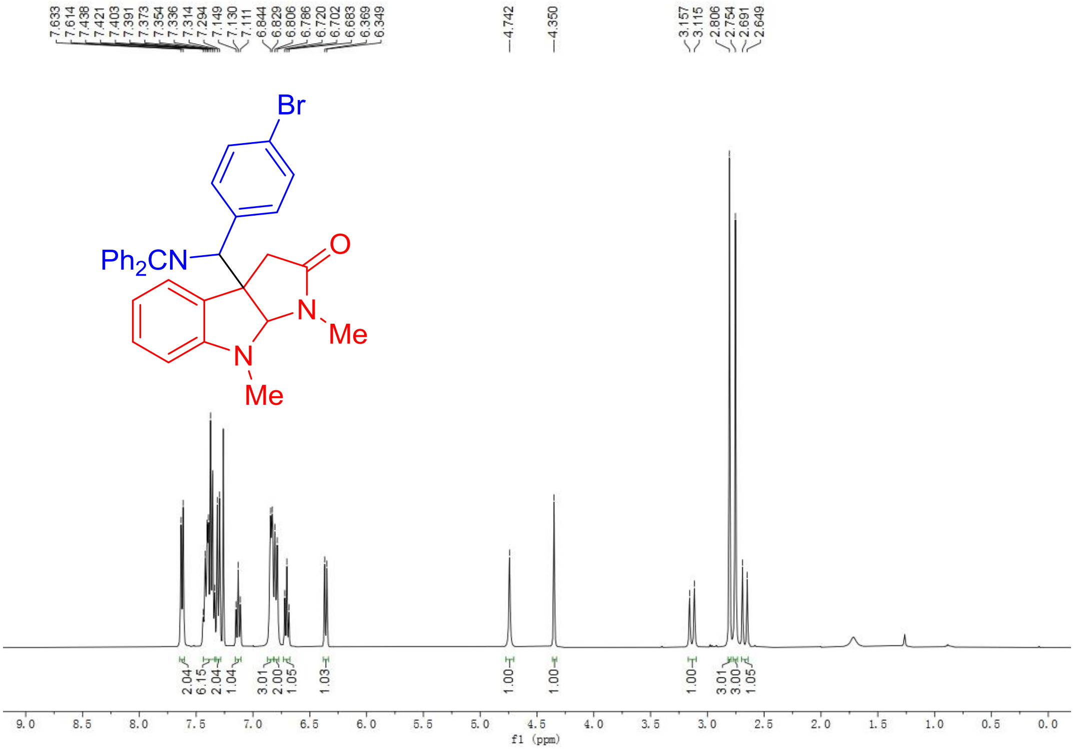

Figure S26. ^{13}C{^{1}H} NMR spectra (100 MHz, Chloroform-*d*) of 3a-((4-bromophenyl)((diphenylmethylene)amino)methyl)-1,8-dimethyl-3,3a,8,8a-tetrahydropyrrolo[2,3-*b*]indol-2(1*H*)-one (3fa")

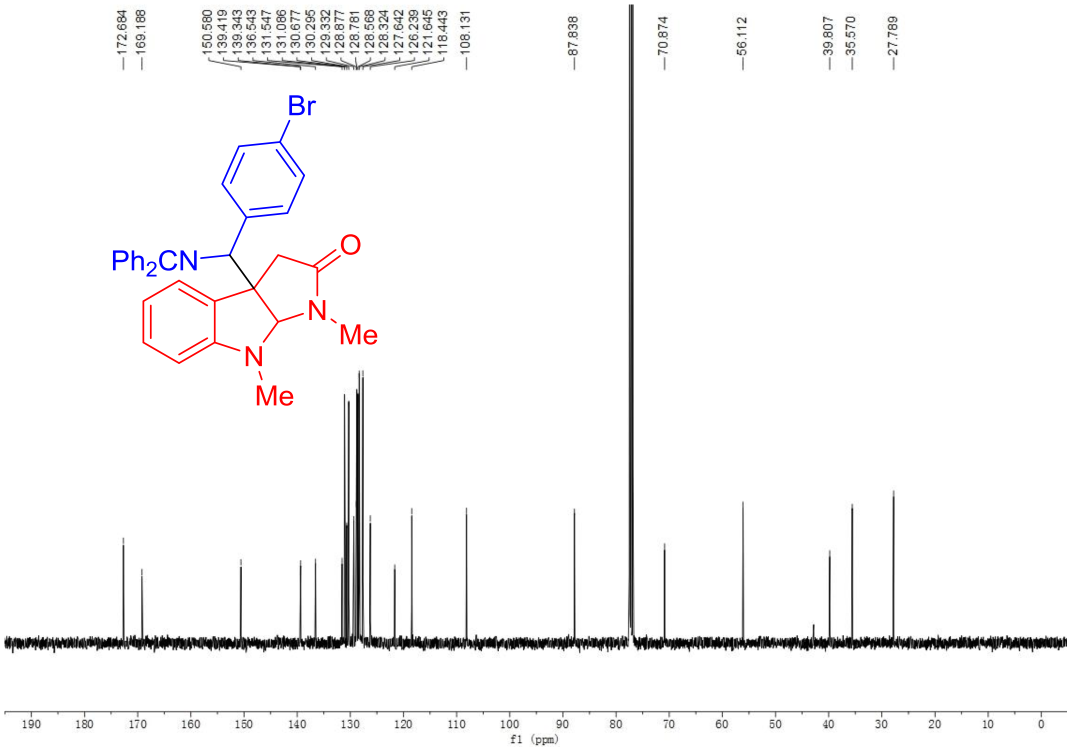

Figure S27. ^{1}H NMR spectra (600 MHz, Chloroform-*d*) of 3a-((3,5-difluorophenyl)((diphenylmethylene)amino)methyl)-1,8-dimethyl-3,3a,8,8a-tetrahydropyrrolo[2,3-*b*]indol-2(1*H*)-one (3ga(major))

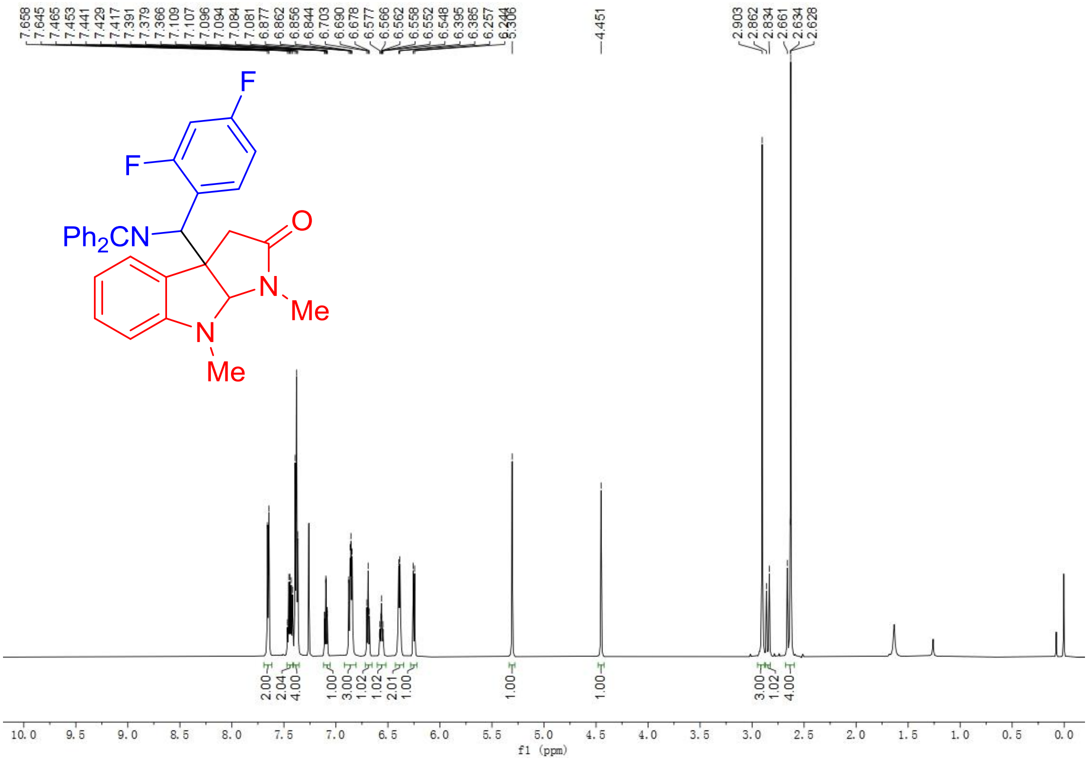

Figure S28. ^{13}C{^{1}H} NMR spectra (150 MHz, Chloroform-*d*) of 3a-((3,5-difluorophenyl)((diphenylmethylene)amino)methyl)-1,8-dimethyl-3,3a,8,8a-tetrahydropyrrolo[2,3-*b*]indol-2(1*H*)-one (3ga(major))

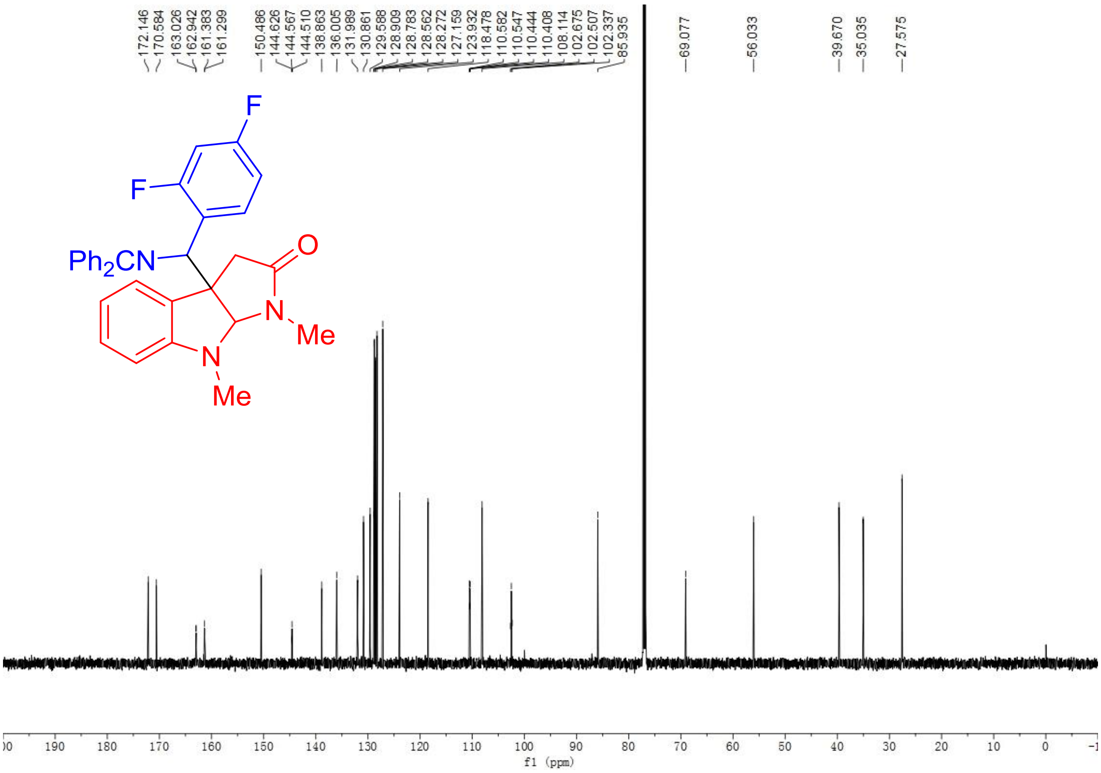

Figure S29. ^{19}**F NMR spectra (565 MHz, Chloroform-*d*) of 3a-((3,5-difluorophenyl)((diphenylmethylene)amino)methyl)-1,8-dimethyl-3,3a,8,8a-tetrahydropyrrolo[2,3-*b*]indol-2(1*H*)-one (3ga(major)**

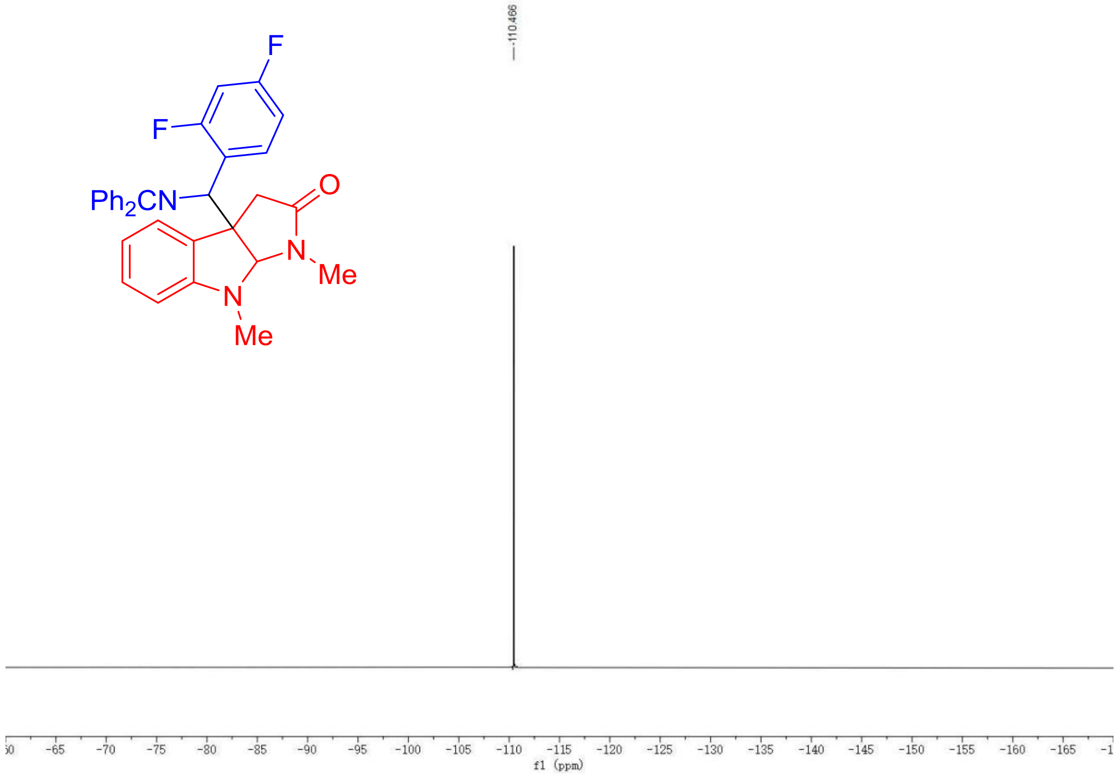

Figure S30. ^{1}**H NMR spectra (600 MHz, Methanol-*d*$_4$) of 3a-((3,5-difluorophenyl)((diphenylmethylene)amino)methyl)-1,8-dimethyl-3,3a,8,8a-tetrahydropyrrolo[2,3-*b*]indol-2(1*H*)-one (3ga(minor))**

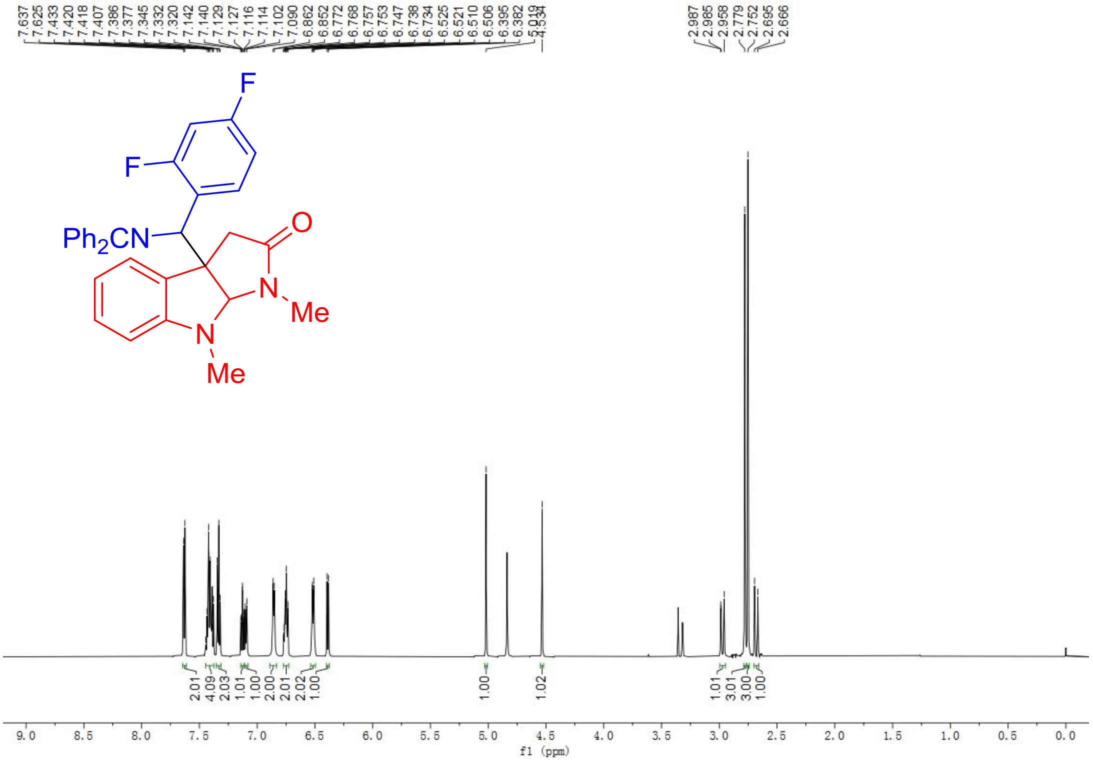

Figure S31. ^{13}**C{**^{1}**H} NMR spectra (150 MHz, Methanol-*d*$_4$) of 3a-((3,5-difluorophenyl)((diphenylmethylene)amino)methyl)-1,8-dimethyl-3,3a,8,8a-tetrahydropyrrolo[2,3-*b*]indol-2(1*H*)-one (3ga(minor))**

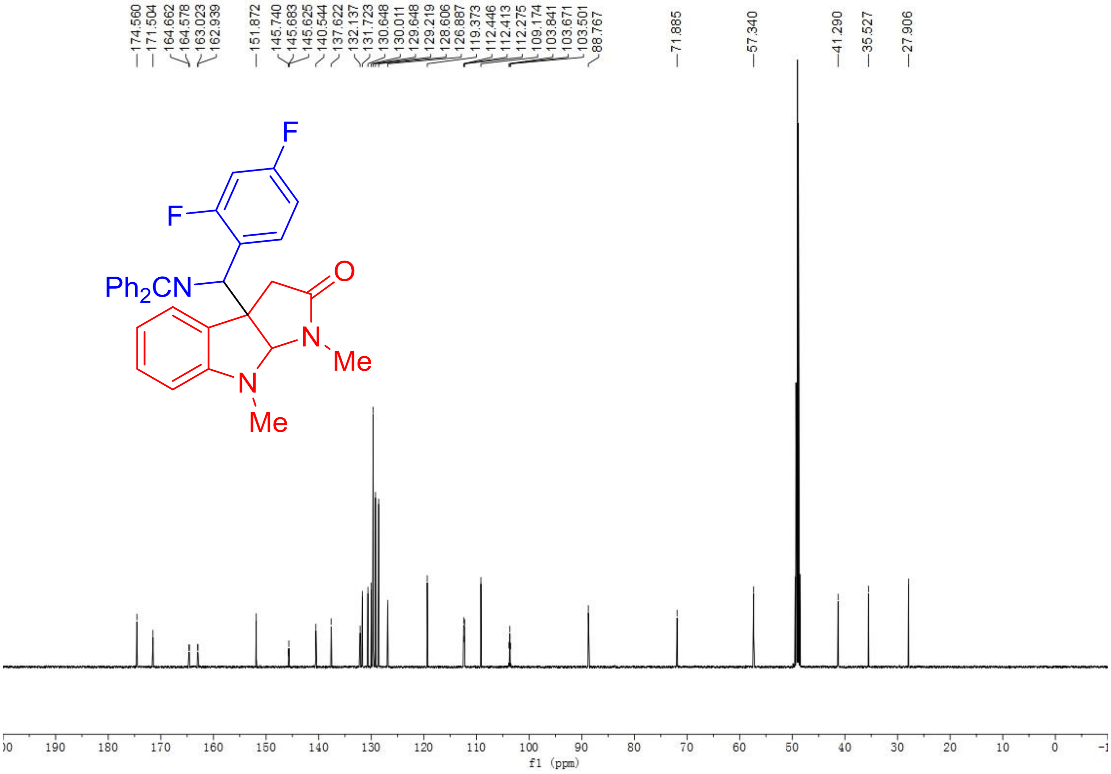

Figure S32. ^{19}F **NMR spectra (565 MHz, Methanol-*d*$_4$) of 3a-((3,5-difluorophenyl)((diphenylmethylene)amino)methyl)-1,8-dimethyl-3,3a,8,8a-tetrahydropyrrolo[2,3-*b*]indol-2(1*H*)-one (3ga(minor))**

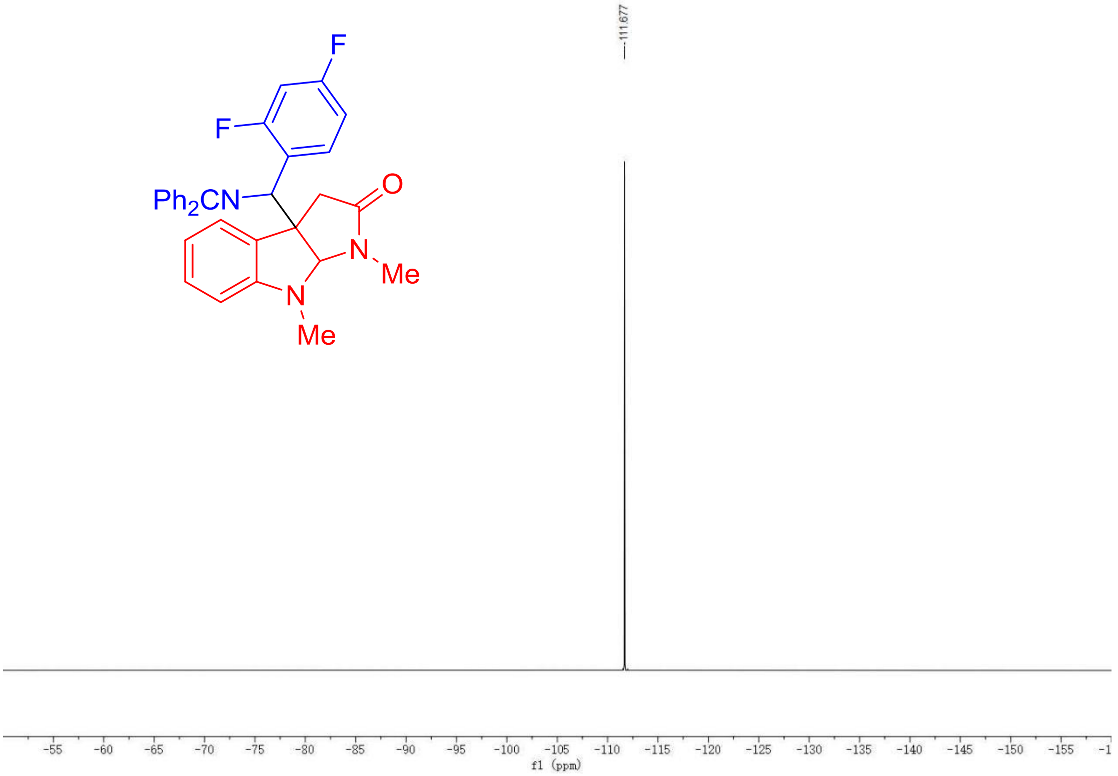

Figure S33. ^{1}H **NMR spectra (600 MHz, Chloroform-*d*) of 3a-(((diphenylmethylene)amino)(4-(trifluoromethyl)phenyl)methyl)-1,8-dimethyl-3,3a,8,8a-tetrahydropyrrolo[2,3-*b*]indol-2(1*H*)-one (3ha(major))**

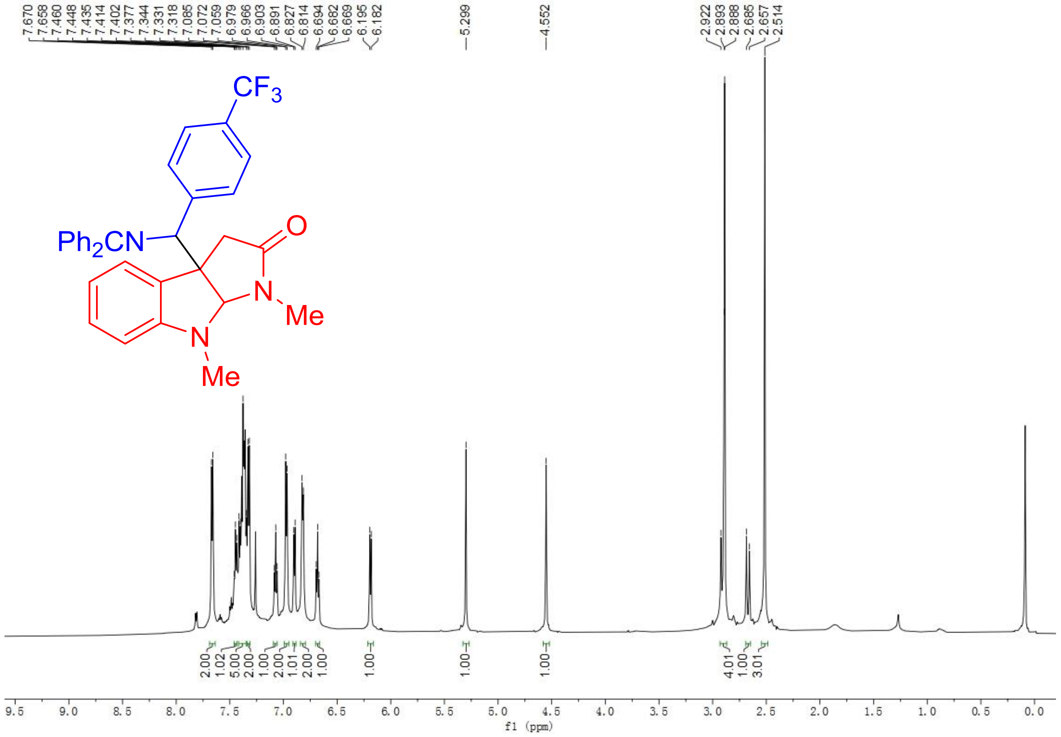

Figure S34. $^{13}C\{^{1}H\}$ **NMR spectra (150 MHz, Chloroform-*d*) of 3a-(((diphenylmethylene)amino)(4-(trifluoromethyl)phenyl)methyl)-1,8-dimethyl-3,3a,8,8a-tetrahydropyrrolo[2,3-*b*]indol-2(1*H*)-one (3ha(major))**

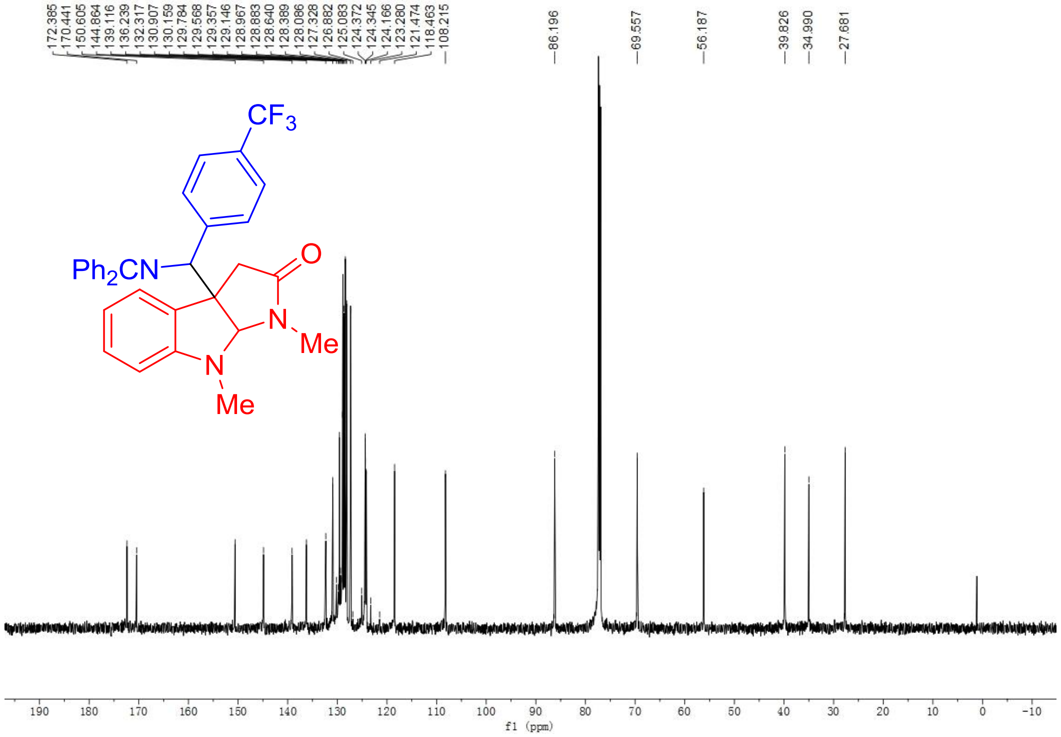

Figure S35. ^{19}**F NMR spectra (565 MHz, Chloroform-*d*) of 3a-(((diphenylmethylene)amino)(4-(trifluoromethyl)phenyl)methyl)-1,8-dimethyl-3,3a,8,8a-tetrahydropyrrolo[2,3-*b*]indol-2(1*H*)-one (3ha(major))**

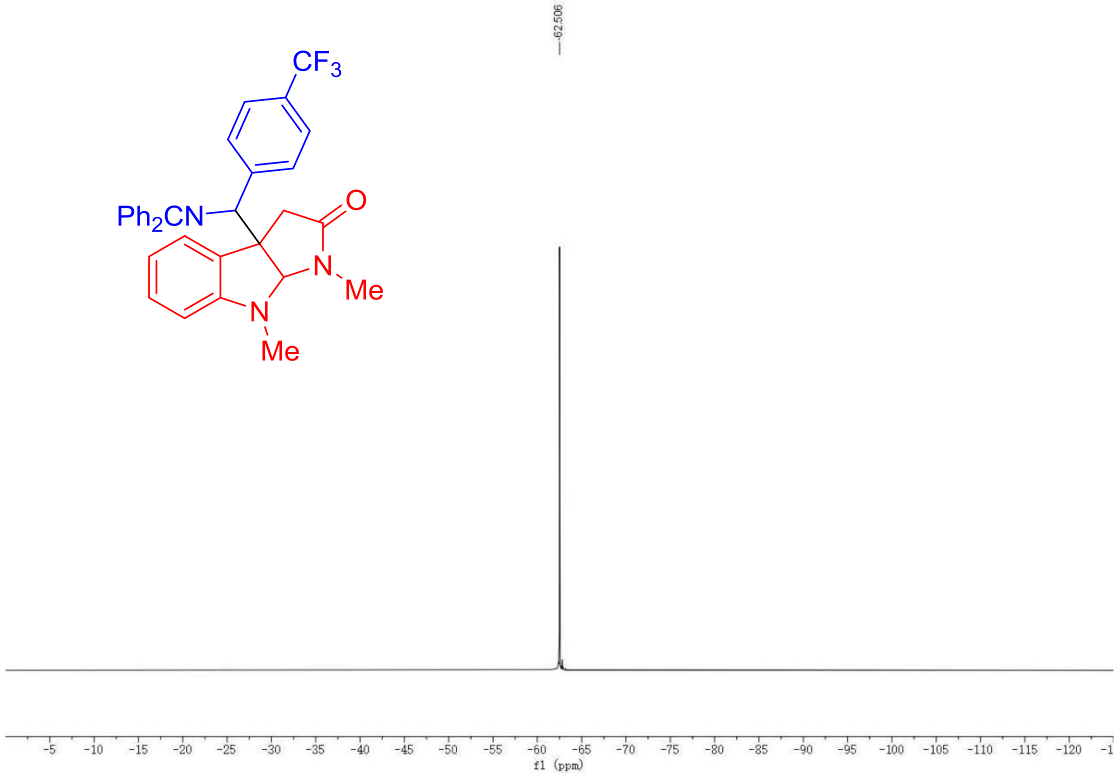

Figure S36. ^{1}H NMR spectra (600 MHz, Chloroform-*d*) of 3a-(((diphenylmethylene)amino)(4-(trifluoromethyl)phenyl)methyl)-1,8-dimethyl-3,3a,8,8a-tetrahydropyrrolo[2,3-*b*]indol-2(1*H*)-one (3ha(minor))

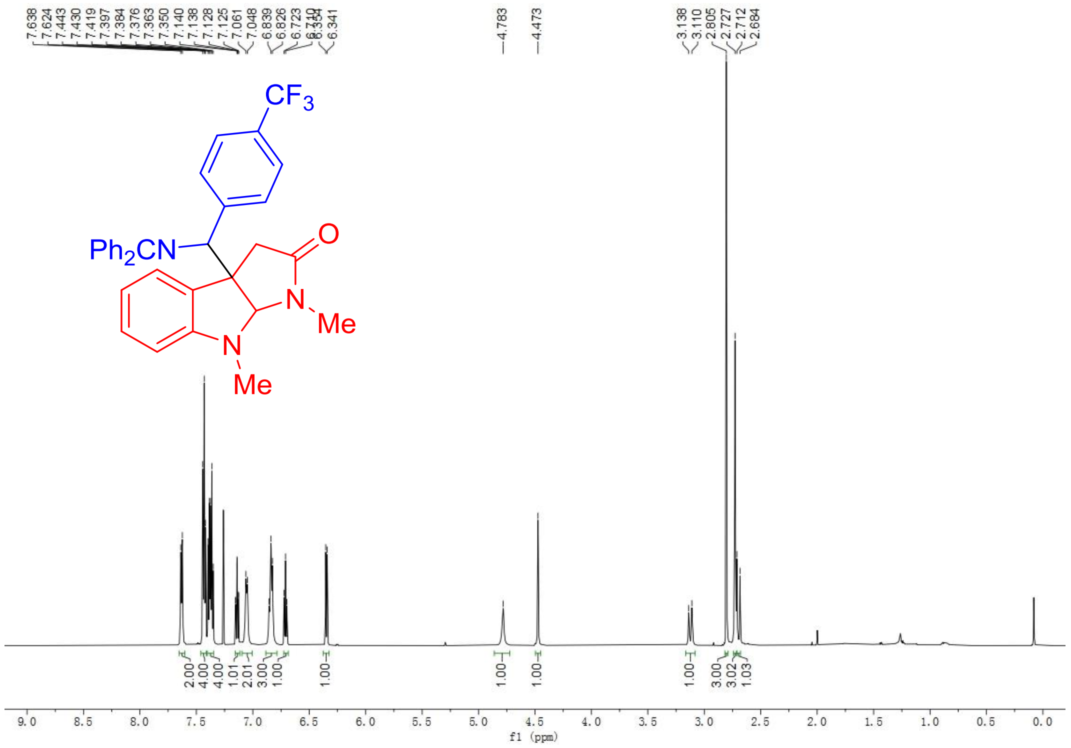

Figure S37. ^{13}C{^{1}H} NMR spectra (150 MHz, Chloroform-*d*) of 3a-(((diphenylmethylene)amino)(4-(trifluoromethyl)phenyl)methyl)-1,8-dimethyl-3,3a,8,8a-tetrahydropyrrolo[2,3-*b*]indol-2(1*H*)-one (3ha(minor))

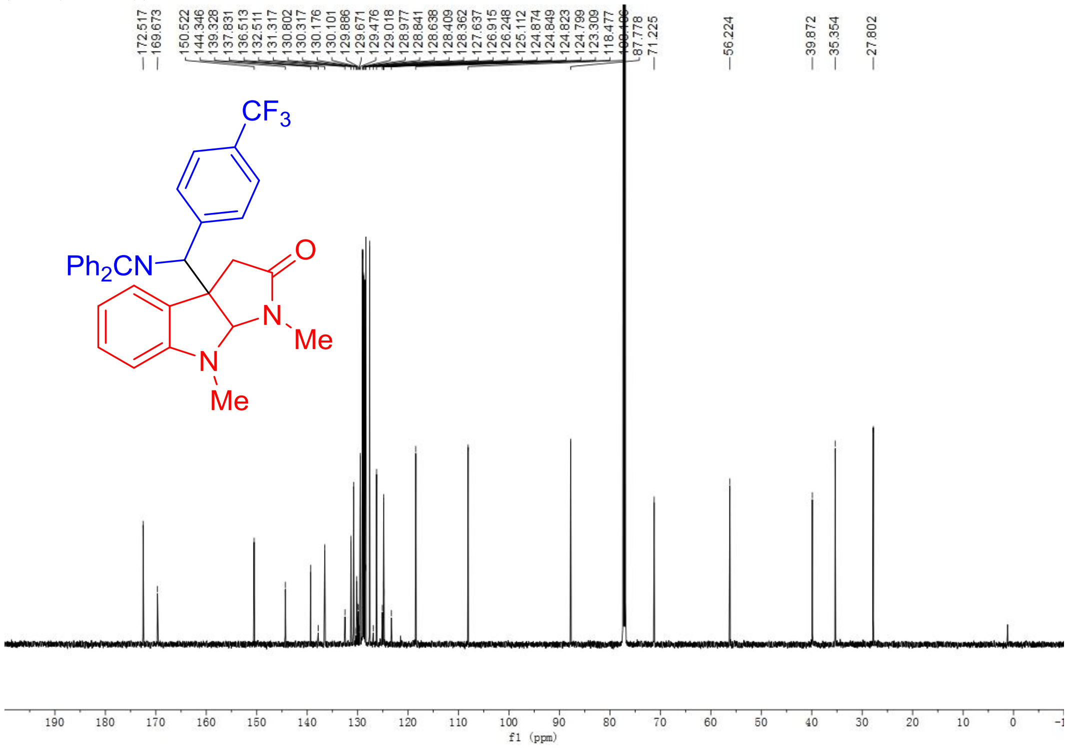

Figure S38. ^{19}F NMR spectra (565 MHz, Chloroform-*d*) of 3a-(((diphenylmethylene)amino)(4-(trifluoromethyl)phenyl)methyl)-1,8-dimethyl-3,3a,8,8a-tetrahydropyrrolo[2,3-*b*]indol-2(1*H*)-one (3ha(minor))

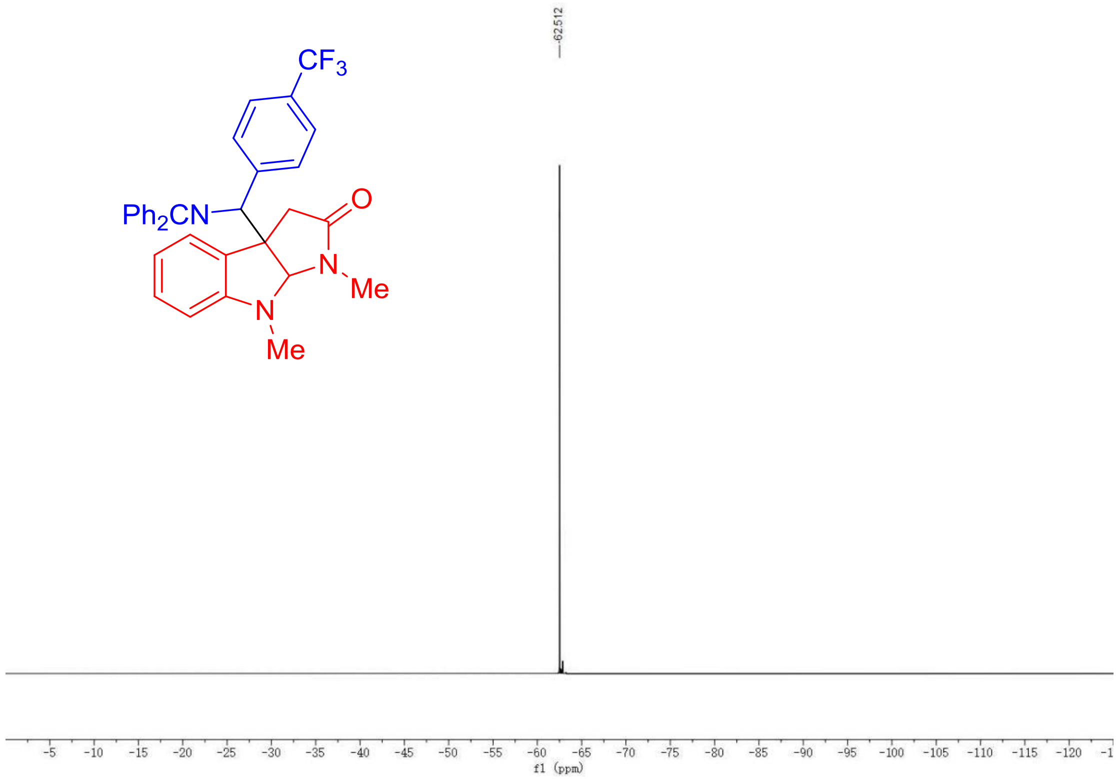

Figure S39. ^{1}**H NMR spectra (600 MHz, Methanol-*d*$_4$) of 3a-(((diphenylmethylene)amino)(o-tolyl)methyl)-1,8-dimethyl-3,3a,8,8a-tetrahydropyrrolo[2,3-*b*]indol-2(1*H*)-one (3ia')**

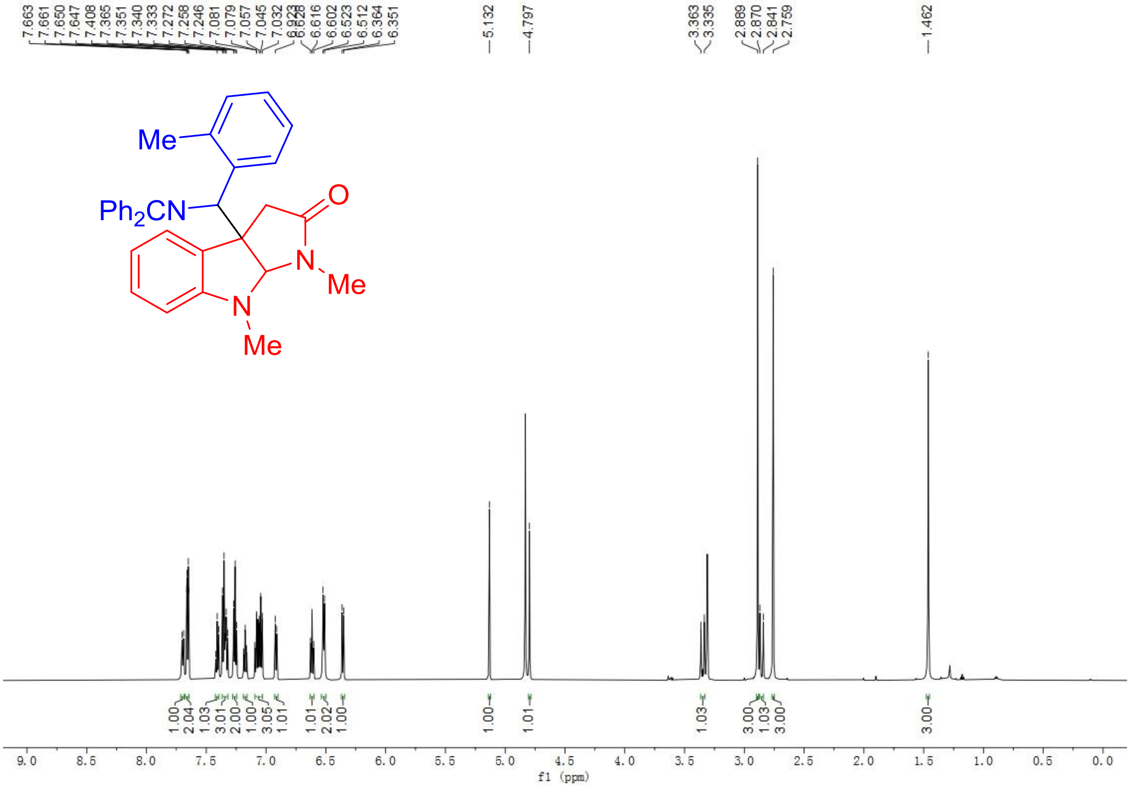

Figure S40. ^{13}**C{**^{1}**H} NMR spectra (150 MHz, Methanol-*d*$_4$) of 3a-(((diphenylmethylene)amino)(o-tolyl)methyl)-1,8-dimethyl-3,3a,8,8a-tetrahydropyrrolo[2,3-*b*]indol-2(1*H*)-one (3ia')**

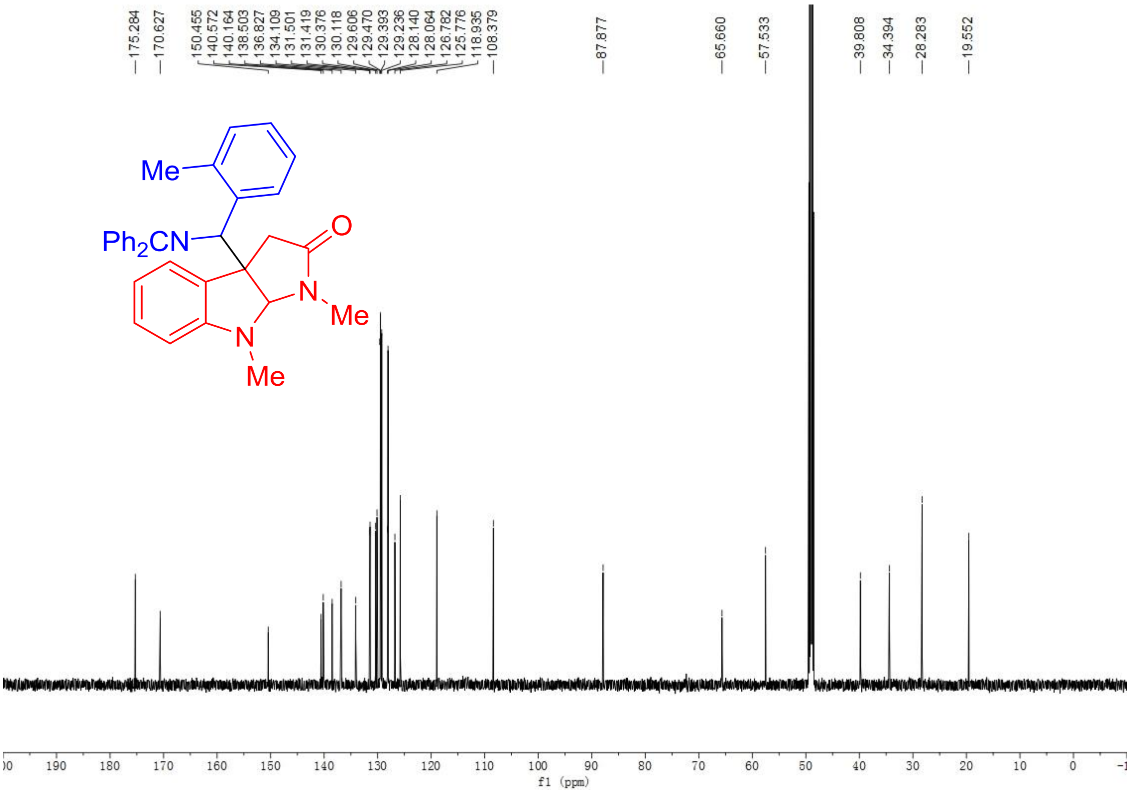

Figure S41. ^{1}H NMR spectra (600 MHz, Methanol-*d*$_4$) of 3a-(((diphenylmethylene)amino)(o-tolyl)methyl)-1,8-dimethyl-3,3a,8,8a-tetrahydropyrrolo[2,3-*b*]indol-2(1*H*)-one (3ia”)

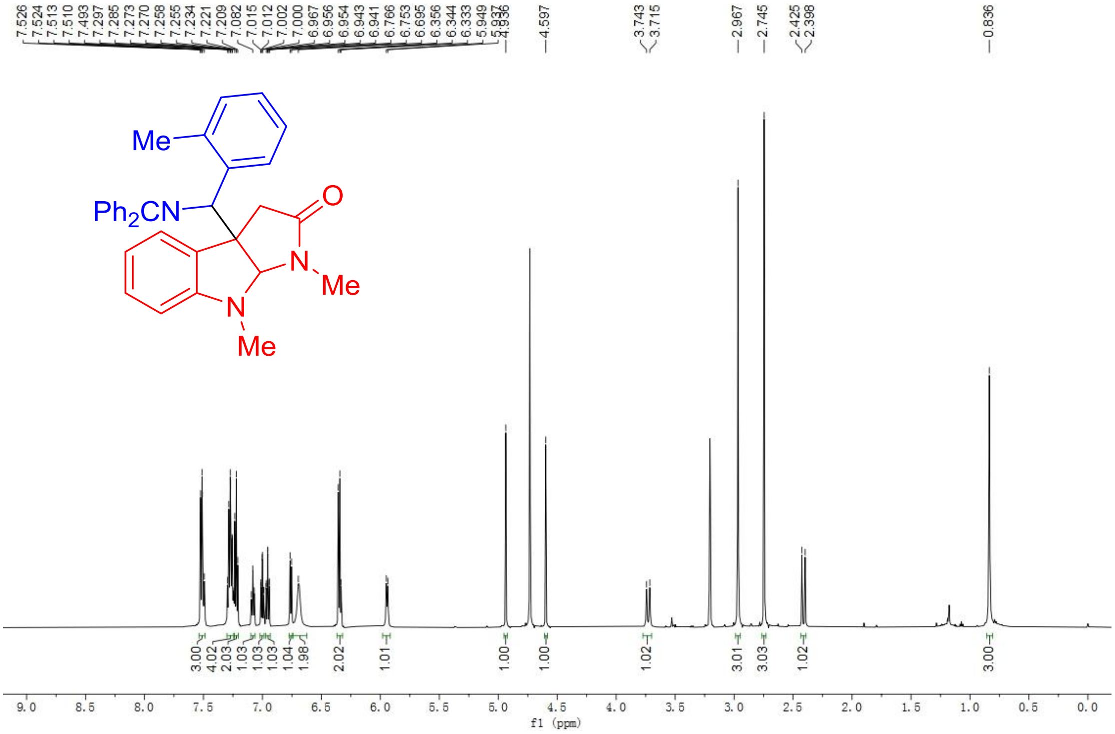

Figure S42. ^{13}C{^{1}H} NMR spectra (150 MHz, Methanol-*d*$_4$) of 3a-(((diphenylmethylene)amino)(o-tolyl)methyl)-1,8-dimethyl-3,3a,8,8a-tetrahydropyrrolo[2,3-*b*]indol-2(1*H*)-one (3ia”)

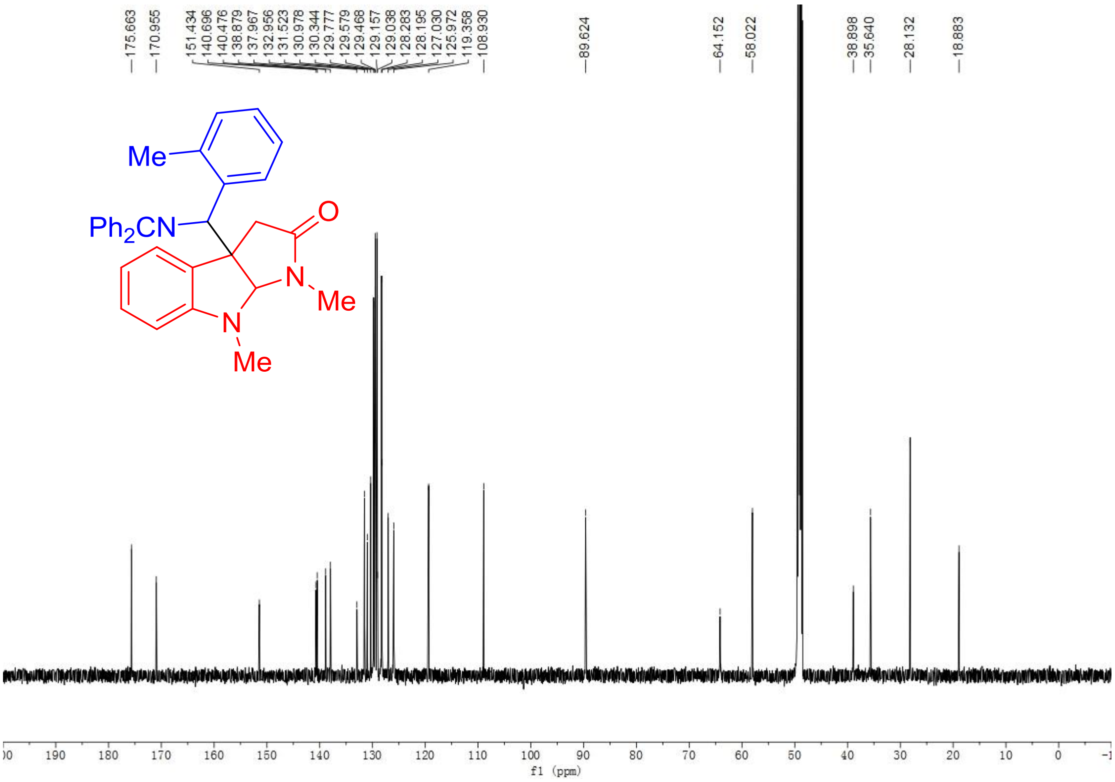

Figure S43. ^{1}H NMR spectra (600 MHz, Methanol-*d*$_4$) of 3a-(((diphenylmethylene)amino)(naphthalen-1-yl)methyl)-1,8-dimethyl-3,3a,8,8a-tetrahydropyrrolo[2,3-*b*]indol-2(1*H*)-one (3ja(major))

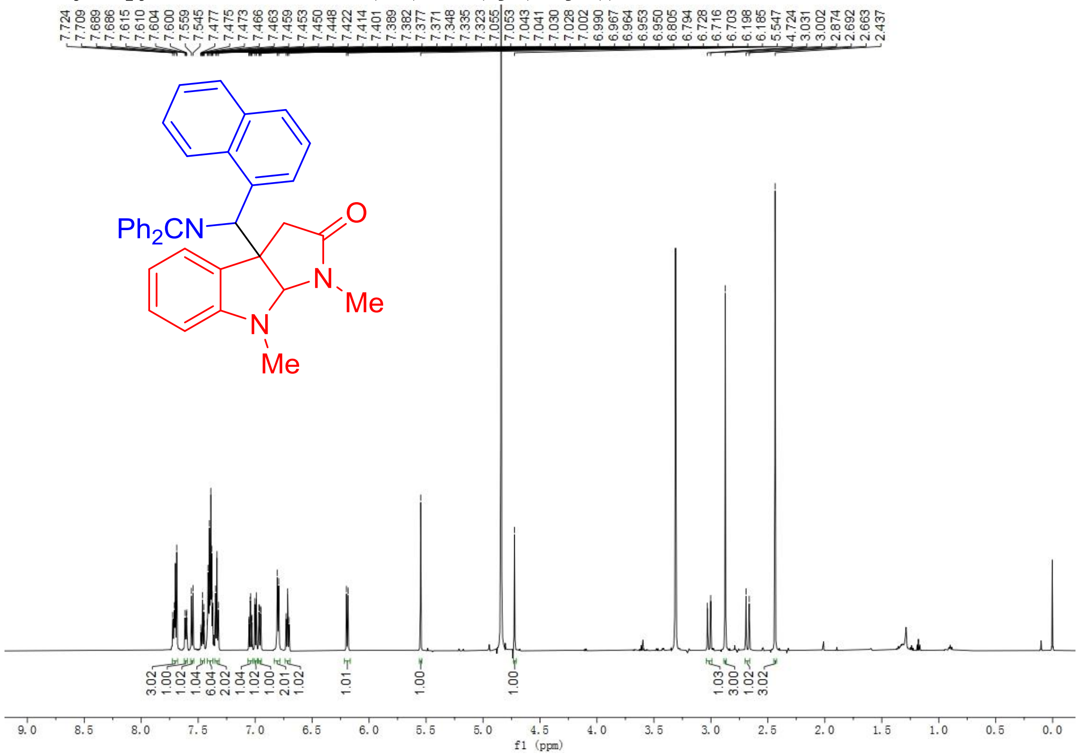

Figure S44. ^{13}C{^{1}H} NMR spectra (150 MHz, Methanol-*d*$_4$) of 3a-(((diphenylmethylene)amino)(naphthalen-1-yl)methyl)-1,8-dimethyl-3,3a,8,8a-tetrahydropyrrolo[2,3-*b*]indol-2(1*H*)-one (3ja(major))

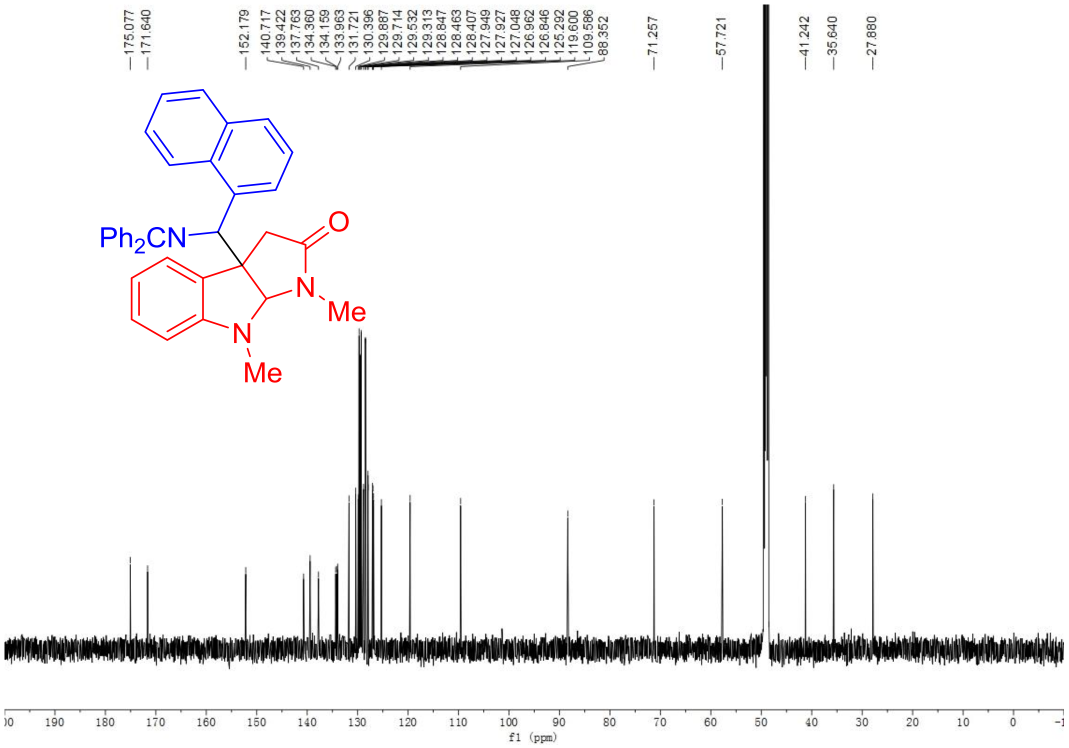

Figure S45. ^{1}H NMR spectra (600 MHz, Methanol-d_4) of 3a-(((diphenylmethylene)amino)(naphthalen-1-yl)methyl)-1,8-dimethyl-3,3a,8,8a-tetrahydropyrrolo[2,3-*b*]indol-2(1*H*)-one (3ja(minor))

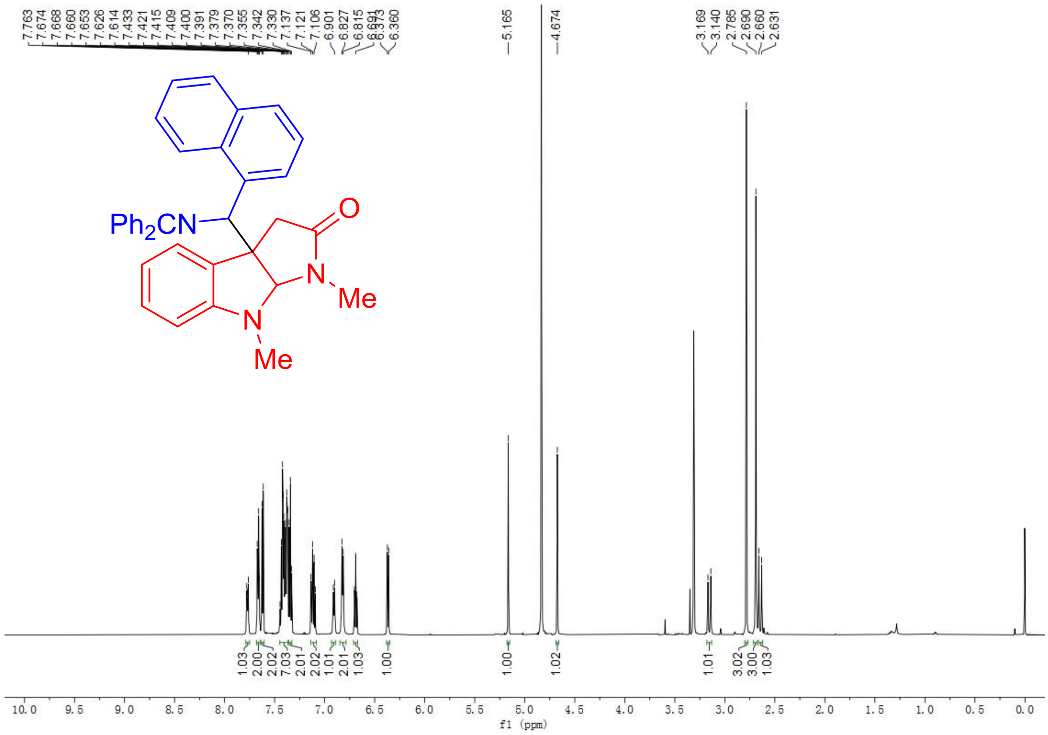

Figure S46. $^{13}C\{^{1}H\}$ NMR spectra (150 MHz, Methanol-d_4) of 3a-(((diphenylmethylene)amino)(naphthalen-1-yl)methyl)-1,8-dimethyl-3,3a,8,8a-tetrahydropyrrolo[2,3-*b*]indol-2(1*H*)-one (3ja(minor))

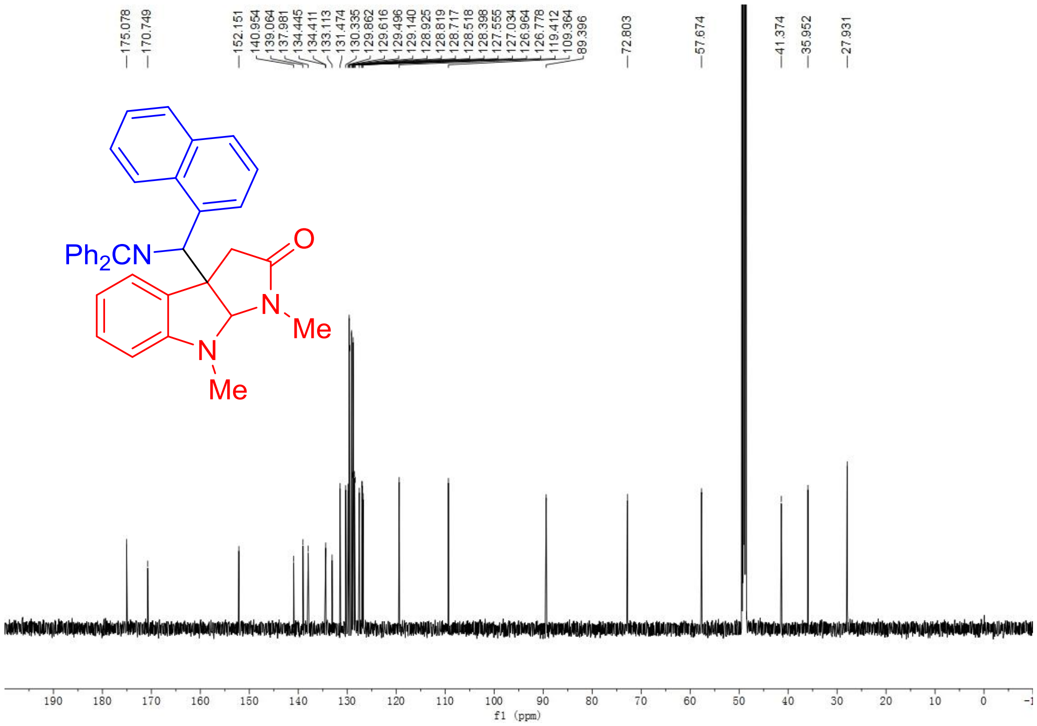

Figure S47. ^{1}H NMR spectra (400 MHz, Chloroform-*d*) of 3a-(((diphenylmethylene)amino)(pyridin-3-yl)methyl)-1,8-dimethyl-3,3a,8,8a-tetrahydropyrrolo[2,3-*b*]indol-2(1*H*)-one (3ka(major))

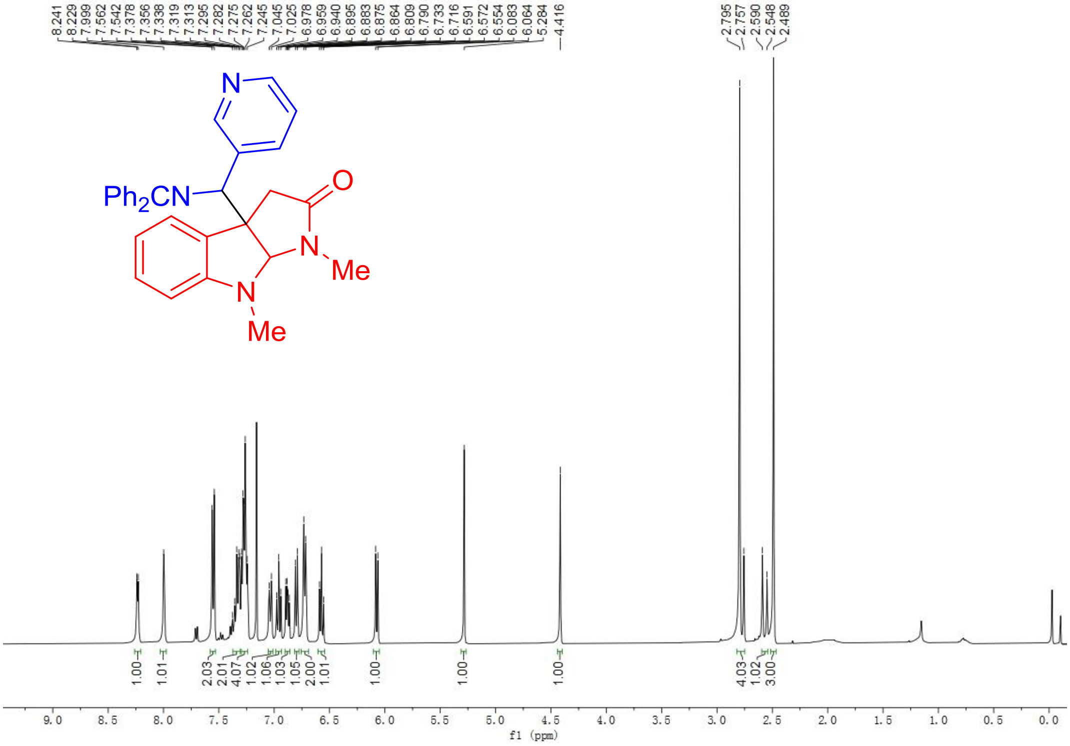

Figure S48. ^{13}C{^{1}H} NMR spectra (100 MHz, Chloroform-*d*) of 3a-(((diphenylmethylene)amino)(pyridin-3-yl)methyl)-1,8-dimethyl-3,3a,8,8a-tetrahydropyrrolo[2,3-*b*]indol-2(1*H*)-one (3ka(major))

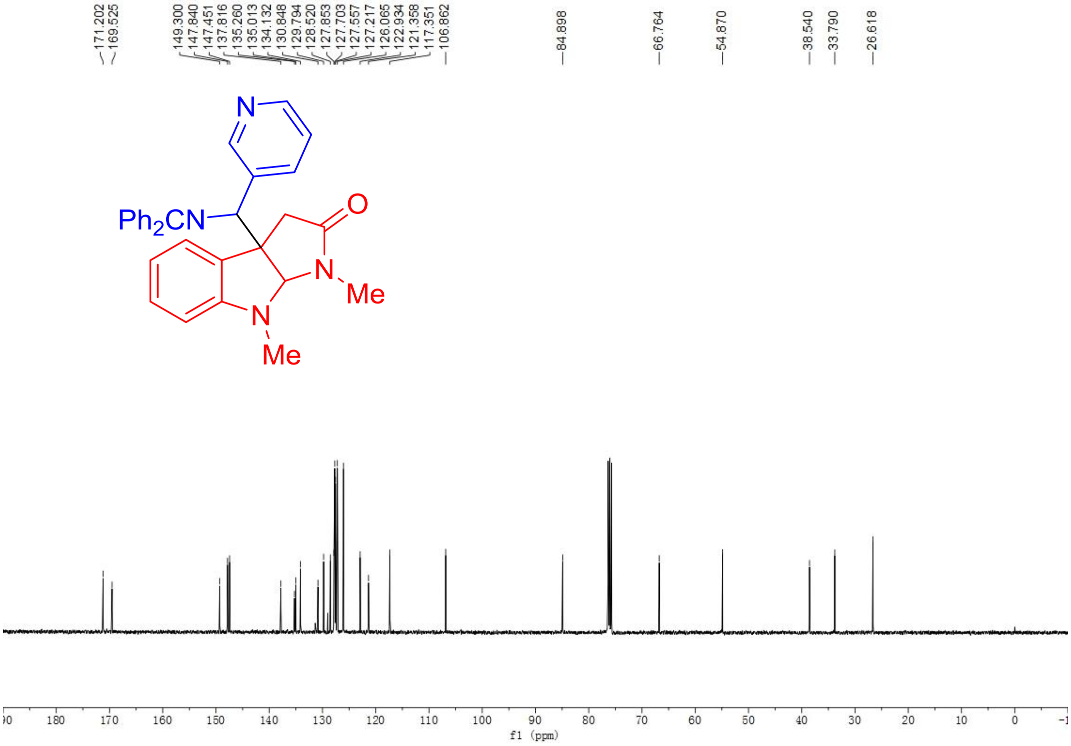

Figure S49. ^{1}H **NMR spectra (400 MHz, Chloroform-*d*) of 3a-(((diphenylmethylene)amino)(pyridin-3-yl)methyl)-1,8-dimethyl-3,3a,8,8a-tetrahydropyrrolo[2,3-*b*]indol-2(1*H*)-one (3ka(minor))**

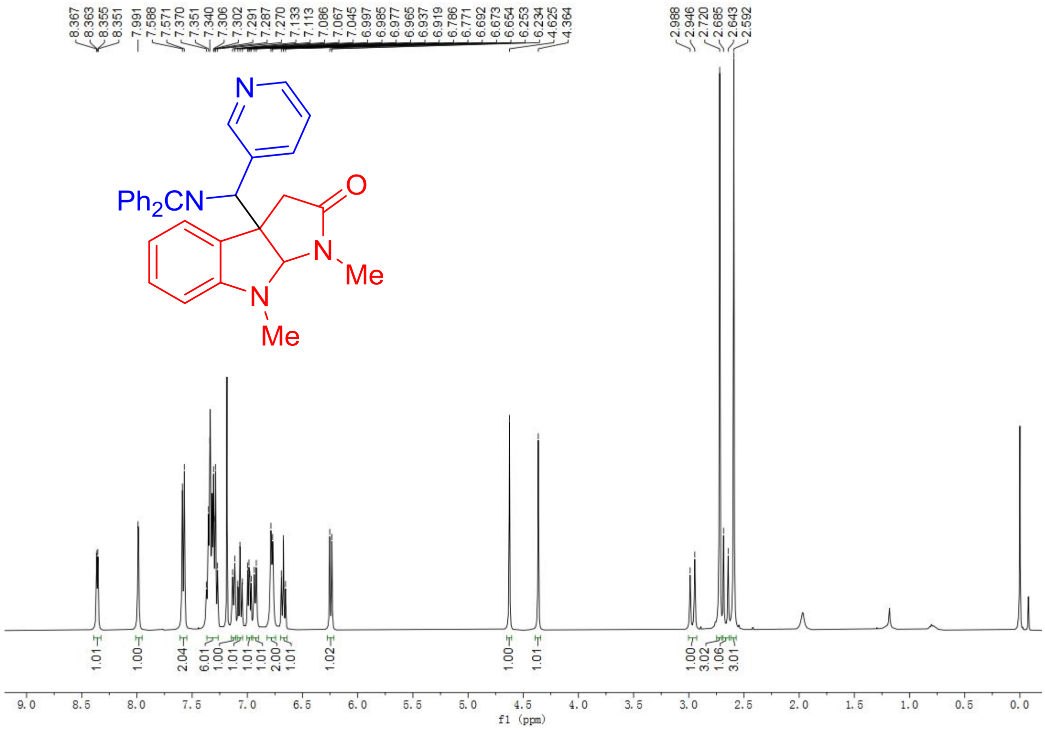

Figure S50. $^{13}C\{^{1}H\}$ **NMR spectra (100 MHz, Chloroform-*d*) of 3a-(((diphenylmethylene)amino)(pyridin-3-yl)methyl)-1,8-dimethyl-3,3a,8,8a-tetrahydropyrrolo[2,3-*b*]indol-2(1*H*)-one (3ka(minor))**

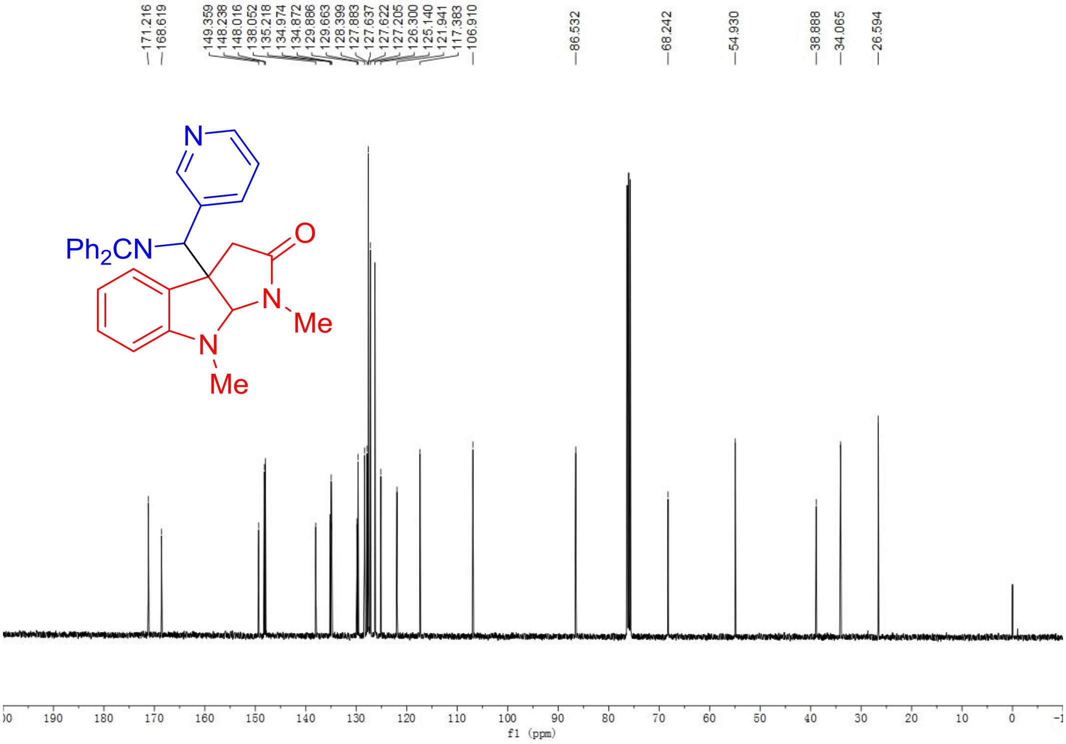

Figure S51. ^{1}**H NMR spectra (400 MHz, Chloroform-*d*) of 3a-(((diphenylmethylene)amino)(furan-2-yl)methyl)-1,8-dimethyl-3,3a,8,8a-tetrahydropyrrolo[2,3-*b*]indol-2(1*H*)-one (3la,** dr = 1.5:1)

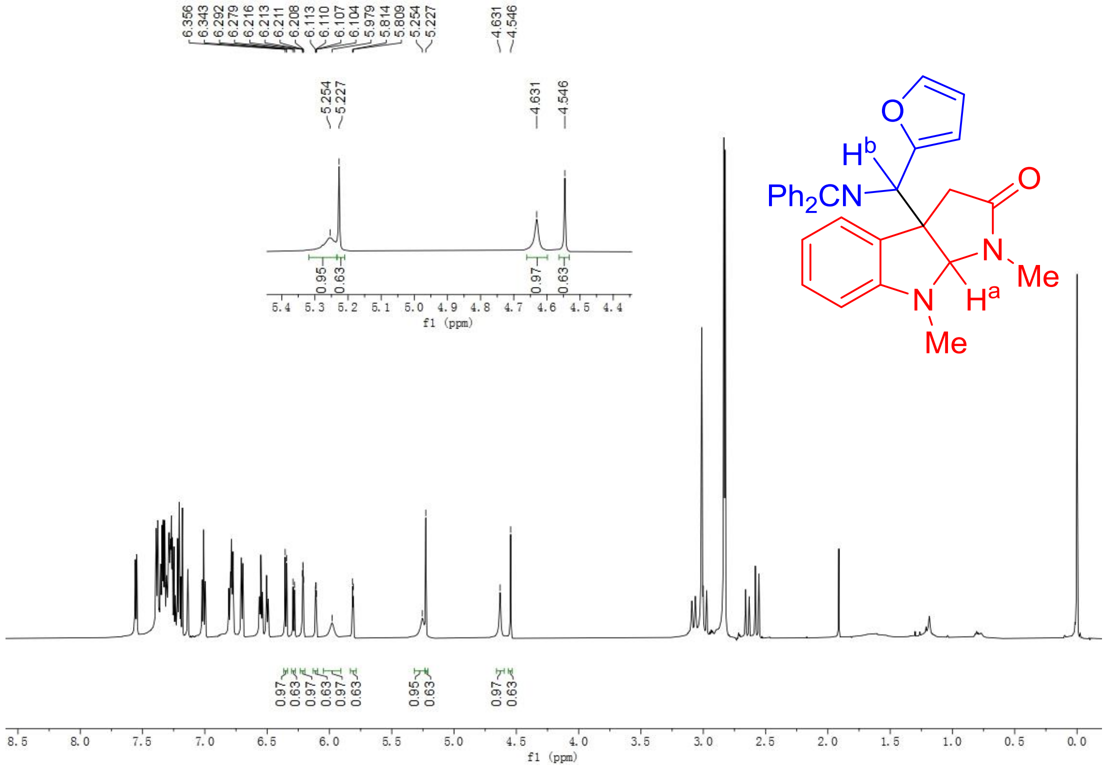

Figure S52. ^{13}**C{**^{1}**H} NMR spectra (100 MHz, Chloroform-*d*) of 3a-(((diphenylmethylene)amino)(furan-2-yl)methyl)-1,8-dimethyl-3,3a,8,8a-tetrahydropyrrolo[2,3-*b*]indol-2(1*H*)-one (3la,** dr = 1.5:1)

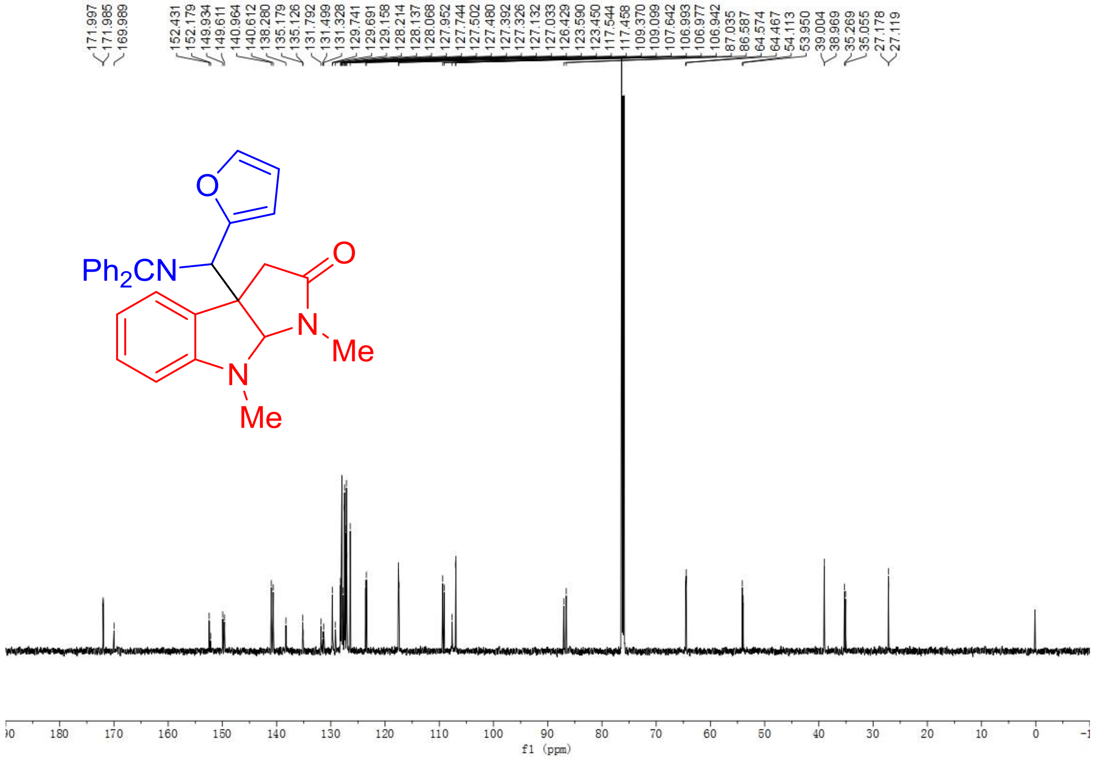

Figure S53. ^{1}H NMR spectra (600 MHz, Methanol-d_4) of 3a-(((diphenylmethylene)amino)(thiophen-2-yl)methyl)-1,8-dimethyl-3,3a,8,8a-tetrahydropyrrolo[2,3-*b*]indol-2(1*H*)-one (3ma(major))

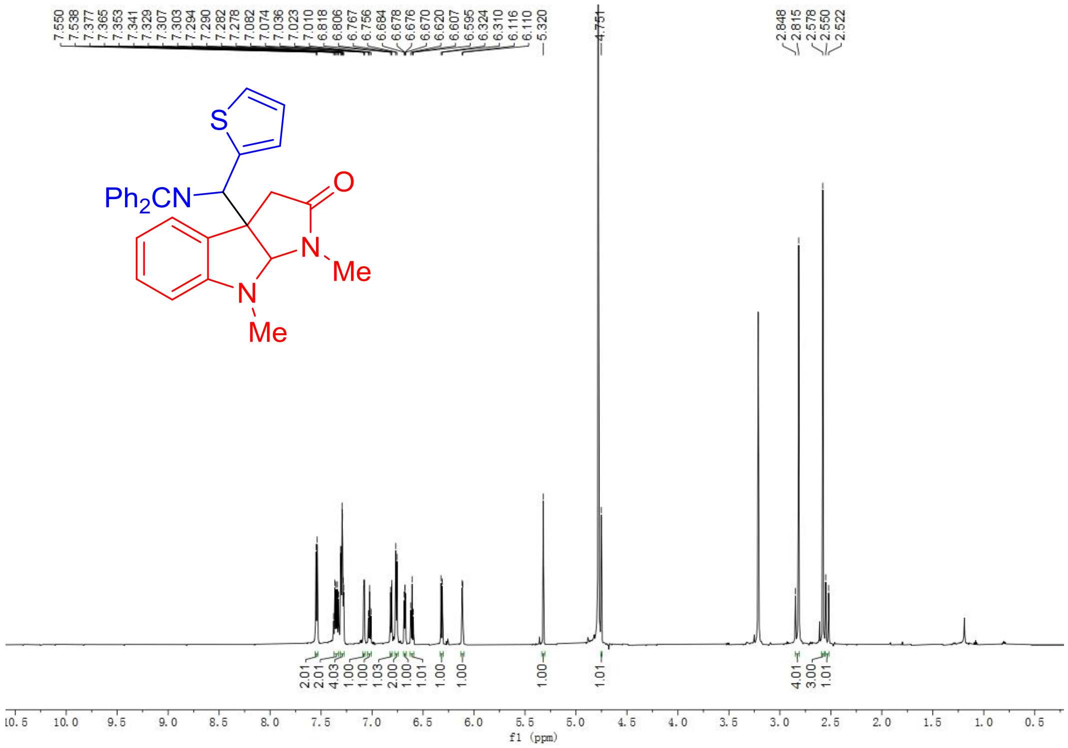

Figure S54. ^{13}C{^{1}H} NMR spectra (150 MHz, Methanol-d_4) of 3a-(((diphenylmethylene)amino)(thiophen-2-yl)methyl)-1,8-dimethyl-3,3a,8,8a-tetrahydropyrrolo[2,3-*b*]indol-2(1*H*)-one (3ma(major))

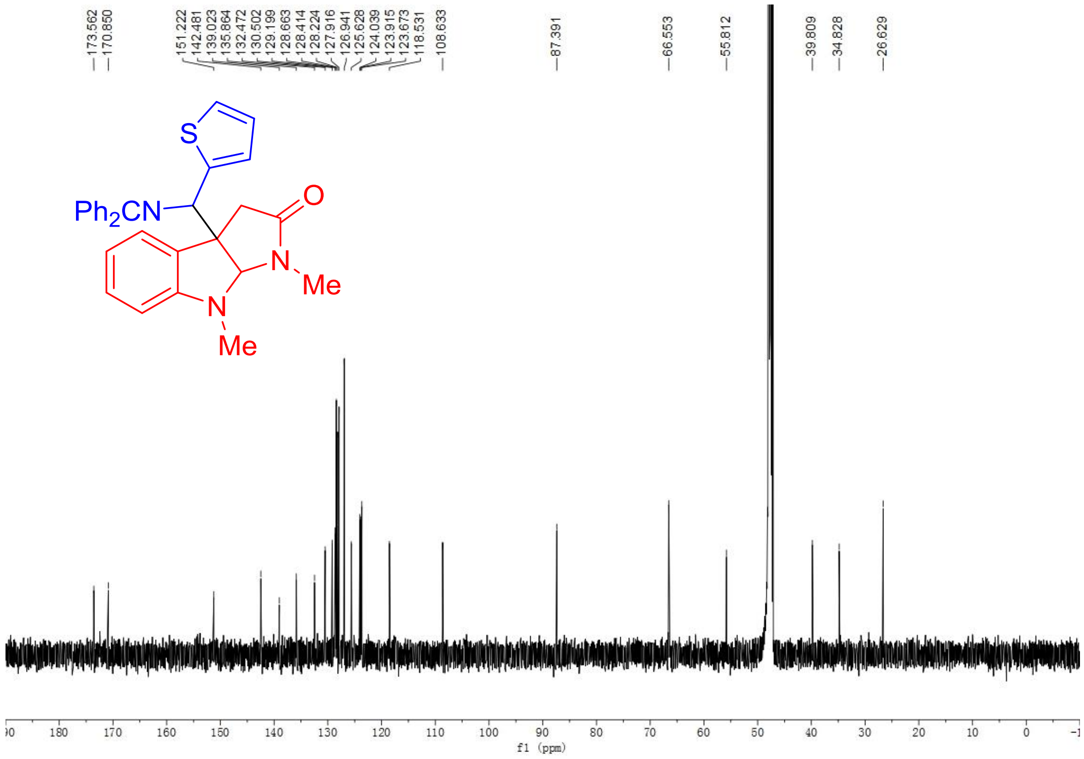

Figure S55. ^{1}H NMR spectra (600 MHz, Chloroform-*d*) of 3a-(((diphenylmethylene)amino)(thiophen-2-yl)methyl)-1,8-dimethyl-3,3a,8,8a-tetrahydropyrrolo[2,3-*b*]indol-2(1*H*)-one (3ma(minor))

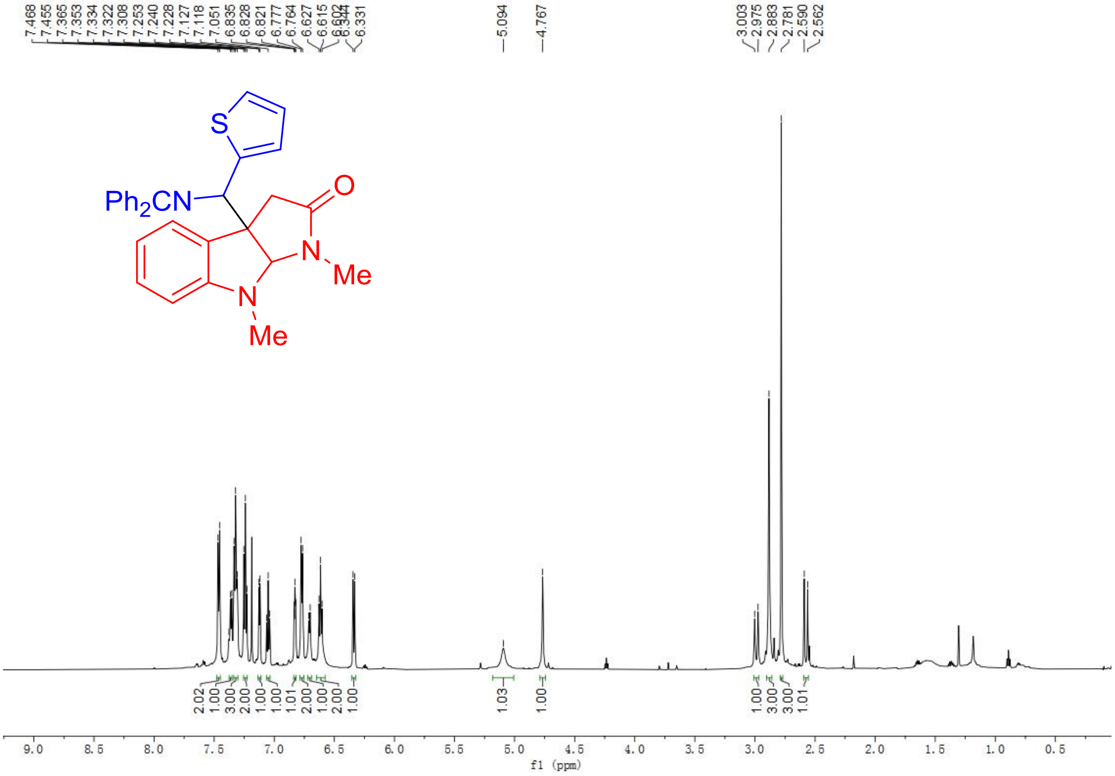

Figure S56. ^{13}C{^{1}H} NMR spectra (150 MHz, Chloroform-*d*) of 3a-(((diphenylmethylene)amino)(thiophen-2-yl)methyl)-1,8-dimethyl-3,3a,8,8a-tetrahydropyrrolo[2,3-*b*]indol-2(1*H*)-one (3ma(minor))

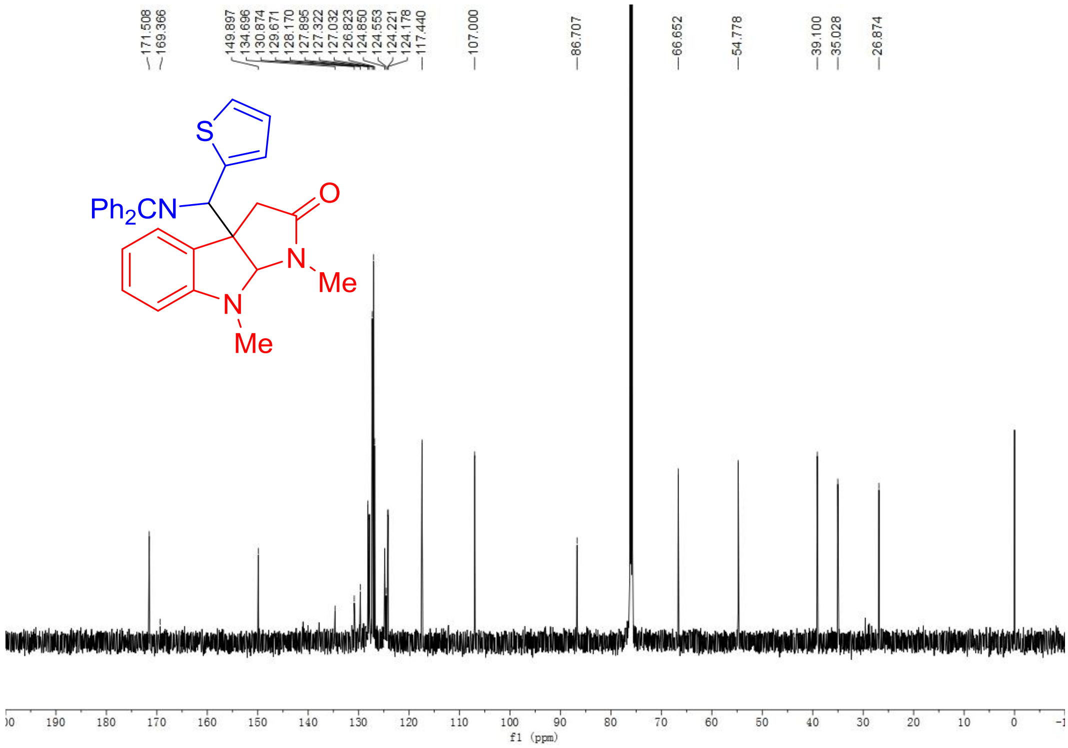

Figure S57. ^{1}H **NMR spectra (400 MHz, Chloroform-*d*) of 3a-(1-((9*H*-fluoren-9-ylidene)amino)ethyl)-1,8-dimethyl-3,3a,8,8a-tetrahydropyrrolo[2,3-*b*]indol-2(1*H*)-one (3na(major))**

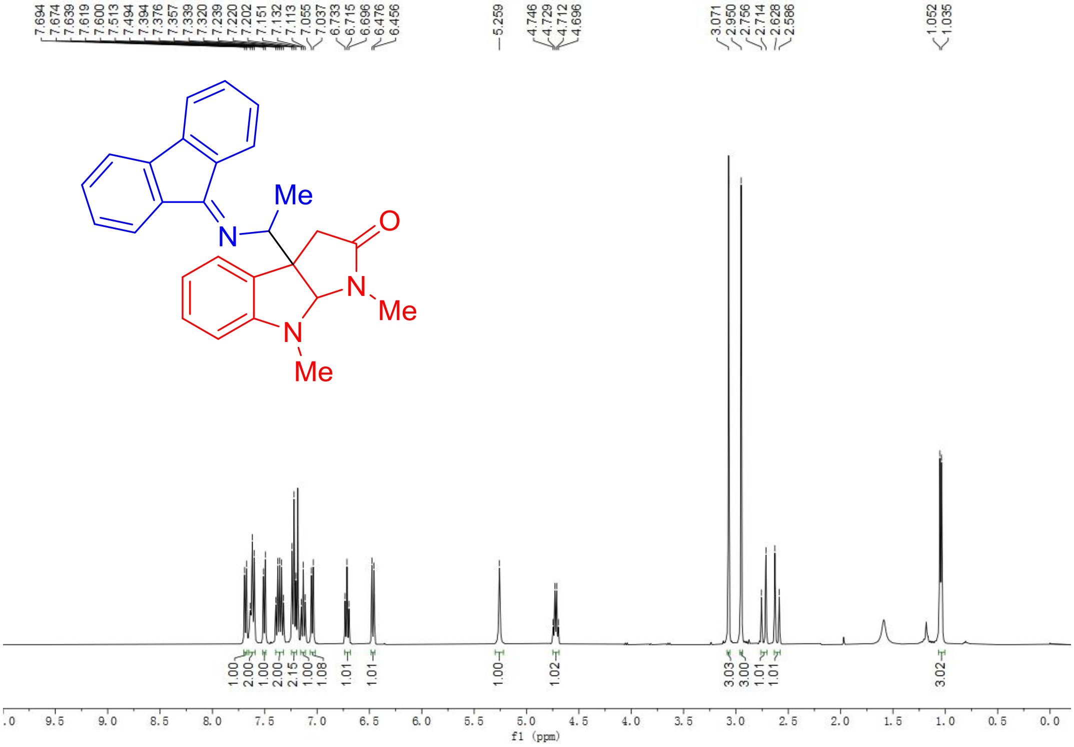

Figure S58. $^{13}C\{^{1}H\}$ **NMR spectra (100 MHz, Chloroform-*d*) of 3a-(1-((9*H*-fluoren-9-ylidene)amino)ethyl)-1,8-dimethyl-3,3a,8,8a-tetrahydropyrrolo[2,3-*b*]indol-2(1*H*)-one (3na(major))**

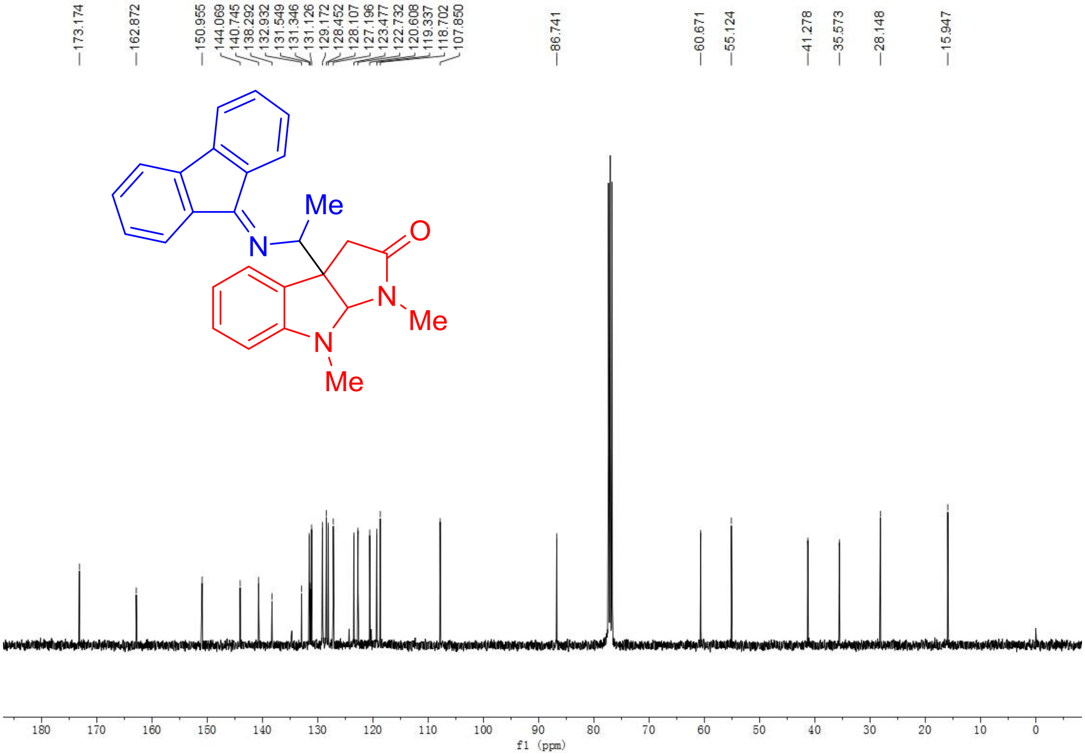

Figure S59. ^{1}H NMR spectra (600 MHz, Chloroform-*d*) of 3a-(1-((9*H*-fluoren-9-ylidene)amino)ethyl)-1,8-dimethyl-3,3a,8,8a-tetrahydropyrrolo[2,3-*b*]indol-2(1*H*)-one (3na(minor))

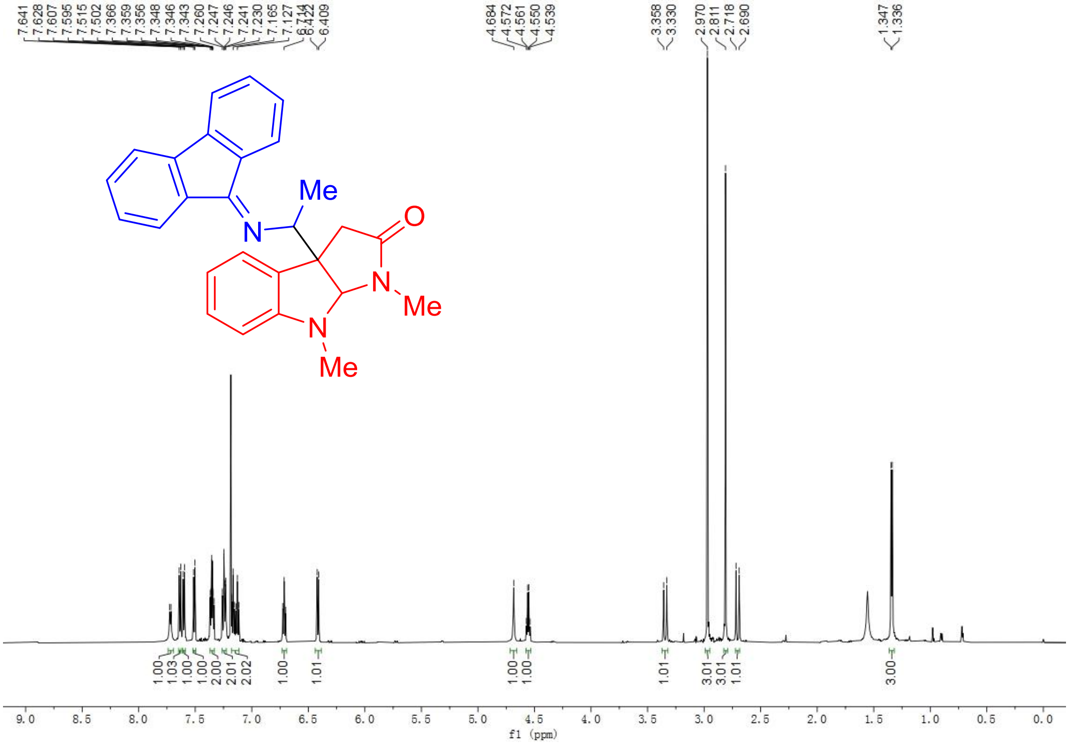

Figure S60. ^{13}C{^{1}H} NMR spectra (150 MHz, Chloroform-*d*) of 3a-(1-((9*H*-fluoren-9-ylidene)amino)ethyl)-1,8-dimethyl-3,3a,8,8a-tetrahydropyrrolo[2,3-*b*]indol-2(1*H*)-one (3na(minor))

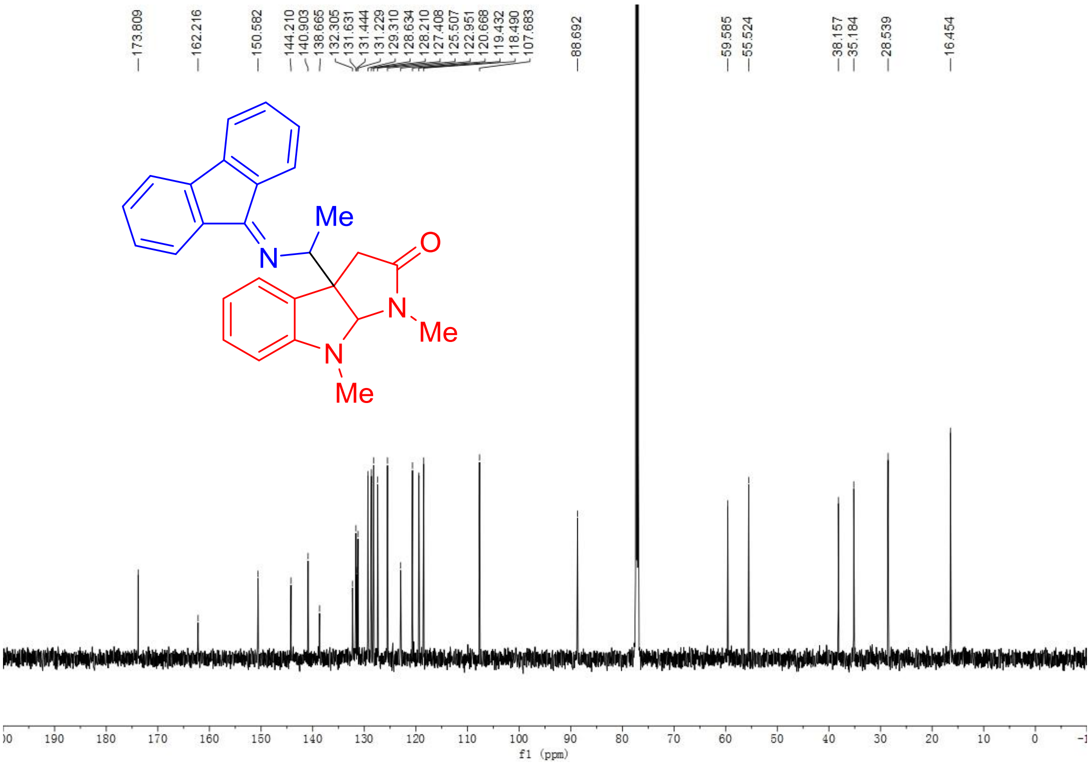

Figure S61. ^{1}**H NMR spectra (600 MHz, Methanol-*d*$_4$) of 3a-(1-((9*H*-fluoren-9-ylidene)amino)-2-methylpropyl)-1,8-dimethyl-3,3a,8,8a-tetrahydropyrrolo[2,3-*b*]indol-2(1*H*)-one (3oa(major))**

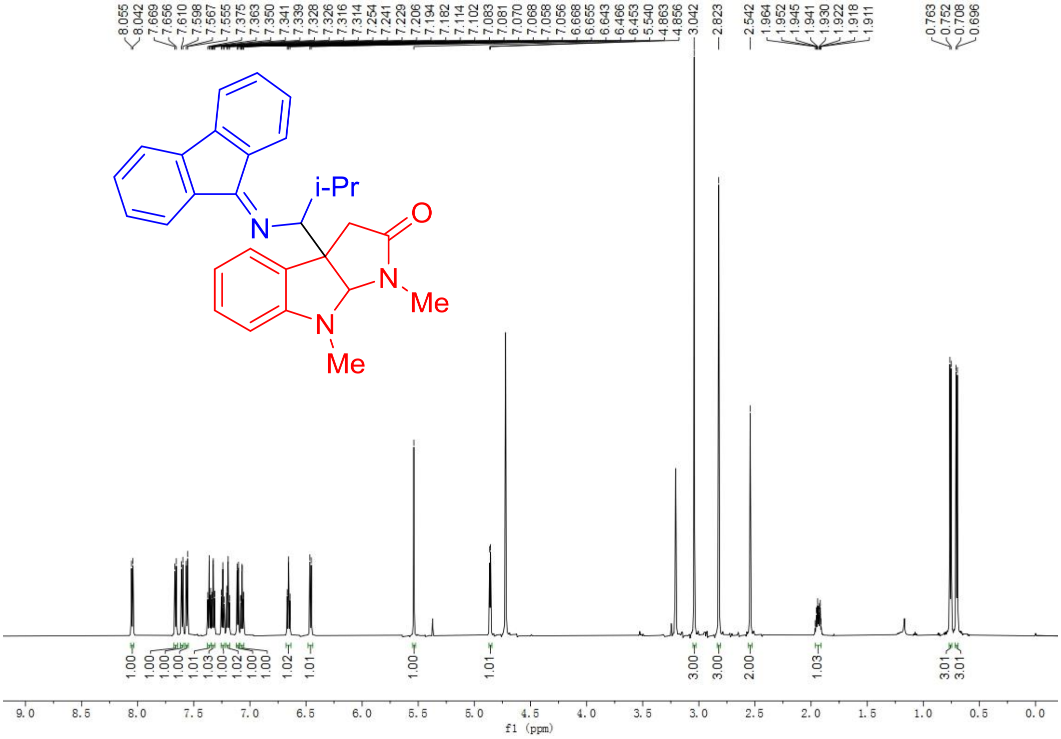

Figure S62. ^{13}**C{**^{1}**H} NMR spectra (150 MHz, Methanol-*d*$_4$) of 3a-(1-((9*H*-fluoren-9-ylidene)amino)-2-methylpropyl)-1,8-dimethyl-3,3a,8,8a-tetrahydropyrrolo[2,3-*b*]indol-2(1*H*)-one (3oa(major))**

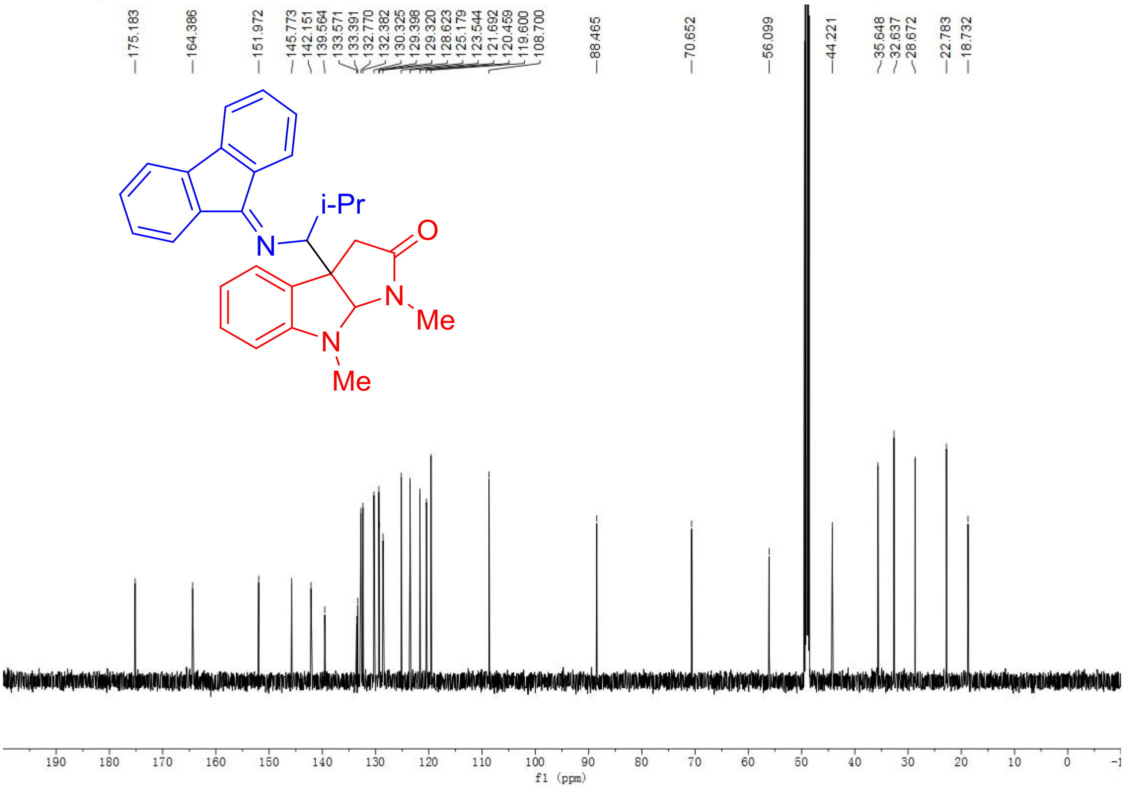

Figure S63. ^{1}H NMR spectra (600 MHz, Methanol-d_4) of 3a-(1-((9*H*-fluoren-9-ylidene)amino)-2-methylpropyl)-1,8-dimethyl-3,3a,8,8a-tetrahydropyrrolo[2,3-*b*]indol-2(1*H*)-one (3oa(minor))

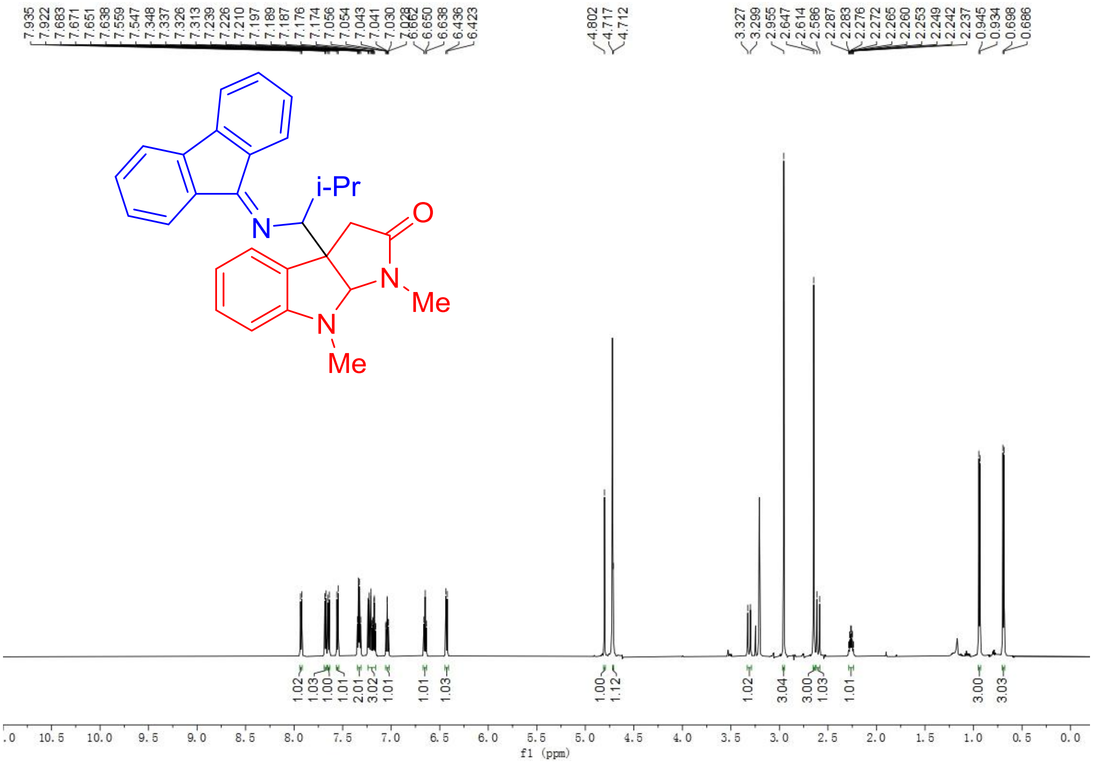

Figure S64. ^{13}C{^{1}H} NMR spectra (150 MHz, Methanol-d_4) of 3a-(1-((9*H*-fluoren-9-ylidene)amino)-2-methylpropyl)-1,8-dimethyl-3,3a,8,8a-tetrahydropyrrolo[2,3-*b*]indol-2(1*H*)-one (3oa(minor))

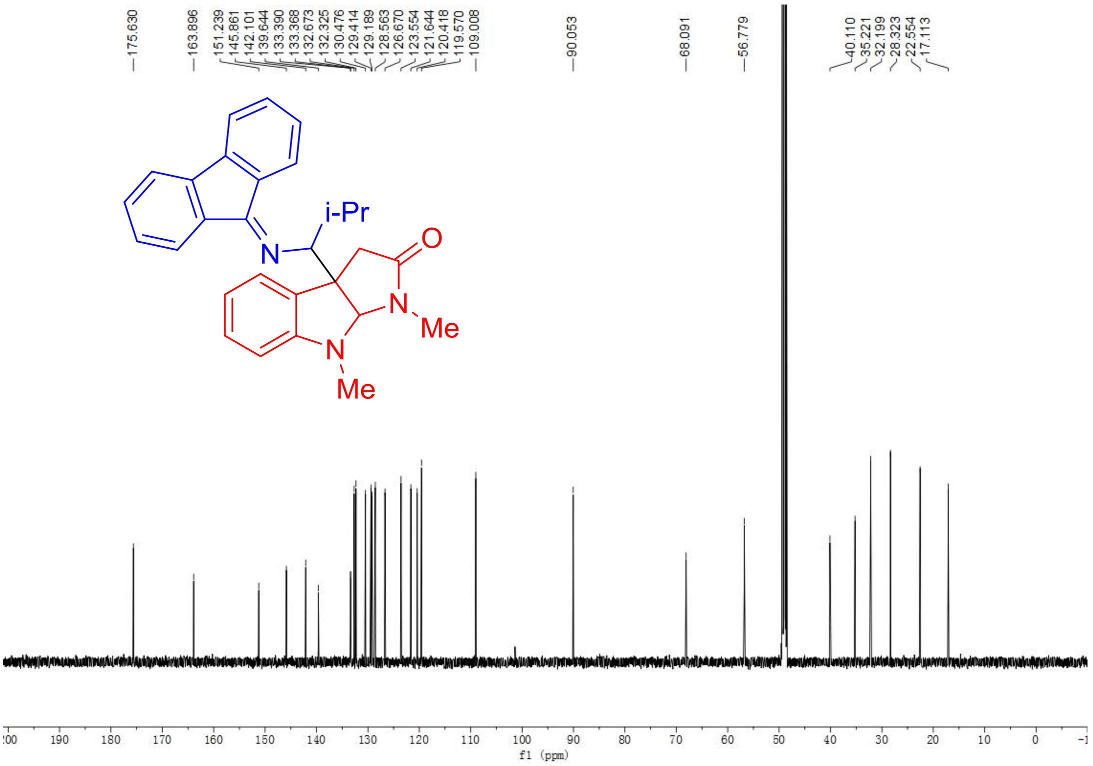

Figure S65. [1]**H NMR spectra (400 MHz, Chloroform-*d*) of 3a-(1-((9*H*-fluoren-9-ylidene)amino)-3-methylbutyl)-1,8-dimethyl-3,3a,8,8a-tetrahydropyrrolo[2,3-*b*]indol-2(1*H*)-one (3pa, dr = 1.5:1)**

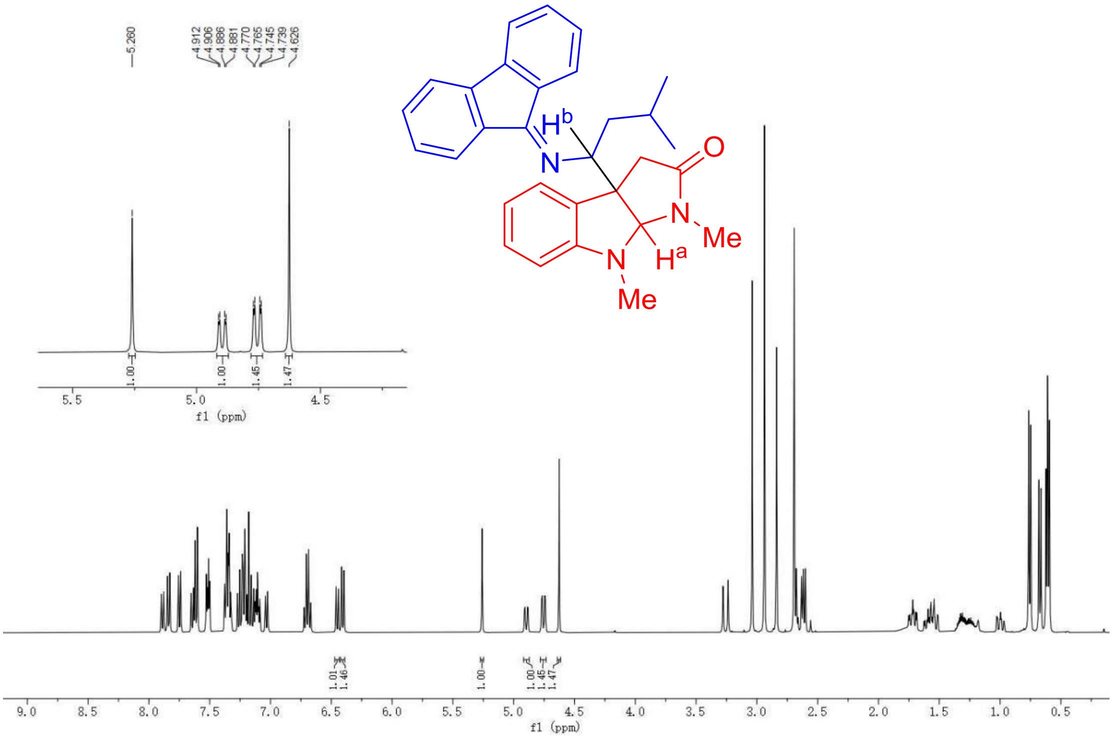

Figure S66. [13]**C{**[1]**H} NMR spectra (100 MHz, Chloroform-*d*) of 3a-(1-((9*H*-fluoren-9-ylidene)amino)-3-methylbutyl)-1,8-dimethyl-3,3a,8,8a-tetrahydropyrrolo[2,3-*b*]indol-2(1*H*)-one (3pa, dr = 1.5:1)**

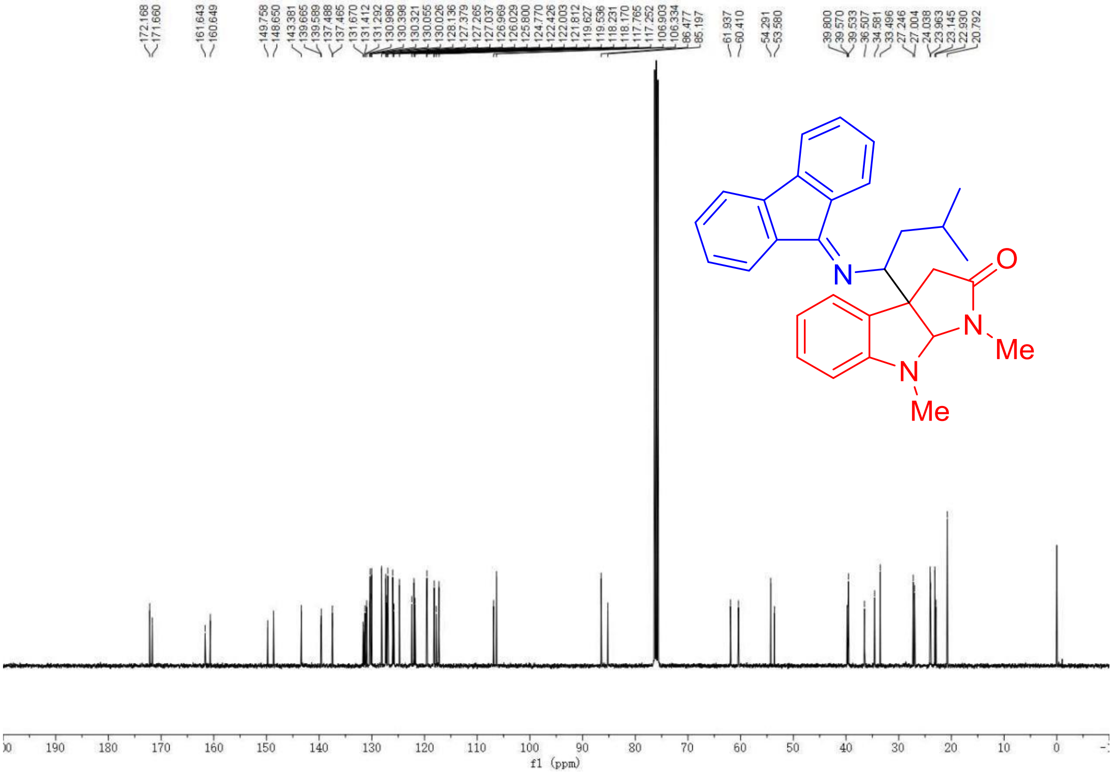

Figure S67. ^{1}H NMR spectra (400 MHz, Chloroform-*d*) of 3a-(((9*H*-fluoren-9-ylidene)amino)(cyclobutyl)methyl)-1,8-dimethyl-3,3a,8,8a-tetrahydropyrrolo[2,3-*b*]indol-2(1*H*)-one (3qa(major))

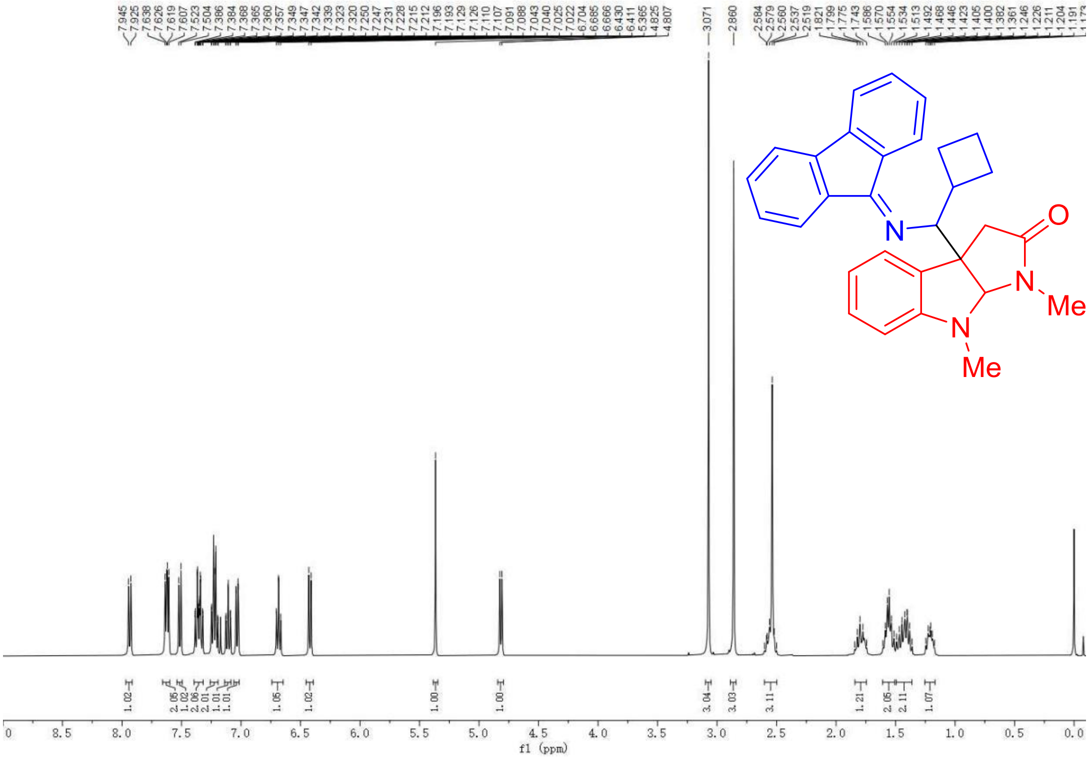

Figure S68. $^{13}C\{^{1}H\}$ NMR spectra (100 MHz, Chloroform-*d*) of 3a-(((9*H*-fluoren-9-ylidene)amino)(cyclobutyl)methyl)-1,8-dimethyl-3,3a,8,8a-tetrahydropyrrolo[2,3-*b*]indol-2(1*H*)-one (3qa(major))

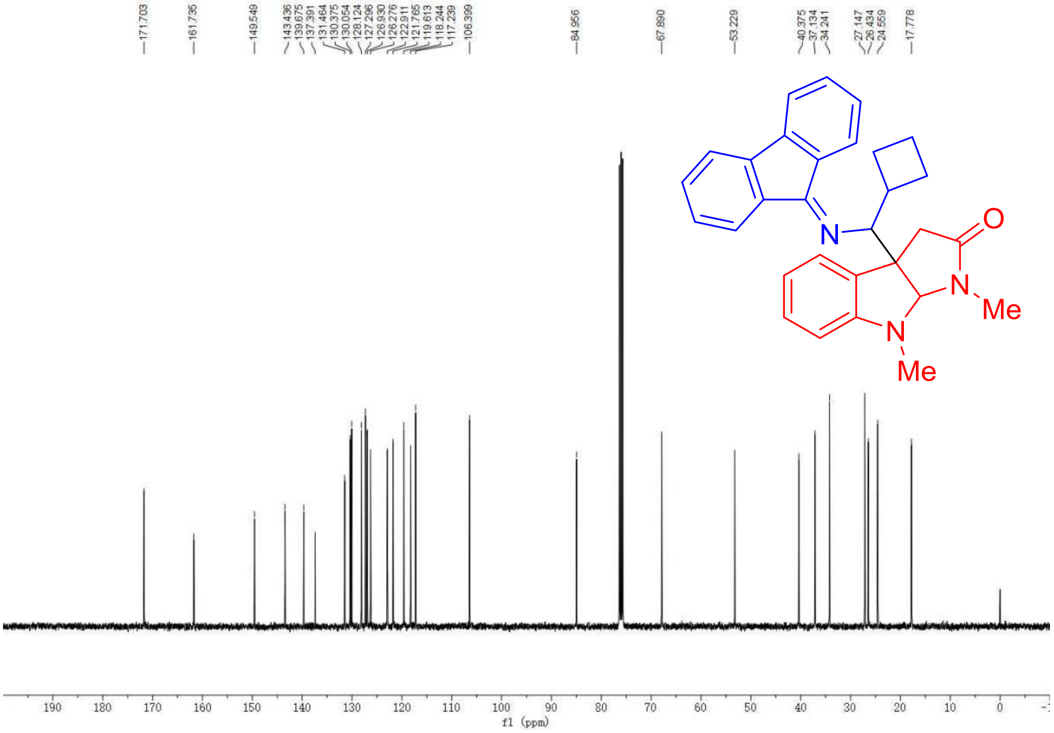

Figure S69. ^{1}H NMR spectra (400 MHz, Chloroform-*d*) of 3a-(((9*H*-fluoren-9-ylidene)amino)(cyclobutyl)methyl)-1,8-dimethyl-3,3a,8,8a-tetrahydropyrrolo[2,3-*b*]indol-2(1*H*)-one (3qa(minor))

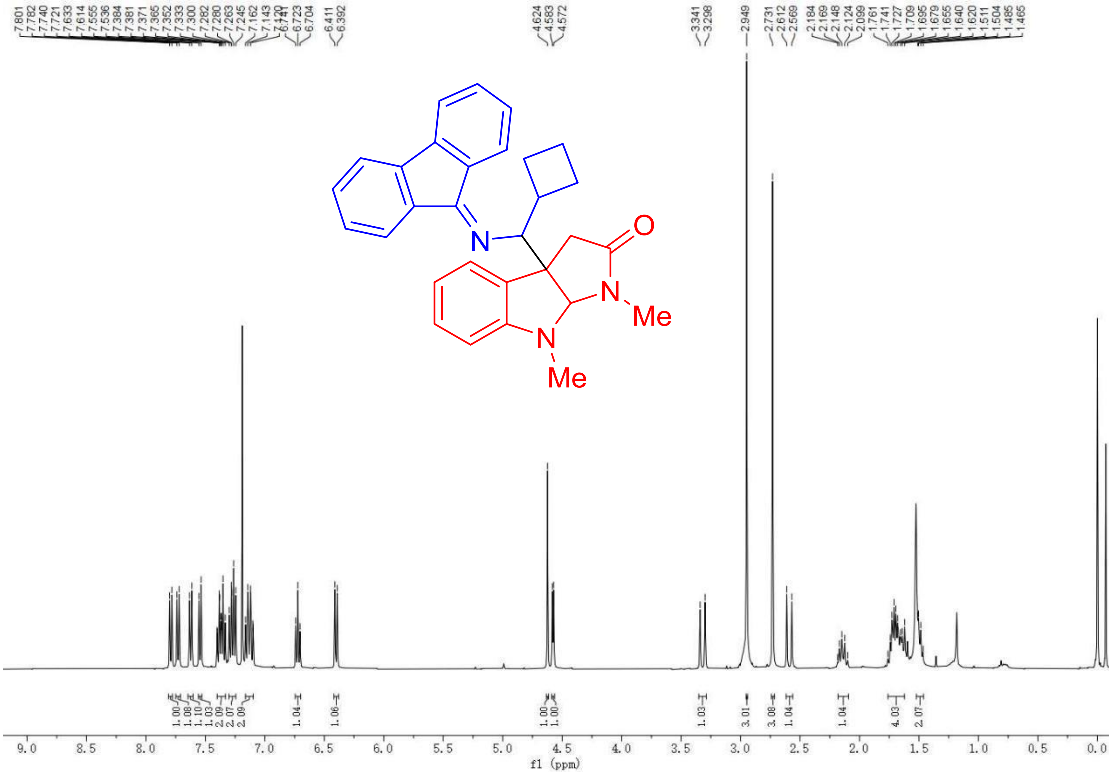

Figure S70. ^{13}C{^{1}H} NMR spectra (100 MHz, Chloroform-*d*) of 3a-(((9*H*-fluoren-9-ylidene)amino)(cyclobutyl)methyl)-1,8-dimethyl-3,3a,8,8a-tetrahydropyrrolo[2,3-*b*]indol-2(1*H*)-one (3qa(minor))

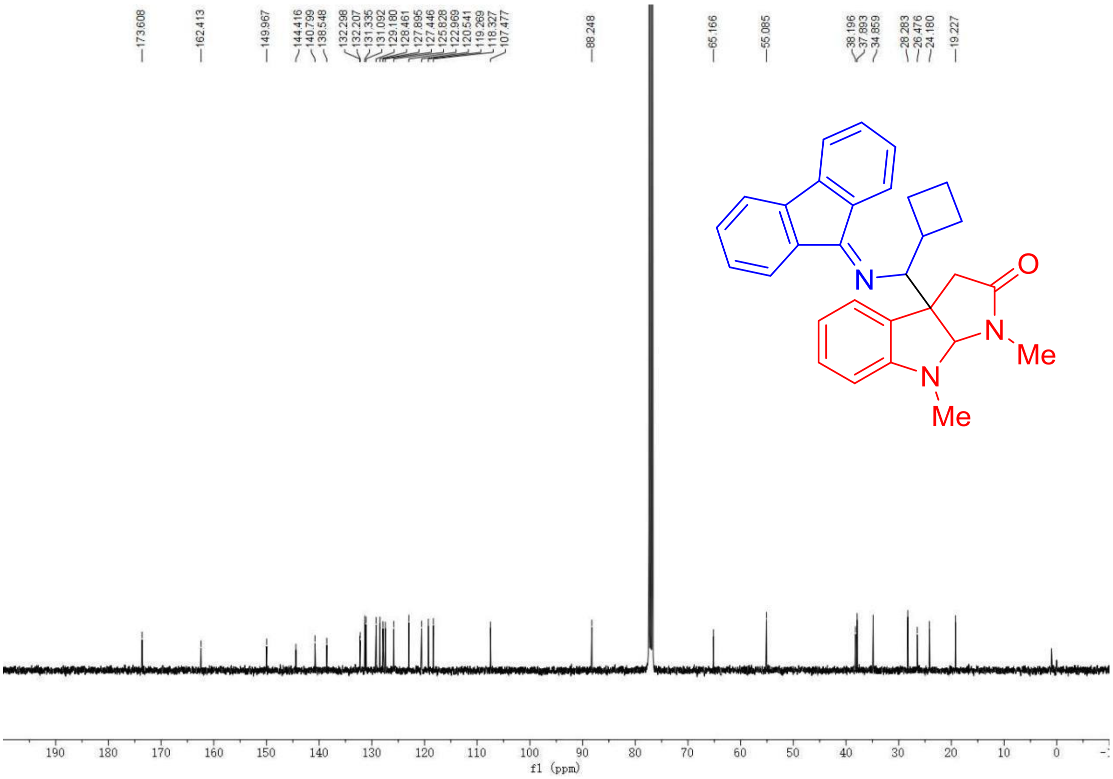

Figure S71. ^{1}H **NMR spectra (400 MHz, Chloroform-*d*) of 3a-(((9*H*-fluoren-9-ylidene)amino)(cyclopentyl)methyl)-1,8-dimethyl-3,3a,8,8a-tetrahydropyrrolo[2,3-*b*]indol-2(1*H*)-one (3ra(major))**

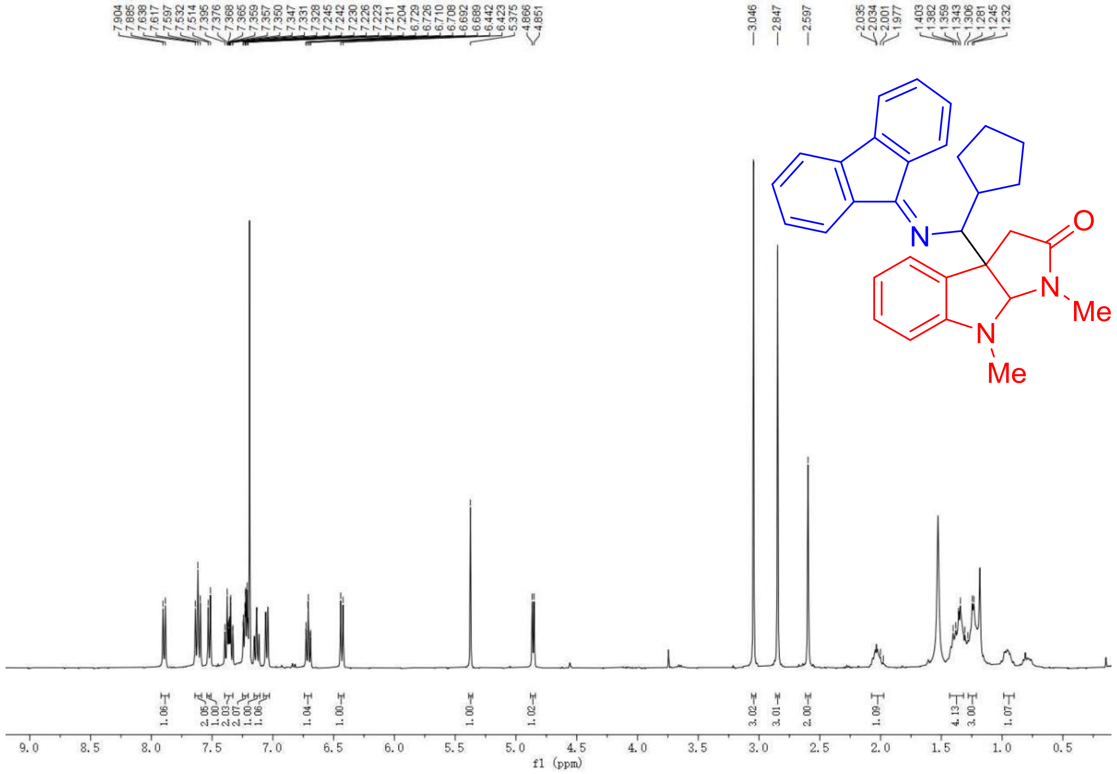

Figure S72. $^{13}C\{^{1}H\}$ **NMR spectra (100 MHz, Chloroform-*d*) of 3a-(((9*H*-fluoren-9-ylidene)amino)(cyclopentyl)methyl)-1,8-dimethyl-3,3a,8,8a-tetrahydropyrrolo[2,3-*b*]indol-2(1*H*)-one (3ra(major))**

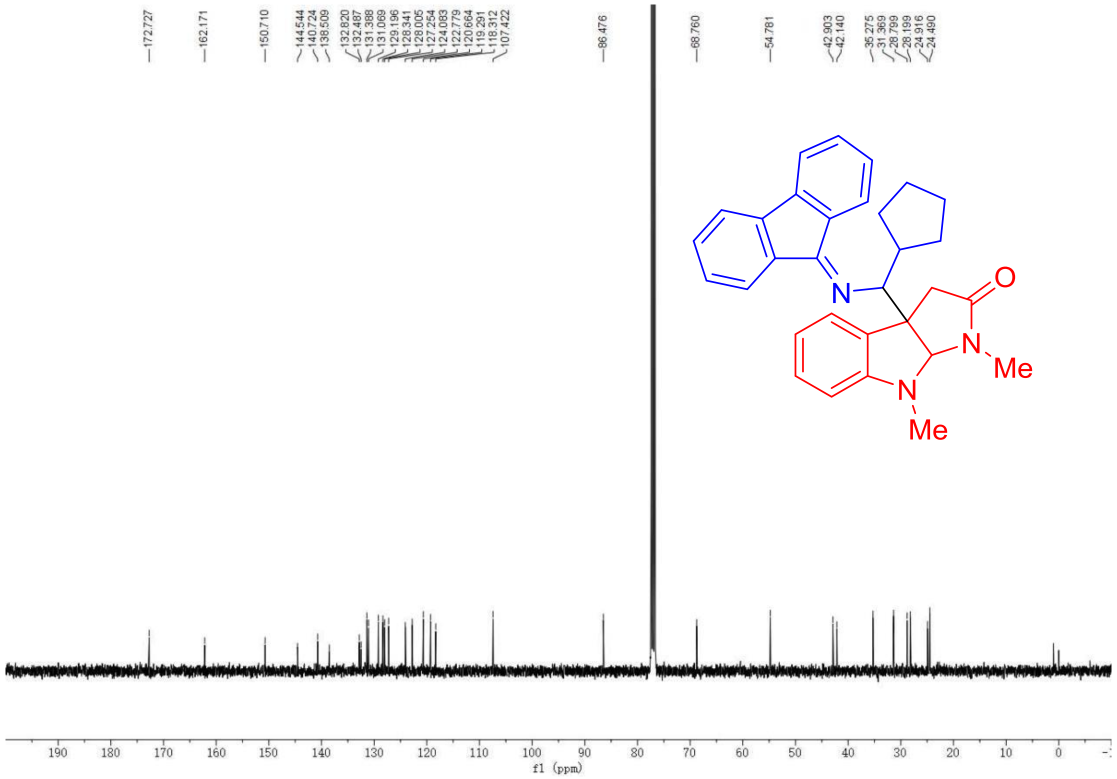

Figure S73. ^{1}H NMR spectra (400 MHz, Chloroform-*d*) of 3a-(((9*H*-fluoren-9-ylidene)amino)(cyclopentyl)methyl)-1,8-dimethyl-3,3a,8,8a-tetrahydropyrrolo[2,3-*b*]indol-2(1*H*)-one (3ra(minor))

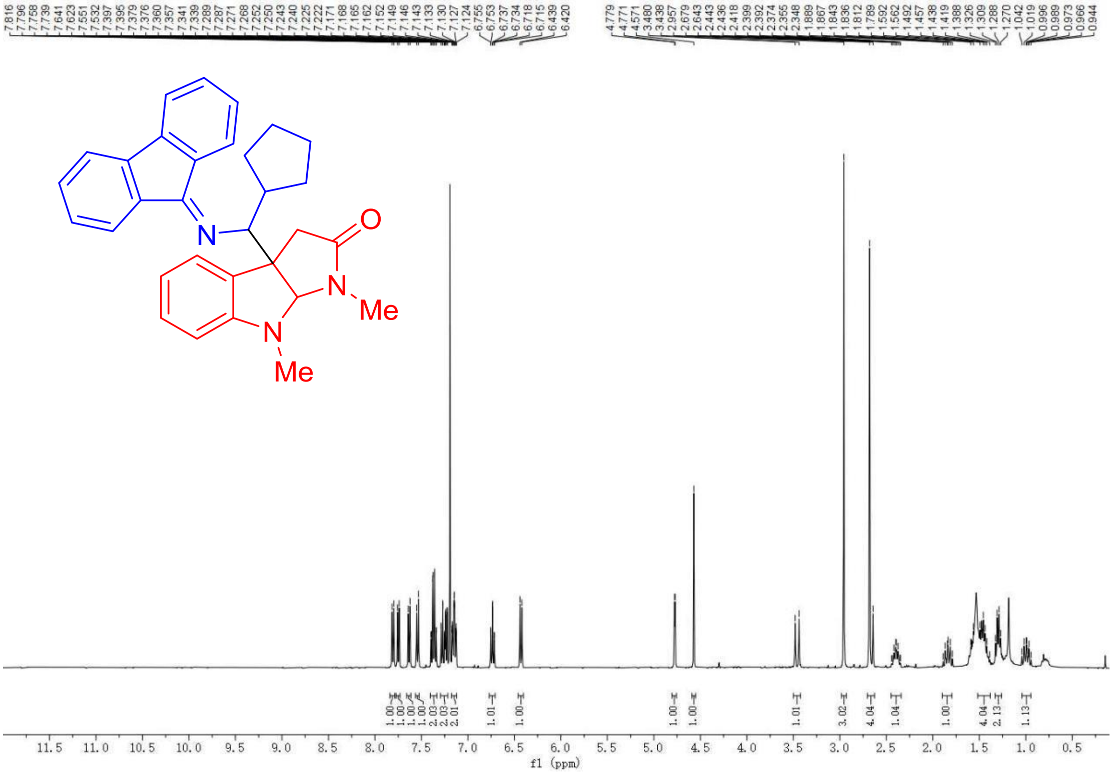

Figure S74. ^{13}C{^{1}H} NMR spectra (100 MHz, Chloroform-*d*) of 3a-(((9*H*-fluoren-9-ylidene)amino)(cyclopentyl)methyl)-1,8-dimethyl-3,3a,8,8a-tetrahydropyrrolo[2,3-*b*]indol-2(1*H*)-one (3ra(minor))

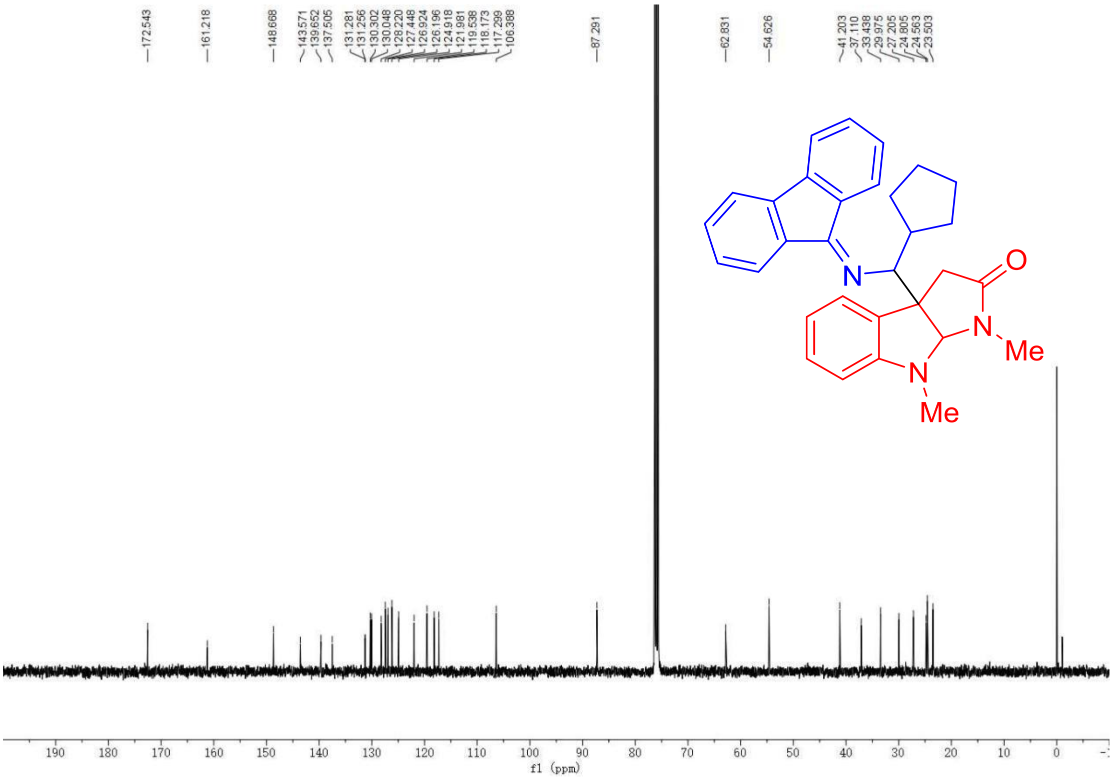

Figure S75. ^{1}H NMR spectra (400 MHz, Chloroform-*d*) of 3a-(((9*H*-fluoren-9-ylidene)amino)(cyclohexyl)methyl)-1,8-dimethyl-3,3a,8,8a-tetrahydropyrrolo[2,3-*b*]indol-2(1*H*)-one (3sa(major))

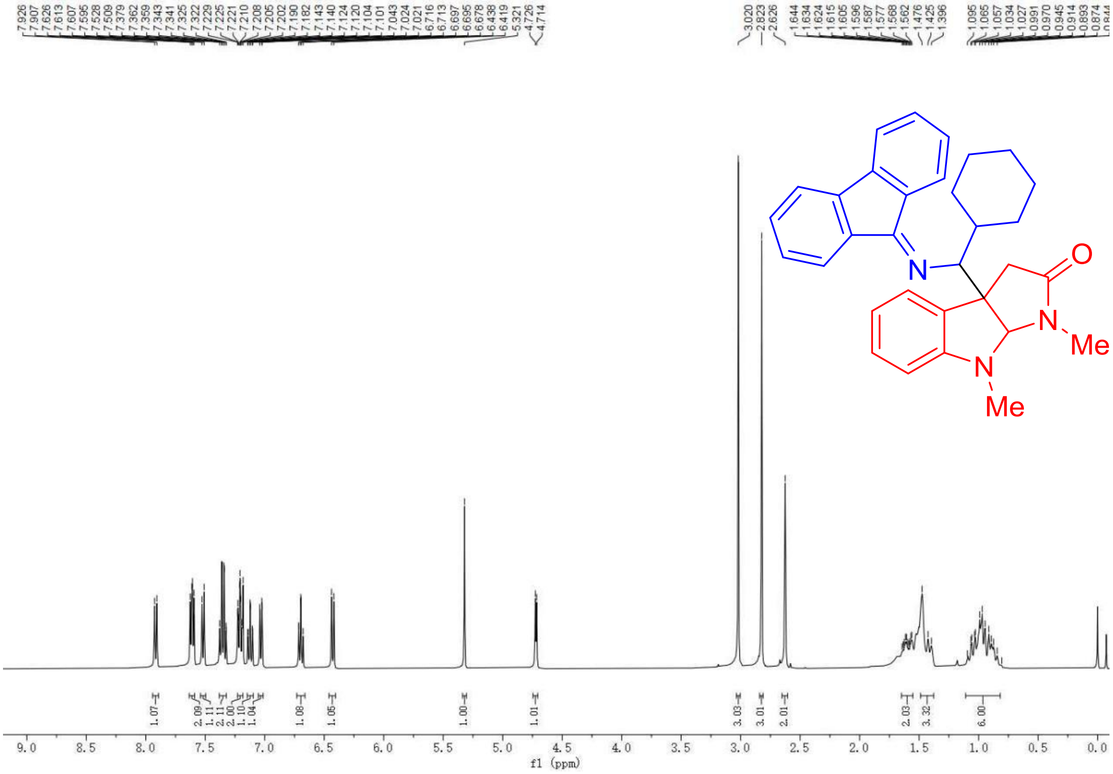

Figure S76. ^{13}C{^{1}H} NMR spectra (100 MHz, Chloroform-*d*) of 3a-(((9*H*-fluoren-9-ylidene)amino)(cyclohexyl)methyl)-1,8-dimethyl-3,3a,8,8a-tetrahydropyrrolo[2,3-*b*]indol-2(1*H*)-one (3sa(major))

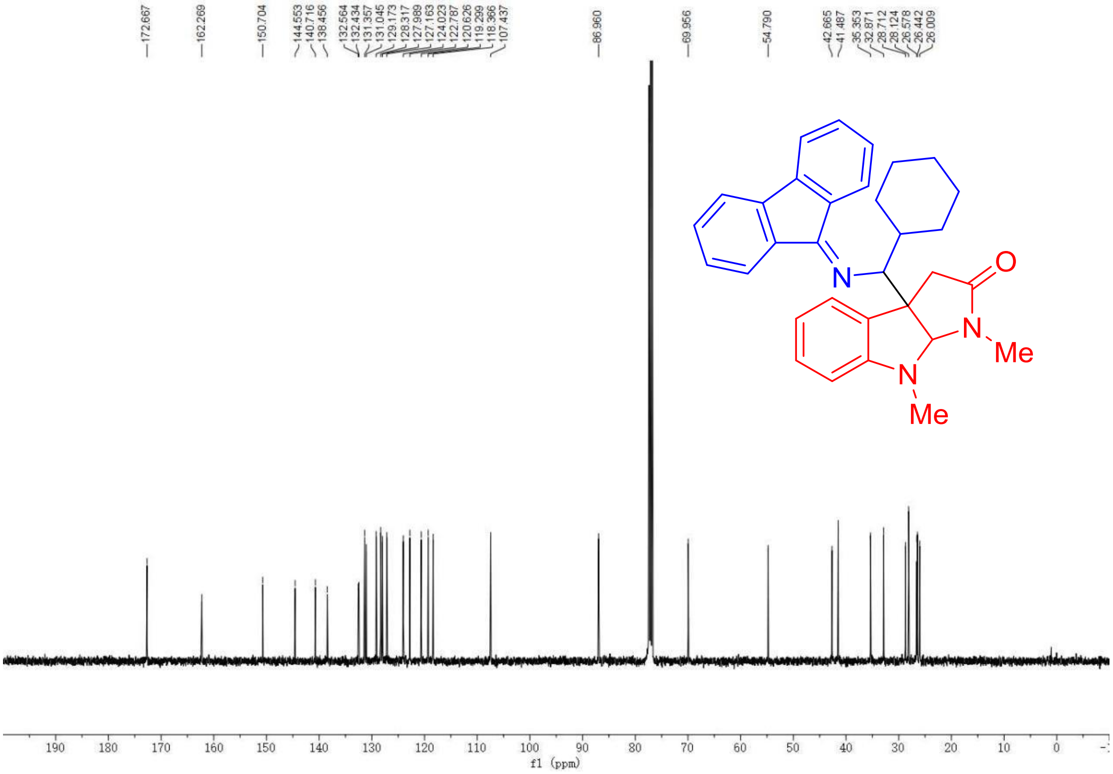

Figure S77. ^{1}**H NMR spectra (400 MHz, Chloroform-*d*) of 3a-(((9*H*-fluoren-9-ylidene)amino)(cyclohexyl)methyl)-1,8-dimethyl-3,3a,8,8a-tetrahydropyrrolo[2,3-*b*]indol-2(1*H*)-one (3sa(minor))**

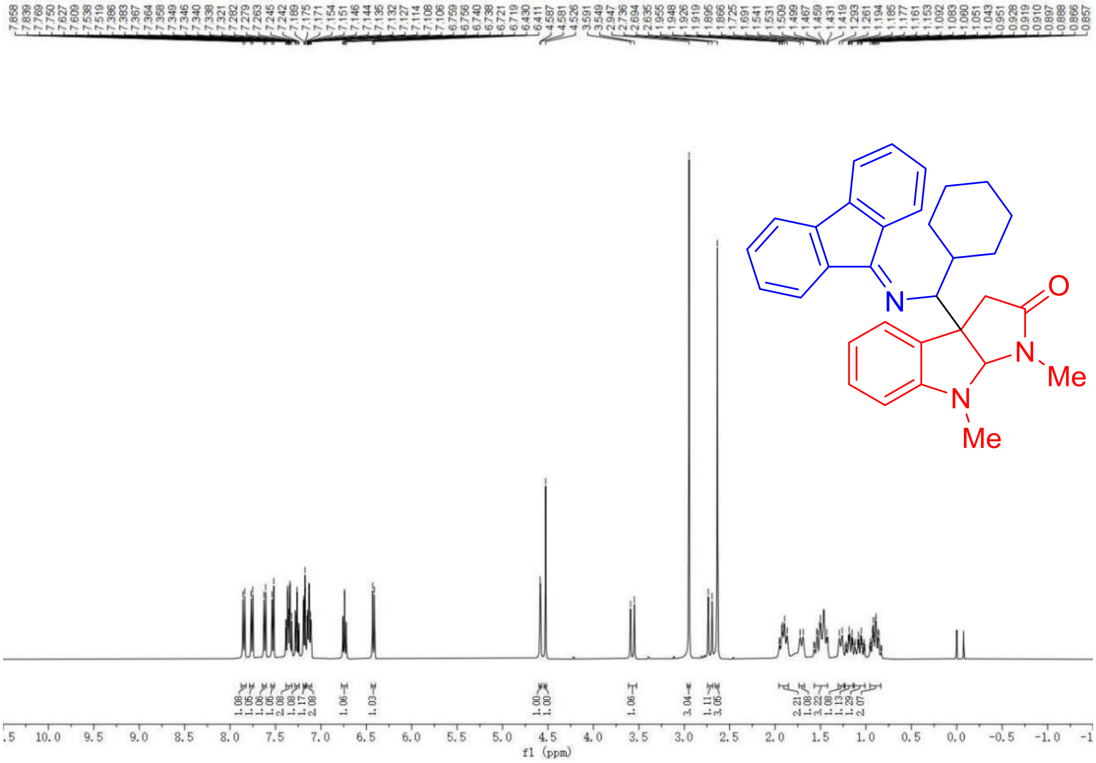

Figure S78. ^{13}**C{**^{1}**H} NMR spectra (100 MHz, Chloroform-*d*) of 3a-(((9*H*-fluoren-9-ylidene)amino)(cyclohexyl)methyl)-1,8-dimethyl-3,3a,8,8a-tetrahydropyrrolo[2,3-*b*]indol-2(1*H*)-one (3sa(minor))**

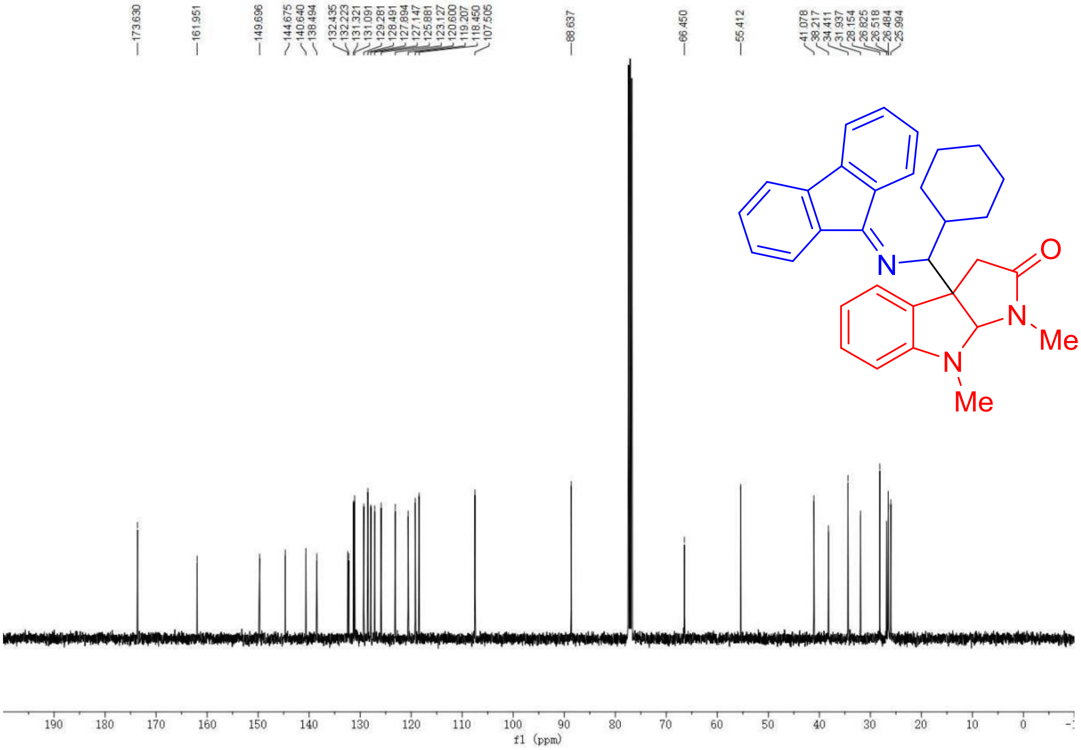

Figure S79. ^{1}H **NMR spectra (600 MHz, Chloroform-*d*) of 3a-(((diphenylmethylene)amino)(phenyl)methyl)-1,7,8-trimethyl-3,3a,8,8a-tetrahydropyrrolo[2,3-*b*]indol-2(1*H*)-one (3ab(major))**

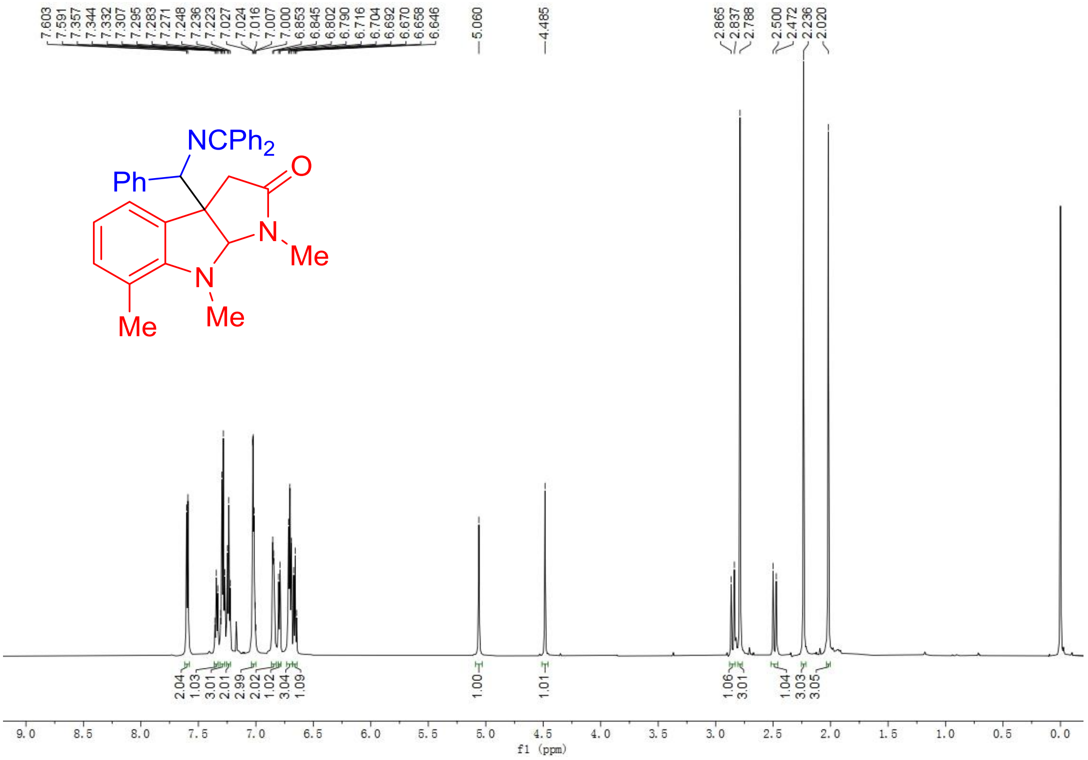

Figure S80. $^{13}C\{^{1}H\}$ **NMR spectra (150 MHz, Chloroform-*d*) of 3a-(((diphenylmethylene)amino)(phenyl)methyl)-1,7,8-trimethyl-3,3a,8,8a-tetrahydropyrrolo[2,3-*b*]indol-2(1*H*)-one (3ab(major))**

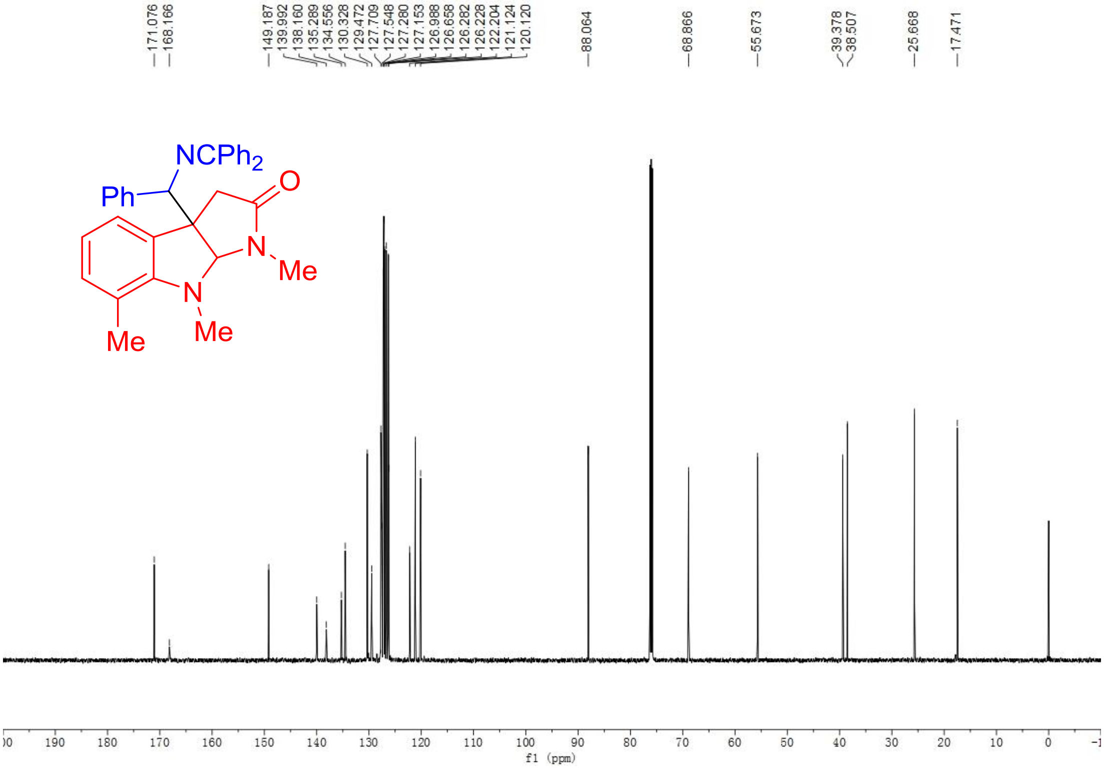

Figure S81. ^{1}H NMR spectra (600 MHz, Chloroform-*d*) of 3a-(((diphenylmethylene)amino)(phenyl)methyl)-1,7,8-trimethyl-3,3a,8,8a-tetrahydropyrrolo[2,3-*b*]indol-2(1*H*)-one (3ab(minor))

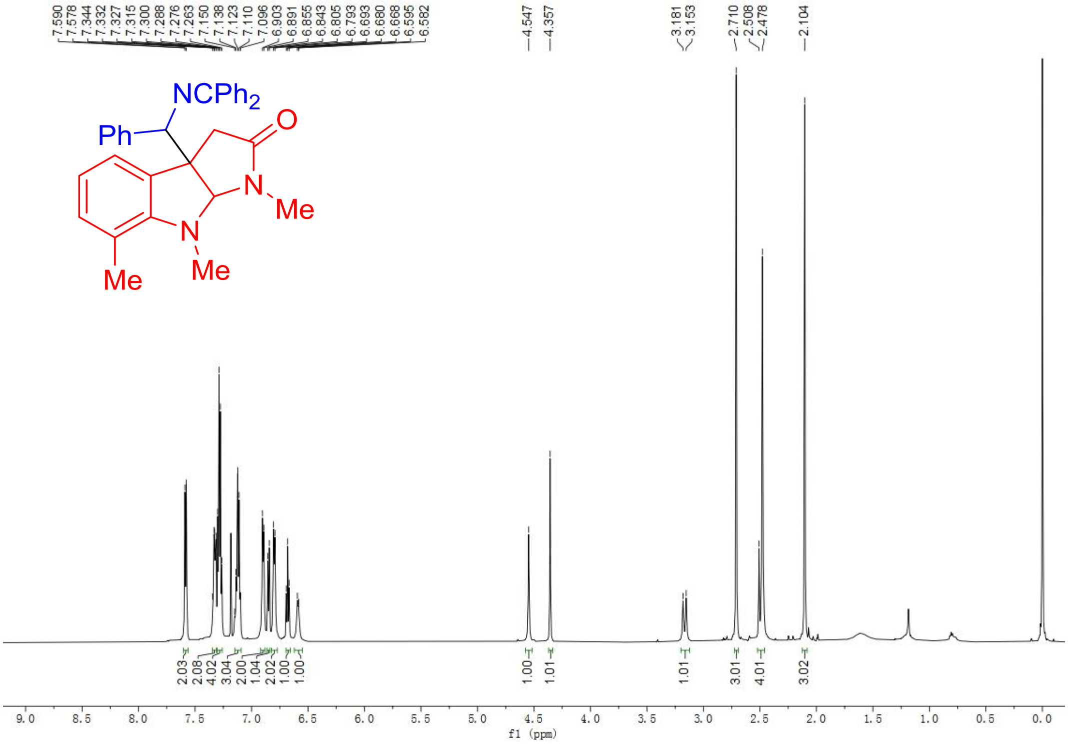

Figure S82. $^{13}C\{^{1}H\}$ NMR spectra (150 MHz, Chloroform-*d*) of 3a-(((diphenylmethylene)amino)(phenyl)methyl)-1,7,8-trimethyl-3,3a,8,8a-tetrahydropyrrolo[2,3-*b*]indol-2(1*H*)-one (3ab(minor))

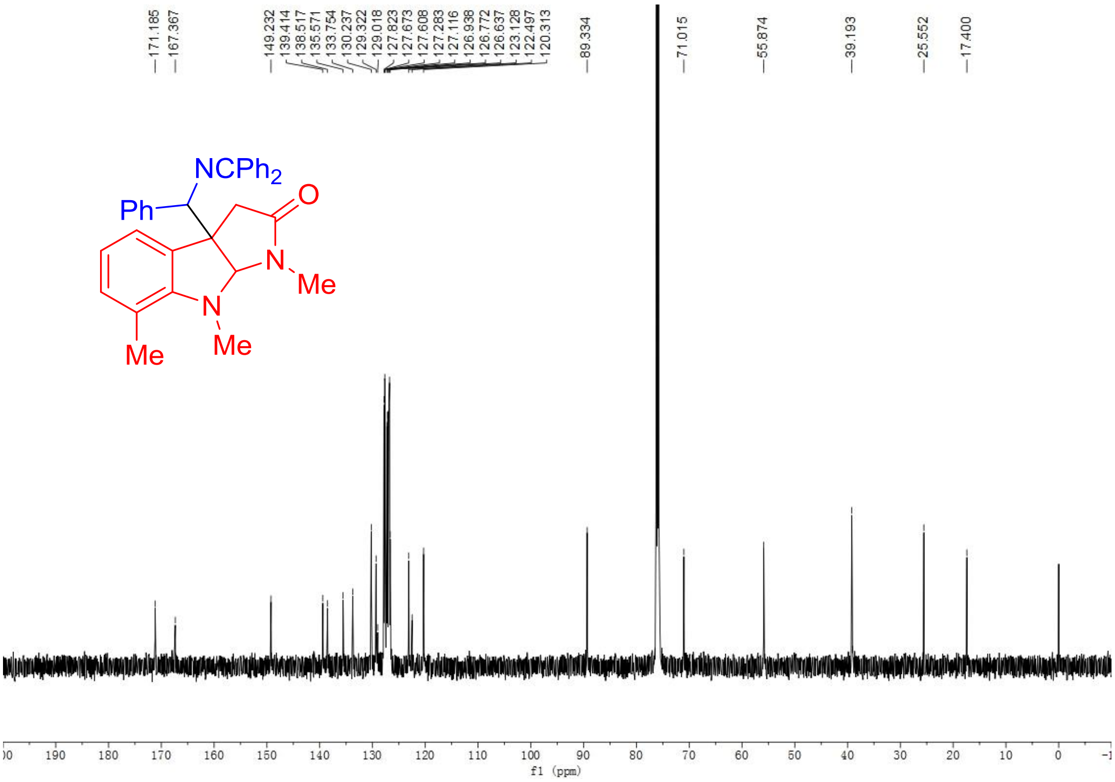

Figure S83. ^{1}H NMR spectra (600 MHz, Chloroform-*d*) of 3a-(((diphenylmethylene)amino)(phenyl)methyl)-5-methoxy-1,8-dimethyl-3,3a,8,8a-tetrahydropyrrolo[2,3-*b*]indol-2(1*H*)-one (3ac(major))

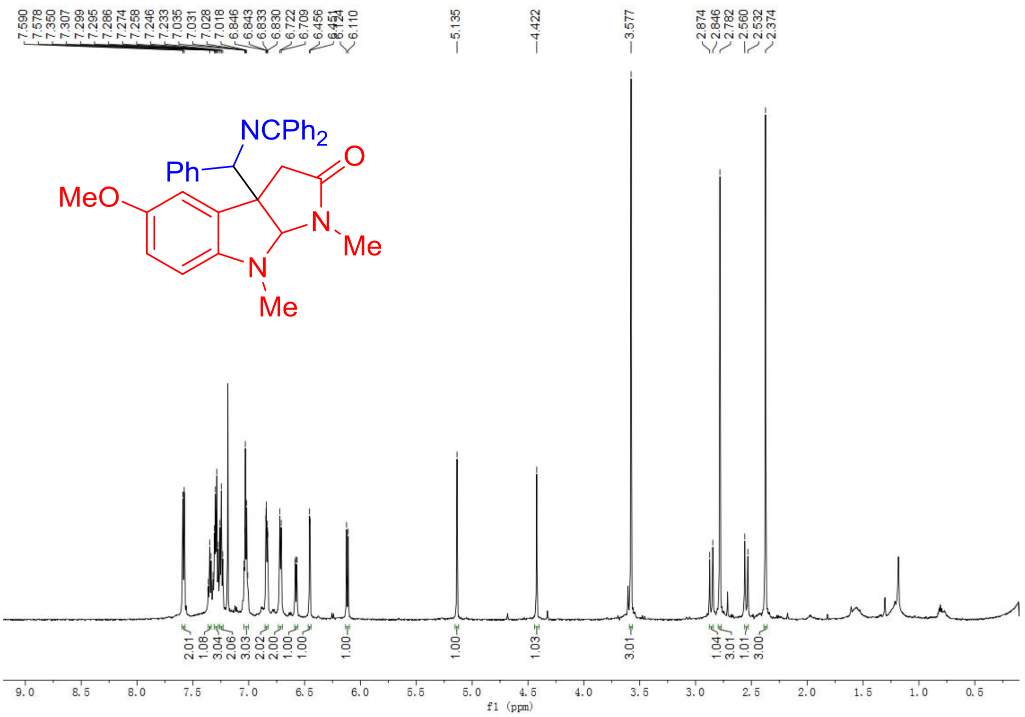

Figure S84. $^{13}C\{^{1}H\}$ NMR spectra (150 MHz, Chloroform-*d*) of 3a-(((diphenylmethylene)amino)(phenyl)methyl)-5-methoxy-1,8-dimethyl-3,3a,8,8a-tetrahydropyrrolo[2,3-*b*]indol-2(1*H*)-one (3ac(major))

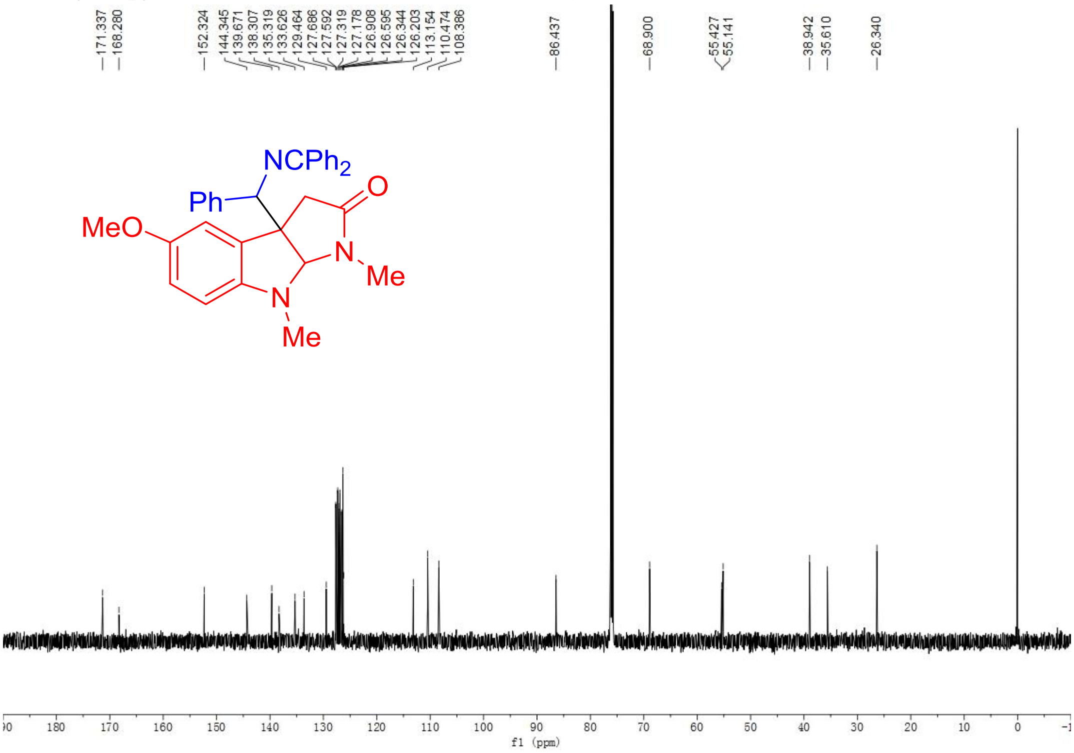

Figure S85. **^{1}H NMR spectra (600 MHz, Chloroform-*d*) of 3a-(((diphenylmethylene)amino)(phenyl)methyl)-5-methoxy-1,8-dimethyl-3,3a,8,8a-tetrahydropyrrolo[2,3-*b*]indol-2(1*H*)-one (3ac(minor))**

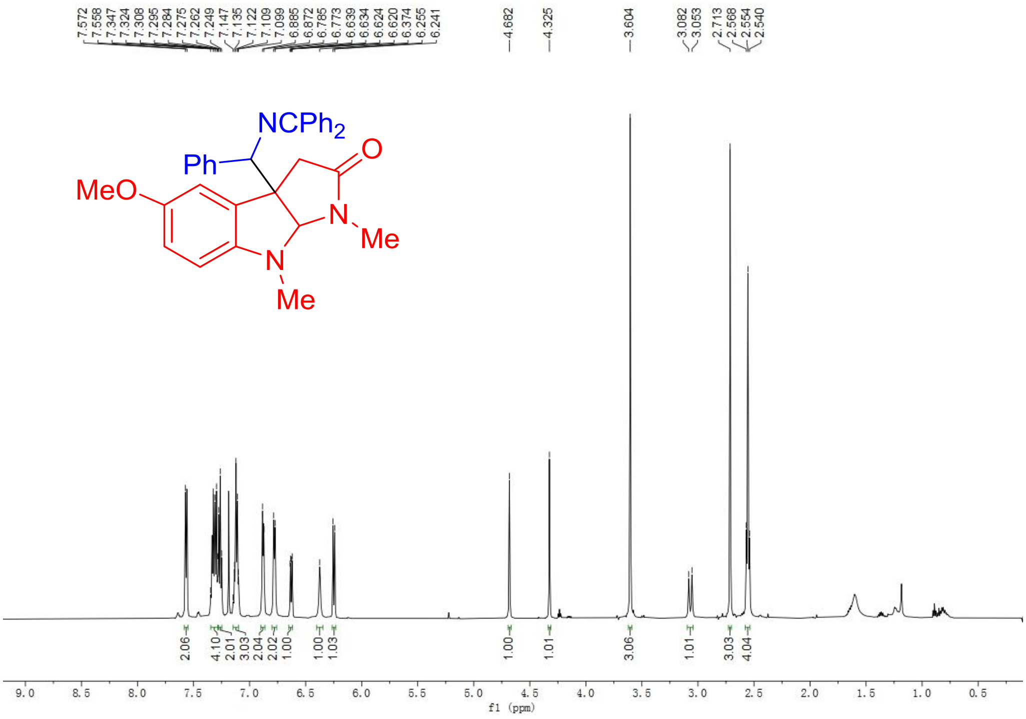

Figure S86. **^{13}C{^{1}H} NMR spectra (150 MHz, Chloroform-*d*) of 3a-(((diphenylmethylene)amino)(phenyl)methyl)-5-methoxy-1,8-dimethyl-3,3a,8,8a-tetrahydropyrrolo[2,3-*b*]indol-2(1*H*)-one (3ac(minor))**

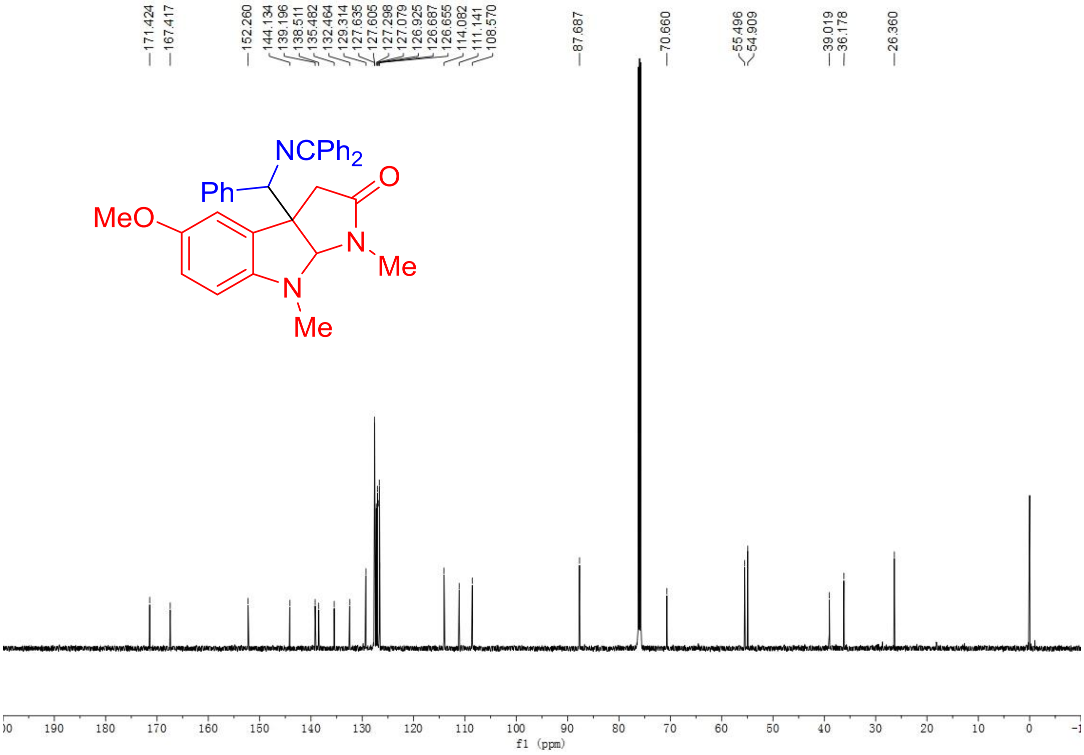

Figure S87. ^{1}H NMR spectra (600 MHz, Chloroform-*d*) of 5-(benzyloxy)-3a-(((diphenylmethylene)amino)(phenyl)methyl)-1,8-dimethyl-3,3a,8,8a-tetrahydropyrrolo[2,3-*b*]indol-2(1*H*)-one (3ad(major))

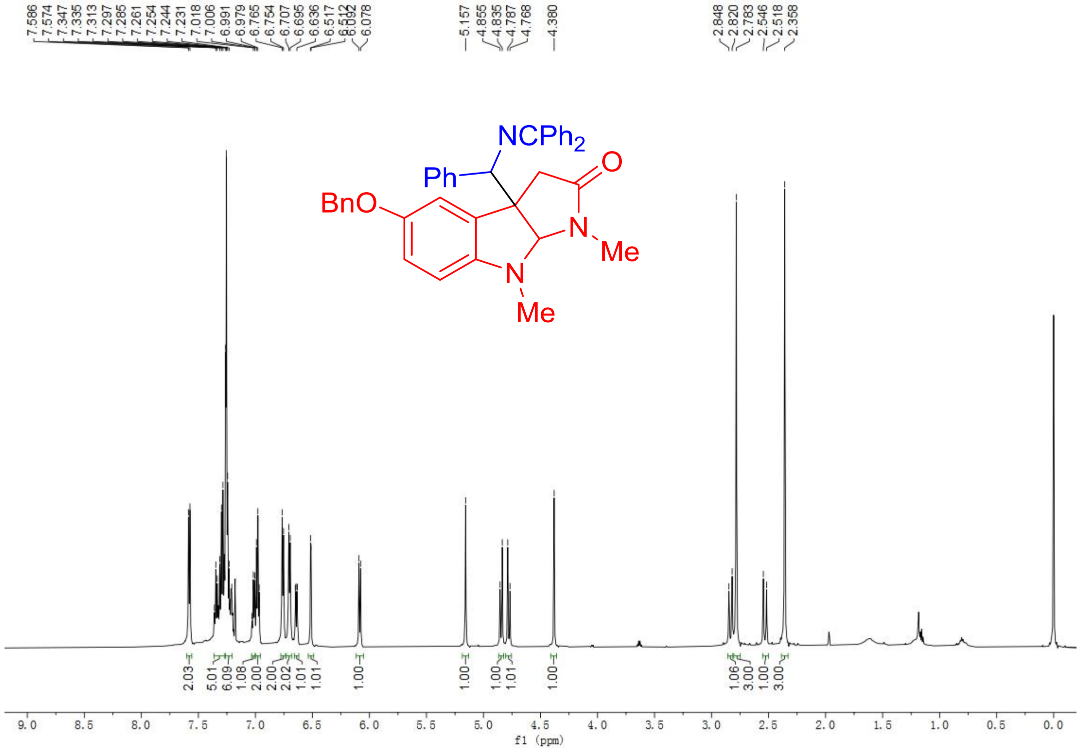

Figure S88. ^{13}C{^{1}H} NMR spectra (150 MHz, Chloroform-*d*) of 5-(benzyloxy)-3a-(((diphenylmethylene)amino)(phenyl)methyl)-1,8-dimethyl-3,3a,8,8a-tetrahydropyrrolo[2,3-*b*]indol-2(1*H*)-one (3ad(major))

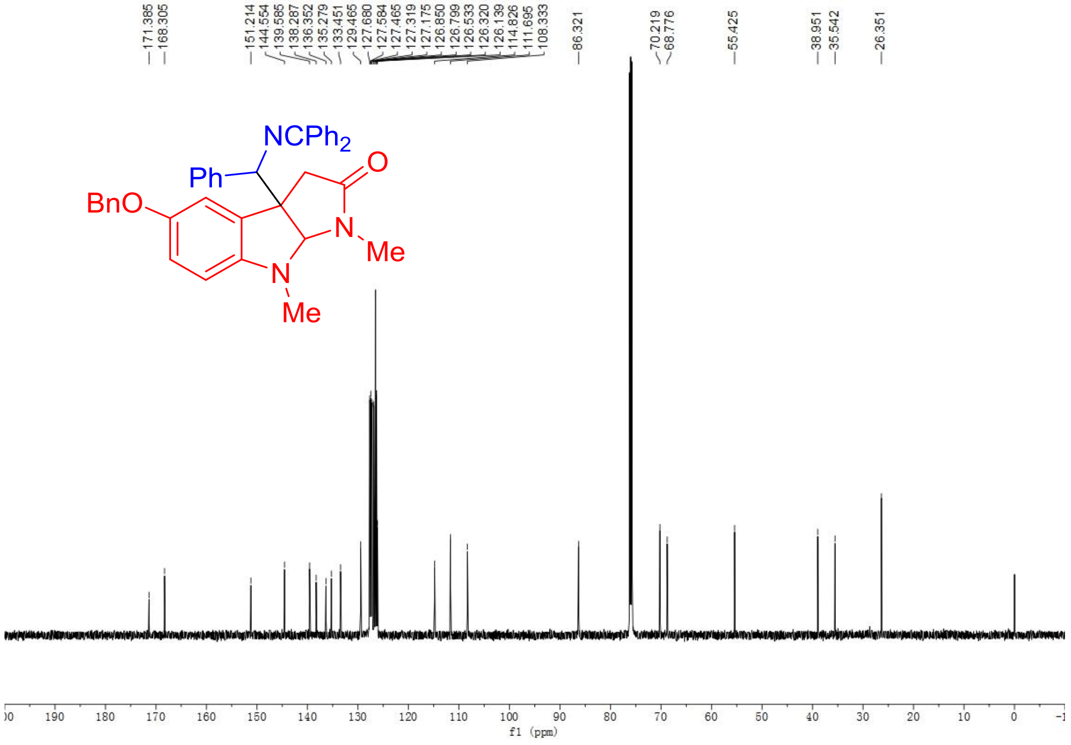

Figure S89. ^{1}H NMR spectra (600 MHz, Chloroform-*d*) of 5-(benzyloxy)-3a-(((diphenylmethylene)amino)(phenyl)methyl)-1,8-dimethyl-3,3a,8,8a-tetrahydropyrrolo[2,3-*b*]indol-2(1*H*)-one (3ad(minor))

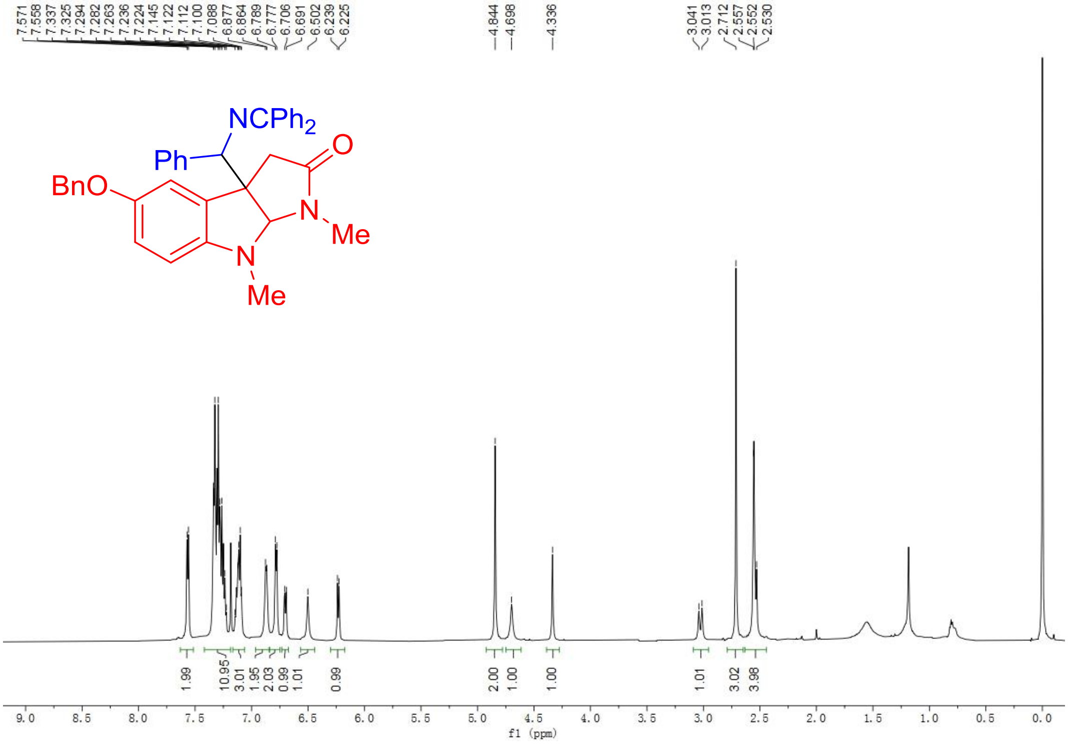

Figure S90. ^{13}C{^{1}H} NMR spectra (150 MHz, Chloroform-*d*) of 5-(benzyloxy)-3a-(((diphenylmethylene)amino)(phenyl)methyl)-1,8-dimethyl-3,3a,8,8a-tetrahydropyrrolo[2,3-*b*]indol-2(1*H*)-one (3ad(minor))

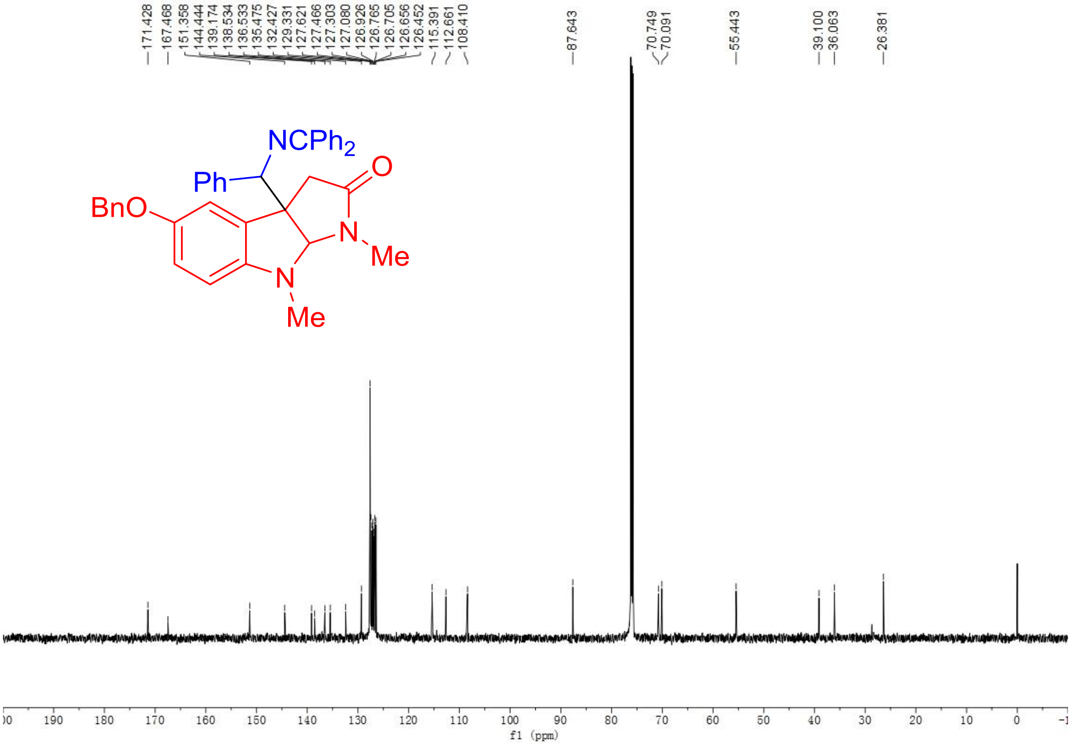

Figure S91. ^{1}H **NMR spectra (600 MHz, Chloroform-*d*) of 3a-(((diphenylmethylene)amino)(phenyl)methyl)-5-fluoro-1,8-dimethyl-3,3a,8,8a-tetrahydropyrrolo[2,3-*b*]indol-2(1*H*)-one (3ae')**

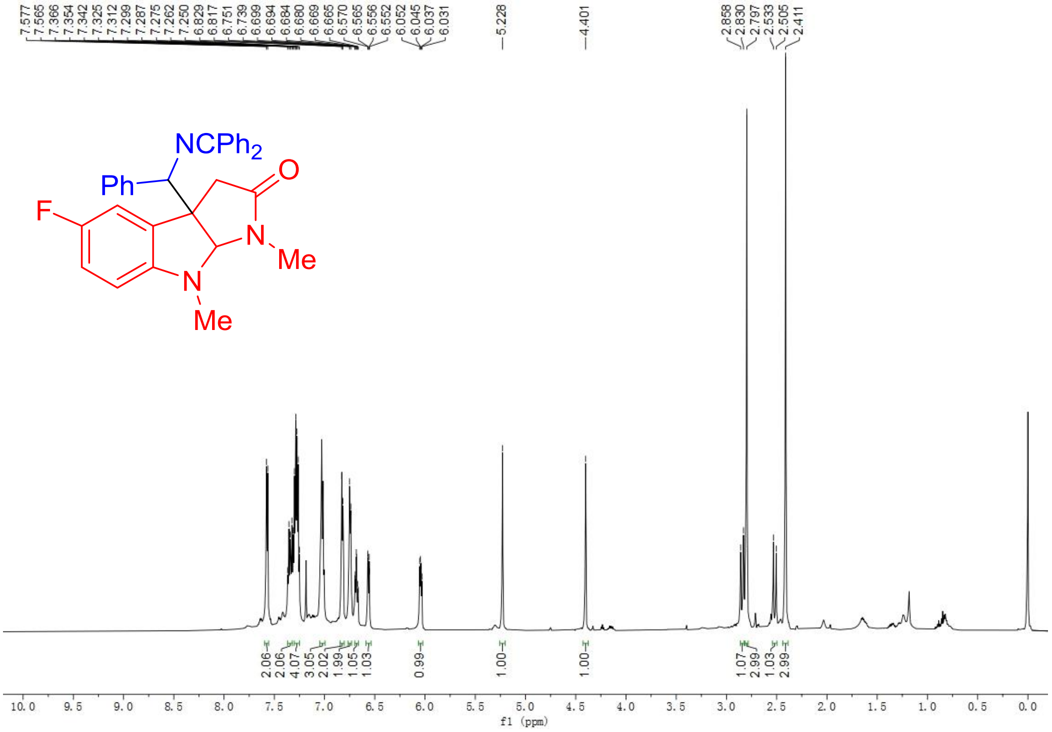

Figure S92. $^{13}C\{^{1}H\}$ **NMR spectra (150 MHz, Chloroform-*d*) of 3a-(((diphenylmethylene)amino)(phenyl)methyl)-5-fluoro-1,8-dimethyl-3,3a,8,8a-tetrahydropyrrolo[2,3-*b*]indol-2(1*H*)-one (3ae')**

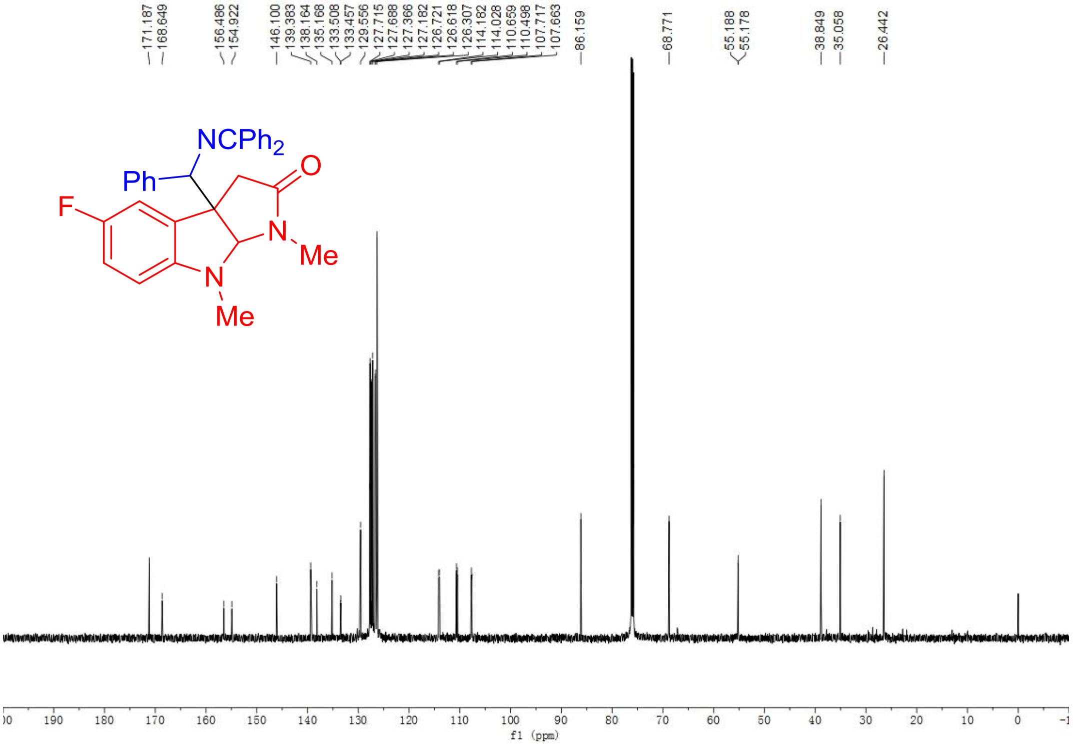

Figure S93. ^{19}F **NMR spectra (565 MHz, Chloroform-*d*) of 3a-(((diphenylmethylene)amino)(phenyl)methyl)-5-fluoro-1,8-dimethyl-3,3a,8,8a-tetrahydropyrrolo[2,3-*b*]indol-2(1*H*)-one (3ae')**

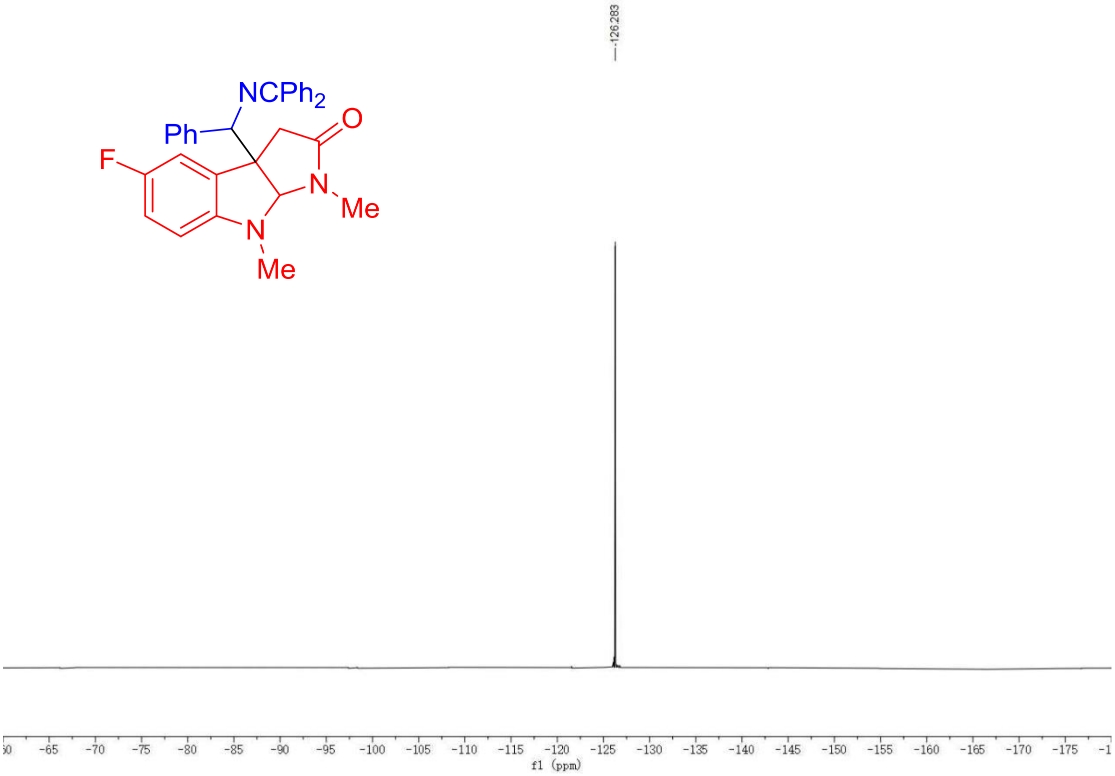

Figure S94. ^{1}H NMR spectra (600 MHz, Chloroform-*d*) of 3a-(((diphenylmethylene)amino)(phenyl)methyl)-5-fluoro-1,8-dimethyl-3,3a,8,8a-tetrahydropyrrolo[2,3-*b*]indol-2(1*H*)-one (3ae")

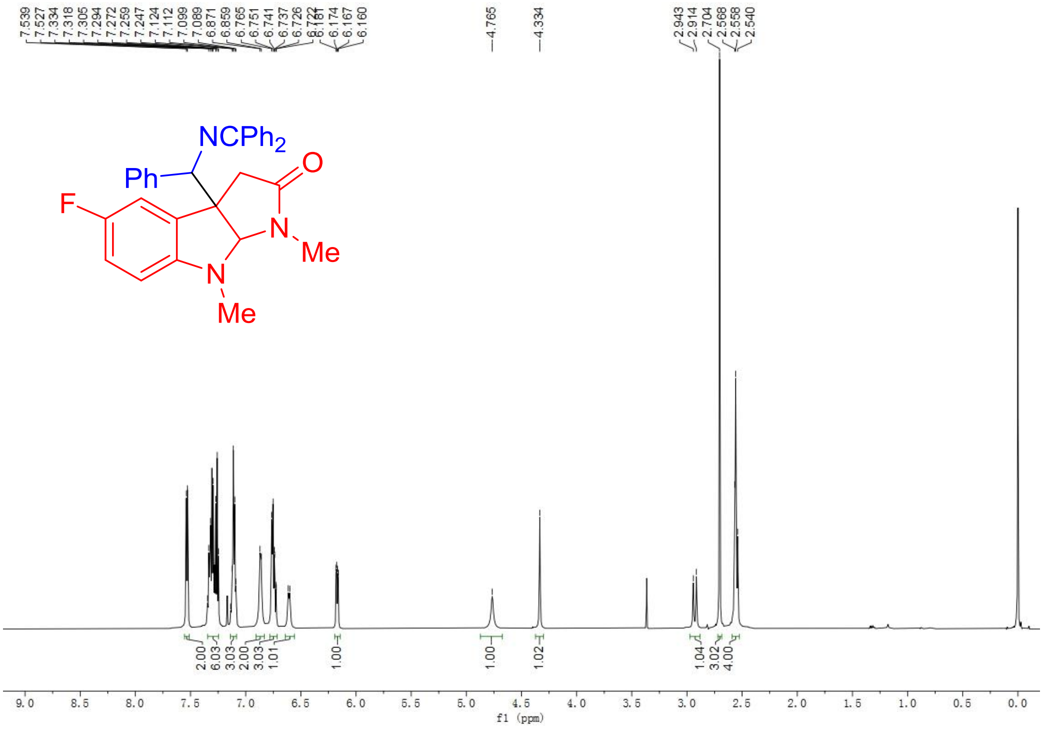

Figure S95. $^{13}C\{^{1}H\}$ NMR spectra (150 MHz, Chloroform-*d*) of 3a-(((diphenylmethylene)amino)(phenyl)methyl)-5-fluoro-1,8-dimethyl-3,3a,8,8a-tetrahydropyrrolo[2,3-*b*]indol-2(1*H*)-one (3ae")

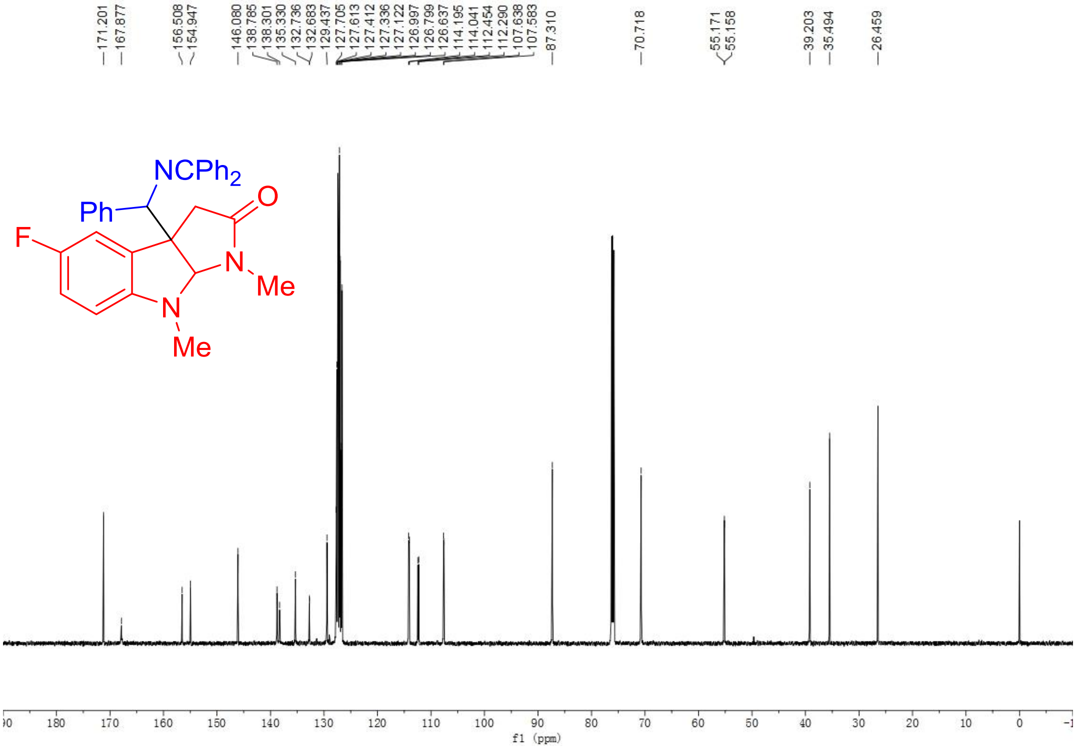

Figure S96. ^{19}**F NMR spectra (565 MHz, Chloroform-*d*) of 3a-(((diphenylmethylene)amino)(phenyl)methyl)-5-fluoro-1,8-dimethyl-3,3a,8,8a-tetrahydropyrrolo[2,3-*b*]indol-2(1*H*)-one (3ae")**

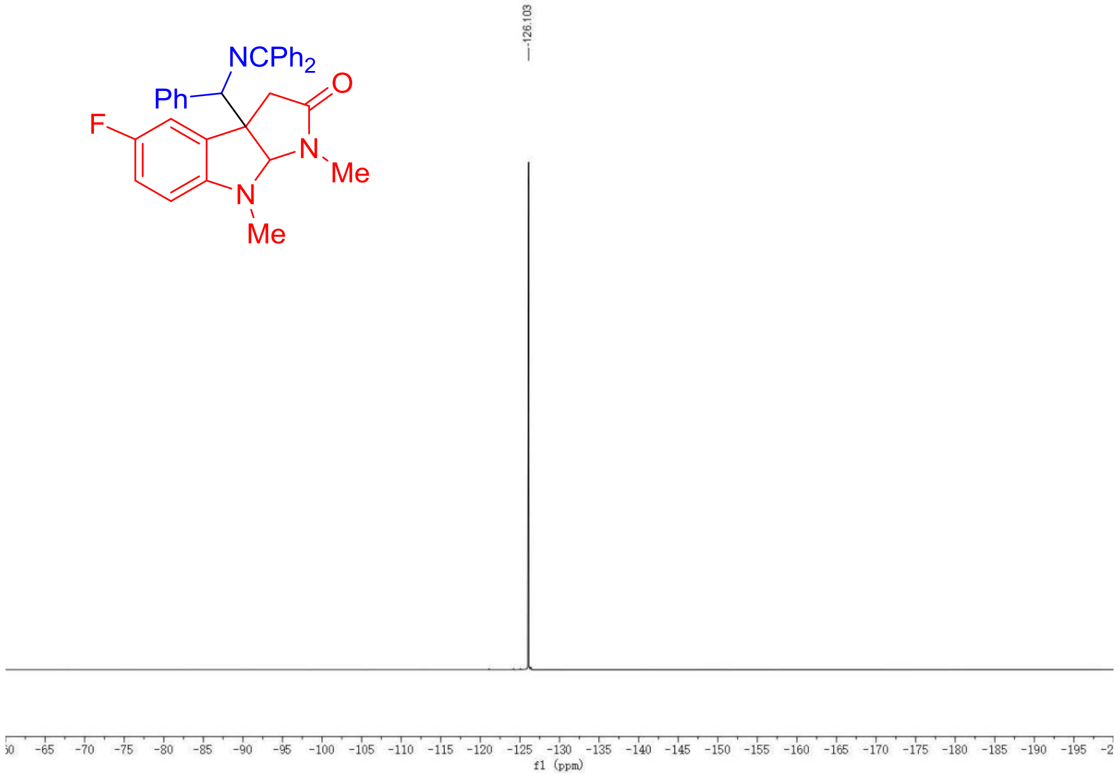

Figure S97. ^{1}H NMR spectra (600 MHz, Chloroform-*d*) of 5-bromo-3a-(((diphenylmethylene)amino)(phenyl)methyl)-1,8-dimethyl-3,3a,8,8a-tetrahydropyrrolo[2,3-*b*]indol-2(1*H*)-one (3af(major))

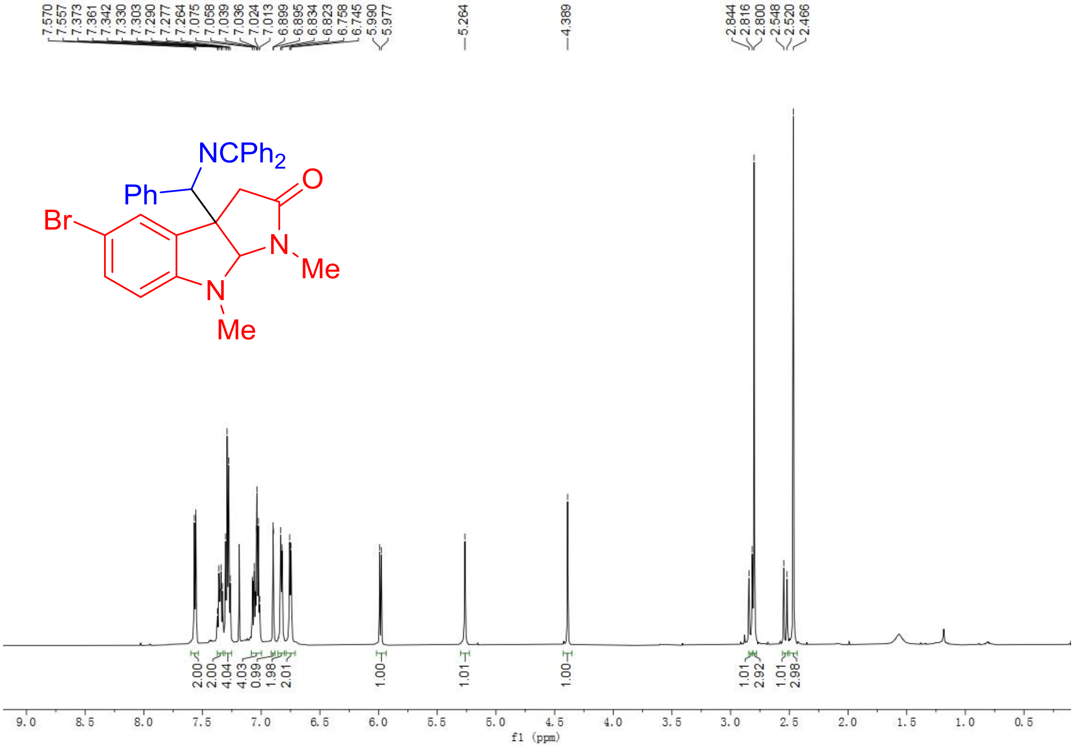

Figure S98. $^{13}C\{^{1}H\}$ NMR spectra (150 MHz, Chloroform-*d*) of 5-bromo-3a-(((diphenylmethylene)amino)(phenyl)methyl)-1,8-dimethyl-3,3a,8,8a-tetrahydropyrrolo[2,3-*b*]indol-2(1*H*)-one (3af(major))

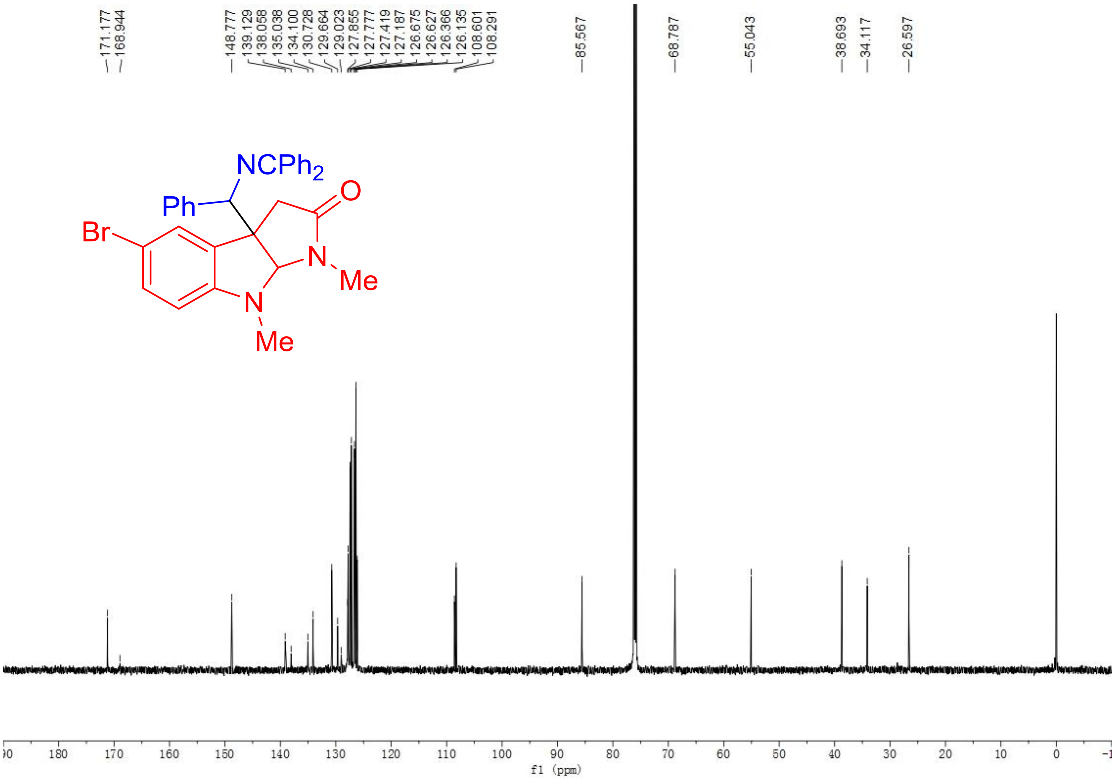

Figure S99. ^{1}H **NMR spectra (600 MHz, Chloroform-*d*) of 5-bromo-3a-(((diphenylmethylene)amino)(phenyl)methyl)-1,8-dimethyl-3,3a,8,8a-tetrahydropyrrolo[2,3-*b*]indol-2(1*H*)-one (3af(minor))**

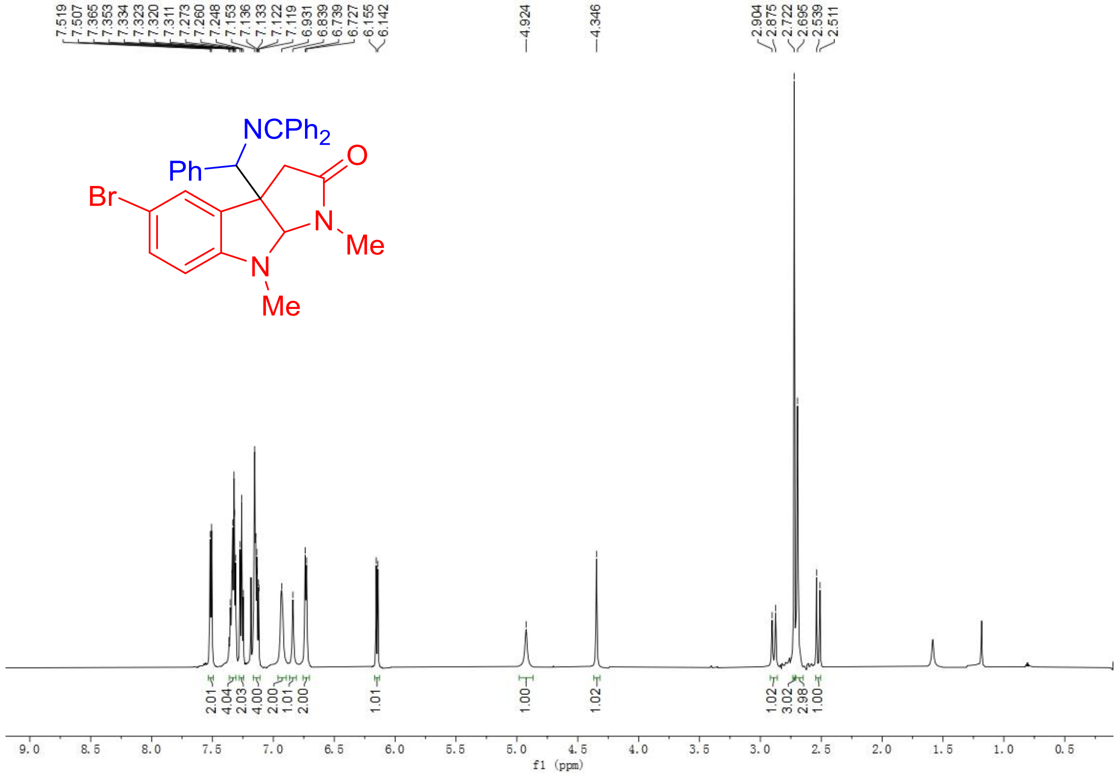

Figure S100. $^{13}C\{^{1}H\}$ **NMR spectra (150 MHz, Chloroform-*d*) of 5-bromo-3a-(((diphenylmethylene)amino)(phenyl)methyl)-1,8-dimethyl-3,3a,8,8a-tetrahydropyrrolo[2,3-*b*]indol-2(1*H*)-one (3af(minor))**

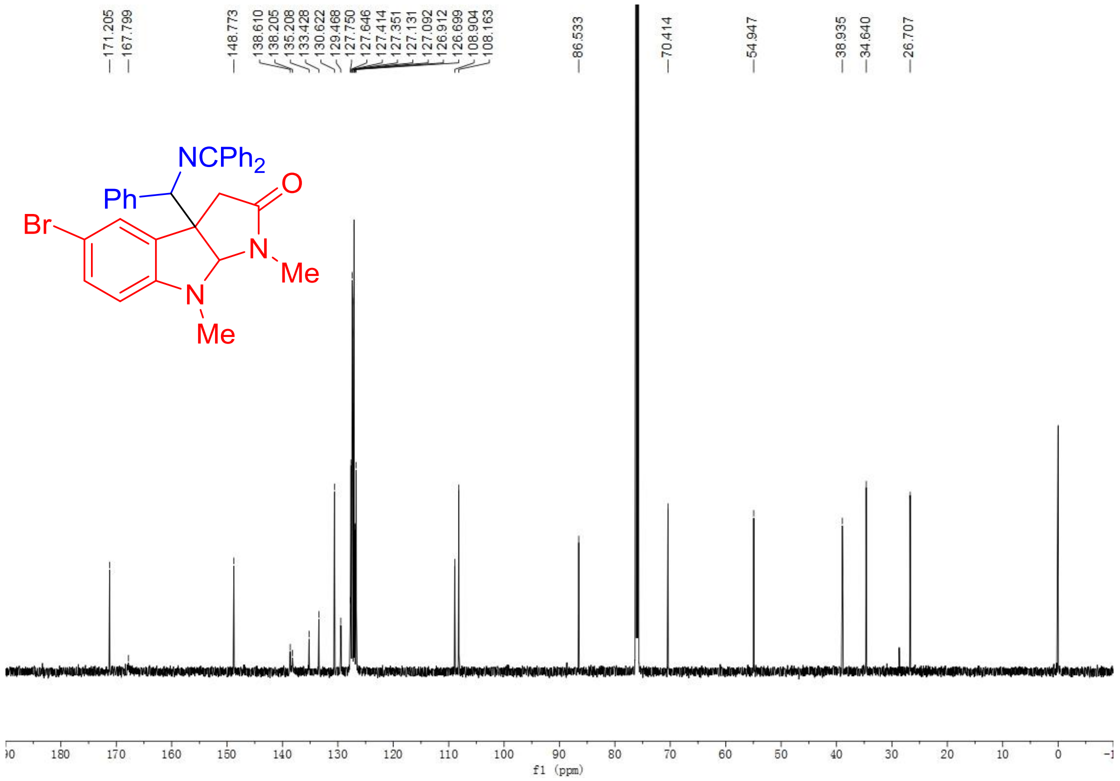

Figure S101. ^{1}**H NMR spectra (600 MHz, Methanol-*d*$_4$) of 8a-(((diphenylmethylene)amino)(phenyl)methyl)-5,6-dimethyl-5a,6,8,8a-tetrahydro-[1,3]dioxolo[4,5-*f*]pyrrolo[2,3-*b*]indol-7(5*H*)-one (3ag(major))**

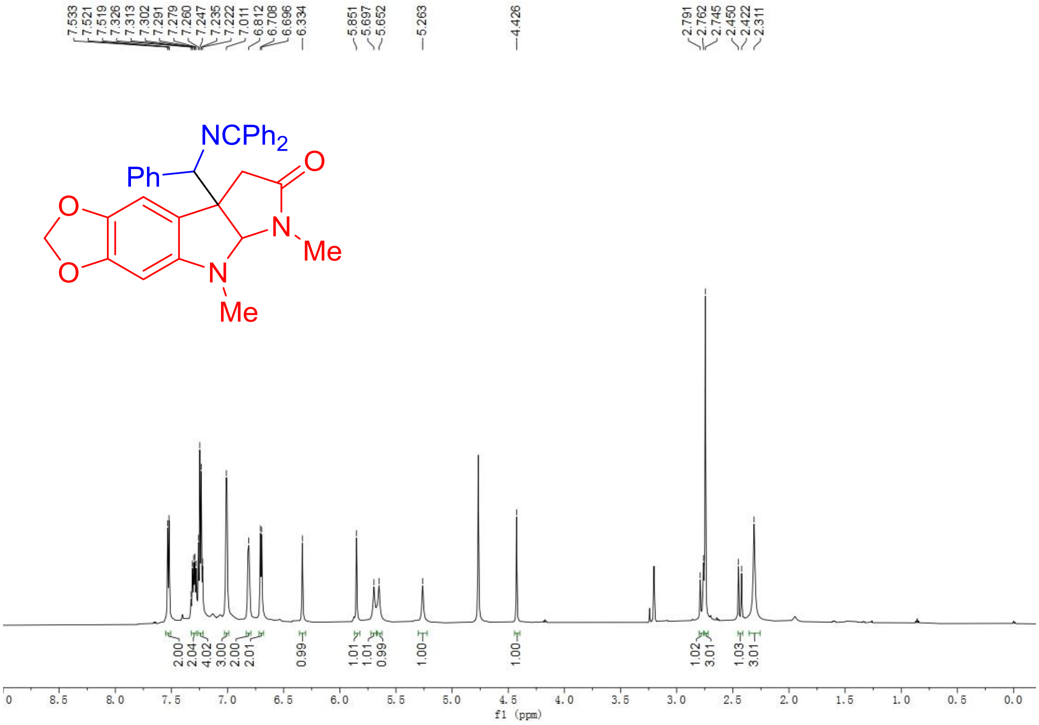

Figure S102. ^{13}**C{^{1}H} NMR spectra (150 MHz, Methanol-*d*$_4$) of 8a-(((diphenylmethylene)amino)(phenyl)methyl)-5,6-dimethyl-5a,6,8,8a-tetrahydro-[1,3]dioxolo[4,5-*f*]pyrrolo[2,3-*b*]indol-7(5*H*)-one (3ag(major))**

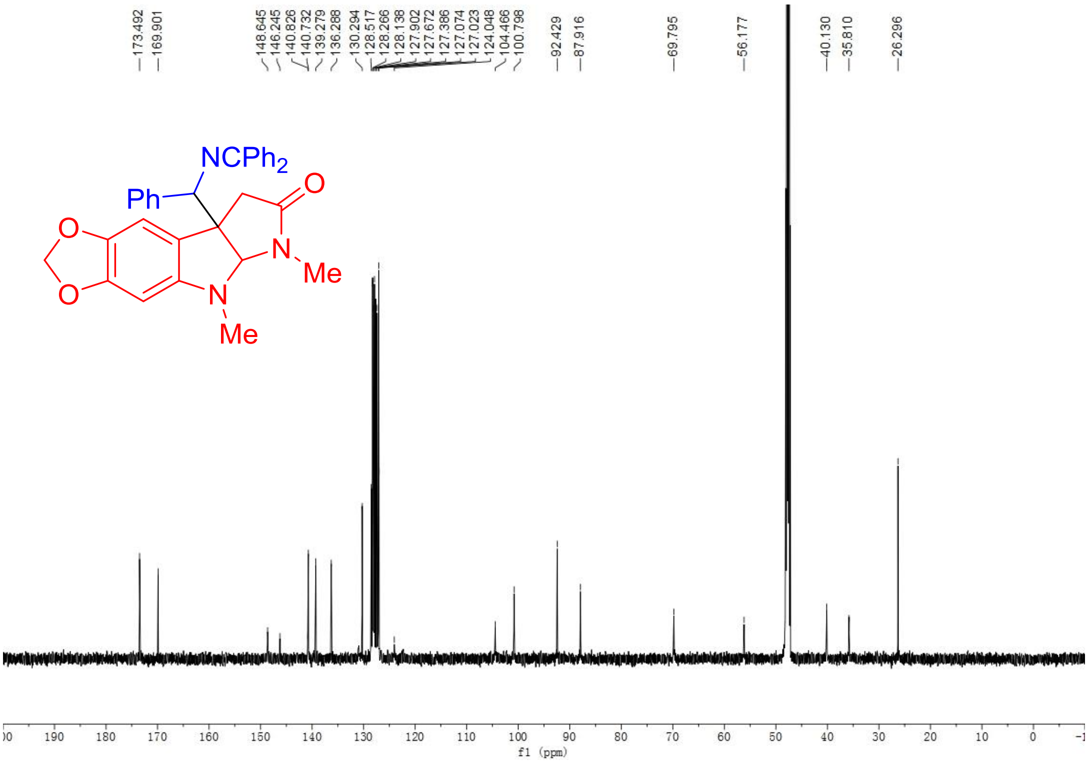

Figure S103. ^{1}**H NMR spectra (600 MHz, Methanol-*d*$_4$) of 8a-(((diphenylmethylene)amino)(phenyl)methyl)-5,6-dimethyl-5a,6,8,8a-tetrahydro-[1,3]dioxolo[4,5-*f*]pyrrolo[2,3-*b*]indol-7(5*H*)-one (3ag(minor))**

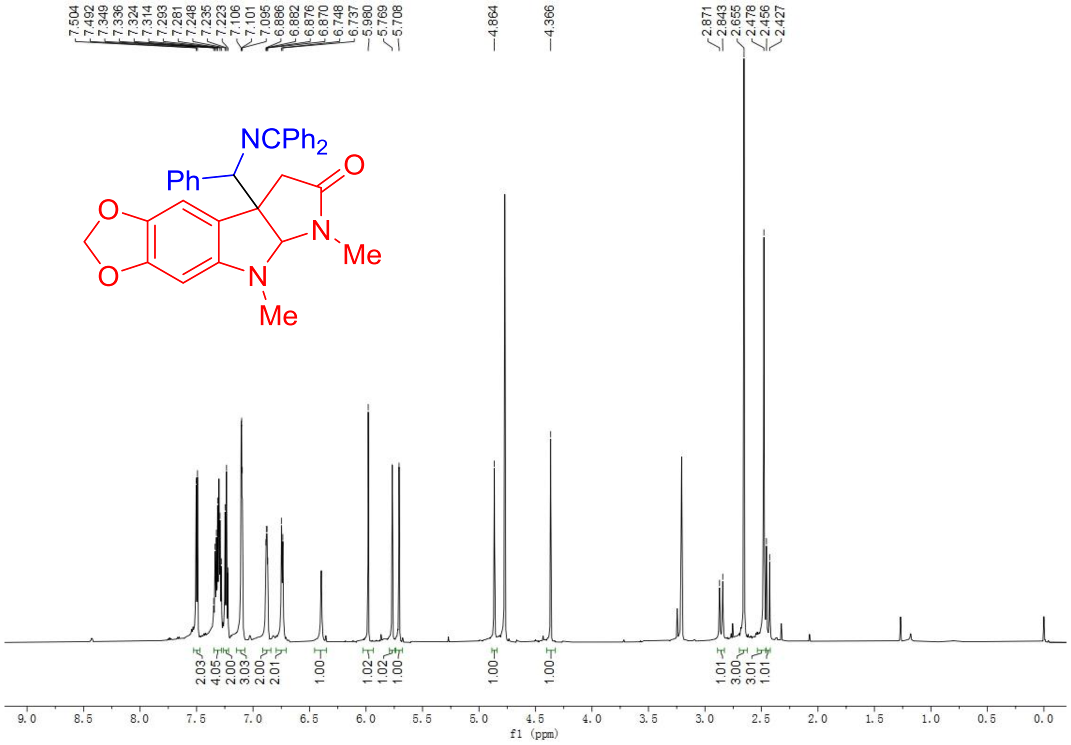

Figure S104. ^{13}**C{^{1}H} NMR spectra (150 MHz, Methanol-*d*$_4$) of 8a-(((diphenylmethylene)amino)(phenyl)methyl)-5,6-dimethyl-5a,6,8,8a-tetrahydro-[1,3]dioxolo[4,5-*f*]pyrrolo[2,3-*b*]indol-7(5*H*)-one (3ag(minor))**

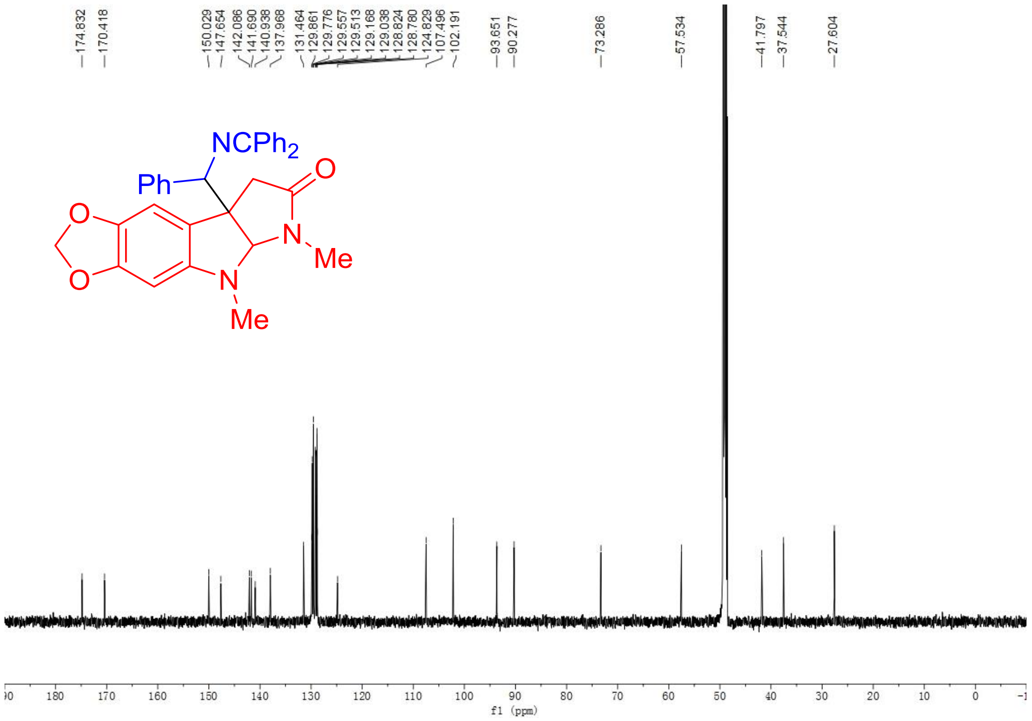

Figure S105. ^{1}H **NMR spectra (600 MHz, Chloroform-*d*) of 6b-(((diphenylmethylene)amino)(phenyl)methyl)-9,10-dimethyl-6b,9,9a,10-tetrahydrobenzo[*g*]pyrrolo[2,3-*b*]indol-8(7*H*)-one (3ah')**

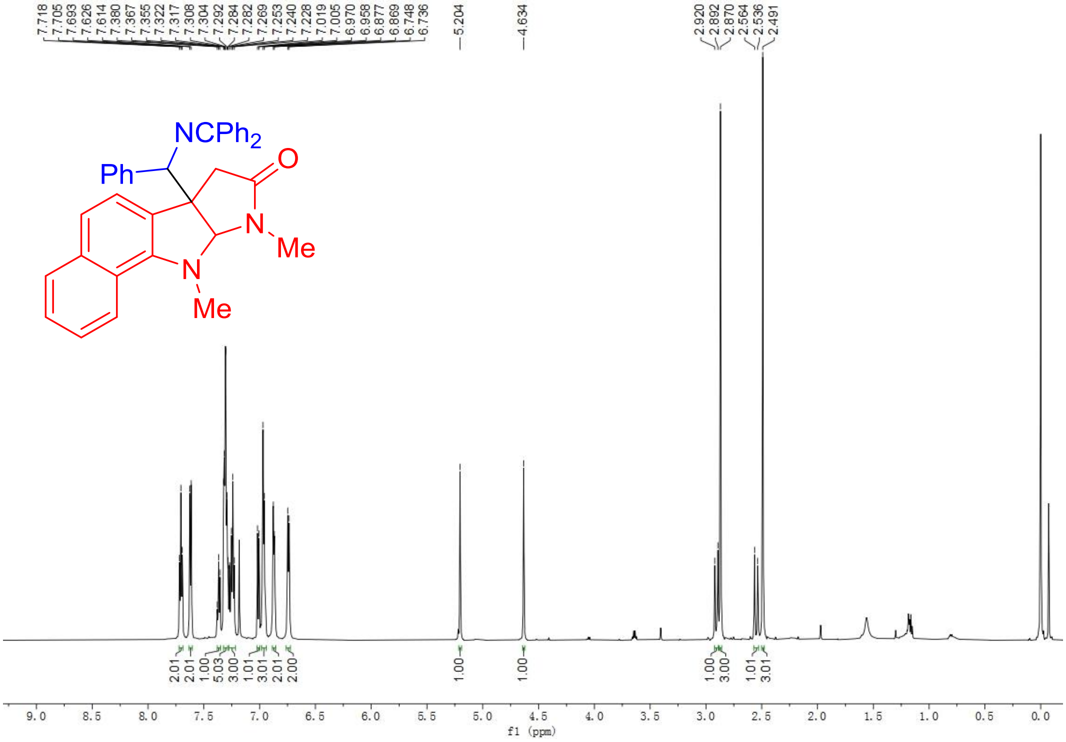

Figure S106. $^{13}C\{^{1}H\}$ **NMR spectra (150 MHz, Chloroform-*d*) of 6b-(((diphenylmethylene)amino)(phenyl)methyl)-9,10-dimethyl-6b,9,9a,10-tetrahydrobenzo[*g*]pyrrolo[2,3-*b*]indol-8(7*H*)-one (3ah')**

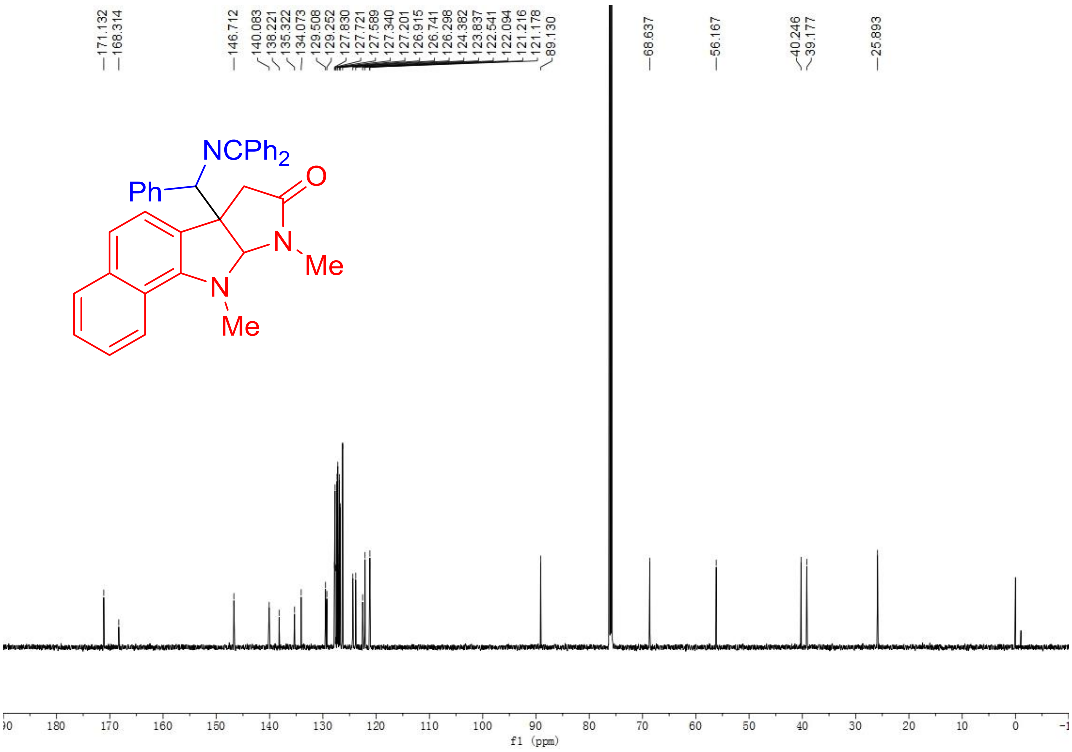

Figure S107. ^{1}H NMR spectra (600 MHz, Chloroform-*d*) of 6b-(((diphenylmethylene)amino)(phenyl)methyl)-9,10-dimethyl-6b,9,9a,10-tetrahydrobenzo[*g*]pyrrolo[2,3-*b*]indol-8(7*H*)-one (3ah'')

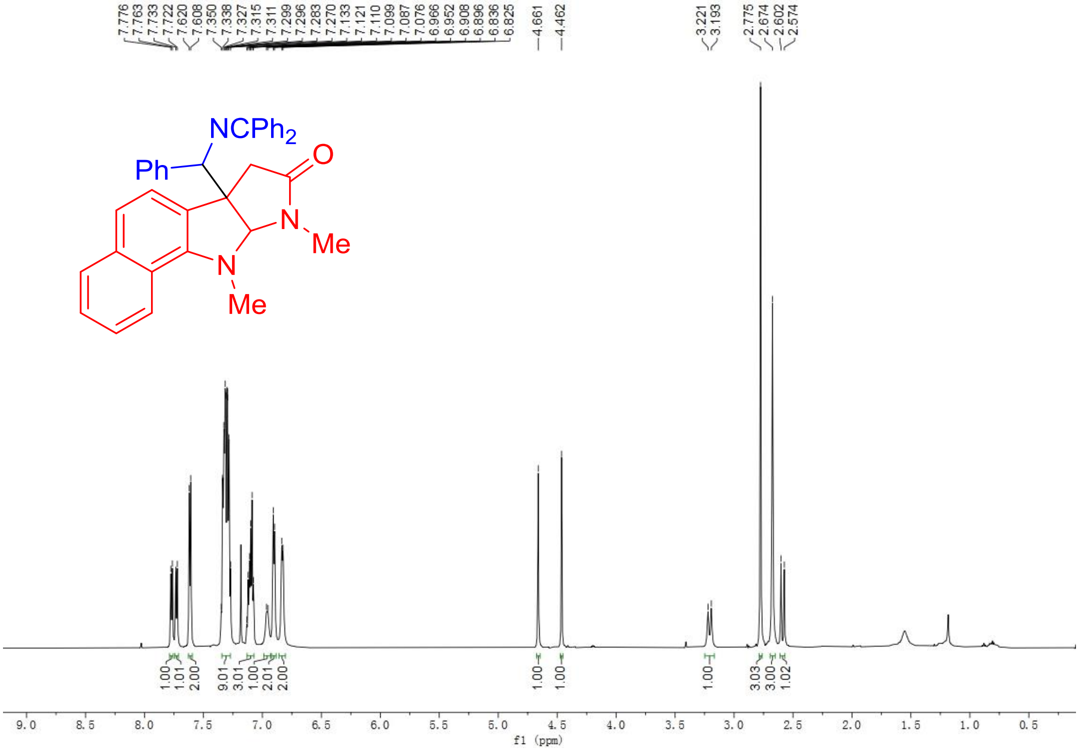

Figure S108. ^{13}C{^{1}H} NMR spectra (150 MHz, Chloroform-*d*) of 6b-(((diphenylmethylene)amino)(phenyl)methyl)-9,10-dimethyl-6b,9,9a,10-tetrahydrobenzo[*g*]pyrrolo[2,3-*b*]indol-8(7*H*)-one (3ah'')

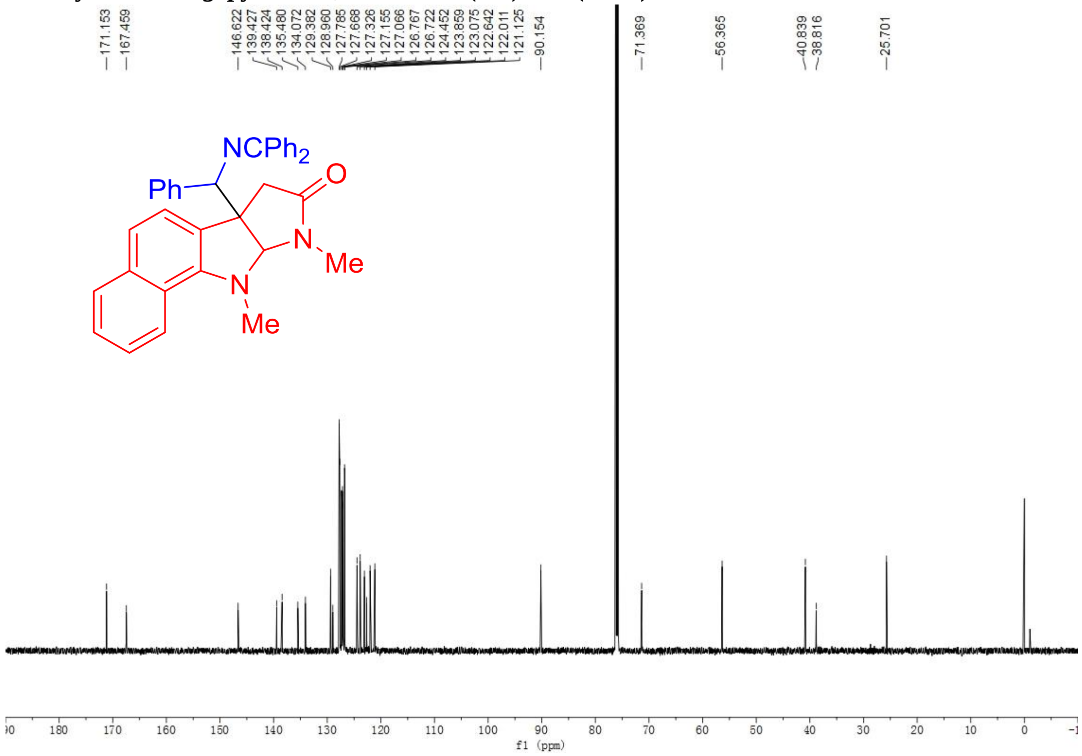

Figure S109. ^{1}**H NMR spectra (600 MHz, Methanol-*d*$_4$) of 4a-(((diphenylmethylene)amino)(phenyl)methyl)-1,9-dimethyl-1,3,4,4a,9,9a-hexahydro-2*H*-pyrido[2,3-*b*]indol-2-one (3ai(major))**

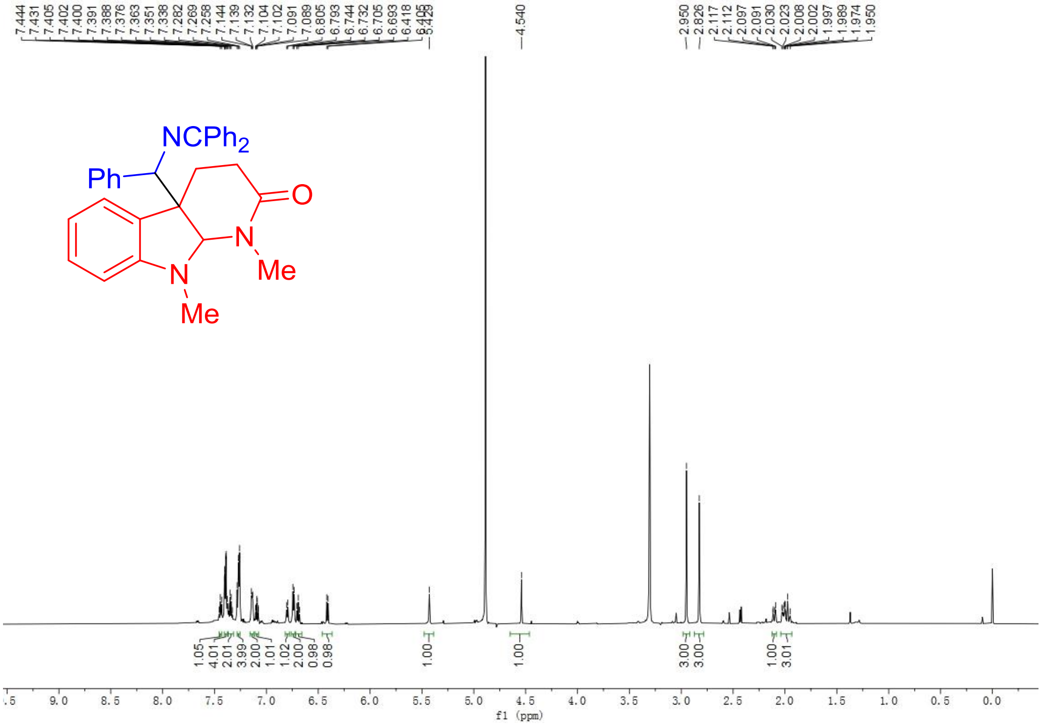

Figure S110. ^{13}**C{**^{1}**H} NMR spectra (150 MHz, Methanol-*d*$_4$) of 4a-(((diphenylmethylene)amino)(phenyl)methyl)-1,9-dimethyl-1,3,4,4a,9,9a-hexahydro-2*H*-pyrido[2,3-*b*]indol-2-one (3ai(major))**

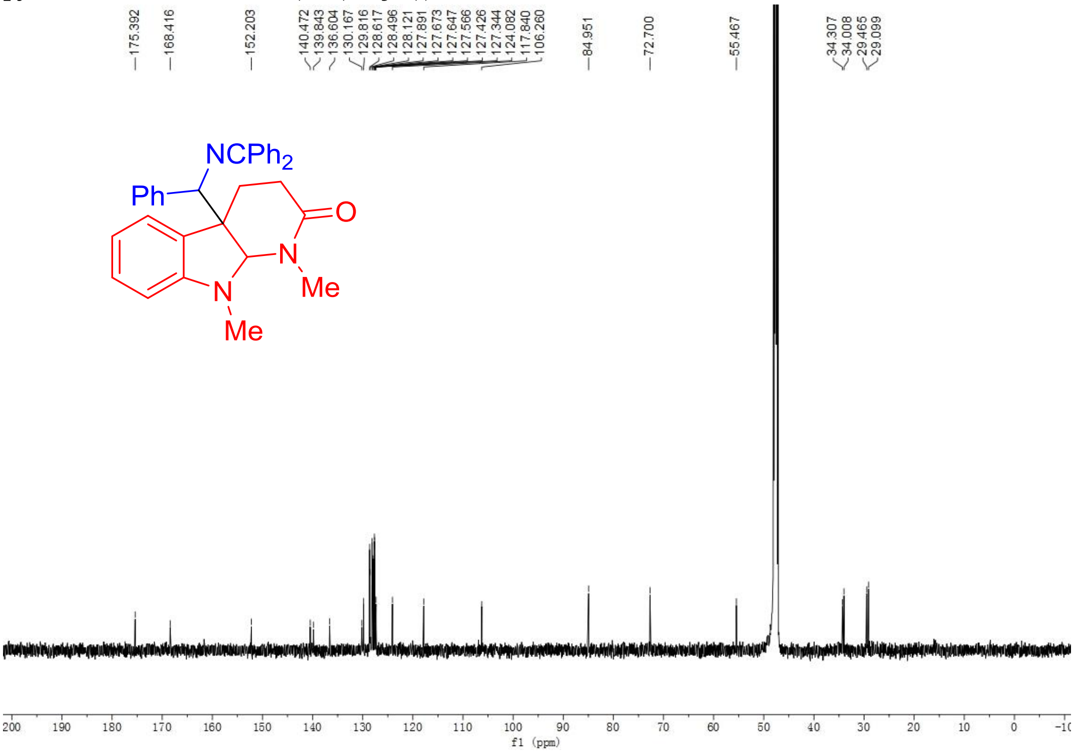

Figure S111. ^{1}H NMR spectra (600 MHz, Methanol-d_4) of 4a-(((diphenylmethylene)amino)(phenyl)methyl)-1,9-dimethyl-1,3,4,4a,9,9a-hexahydro-2*H*-pyrido[2,3-*b*]indol-2-one (3ai(minor))

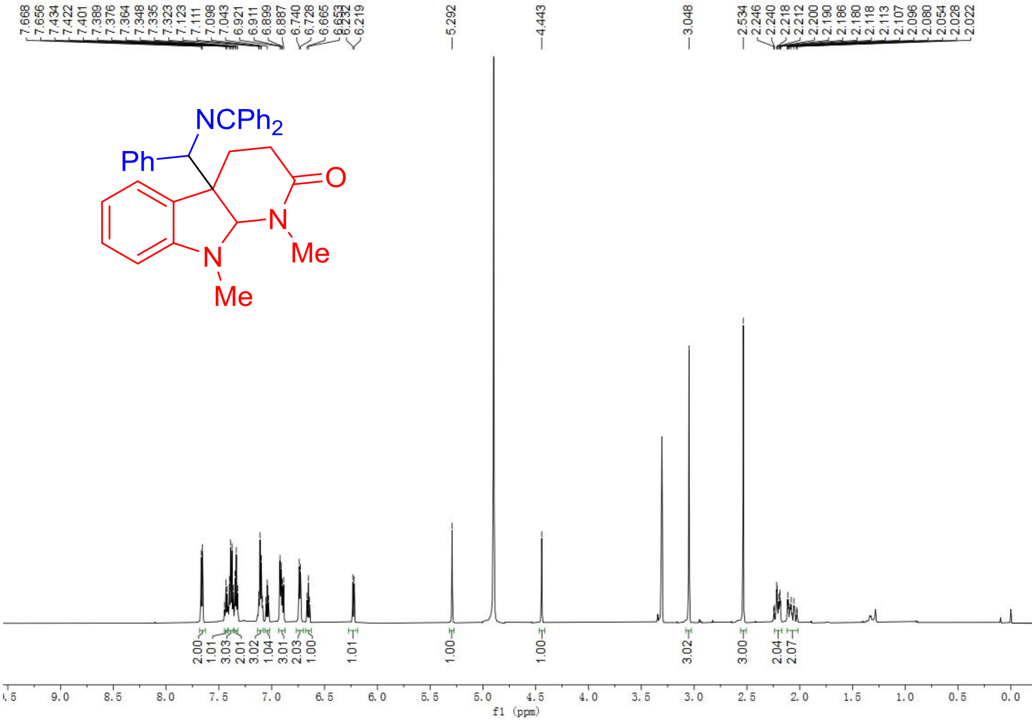

Figure S112. ^{13}C{^{1}H} NMR spectra (150 MHz, Methanol-d_4) of 4a-(((diphenylmethylene)amino)(phenyl)methyl)-1,9-dimethyl-1,3,4,4a,9,9a-hexahydro-2*H*-pyrido[2,3-*b*]indol-2-one (3ai (minor))

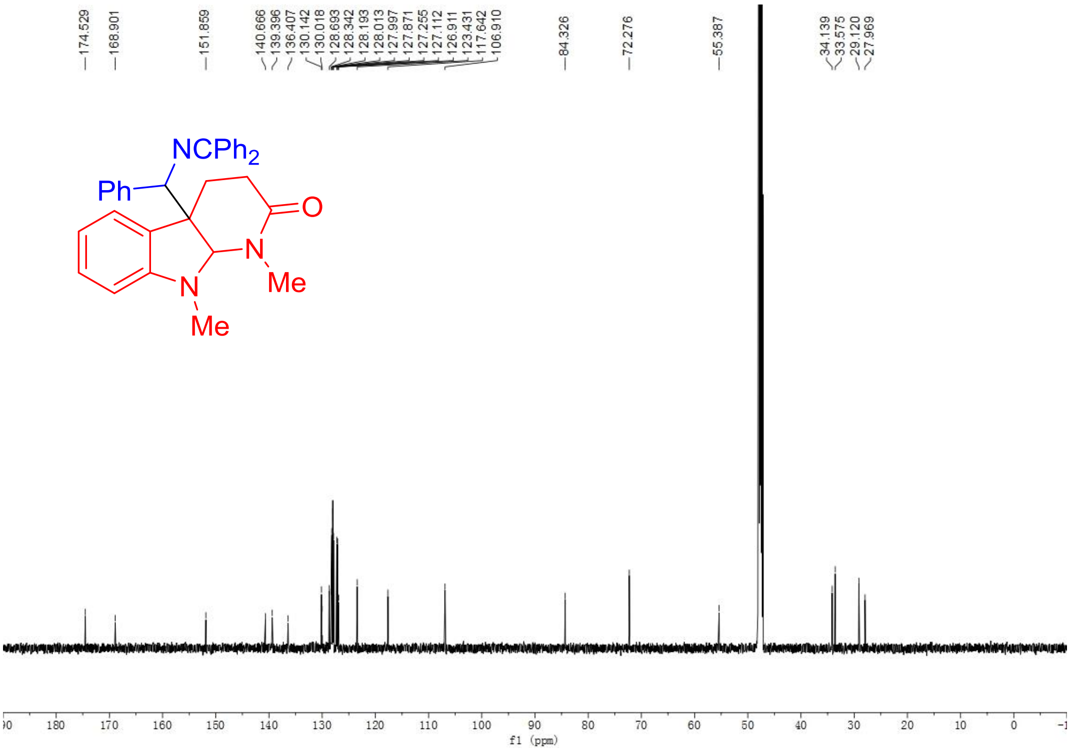

Figure S113. ^{1}H NMR spectra (600 MHz, Methanol-d_4) of 5a-(((diphenylmethylene)amino)(phenyl)methyl)-1,10-dimethyl-3,4,5,5a,10,10a-hexahydroazepino[2,3-*b*]indol-2(1*H*)-one (3aj(major))

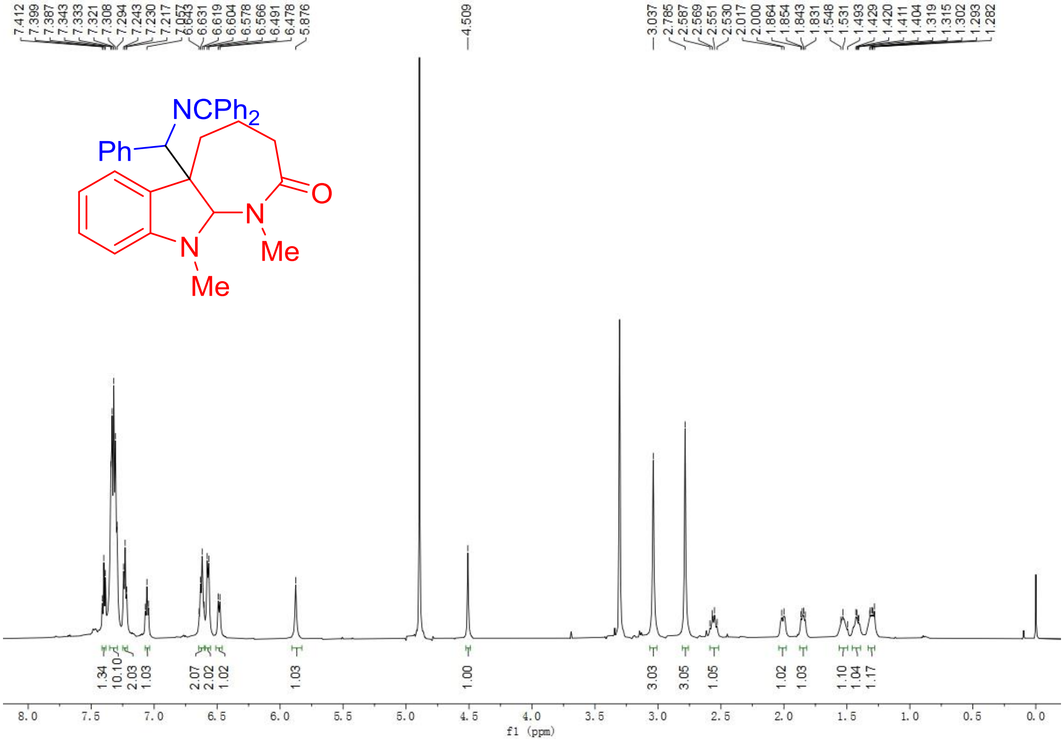

Figure S114. ^{13}C{^{1}H} NMR spectra (150 MHz, Methanol-d_4) of 5a-(((diphenylmethylene)amino)(phenyl)methyl)-1,10-dimethyl-3,4,5,5a,10,10a-hexahydroazepino[2,3-*b*]indol-2(1*H*)-one (3aj(major))

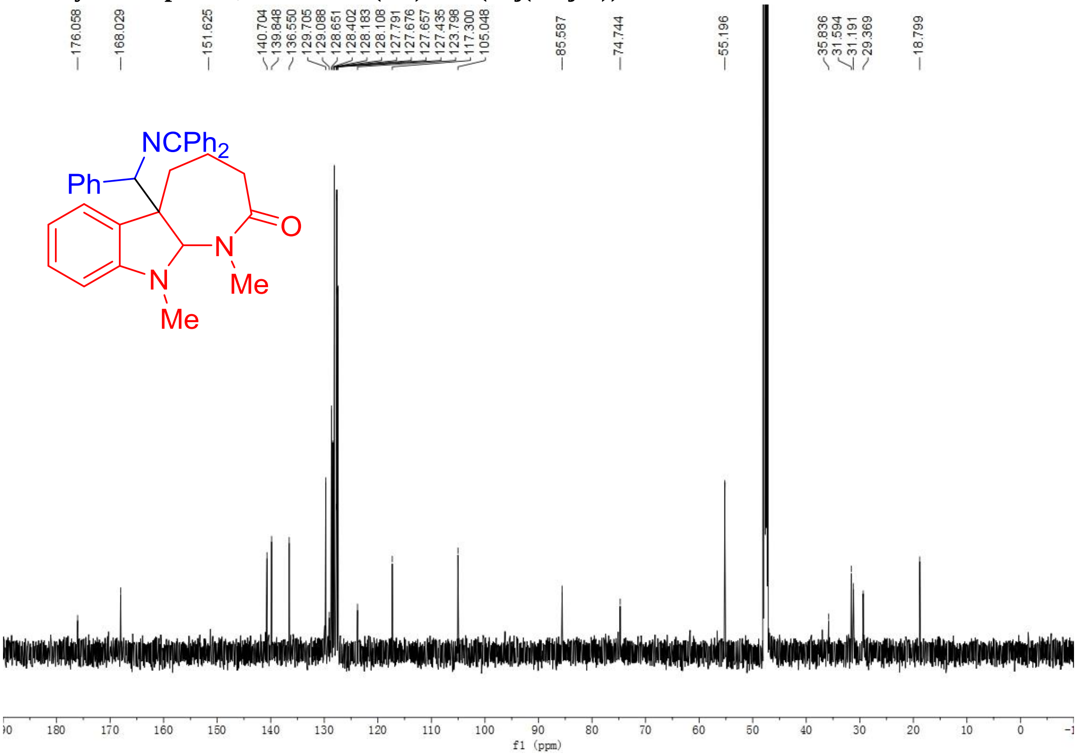

Figure S115. ^{1}H **NMR spectra (600 MHz, Chloroform-*d*) of 5a-(((diphenylmethylene)amino)(phenyl)methyl)-1,10-dimethyl-3,4,5,5a,10,10a-hexahydroazepino[2,3-*b*]indol-2(1*H*)-one (3aj(minor))**

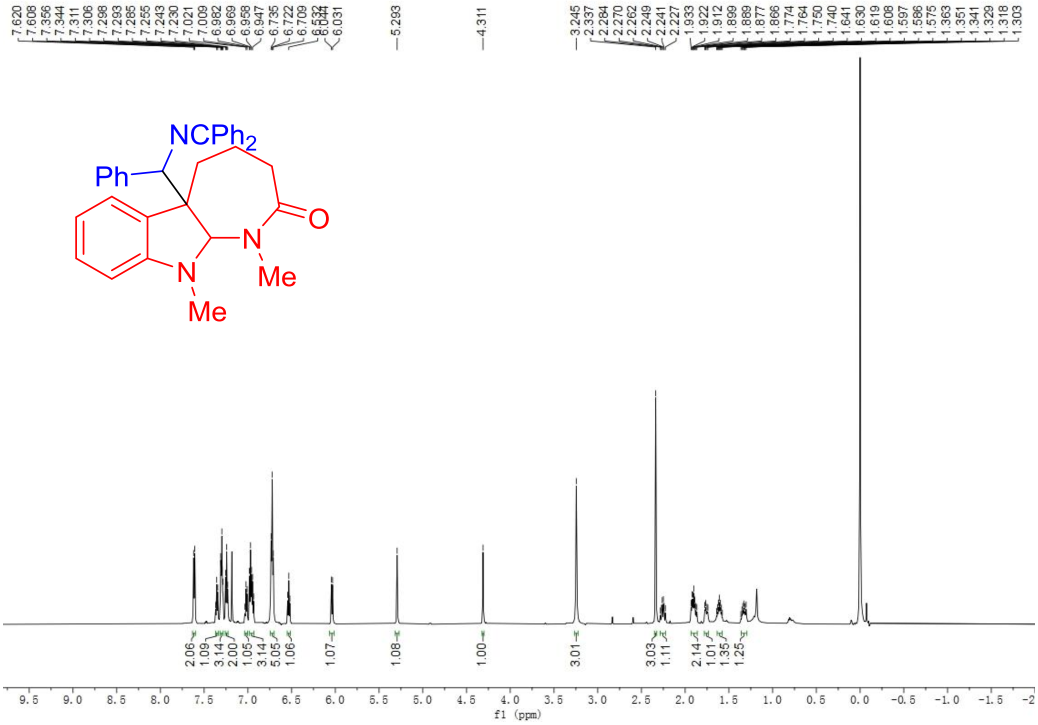

Figure S116. $^{13}C\{^{1}H\}$ **NMR spectra (150 MHz, Chloroform-*d*) of 5a-(((diphenylmethylene)amino)(phenyl)methyl)-1,10-dimethyl-3,4,5,5a,10,10a-hexahydroazepino[2,3-*b*]indol-2(1*H*)-one (3aj(minor))**

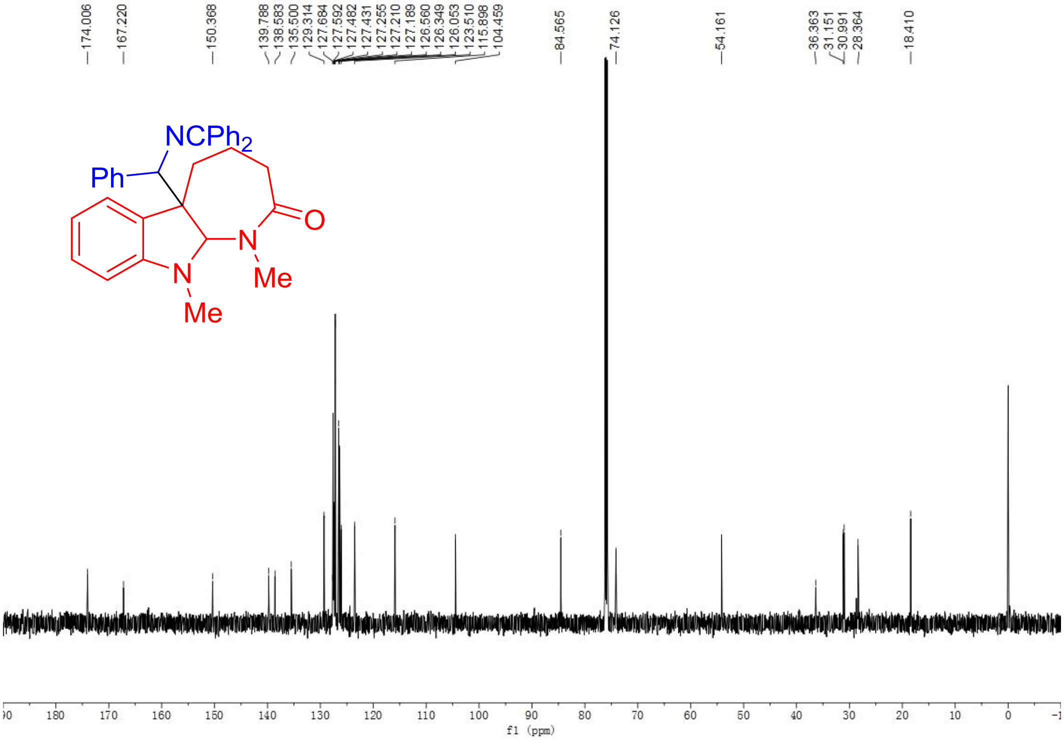

Figure S117. ^{1}H NMR spectra (600 MHz, Chloroform-*d*) of 3a-(((diphenylmethylene)amino)(phenyl)methyl)-1,8,8a-trimethyl-3,3a,8,8a-tetrahydropyrrolo[2,3-*b*]indol-2(1*H*)-one (3ak')

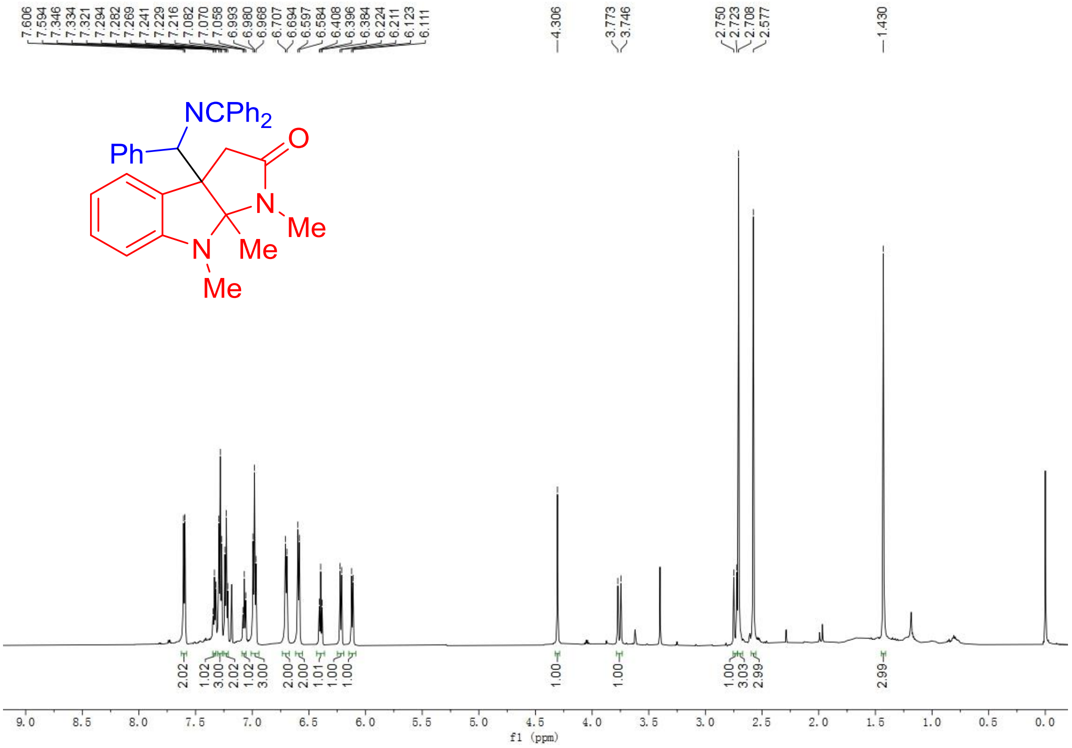

Figure S118. $^{13}C\{^{1}H\}$ NMR spectra (150 MHz, Chloroform-*d*) of 3a-(((diphenylmethylene)amino)(phenyl)methyl)-1,8,8a-trimethyl-3,3a,8,8a-tetrahydropyrrolo[2,3-*b*]indol-2(1*H*)-one (3ak')

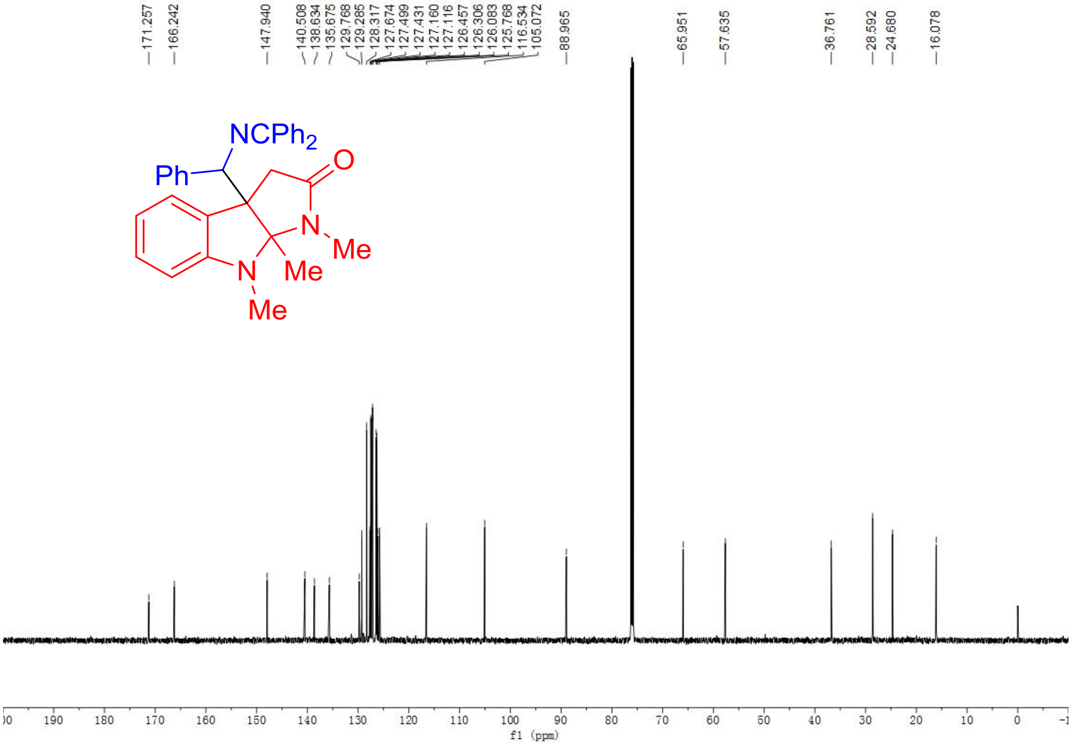

Figure S119. ^{1}H NMR spectra (400 MHz, Chloroform-*d*) of 3a-(((diphenylmethylene)amino)(phenyl)methyl)-1,8,8a-trimethyl-3,3a,8,8a-tetrahydropyrrolo[2,3-*b*]indol-2(1*H*)-one (3ak”)

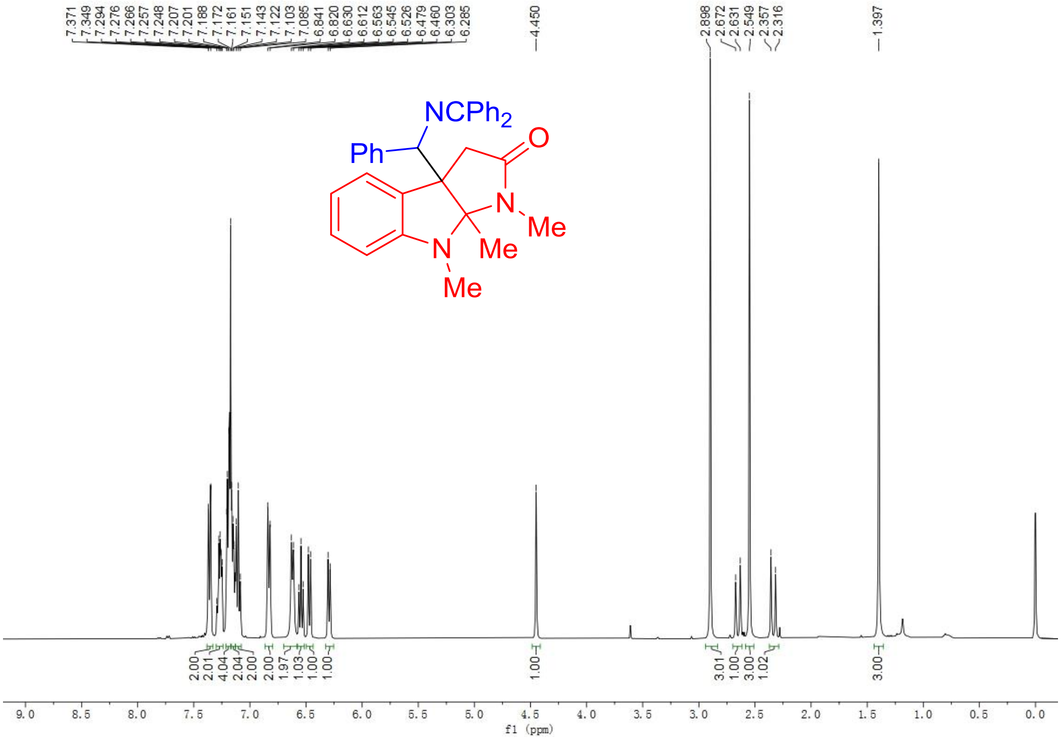

Figure S120. ^{13}C{^{1}H} spectra (100 MHz, Chloroform-*d*) of 3a-(((diphenylmethylene)amino)(phenyl)methyl)-1,8,8a-trimethyl-3,3a,8,8a-tetrahydropyrrolo[2,3-*b*]indol-2(1*H*)-one (3ak”)

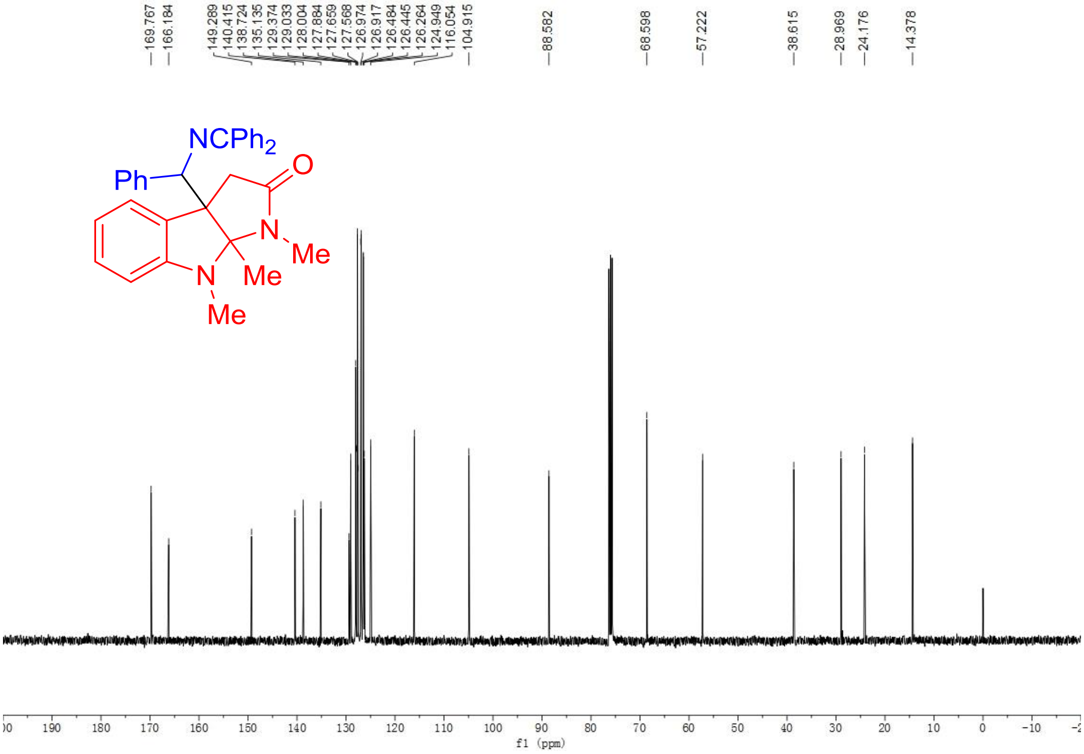

Figure S121. ^{1}H **NMR spectra (600 MHz, Chloroform-*d*) of 3a-(((diphenylmethylene)amino)(phenyl)methyl)-8a-ethyl-1,8-dimethyl-3,3a,8,8a-tetrahydropyrrolo[2,3-*b*]indol-2(1*H*)-one (3al(major))**

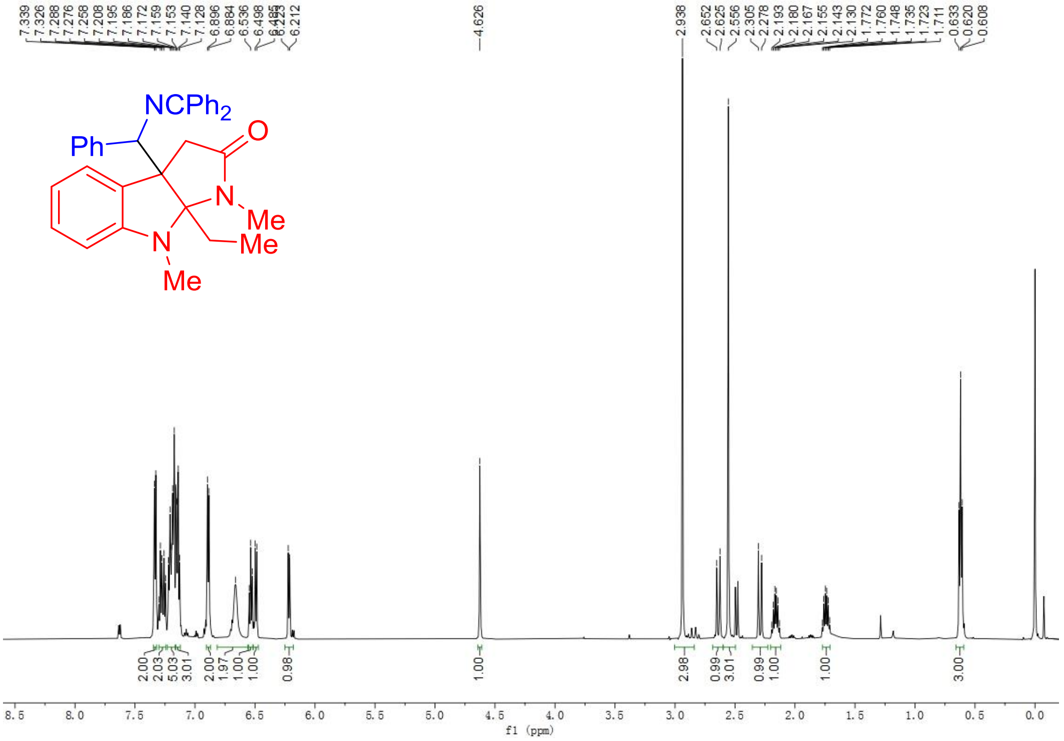

Figure S122. $^{13}C\{^{1}H\}$ **NMR spectra (150 MHz, Chloroform-*d*) of 3a-(((diphenylmethylene)amino)(phenyl)methyl)-8a-ethyl-1,8-dimethyl-3,3a,8,8a-tetrahydropyrrolo[2,3-*b*]indol-2(1*H*)-one (3al(major))**

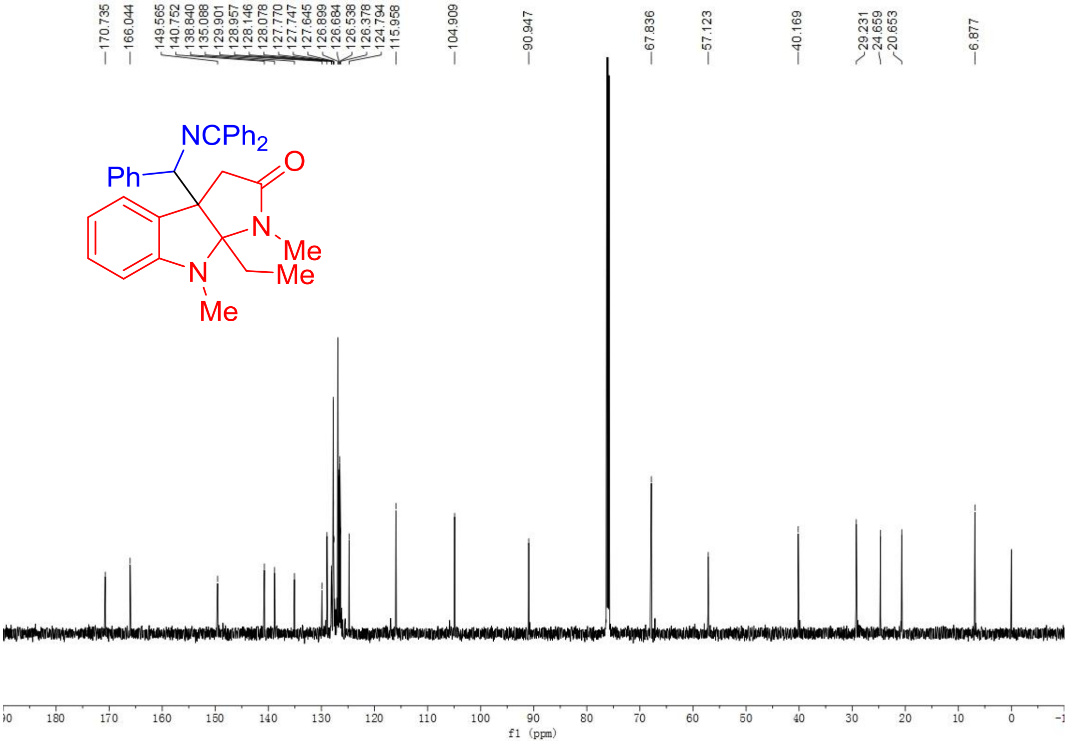

Figure S123. ^{1}H NMR spectra (600 MHz, Chloroform-*d*) of 3a-(((diphenylmethylene)amino)(phenyl)methyl)-8a-ethyl-1,8-dimethyl-3,3a,8,8a-tetrahydropyrrolo[2,3-*b*]indol-2(1*H*)-one (3al(minor))

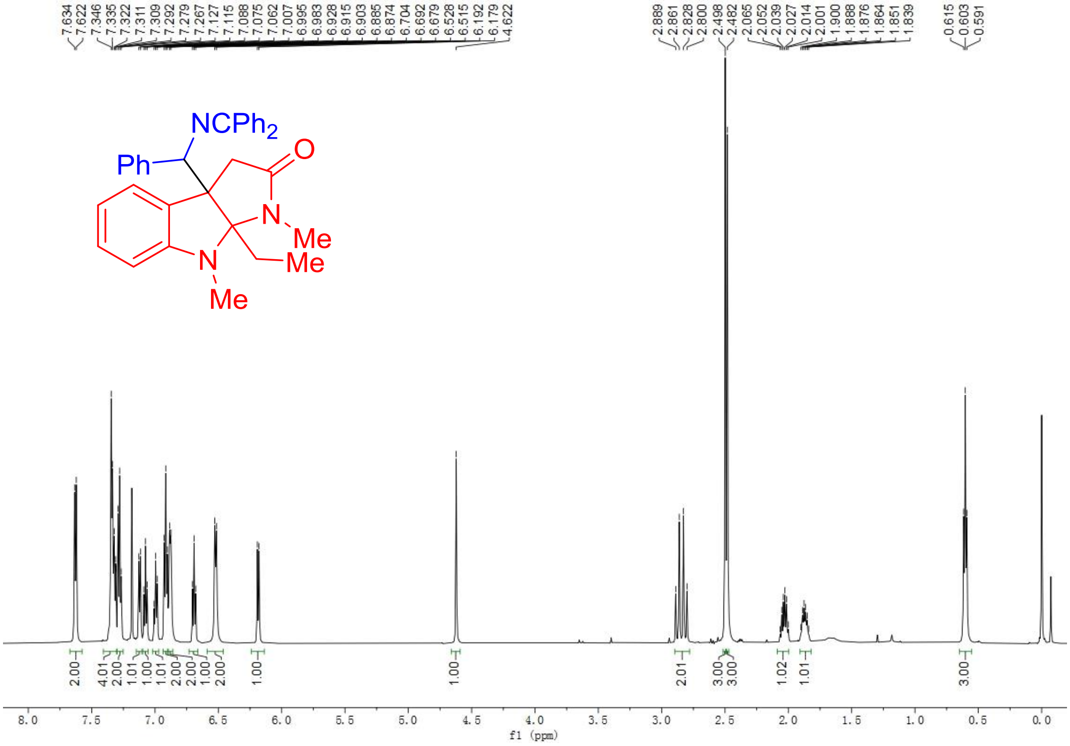

Figure S124. ^{13}C{^{1}H} NMR spectra (150 MHz, Chloroform-*d*) of 3a-(((diphenylmethylene)amino)(phenyl)methyl)-8a-ethyl-1,8-dimethyl-3,3a,8,8a-tetrahydropyrrolo[2,3-*b*]indol-2(1*H*)-one (3al(minor))

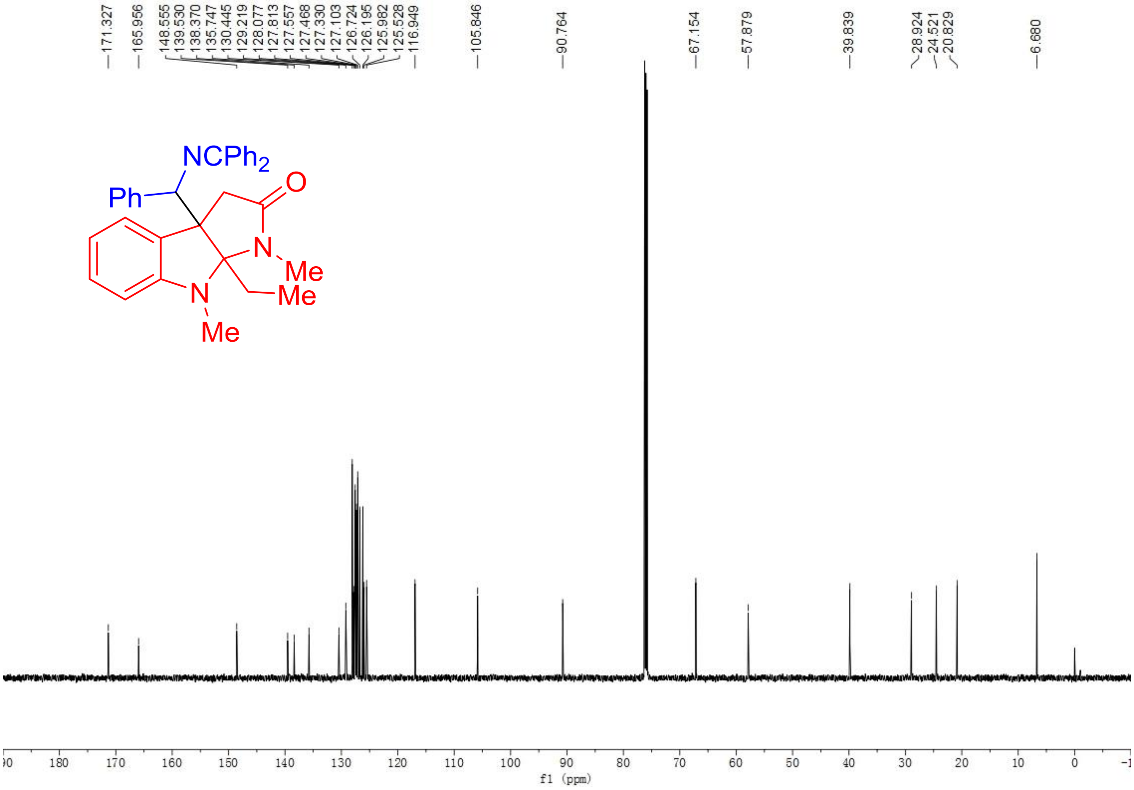

Figure S125. ^{1}H **NMR spectra (400 MHz, Chloroform-*d*) of 3a-(((9*H*-fluoren-9-ylidene)amino)(cyclohexyl)methyl)-8-benzyl-1-methyl-3,3a,8,8a-tetrahydropyrrolo[2,3-*b*]indol-2(1*H*)-one (3sm(major))**

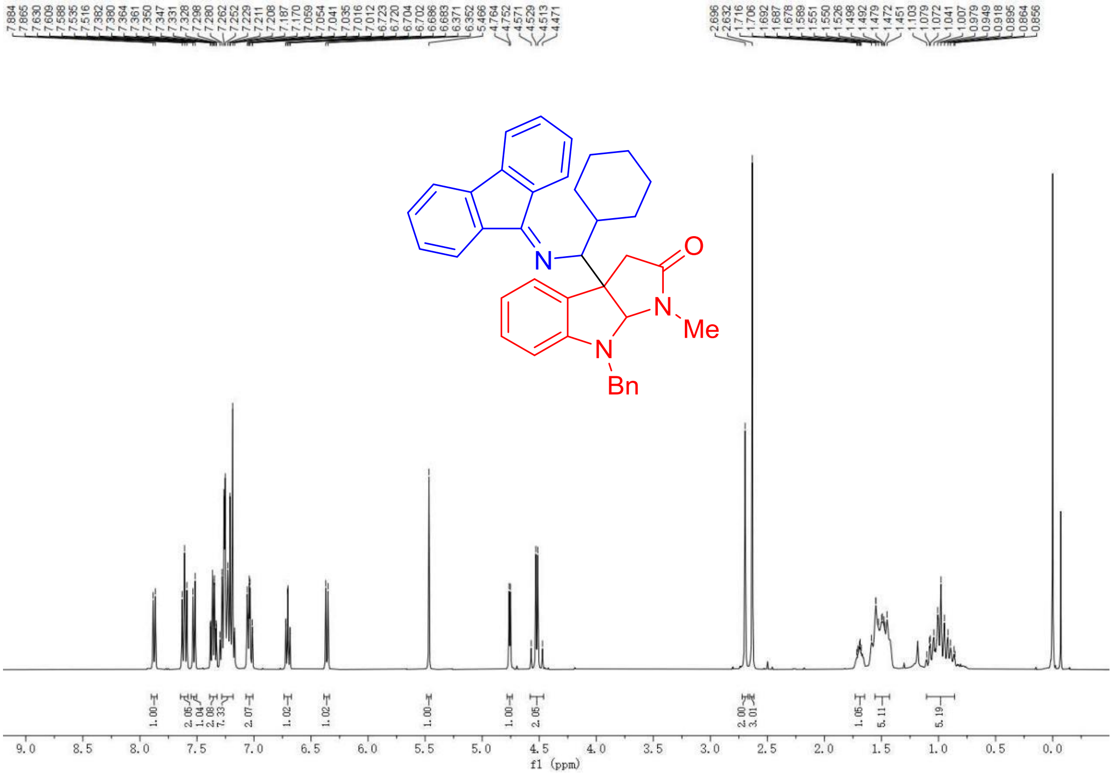

Figure S126. $^{13}C\{^{1}H\}$ **NMR spectra (100 MHz, Chloroform-*d*) of 3a-(((9*H*-fluoren-9-ylidene)amino)(cyclohexyl)methyl)-8-benzyl-1-methyl-3,3a,8,8a-tetrahydropyrrolo[2,3-*b*]indol-2(1*H*)-one (3sm(major))**

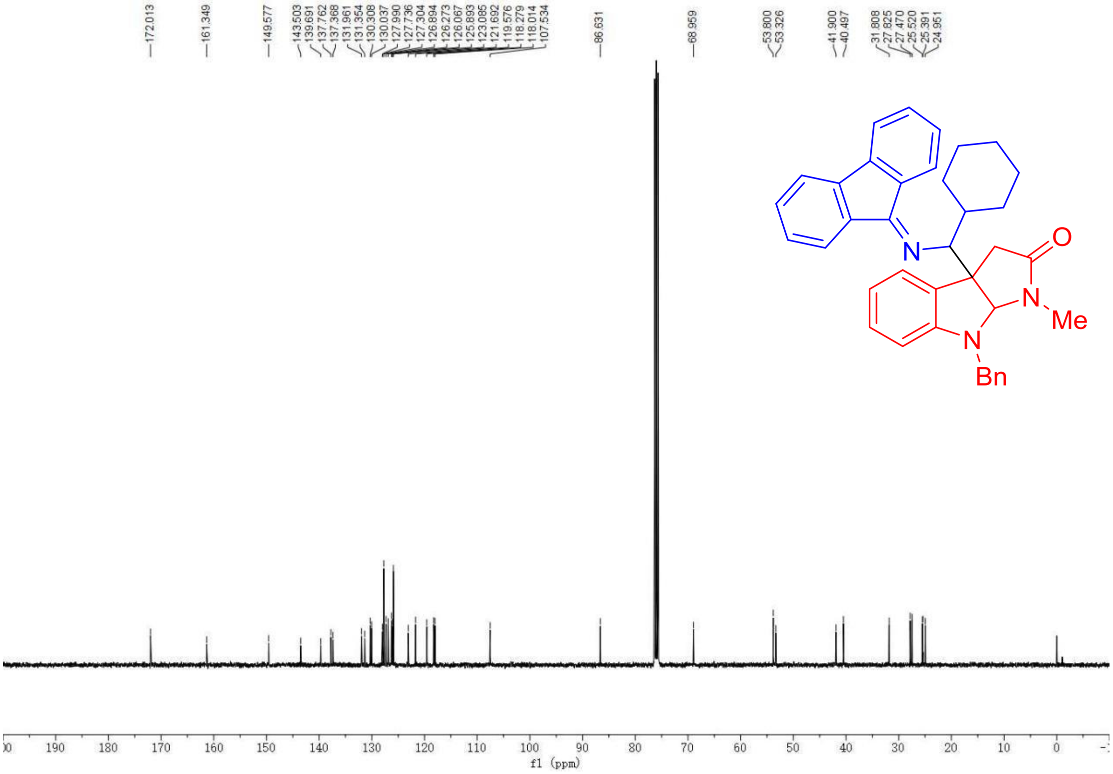

Figure S127. ^{1}H NMR spectra (400 MHz, Chloroform-*d*) of 3a-(((9*H*-fluoren-9-ylidene)amino)(cyclohexyl)methyl)-8-benzyl-1-methyl-3,3a,8,8a-tetrahydropyrrolo[2,3-*b*]indol-2(1*H*)-one (3sm(minor))

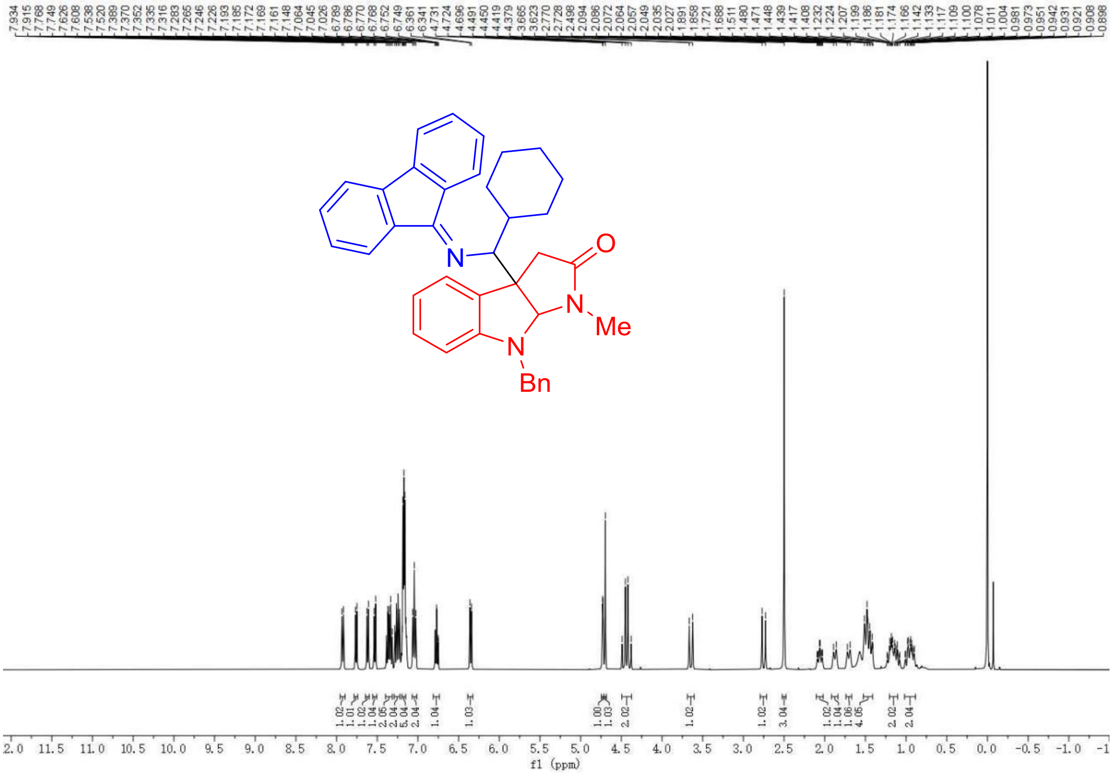

Figure S128. ^{13}C{^{1}H} NMR spectra (100 MHz, Chloroform-*d*) of 3a-(((9*H*-fluoren-9-ylidene)amino)(cyclohexyl)methyl)-8-benzyl-1-methyl-3,3a,8,8a-tetrahydropyrrolo[2,3-*b*]indol-2(1*H*)-one (3sm(minor))

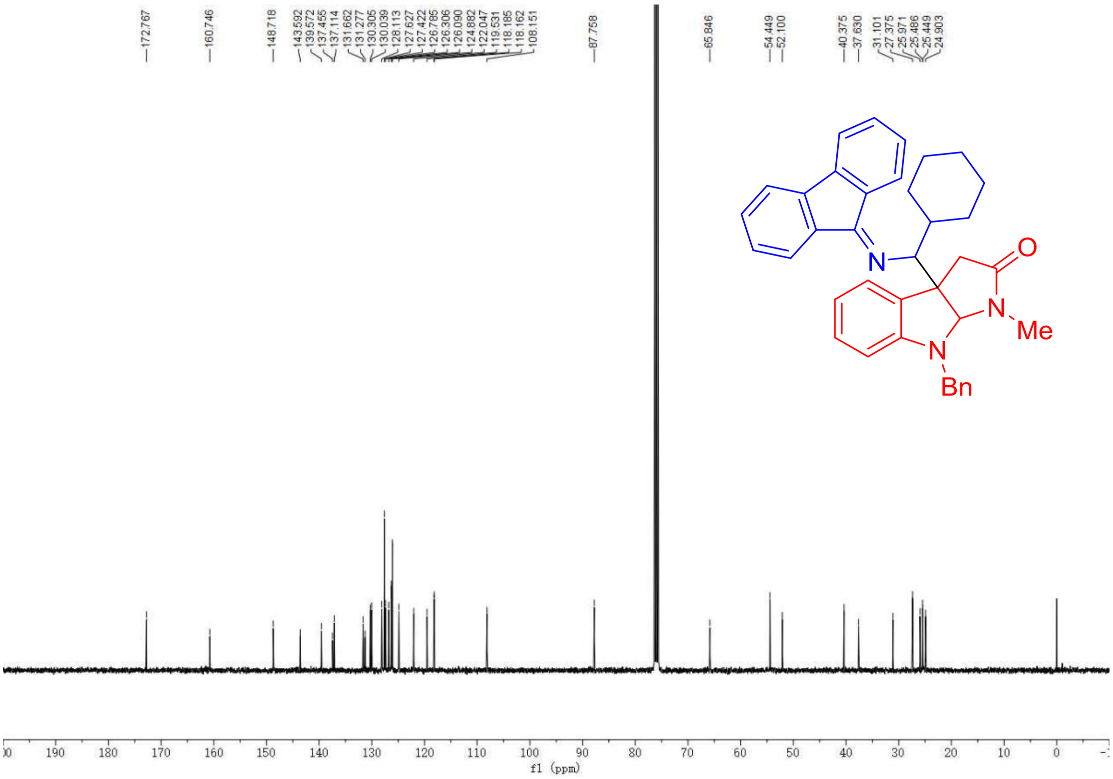

Figure S129. ^{1}H NMR spectra (400 MHz, Chloroform-*d*) of 3a-(((9*H*-fluoren-9-ylidene)amino)(cyclohexyl)methyl)-8-allyl-1-methyl-3,3a,8,8a-tetrahydropyrrolo[2,3-*b*]indol-2(1*H*)-one (3sn(major))

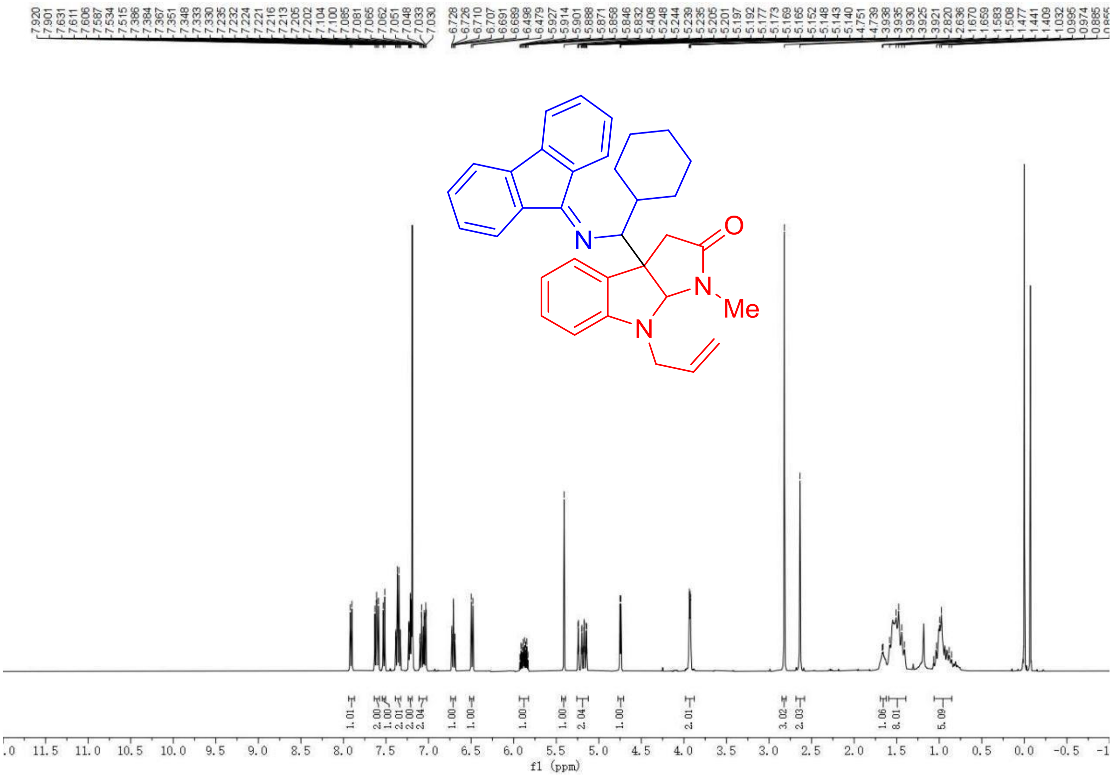

Figure S130. ^{13}C{^{1}H} NMR spectra (100 MHz, Chloroform-*d*) of 3a-(((9*H*-fluoren-9-ylidene)amino)(cyclohexyl)methyl)-8-allyl-1-methyl-3,3a,8,8a-tetrahydropyrrolo[2,3-*b*]indol-2(1*H*)-one (3sn(major))

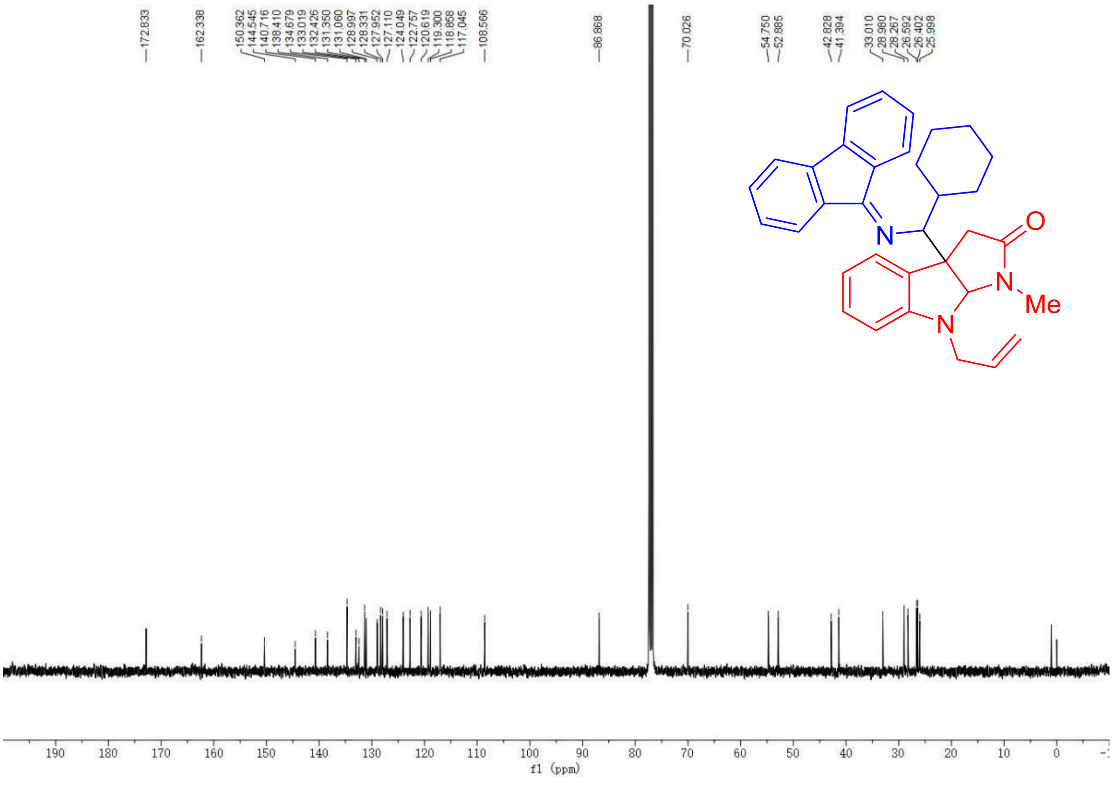

Figure S131. ^{1}**H NMR spectra (400 MHz, Chloroform-*d*) of 3a-(((9*H*-fluoren-9-ylidene)amino)(cyclohexyl)methyl)-8-allyl-1-methyl-3,3a,8,8a-tetrahydropyrrolo[2,3-*b*]indol-2(1*H*)-one (3sn(minor))**

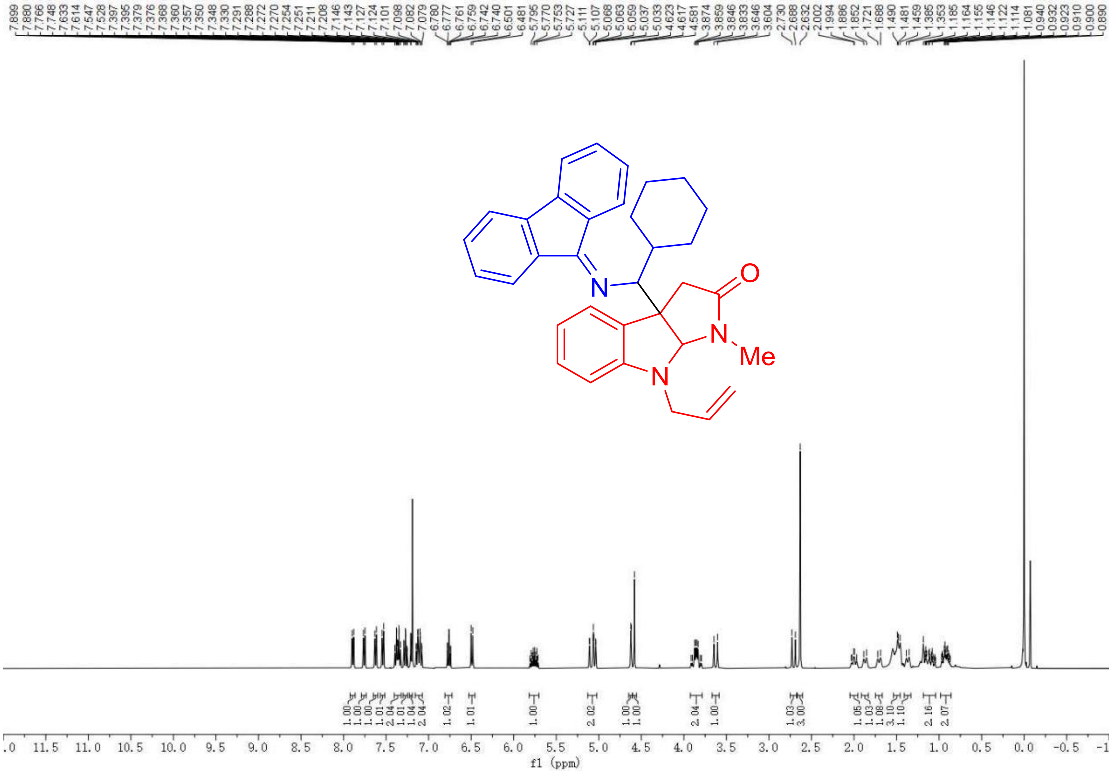

Figure S132. ^{13}**C{**^{1}**H} NMR spectra (100 MHz, Chloroform-*d*) of 3a-(((9*H*-fluoren-9-ylidene)amino)(cyclohexyl)methyl)-8-allyl-1-methyl-3,3a,8,8a-tetrahydropyrrolo[2,3-*b*]indol-2(1*H*)-one (3sn(minor))**

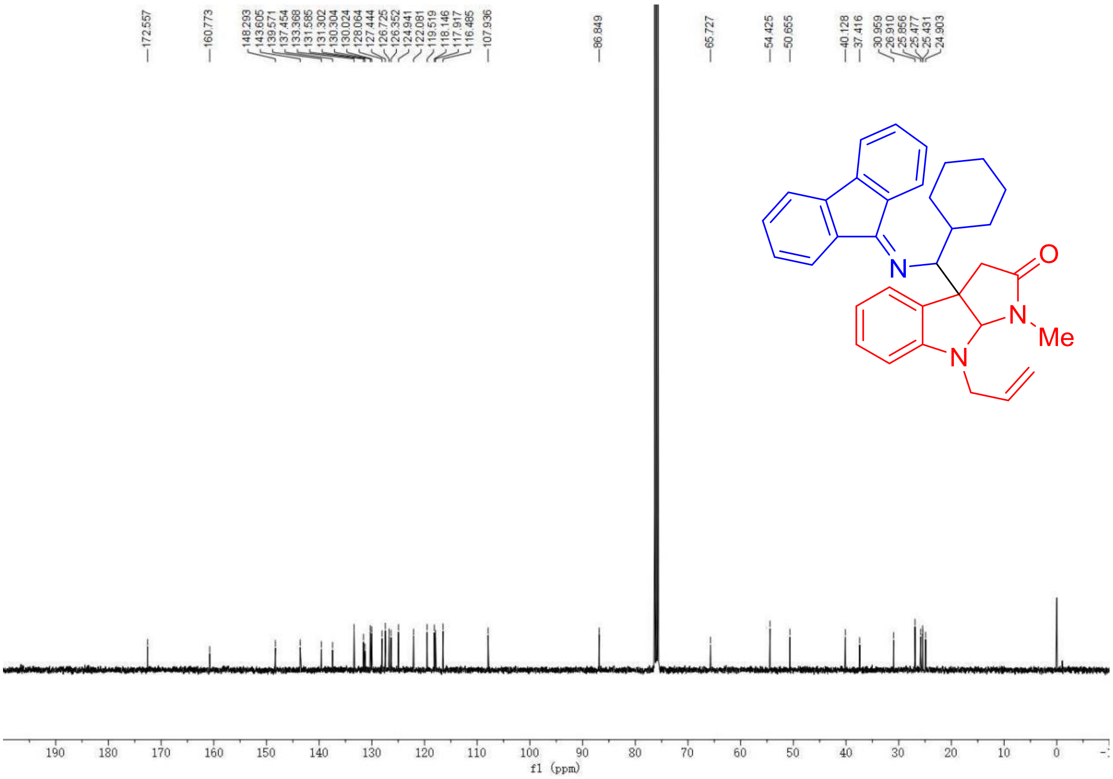

Figure S133. ^{1}H NMR spectra (400 MHz, Chloroform-*d*) of tert-butyl 3a-(((9*H*-fluoren-9-ylidene)amino)(cyclohexyl)methyl)-8-methyl-2-oxo-3,3a,8,8a-tetrahydropyrrolo[2,3-*b*]indole-1(2*H*)-carboxylate (3so(major))

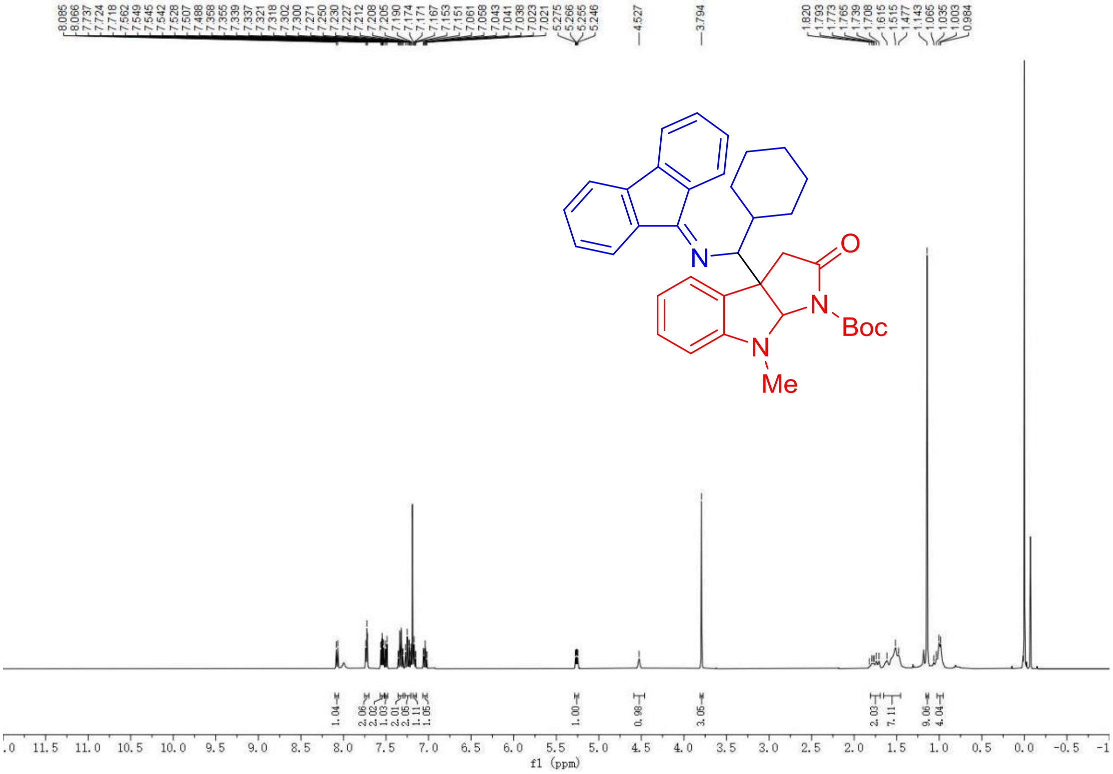

Figure S134. ^{13}C{^{1}H} NMR spectra (100 MHz, Chloroform-*d*) of tert-butyl 3a-(((9*H*-fluoren-9-ylidene)amino)(cyclohexyl)methyl)-8-methyl-2-oxo-3,3a,8,8a-tetrahydropyrrolo[2,3-*b*]indole-1(2*H*)-carboxylate (3so(major))

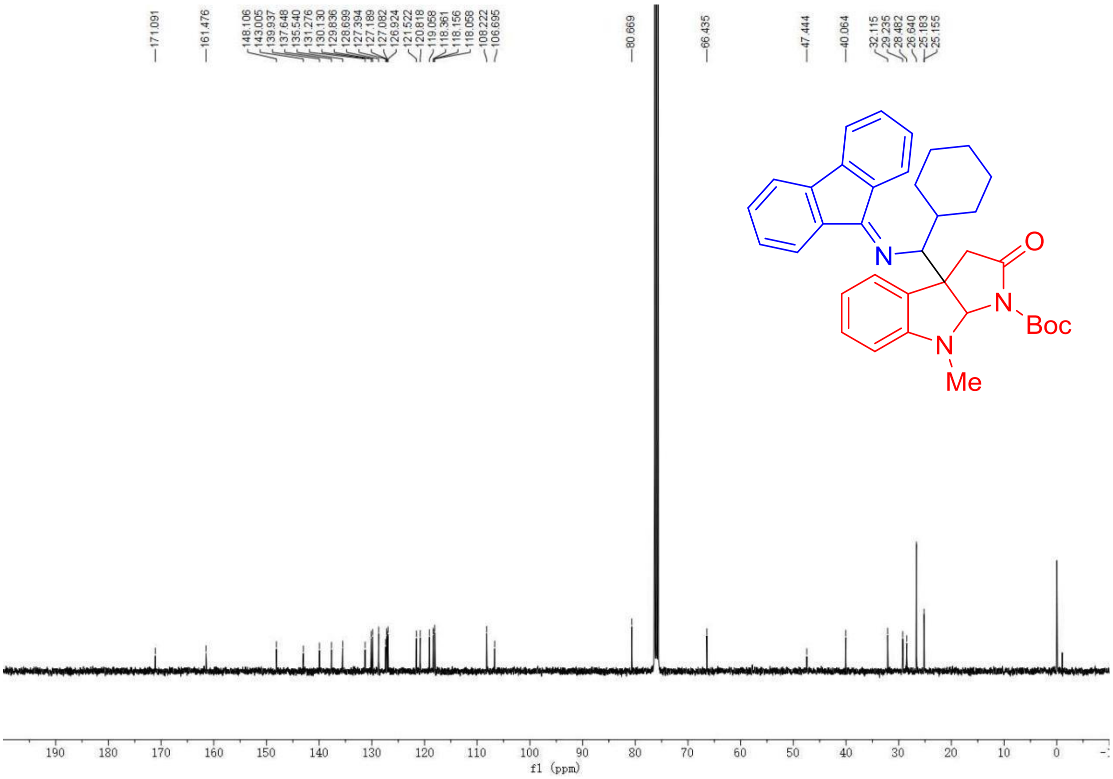

Figure S135. ^{1}H **NMR spectra (600 MHz, Chloroform-*d*) of tert-butyl 3a-(((9*H*-fluoren-9-ylidene)amino)(cyclohexyl)methyl)-8-methyl-2-oxo-3,3a,8,8a-tetrahydropyrrolo[2,3-*b*]indole-1(2*H*)-carboxylate (3so(minor))**

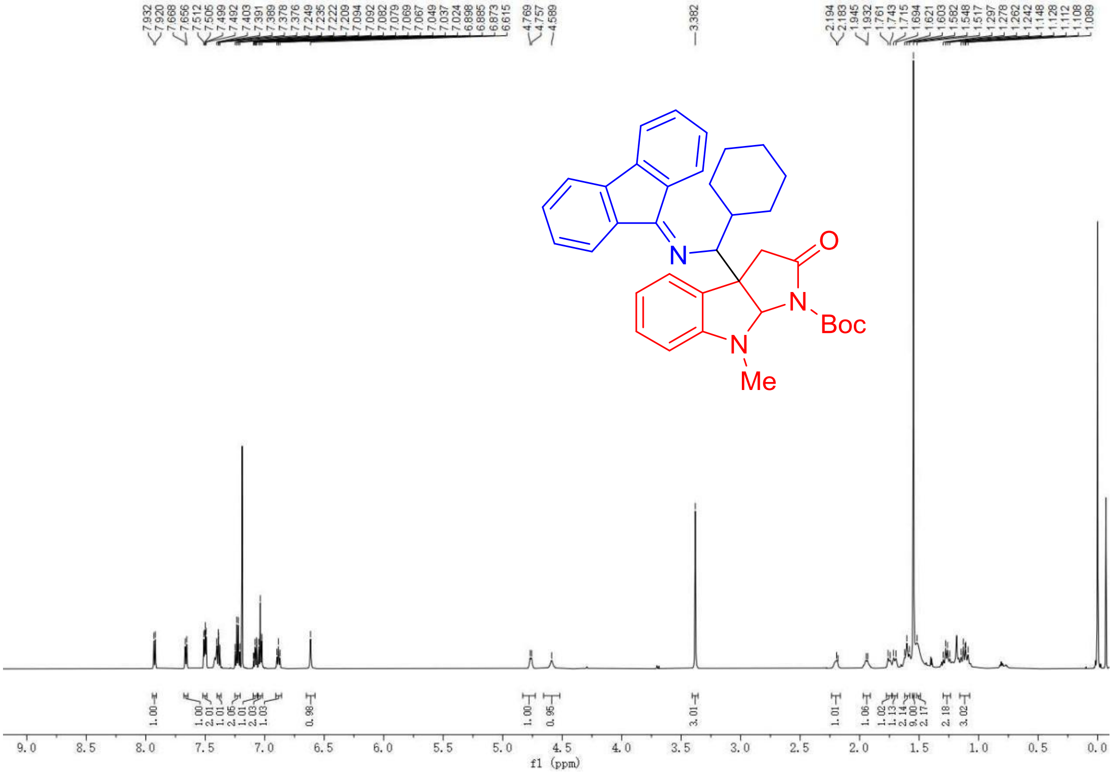

Figure S136. $^{13}C\{^{1}H\}$ **NMR spectra (150 MHz, Chloroform-*d*) of tert-butyl 3a-(((9*H*-fluoren-9-ylidene)amino)(cyclohexyl)methyl)-8-methyl-2-oxo-3,3a,8,8a-tetrahydropyrrolo[2,3-*b*]indole-1(2*H*)-carboxylate (3so(minor))**

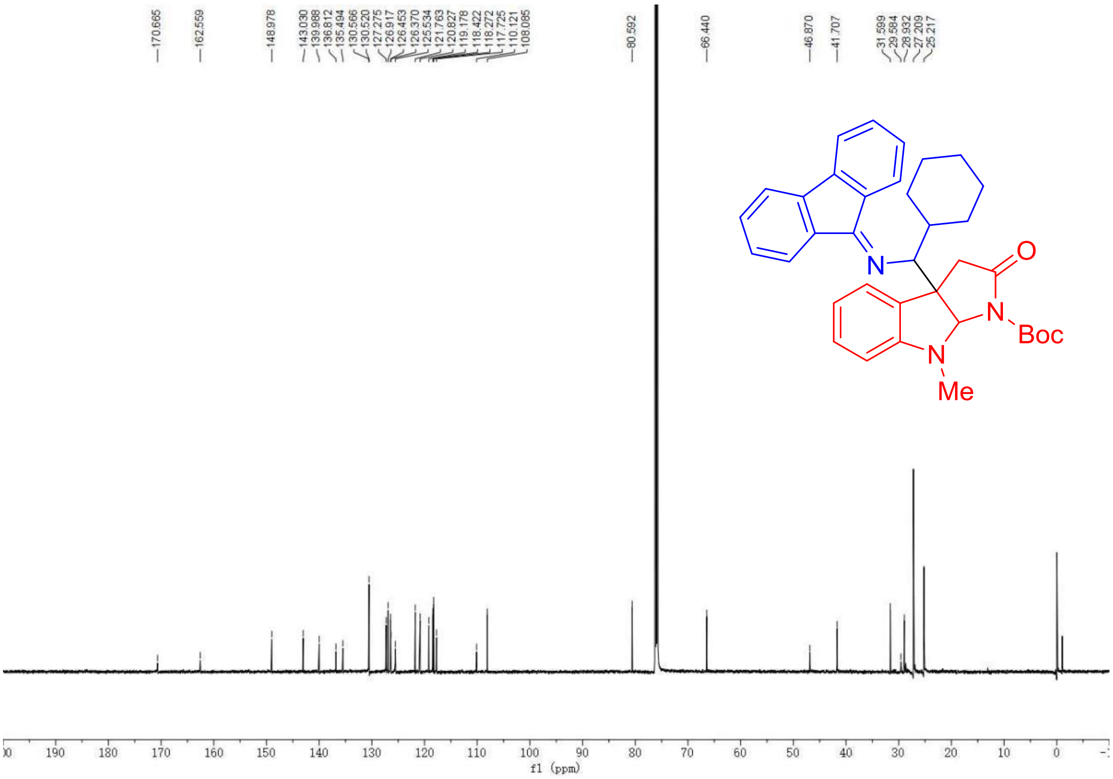

Figure S137. ^{1}H NMR spectra (600 MHz, Methanol-*d*$_4$) 3a-(amino(phenyl)methyl)-1,8-dimethyl-3,3a,8,8a-tetrahydropyrrolo[2,3-*b*]indol-2(1*H*)-one (4aa)

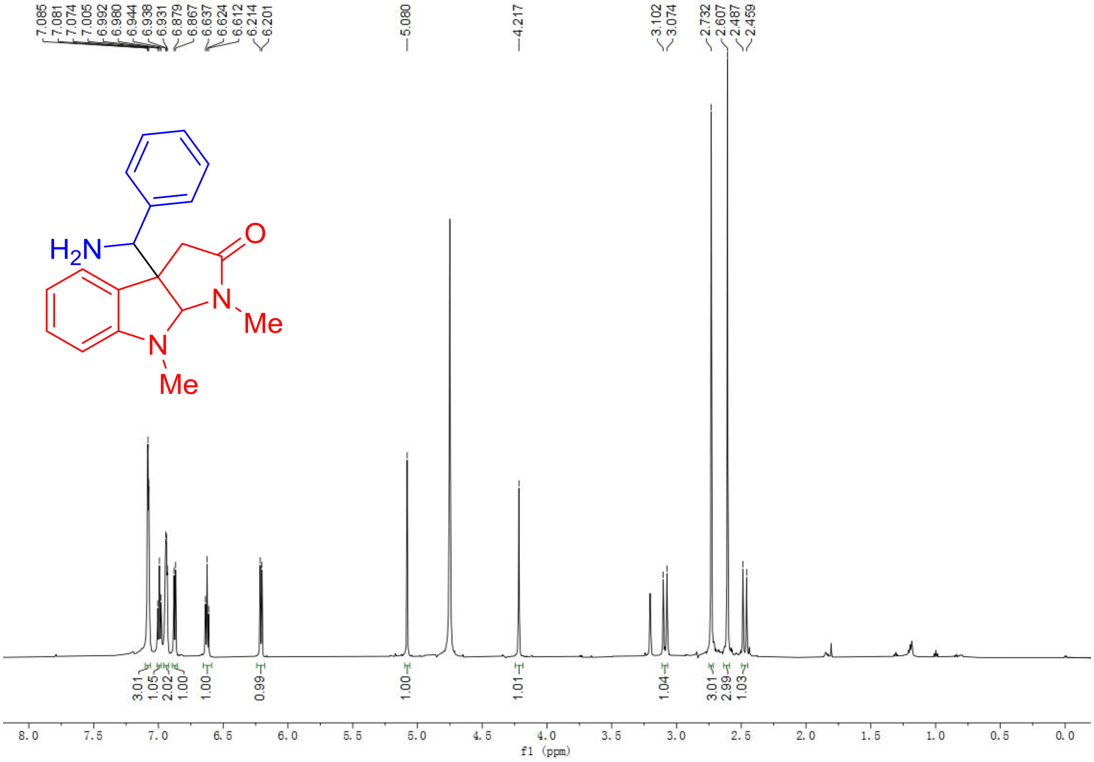

Figure S138. ^{13}C{^{1}H} NMR spectra (150 MHz, Methanol-*d*$_4$) 3a-(amino(phenyl)methyl)-1,8-dime thyl -3,3a,8,8a-tetrahydropyrrolo[2,3-*b*]indol-2(1*H*)-one (4aa)

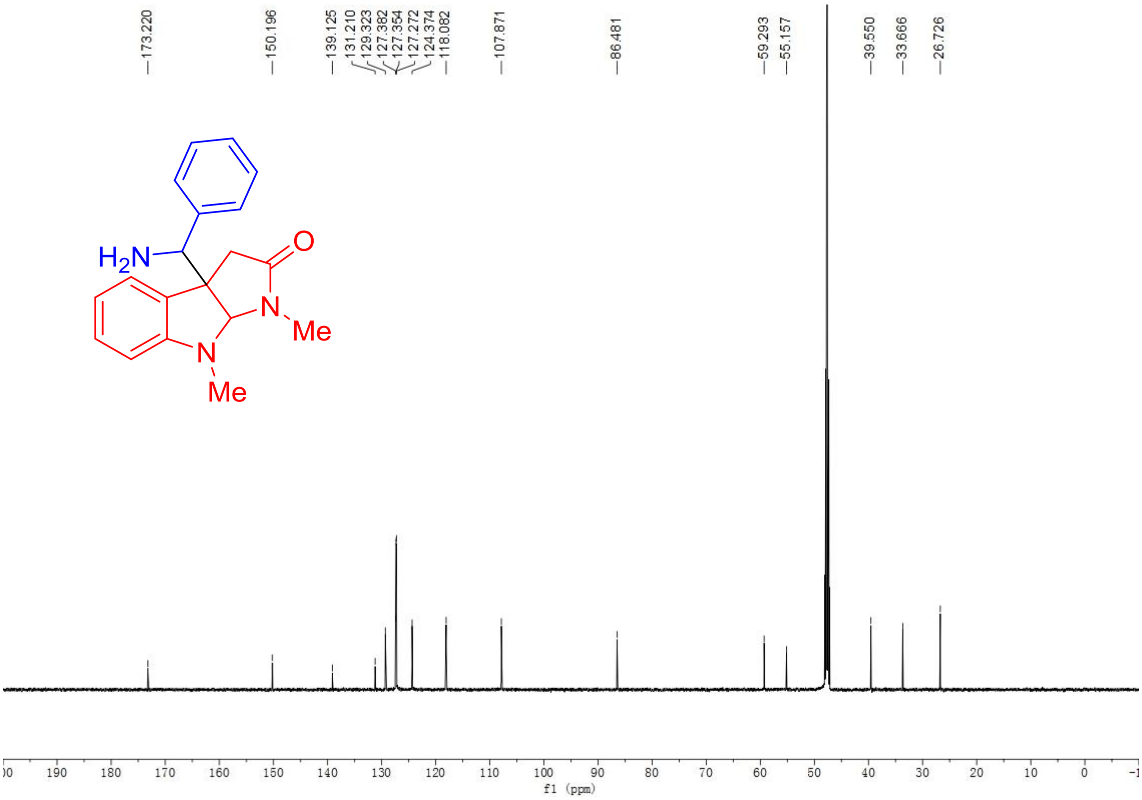

Figure S139. ^{1}**H NMR spectra (400 MHz, Chloroform-*d*) 1,8-dimethyl-3a-((2,2,6,6-tetramethylpiperidin-1-yl)oxy)-3,3a,8,8a-tetrahydropyrrolo[2,3-*b*]indol-2(1*H*)-one (5aa)**

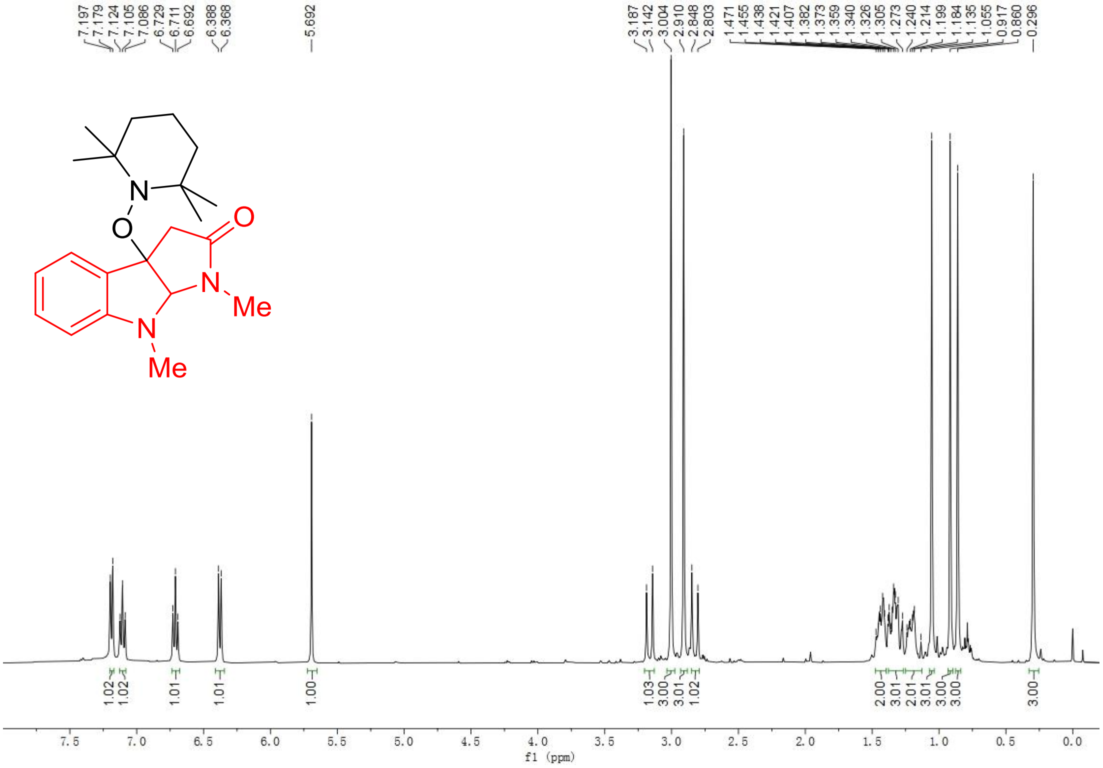

Figure S140. ^{13}**C{**^{1}**H} NMR spectra (150 MHz, Chloroform-*d*) 1,8-dimethyl-3a-((2,2,6,6-tetramethylpiperidin-1-yl)oxy)-3,3a,8,8a-tetrahydropyrrolo[2,3-*b*]indol-2(1*H*)-one (5aa)**

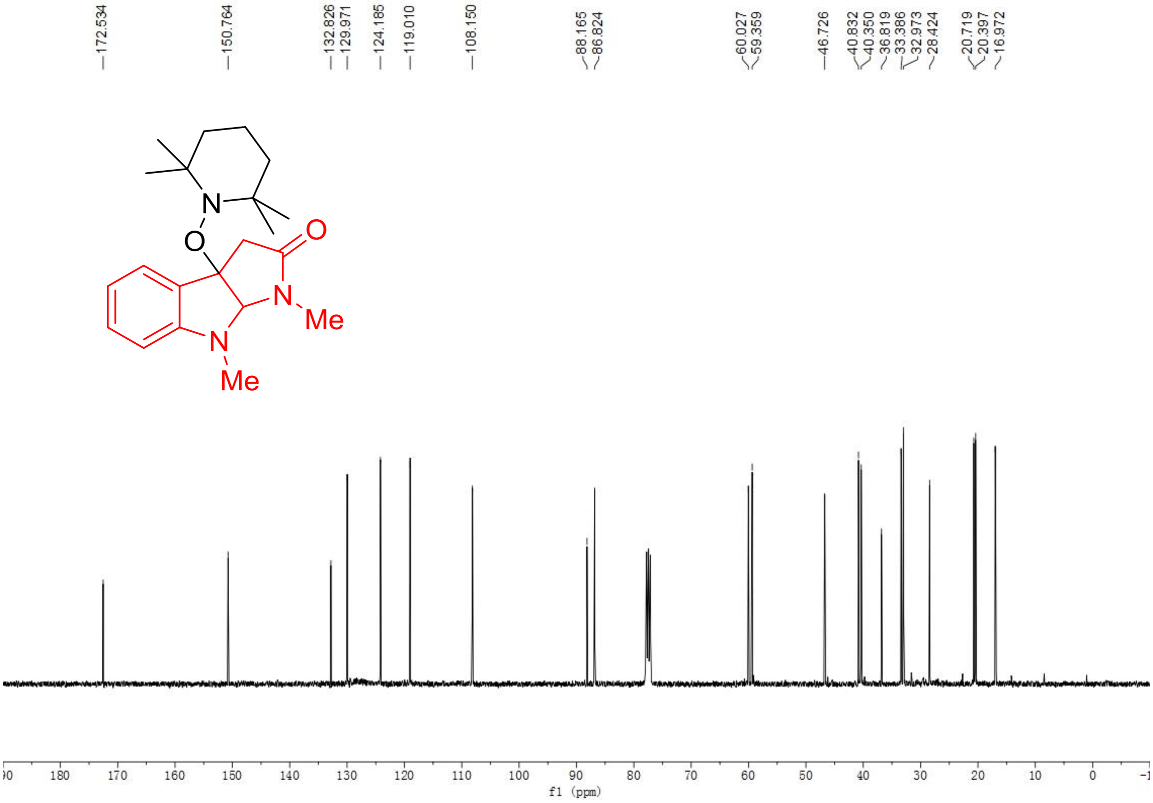

Figure S141. ^{1}H **NMR spectra (600 MHz, Chloroform-*d*) 1,1-diphenyl-*N*-(phenyl((2,2,6,6-tetrame thylpiperidin-1-yl)oxy)methyl)methanimine (6aa)**

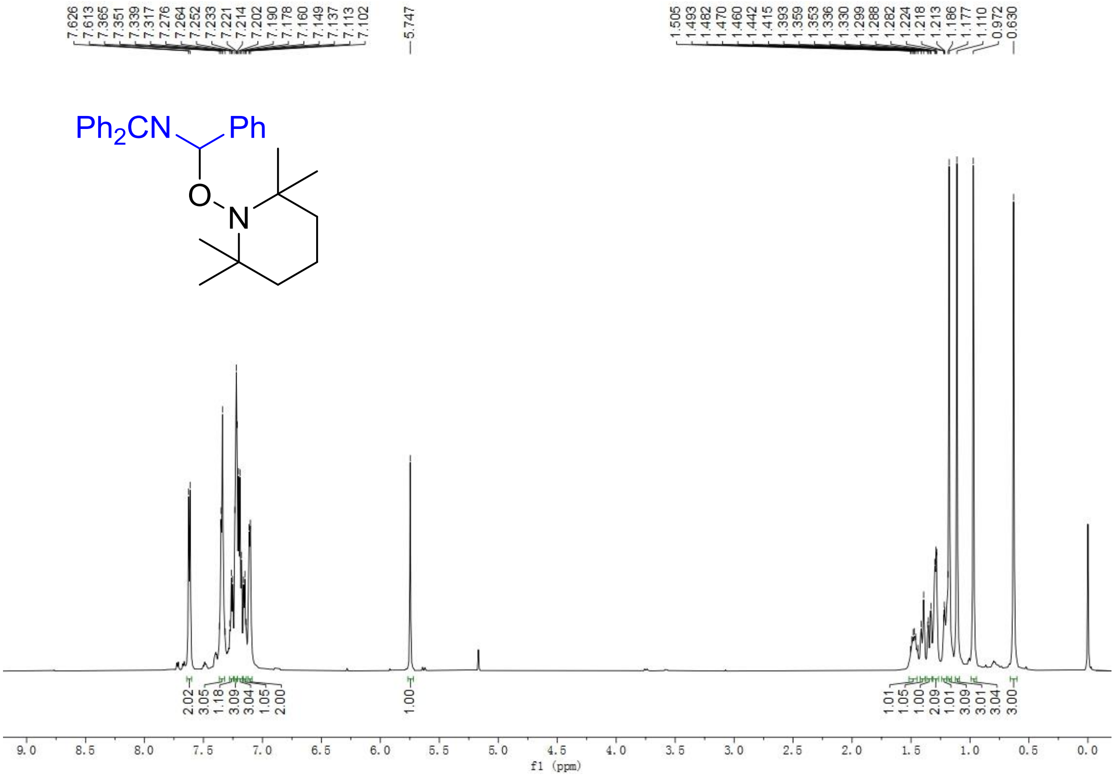

Figure S142. $^{13}C\{^{1}H\}$ **NMR spectra (150 MHz, Chloroform-*d*) 1,1-diphenyl-*N*-(phenyl((2, 2,6,6-tetrame thylpiperidin-1-yl)oxy)methyl)methanimine (6aa)**

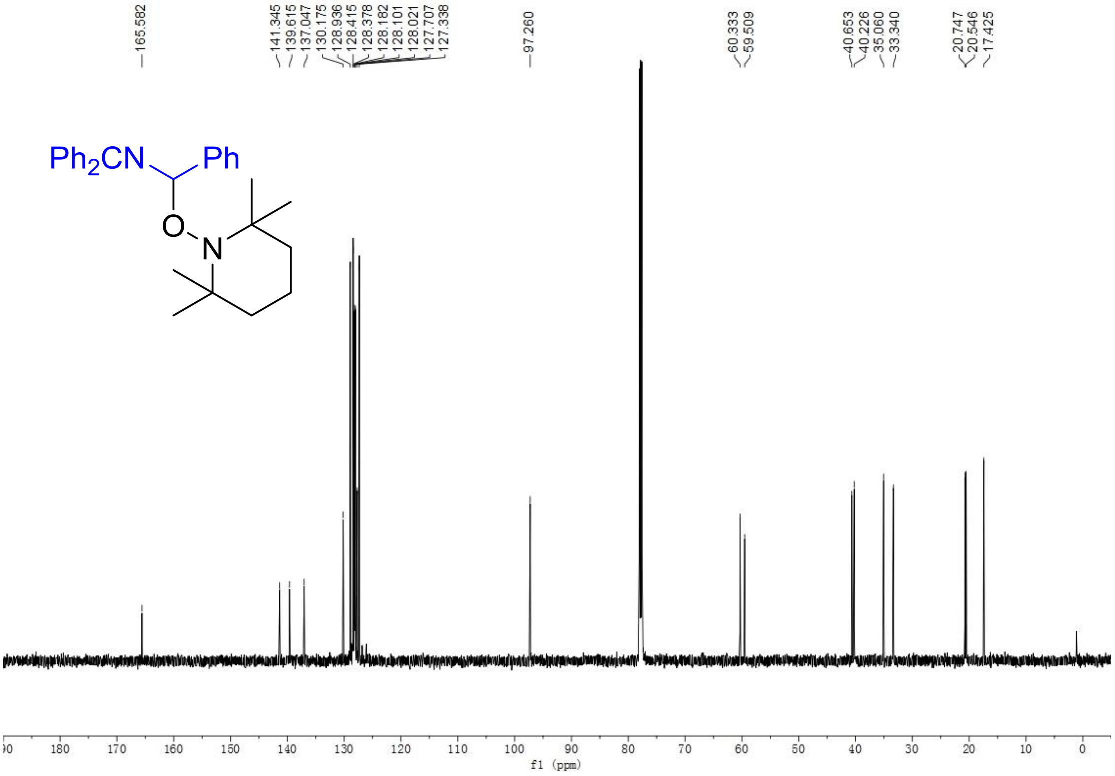

HRMS data

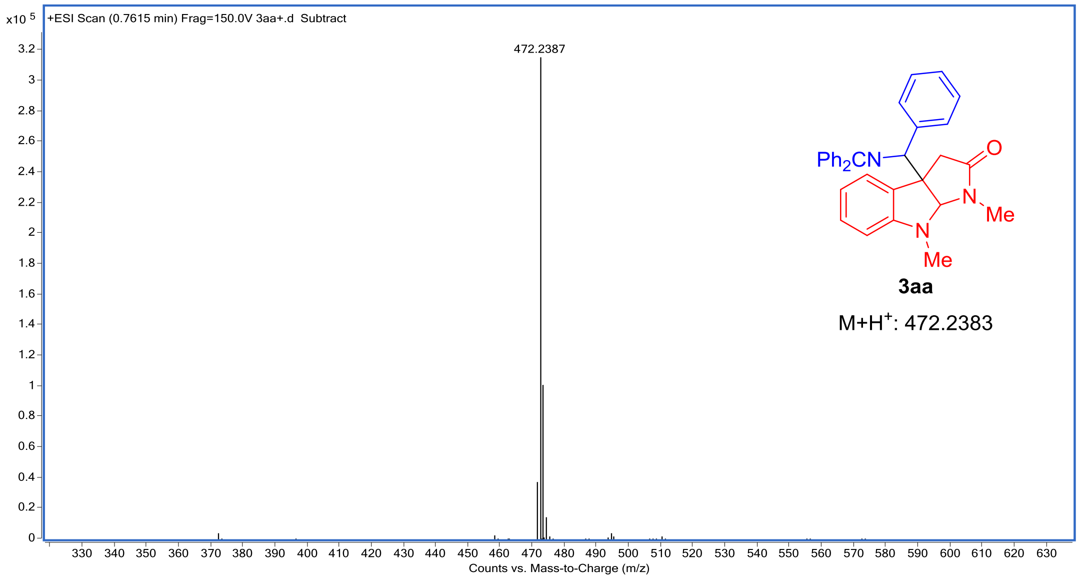
x10 5
+ESI Scan (0.7615 min) Frag=150.0V 3aa+.d Subtract
472.2387
Ph2CN
O
N
Me
N
Me
3aa
M+H+: 472.2383
Counts vs. Mass-to-Charge (m/z)

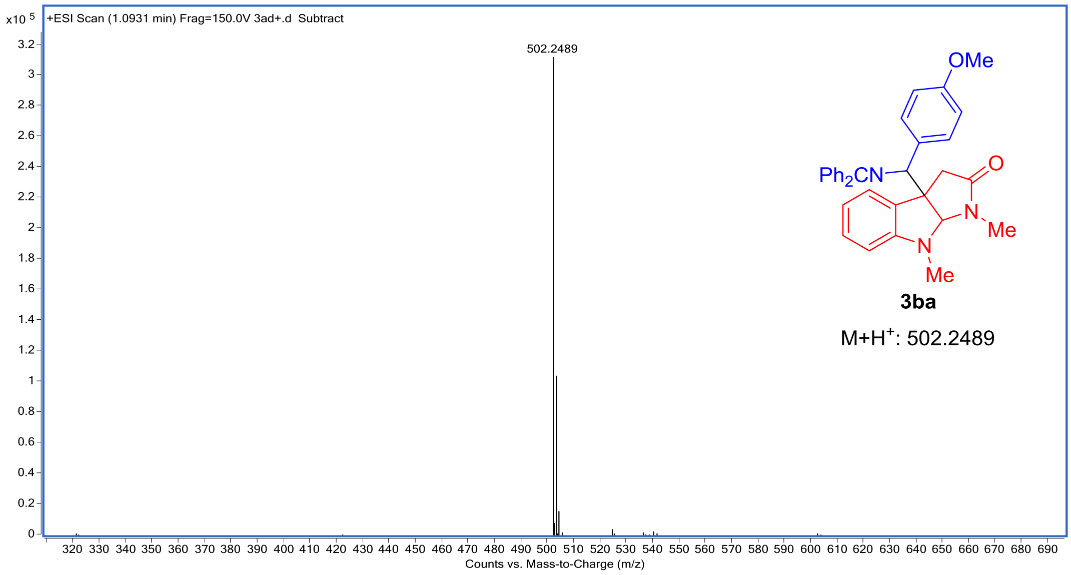
x10 5
+ESI Scan (1.0931 min) Frag=150.0V 3ad+.d Subtract
502.2489
OMe
Ph2CN
O
N
Me
N
Me
3ba
M+H+: 502.2489
Counts vs. Mass-to-Charge (m/z)

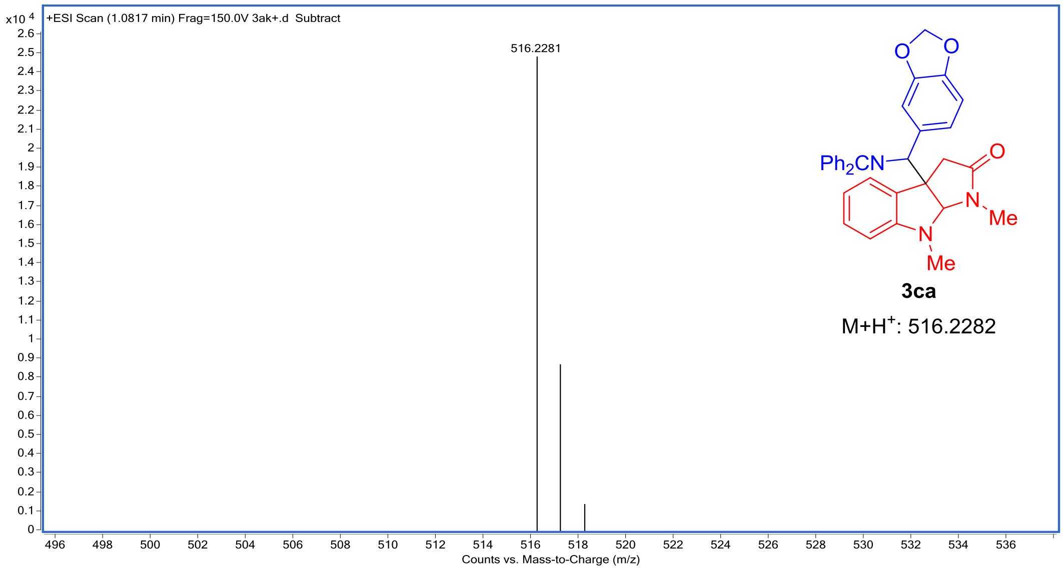
x10 4
+ESI Scan (1.0817 min) Frag=150.0V 3ak+.d Subtract
516.2281
O
O
Ph2CN
O
N
Me
N
Me
3ca
M+H+: 516.2282
Counts vs. Mass-to-Charge (m/z)

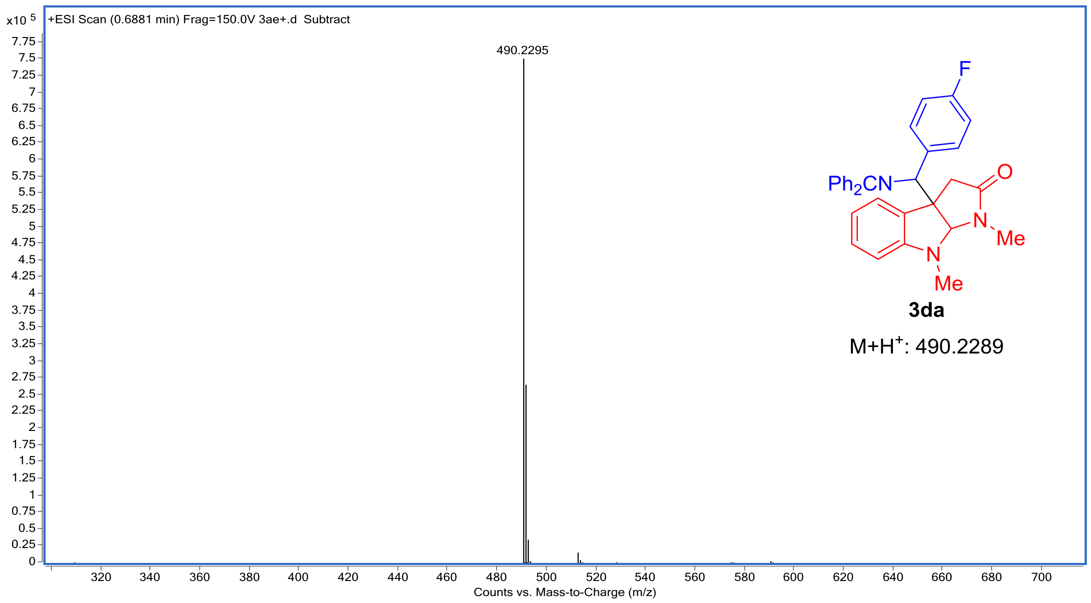
x10 5
+ESI Scan (0.6881 min) Frag=150.0V 3ae+.d Subtract
490.2295
F
Ph2CN
O
N
Me
N
Me
3da
M+H+: 490.2289
Counts vs. Mass-to-Charge (m/z)

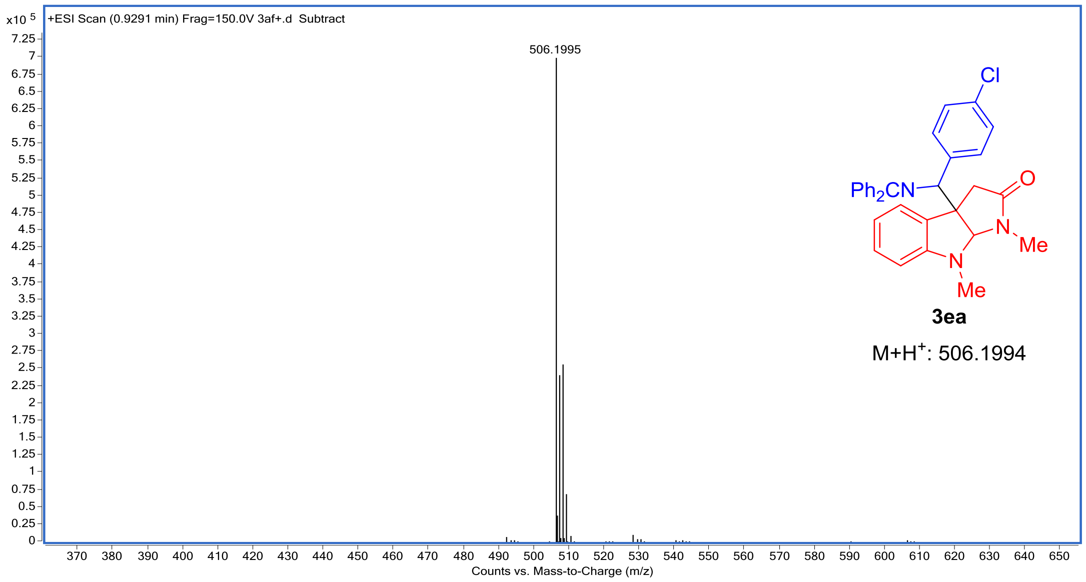
x10 5
+ESI Scan (0.9291 min) Frag=150.0V 3af+.d Subtract
506.1995
Cl
Ph2CN
O
N
Me
N
Me
3ea
M+H+: 506.1994
Counts vs. Mass-to-Charge (m/z)

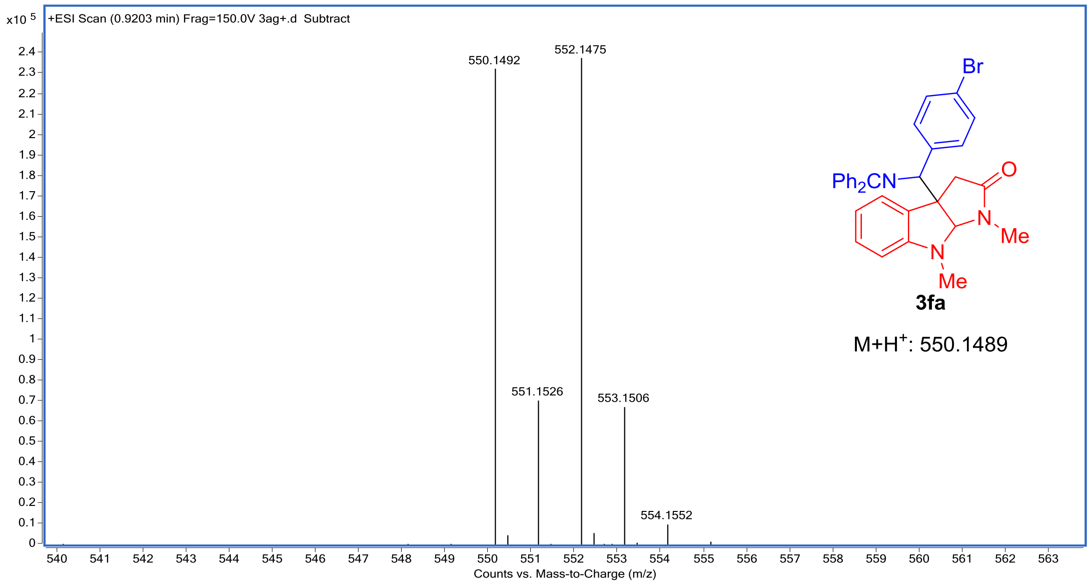
x10 5
+ESI Scan (0.9203 min) Frag=150.0V 3ag+.d Subtract
550.1492
552.1475
551.1526
553.1506
554.1552
Br
Ph2CN
O
N
Me
N
Me
3fa
M+H+: 550.1489
Counts vs. Mass-to-Charge (m/z)

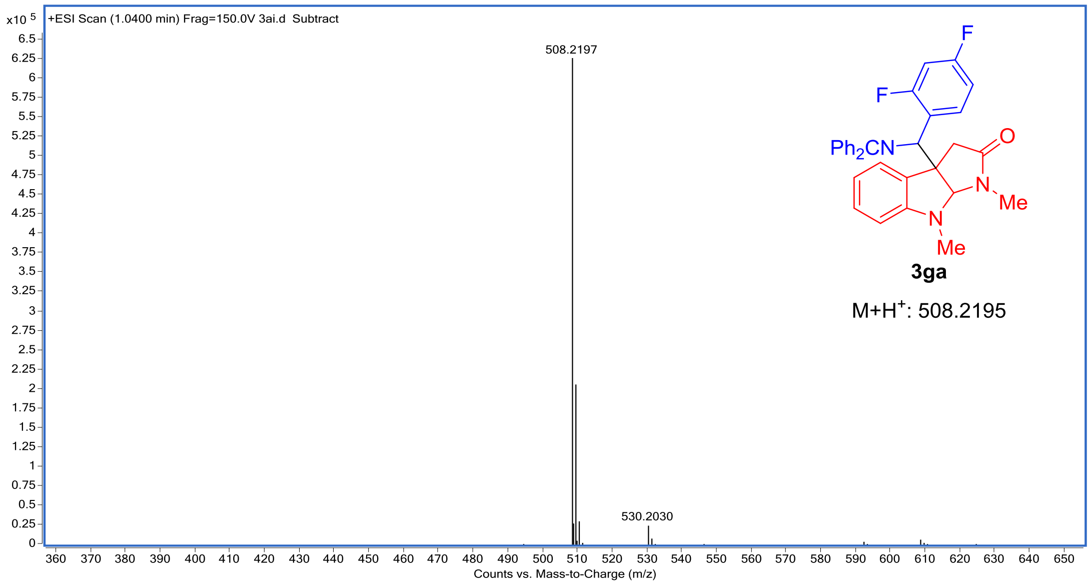

x10 5
+ESI Scan (1.0400 min) Frag=150.0V 3ai.d Subtract
508.2197
530.2030
Counts vs. Mass-to-Charge (m/z)
Ph2CN
F
F
O
N
Me
N
Me
3ga
M+H+: 508.2195

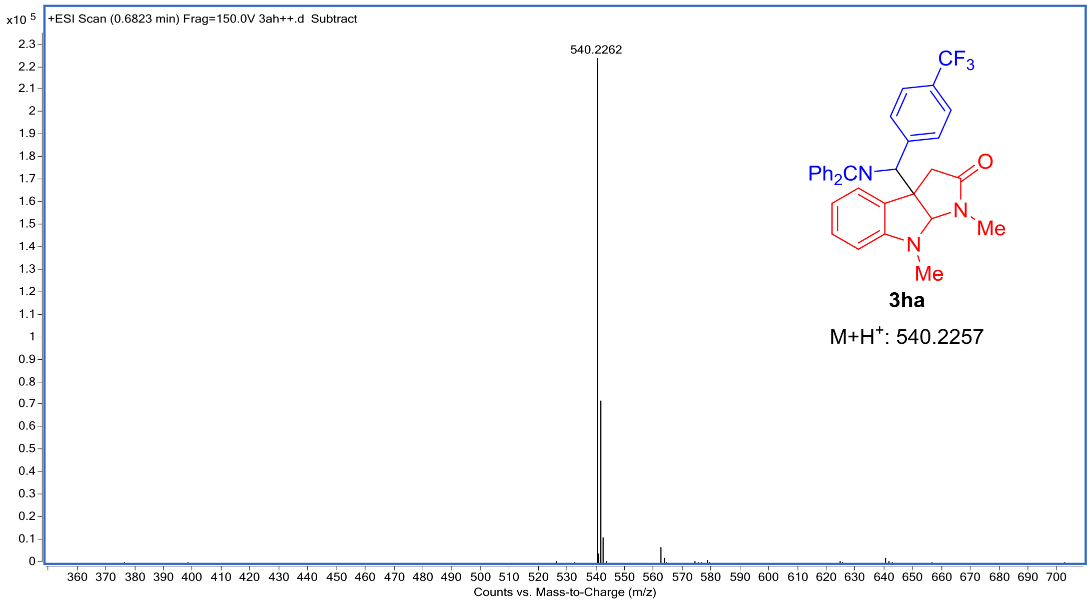

x10 5
+ESI Scan (0.6823 min) Frag=150.0V 3ah++.d Subtract
540.2262
Counts vs. Mass-to-Charge (m/z)
CF3
Ph2CN
O
N
Me
N
Me
3ha
M+H+: 540.2257

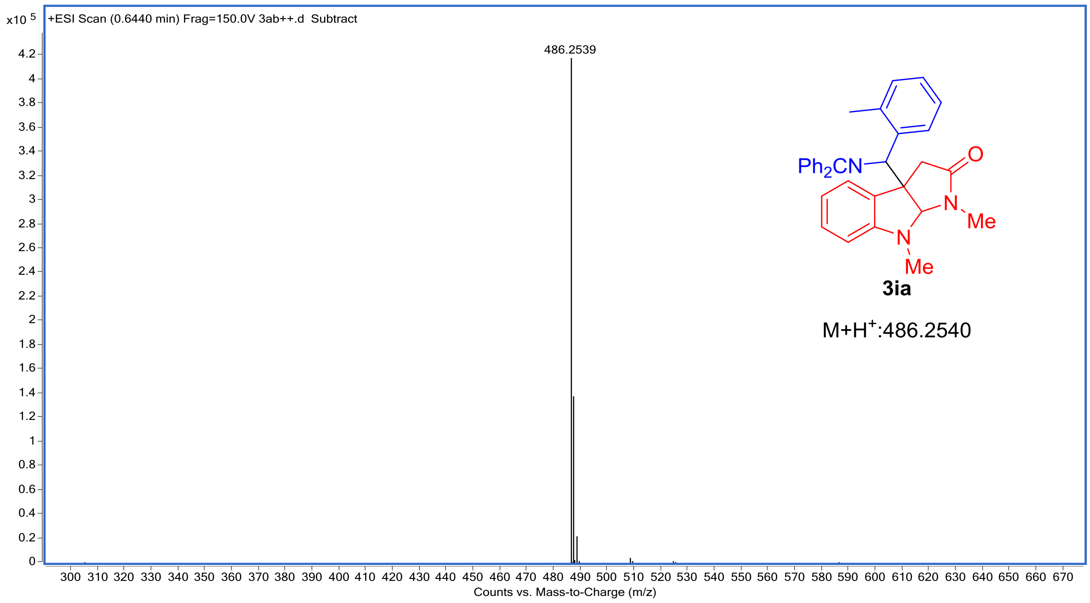

x10 5
+ESI Scan (0.6440 min) Frag=150.0V 3ab++.d Subtract
486.2539
Counts vs. Mass-to-Charge (m/z)
Ph2CN
O
N
Me
N
Me
3ia
M+H+:486.2540

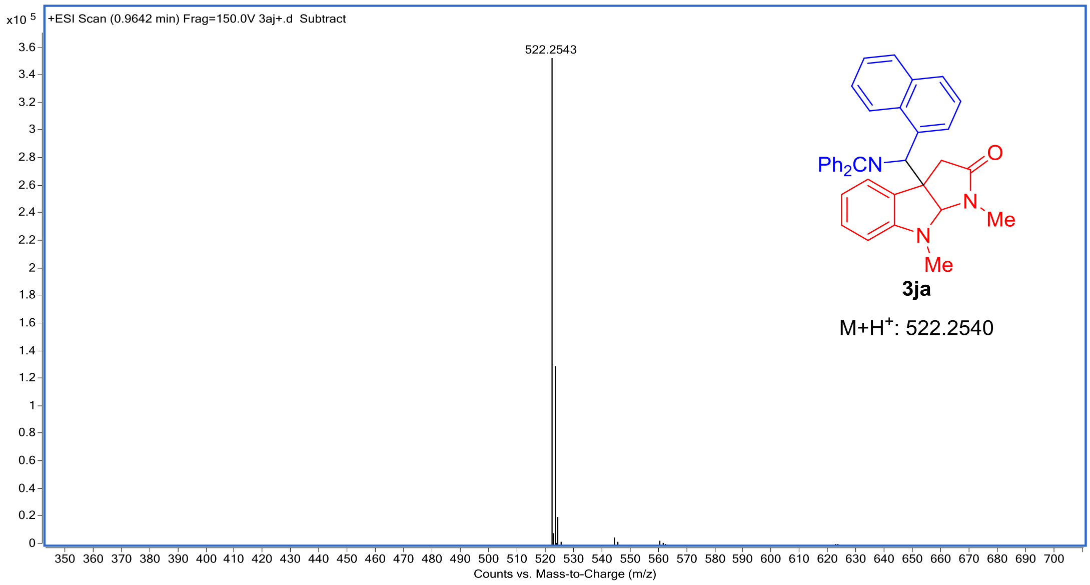
x10 5
+ESI Scan (0.9642 min) Frag=150.0V 3aj+.d Subtract
522.2543
Ph2CN
N
Me
O
3ja
M+H+: 522.2540
Counts vs. Mass-to-Charge (m/z)

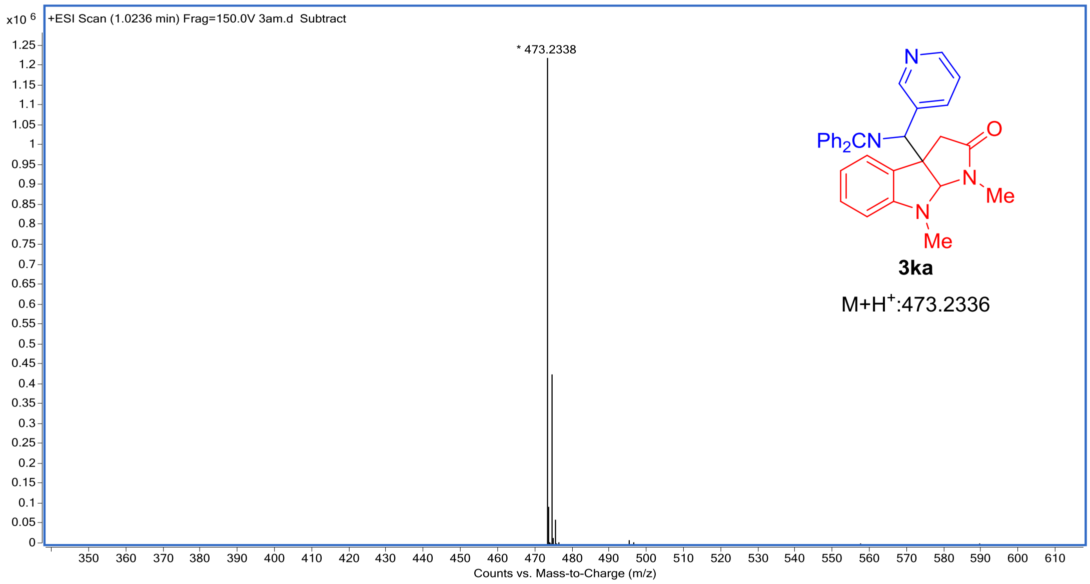
x10 6
+ESI Scan (1.0236 min) Frag=150.0V 3am.d Subtract
* 473.2338
Ph2CN
N
Me
O
3ka
M+H+:473.2336
Counts vs. Mass-to-Charge (m/z)

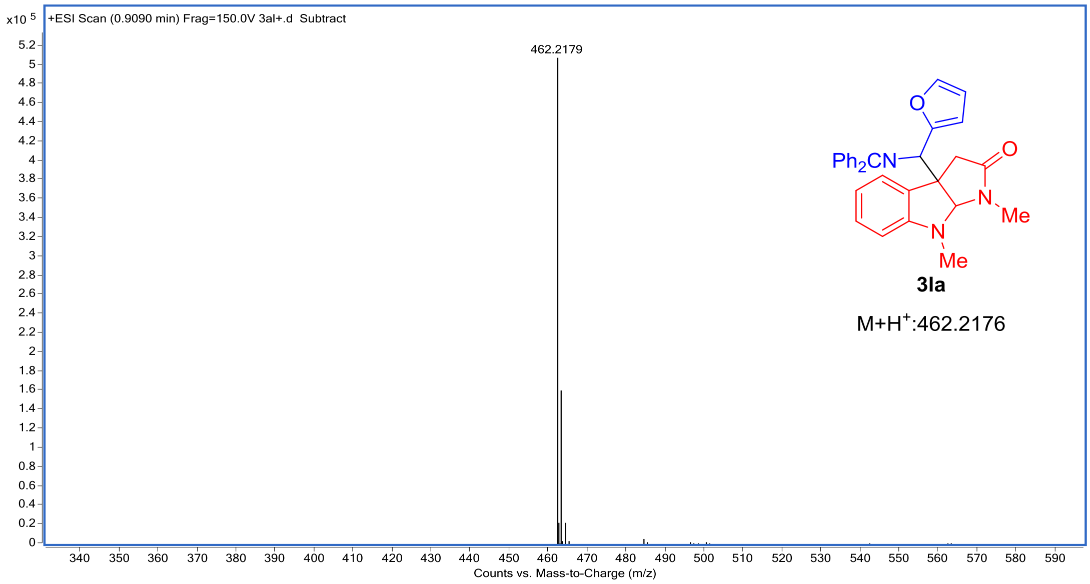
x10 5
+ESI Scan (0.9090 min) Frag=150.0V 3al+.d Subtract
462.2179
Ph2CN
N
Me
O
3la
M+H+:462.2176
Counts vs. Mass-to-Charge (m/z)

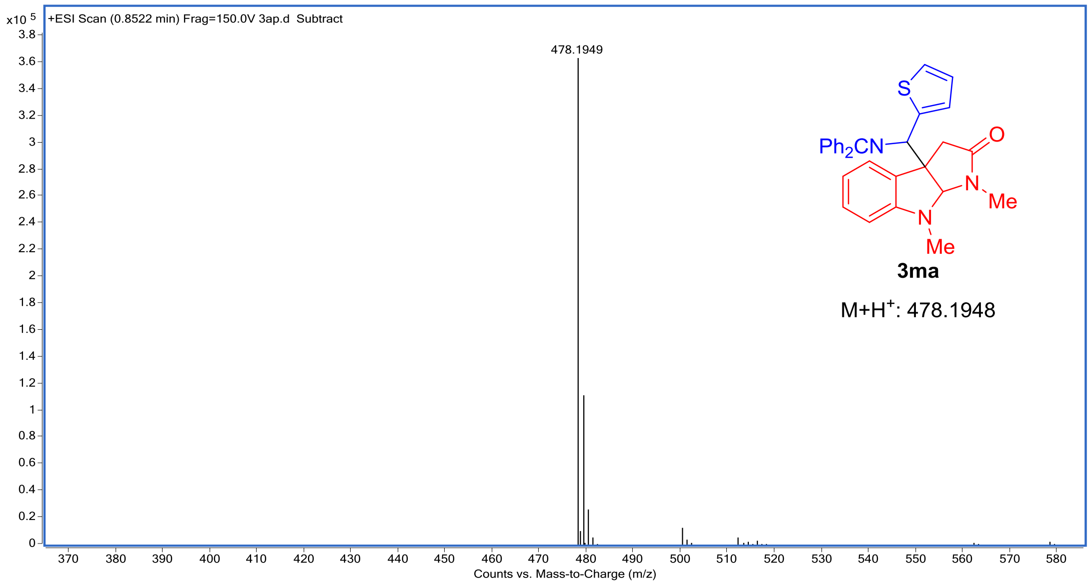
x10 5
+ESI Scan (0.8522 min) Frag=150.0V 3ap.d Subtract
478.1949
Ph2CN
S
O
N
Me
N
Me
3ma
M+H+: 478.1948
Counts vs. Mass-to-Charge (m/z)

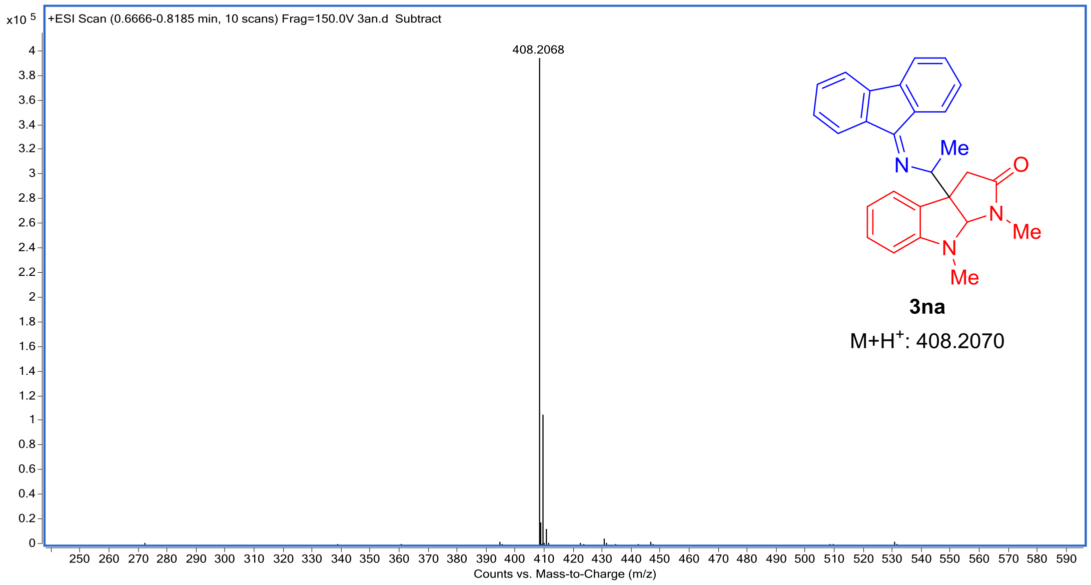
x10 5
+ESI Scan (0.6666-0.8185 min, 10 scans) Frag=150.0V 3an.d Subtract
408.2068
Me
N
O
N
Me
N
Me
3na
M+H+: 408.2070
Counts vs. Mass-to-Charge (m/z)

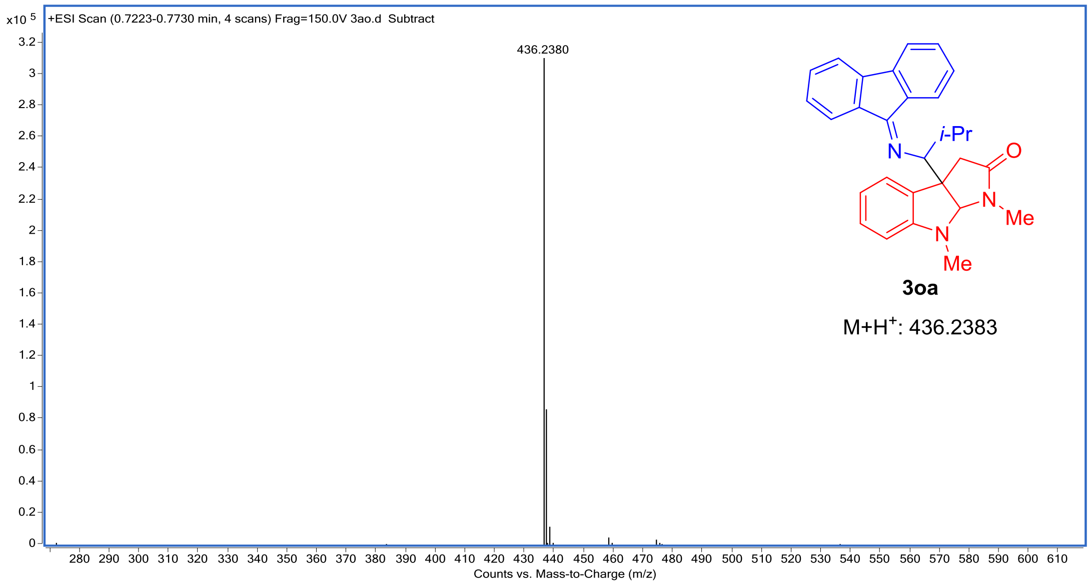
x10 5
+ESI Scan (0.7223-0.7730 min, 4 scans) Frag=150.0V 3ao.d Subtract
436.2380
i-Pr
N
O
N
Me
N
Me
3oa
M+H+: 436.2383
Counts vs. Mass-to-Charge (m/z)

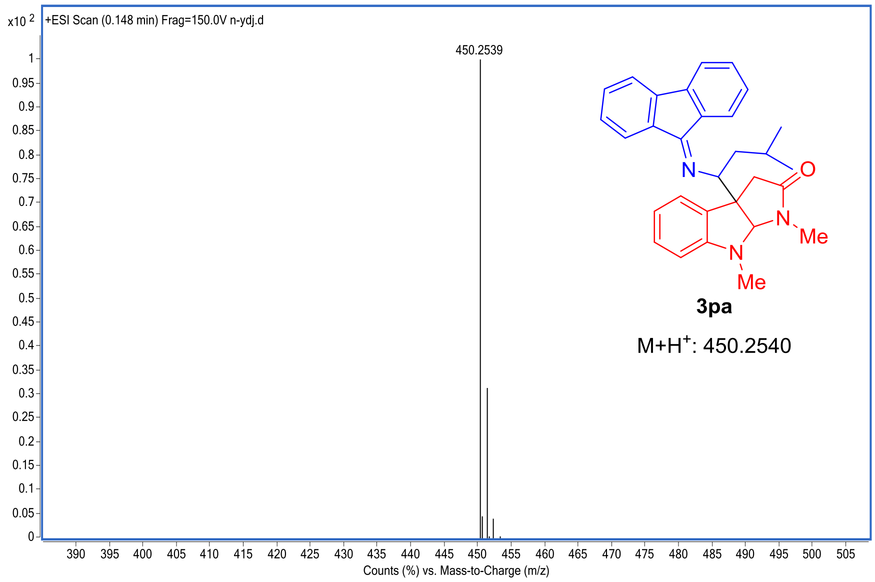

x10 2
+ESI Scan (0.148 min) Frag=150.0V n-ydj.d
450.2539
3pa
M+H+: 450.2540
Counts (%) vs. Mass-to-Charge (m/z)

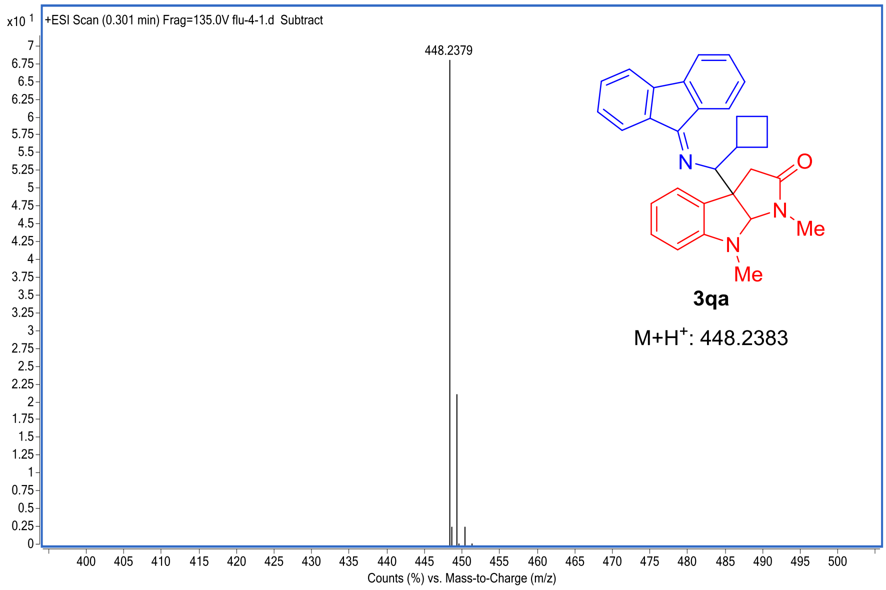

x10 1
+ESI Scan (0.301 min) Frag=135.0V flu-4-1.d Subtract
448.2379
3qa
M+H+: 448.2383
Counts (%) vs. Mass-to-Charge (m/z)

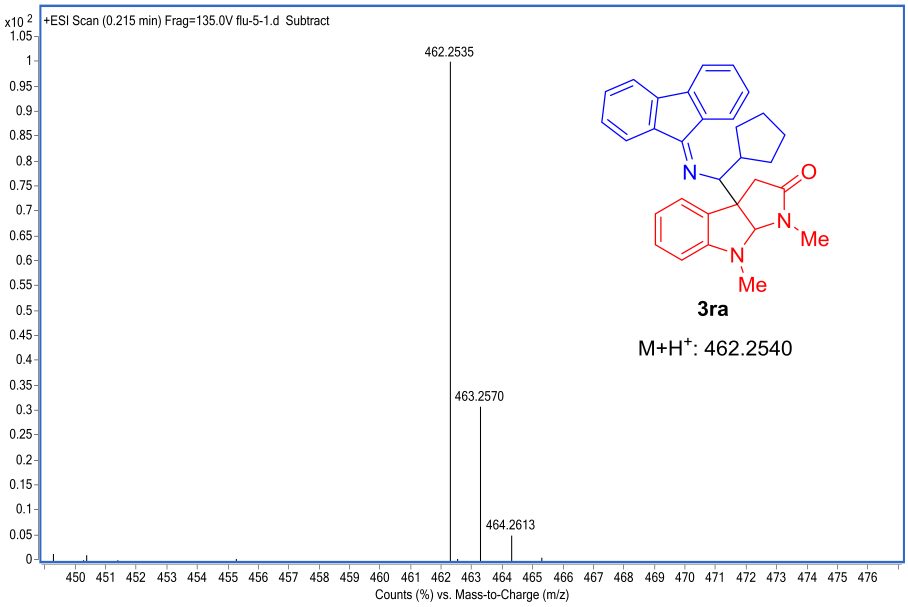

x10 2
+ESI Scan (0.215 min) Frag=135.0V flu-5-1.d Subtract
462.2535
463.2570
464.2613
3ra
M+H+: 462.2540
Counts (%) vs. Mass-to-Charge (m/z)

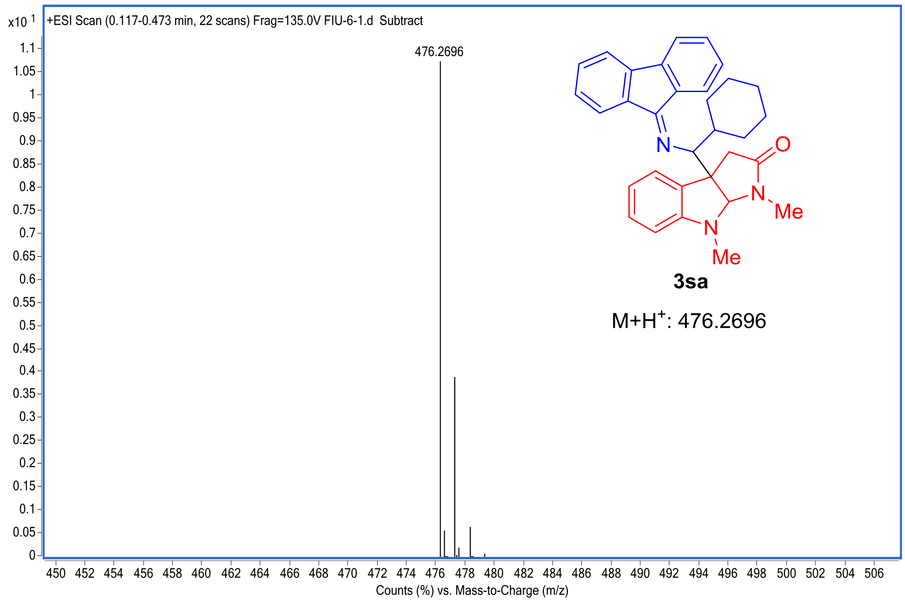
x10 1
+ESI Scan (0.117-0.473 min, 22 scans) Frag=135.0V FIU-6-1.d Subtract
476.2696
3sa
M+H+: 476.2696
Counts (%) vs. Mass-to-Charge (m/z)

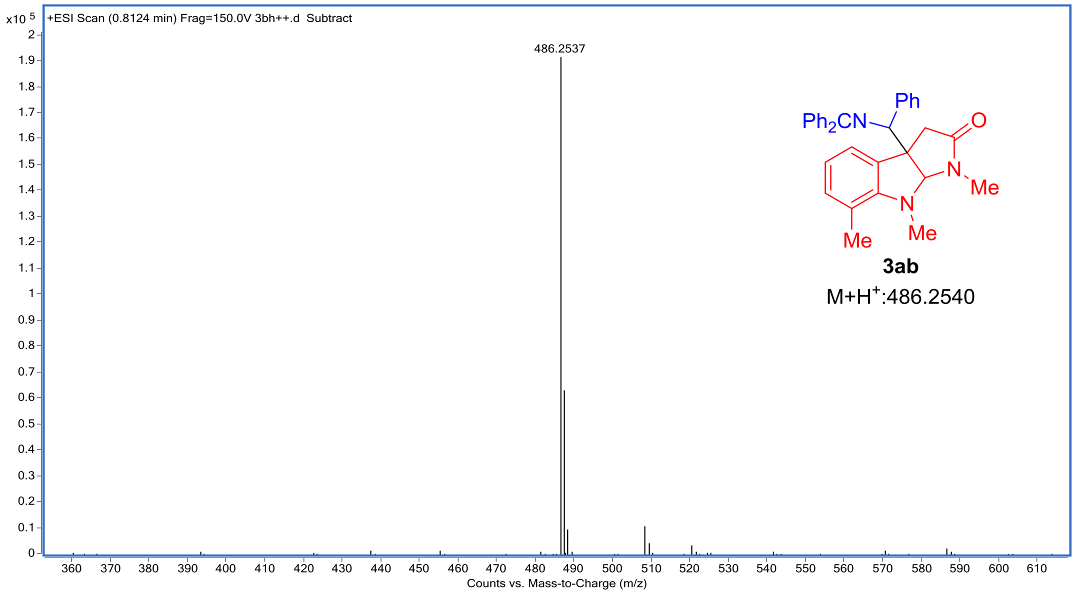
x10 5
+ESI Scan (0.8124 min) Frag=150.0V 3bh++.d Subtract
486.2537
Ph2CN
Ph
3ab
M+H+:486.2540
Counts vs. Mass-to-Charge (m/z)

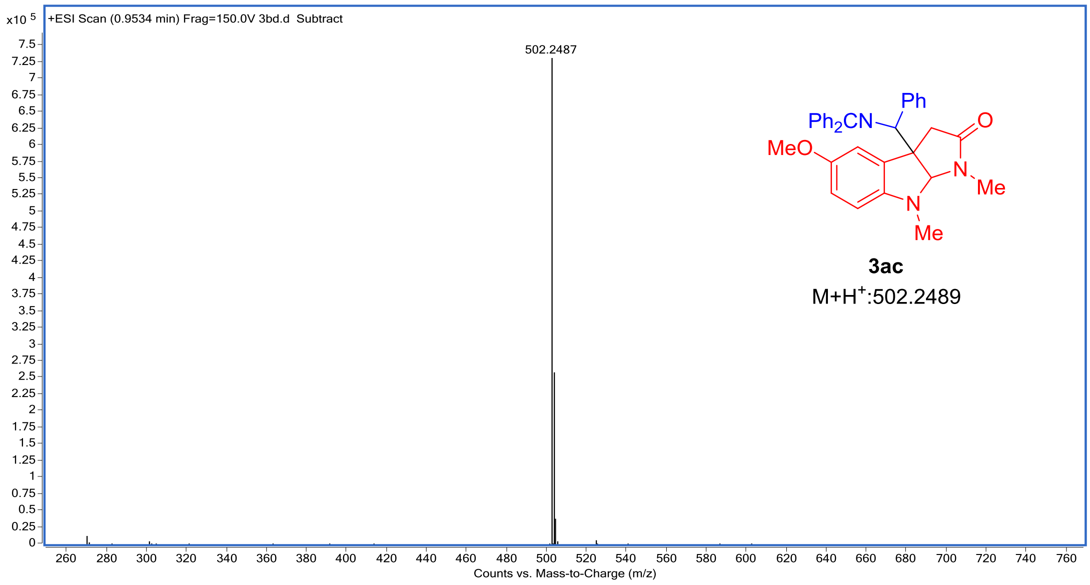
x10 5
+ESI Scan (0.9534 min) Frag=150.0V 3bd.d Subtract
502.2487
Ph2CN
Ph
MeO
3ac
M+H+:502.2489
Counts vs. Mass-to-Charge (m/z)

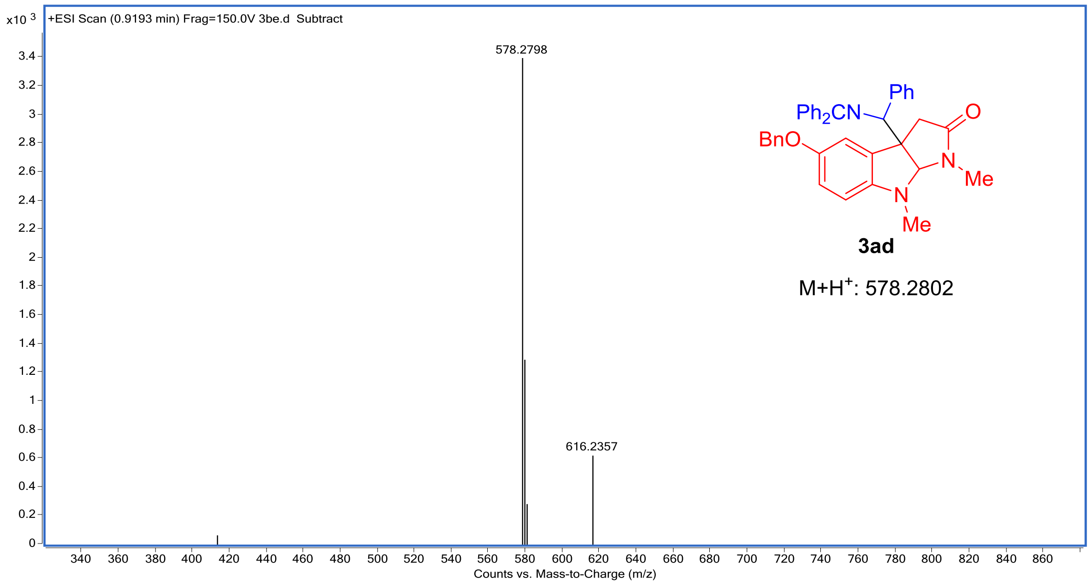
x10 3
+ESI Scan (0.9193 min) Frag=150.0V 3be.d Subtract
578.2798
616.2357
Counts vs. Mass-to-Charge (m/z)
3ad
M+H+: 578.2802

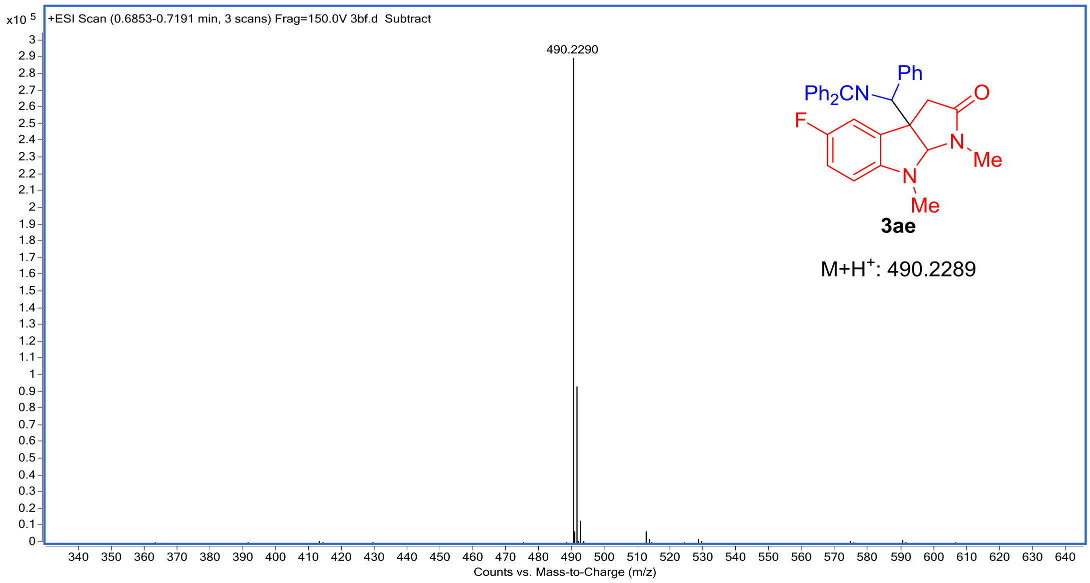
x10 5
+ESI Scan (0.6853-0.7191 min, 3 scans) Frag=150.0V 3bf.d Subtract
490.2290
Counts vs. Mass-to-Charge (m/z)
3ae
M+H+: 490.2289

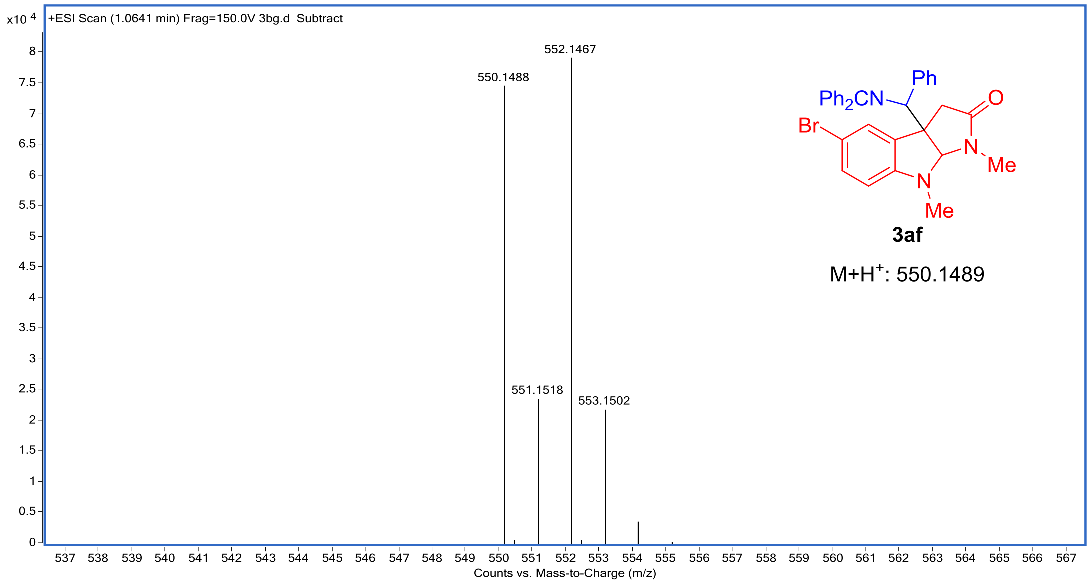
x10 4
+ESI Scan (1.0641 min) Frag=150.0V 3bg.d Subtract
552.1467
550.1488
551.1518
553.1502
Counts vs. Mass-to-Charge (m/z)
3af
M+H+: 550.1489

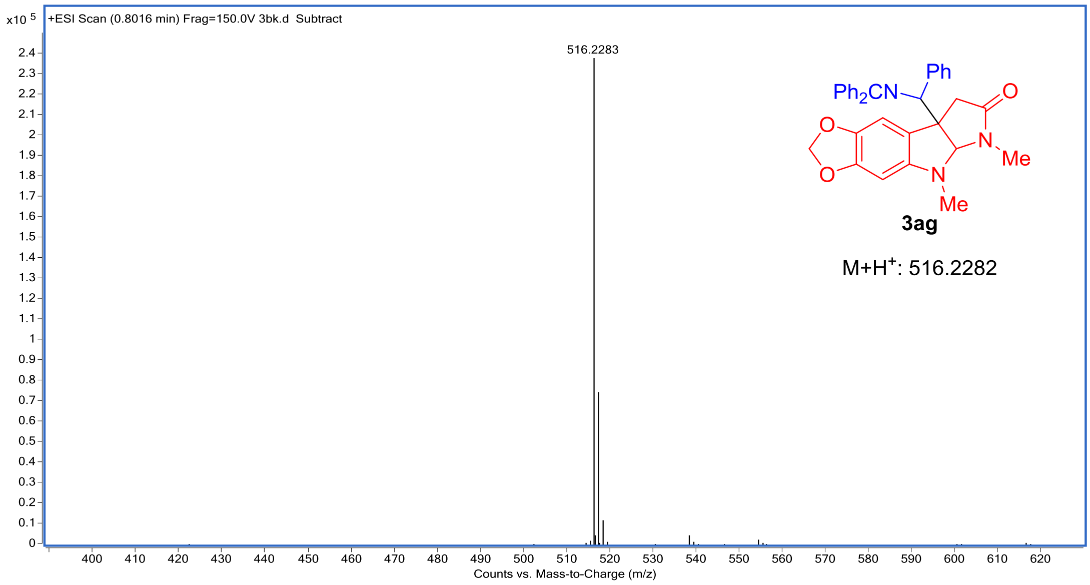
+ESI Scan (0.8016 min) Frag=150.0V 3bk.d Subtract
516.2283
Counts vs. Mass-to-Charge (m/z)
3ag
M+H+: 516.2282

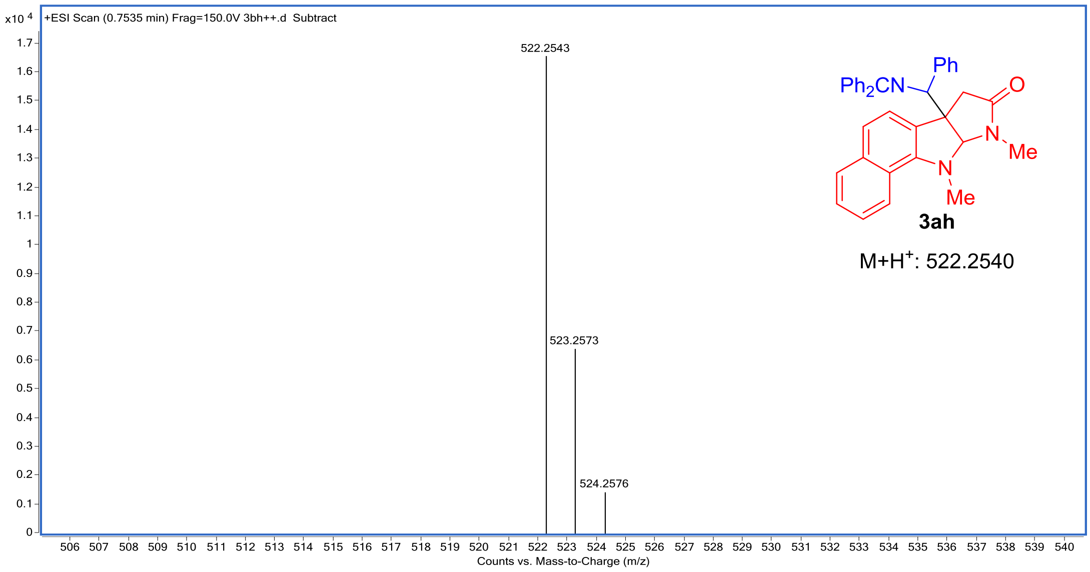
+ESI Scan (0.7535 min) Frag=150.0V 3bh++.d Subtract
522.2543
523.2573
524.2576
Counts vs. Mass-to-Charge (m/z)
3ah
M+H+: 522.2540

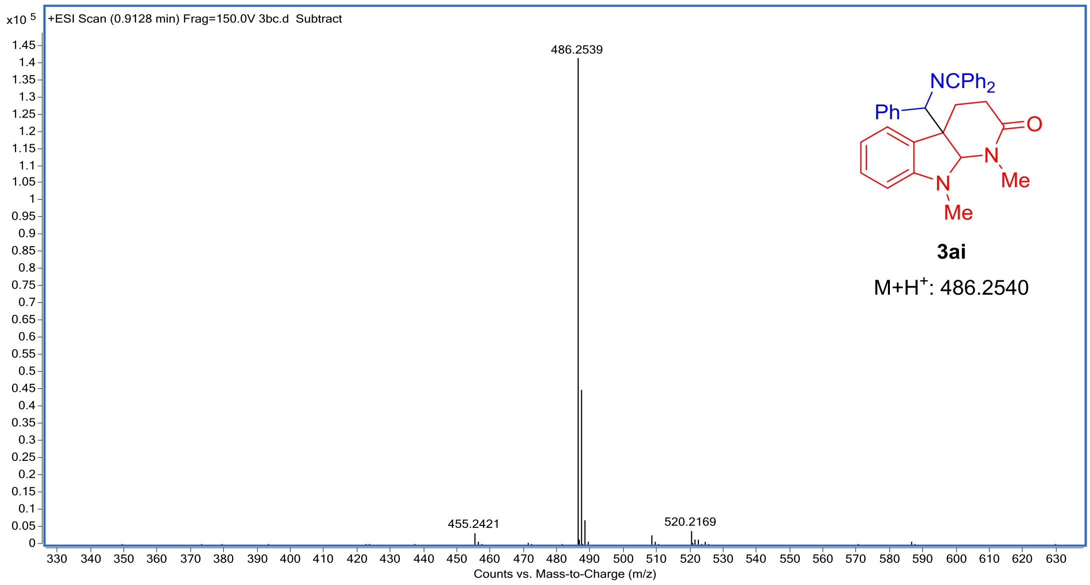
+ESI Scan (0.9128 min) Frag=150.0V 3bc.d Subtract
486.2539
455.2421
520.2169
Counts vs. Mass-to-Charge (m/z)
3ai
M+H+: 486.2540

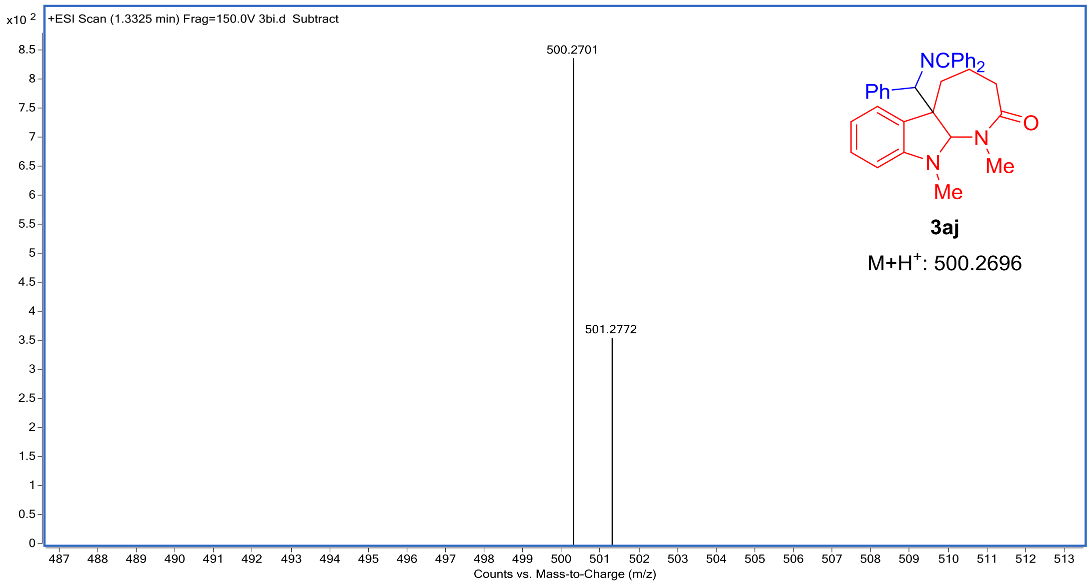
+ESI Scan (1.3325 min) Frag=150.0V 3bi.d Subtract
x10 2
500.2701
501.2772
Counts vs. Mass-to-Charge (m/z)
NCPh2
Ph
O
N
Me
3aj
M+H+: 500.2696

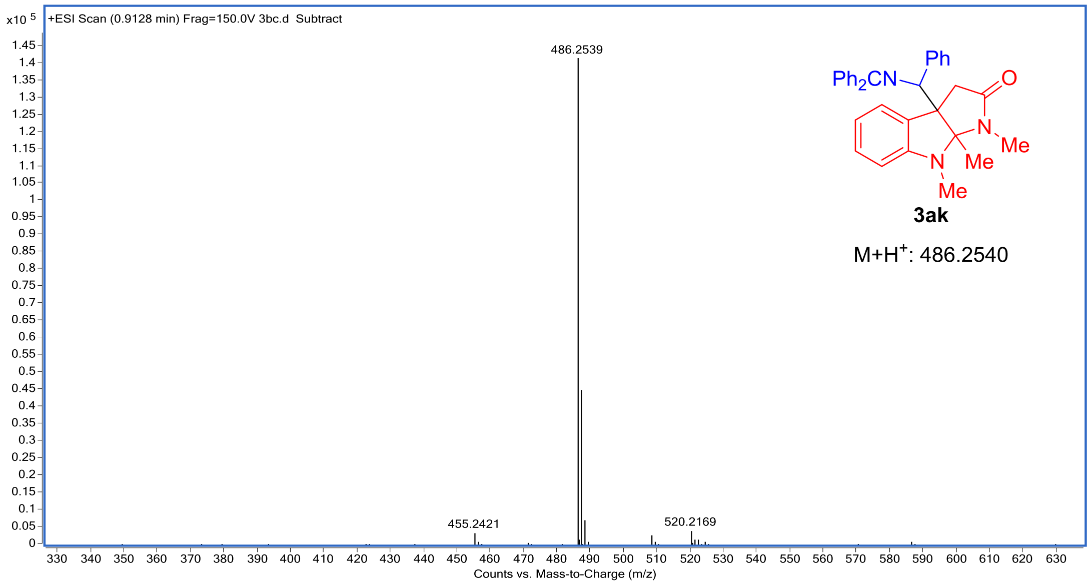
+ESI Scan (0.9128 min) Frag=150.0V 3bc.d Subtract
x10 5
486.2539
455.2421
520.2169
Counts vs. Mass-to-Charge (m/z)
Ph
Ph2CN
O
N
Me
3ak
M+H+: 486.2540

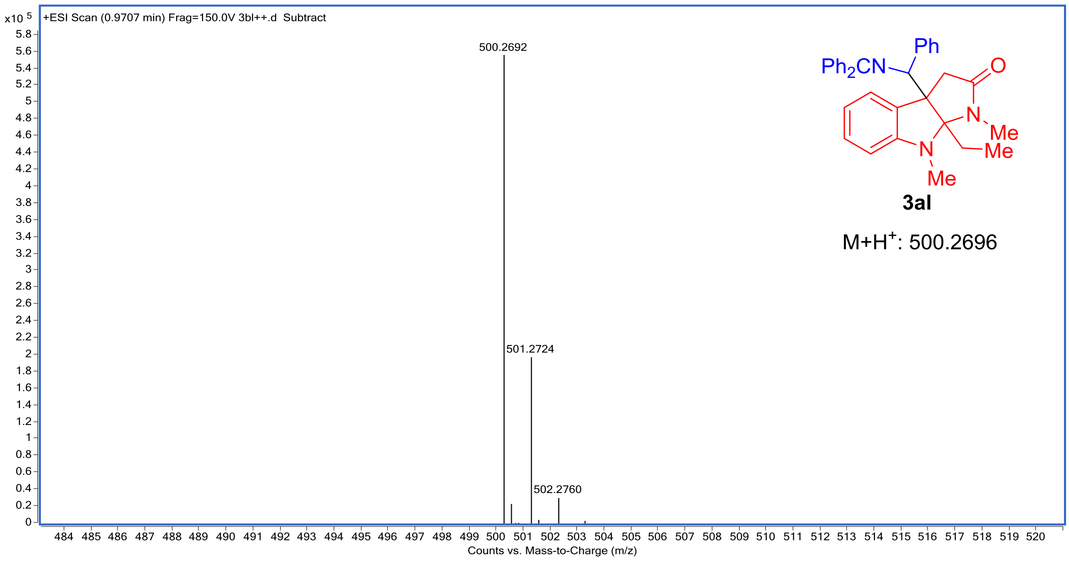
+ESI Scan (0.9707 min) Frag=150.0V 3bl++.d Subtract
x10 5
500.2692
501.2724
502.2760
Counts vs. Mass-to-Charge (m/z)
Ph
Ph2CN
O
N
Me
3al
M+H+: 500.2696

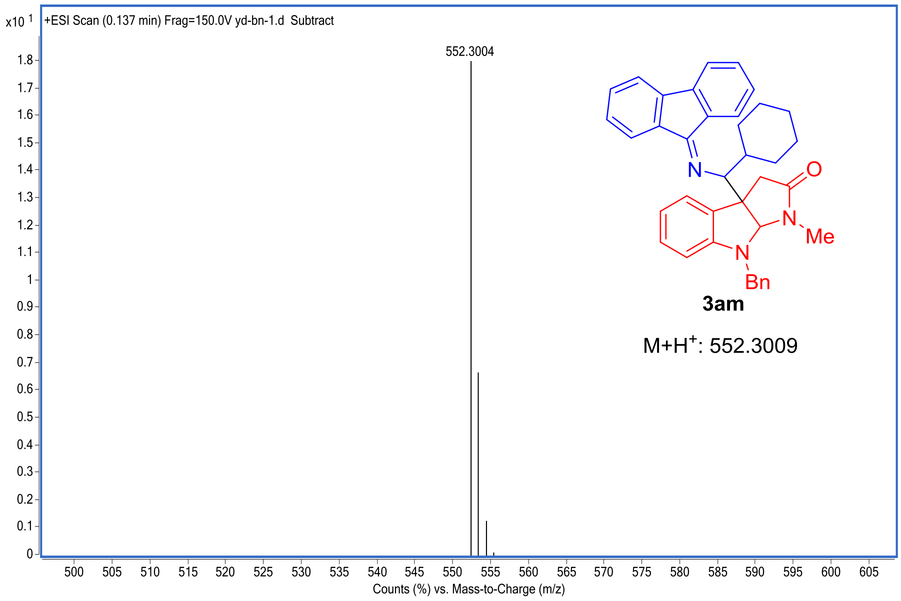
x10 1
+ESI Scan (0.137 min) Frag=150.0V yd-bn-1.d Subtract
552.3004
N
O
N
Me
N
Bn
3am
M+H+: 552.3009
Counts (%) vs. Mass-to-Charge (m/z)

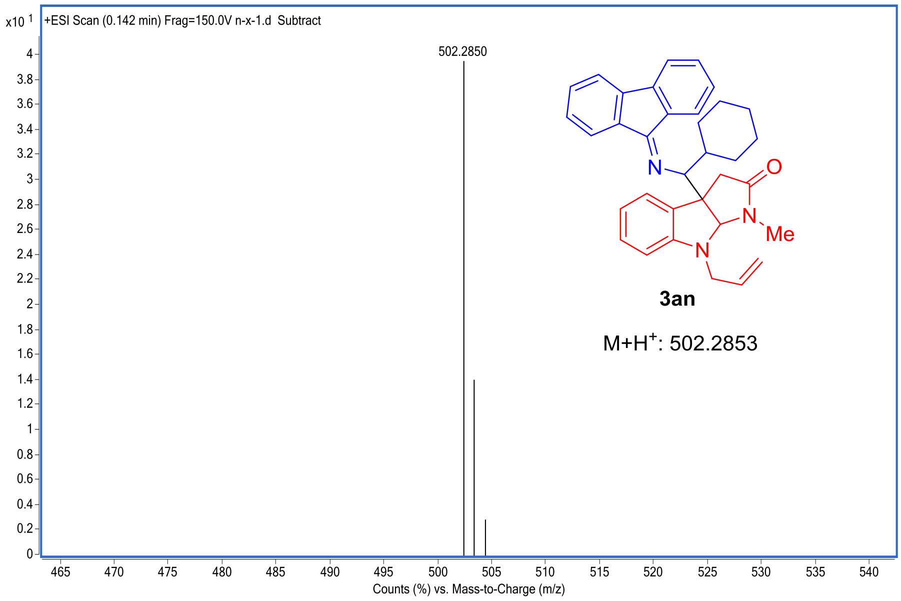
x10 1
+ESI Scan (0.142 min) Frag=150.0V n-x-1.d Subtract
502.2850
N
O
N
Me
N
3an
M+H+: 502.2853
Counts (%) vs. Mass-to-Charge (m/z)

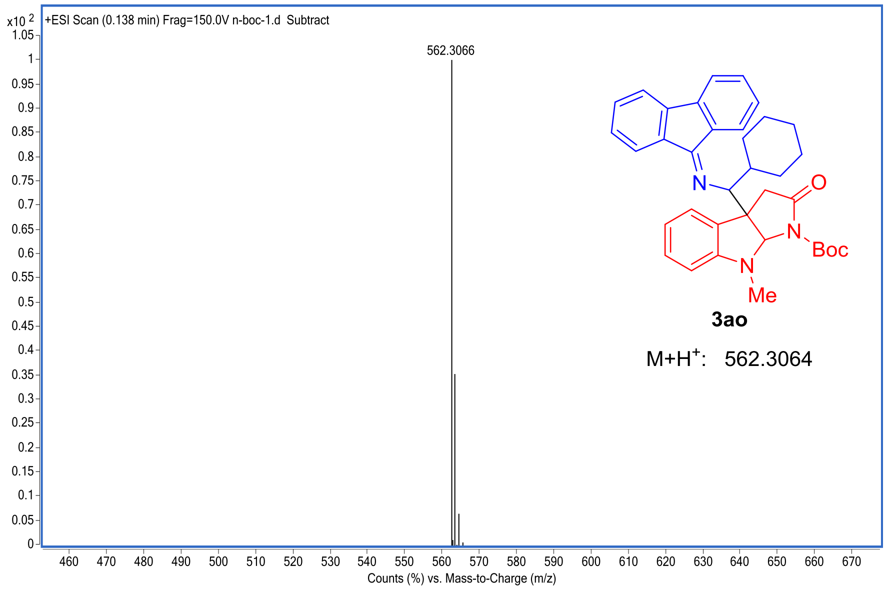
x10 2
+ESI Scan (0.138 min) Frag=150.0V n-boc-1.d Subtract
562.3066
N
O
N
Boc
N
Me
3ao
M+H+: 562.3064
Counts (%) vs. Mass-to-Charge (m/z)

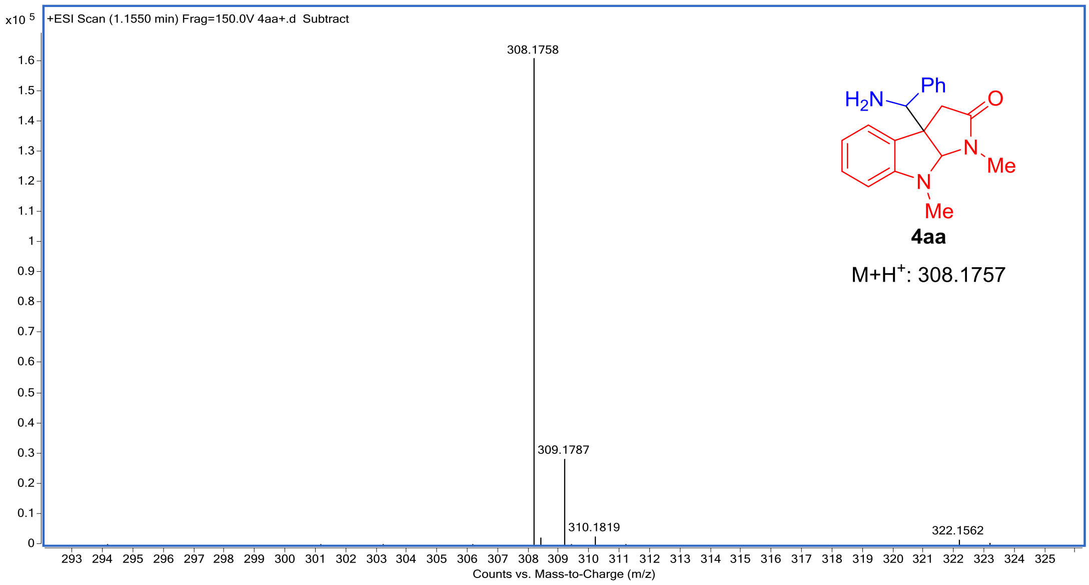

x10 5
+ESI Scan (1.1550 min) Frag=150.0V 4aa+.d Subtract
308.1758
309.1787
310.1819
322.1562
Counts vs. Mass-to-Charge (m/z)
H2N
Ph
O
N
Me
N
Me
4aa
M+H+: 308.1757
